# Pd-catalyzed access to mono- and di-fluoroallylic amines from primary anilines†

**DOI:** 10.1039/d2cc05844h

**Published:** 2022-12-16

**Authors:** Xingben Wang, Frederic W. Patureau

**Affiliations:** a Institute of Organic Chemistry, RWTH Aachen University Landoltweg 1 Aachen 52074 Germany Frederic.patureau@rwth-aachen.de https://www.patureau-oc-rwth-aachen.de

## Abstract

The Pd-catalyzed highly selective synthesis of mono- and di-2-fluoroallylic amines from *gem*-difluorocyclopropanes and ubiquitous unprotected primary anilines is herein described. Initial kinetic investigations suggest a first order in the *gem*-difluorocyclopropane substrate, as well as a *circa* zeroth order in the aniline coupling partner. The newly produced fluoroallylic motifs should find important applications in synthetic as well as medicinal chemistry and stimulate the further development of coupling methods based on strained cyclic building blocks.

## Abstract 2

The Pd-catalyzed highly selective synthesis of mono- and di-2-fluoroallylic amines is herein described from *gem*-difluorocyclopropanes and simple primary anilines.

## Graphical abstract

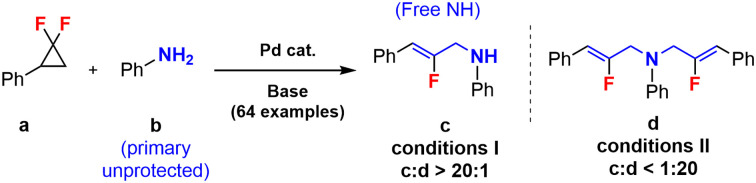

Allylamines are important structures found in both natural products and biologically active molecules.^1–3^ This is the case for example in naftifine,^4,5^ an antifungal drug, or flunarizine,^6,7^ a calcium blocker. Meanwhile, research and development of fluorine-containing drugs^8–12^ have considerably expanded in the last 20 years. This is because fluorine incorporation can impose remarkable electronic, physical and biological properties on organic compounds. Thus, we envisioned that developing simple synthetic methods granting access to fluorinated allylamines would represent an important objective in order to develop new potent drugs, or fluorinated variants of the existing ones. It is noteworthy that the synthesis of allylamine structures with transition metal catalysts^13–22^ is a frequently utilized and efficient approach. However, the synthesis of their fluorinated equivalents is starkly underdeveloped, and constitutes a major challenge. In this context, *gem*-difluorocyclopropanes^23–25^ are composed of a fluorine-containing strained three-membered ring structure, and are easily accessible (see the ESI†). Moreover, these motifs can be readily activated at the C–C and C–F bonds under transition metal catalysis, such as with Pd,^26–36^ Rh,^37^ or other metals, usually leading to the corresponding fluorinated allylic metal complexes. These organometallic intermediates can then be utilized to make fluorine-containing allylamide and allylamine compounds, when combined with N-derived nucleophiles. This was elegantly demonstrated by the seminal work of Fu and coauthors in 2015 (Scheme 1a).^26^ We propose herein such a C–N bond forming method, however with unprecedented primary aniline N-nucleophiles, which unfortunately perform very poorly under the method described by Fu,^26^ either towards the mono-, or alternatively towards the di-fluoroallylic amines, selectively (Scheme 1b). This strategy moreover represents a different retrosynthetic disconnection compared to the Ir-catalyzed photochemical C–C coupling method of Hashmi and collaborators (Scheme 1a),^38^ and allows for the direct synthesis of elusive NH-unprotected fluoroallylic anilines.

**Scheme 1:**
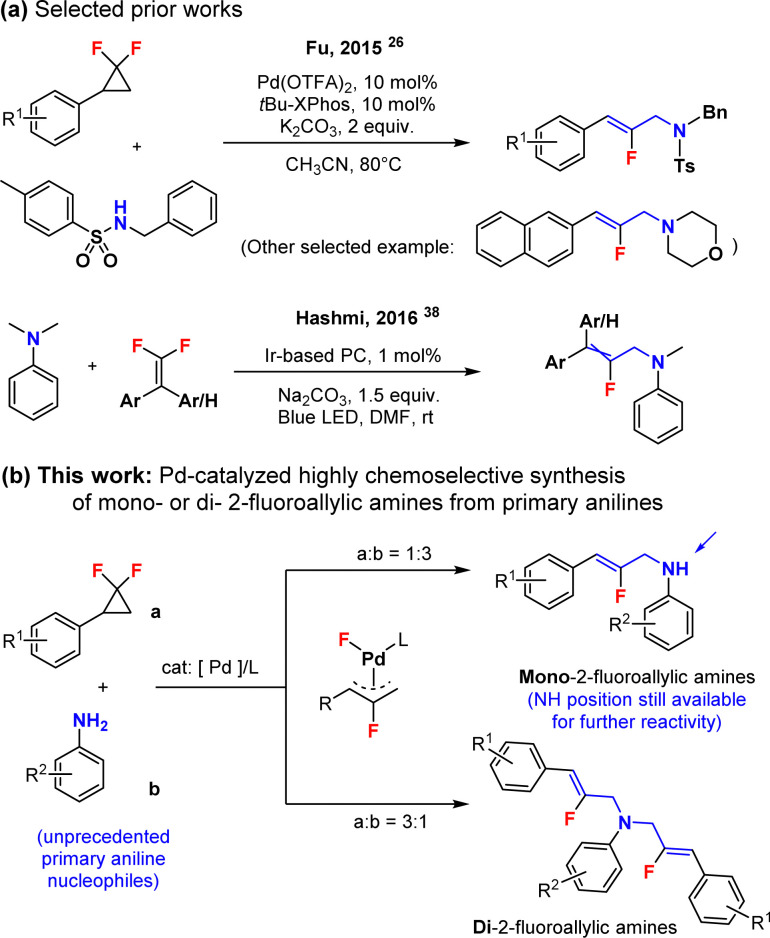
Towards fluorinated allylamines, (a) selected prior works, (b) this work.

Therefore, 4-methyl *gem*-difluorocyclopropane a1 (1-(2,2-difluorocyclopropyl)-4-methylbenzene) and simple aniline (b1) were originally selected as model substrates. Setting the a1 : b1 ratio to 1 : 1, under Pd(dba)_2_ catalysis (5 mol%), with XPhos (12.5 mol%) and K_3_PO_4_ (2 equiv.) in *p*-xylene at 110 °C for 12 h afforded the mono-functionalized product c1 in 29% yield. Under these conditions, the di-functionalized product d1 was obtained in 4% yield (entry 1, Table S1, see the ESI†). Increasing the amount of aniline b1 afforded the mono-functionalized 2-fluoroallylic amine product c1 in an impressive 89% isolated yield, with a selectivity as high as 20 : 1 (a1 : b1 ratio of 1 : 3, entries 2 and 3). The higher XPhos/Pd catalytic ratio was moreover found necessary, as reducing the XPhos loading to only 5 mol% almost shuts down the reaction (entry 4). In contrast to entry 3, an a1 : b1 ratio of 3 : 1 afforded the new di-2-fluoroallylic amine substance d1 in an impressive 90% isolated yield and also high chemical selectivity (20 : 1, entries 5 and 6). Finally, we verified that the original reaction conditions reported by Fu^26^ (Scheme 1b) do not provide product c1 or d1 in any significant yield regardless of the substrate ratio (entries 7 and 8), thus validating the superiority of our method for the challenging primary anilines.

In terms of scope, many functional groups were found well tolerated in very diverse positions (c1–c28), with typically excellent yields and mono-selectivity (usually *m* : *d* > 20 : 1, Scheme 2). Moreover, a broad series of bioactive moieties (c29–c33) were well accommodated, with high isolated yields (26–99%) and good to excellent mono-selectivity (*m* : *d* 7 : 1 to 20 : 1) in some cases. In the low yielding case of c30, the substrate ratio was adjusted to 1 : 1 in order to minimize di-functionalization as well as other undefined side products. These results arguably demonstrate the high relevance of the herein described synthetic method, in spite of some other substrate limitations such as amide or aliphatic amine N-nucleophiles (c34–c38). This includes Fu's typical N-nucleophile,^26^ which did not efficiently furnish product c39. In such cases, some unreacted *gem*-difluorocyclopropane is typically observed at the end of the reaction. This indicates the high specificity of our method for anilines.

**Scheme 2:**
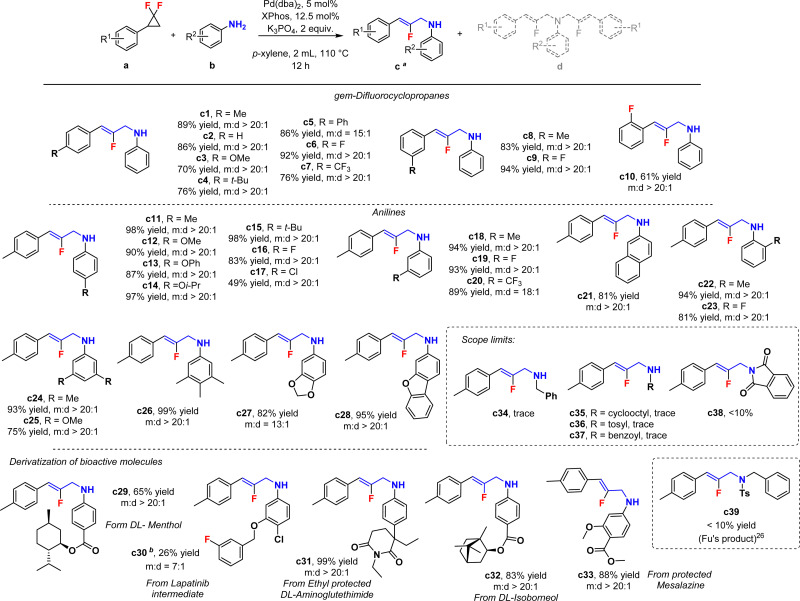
Substrate scope for the mono-2-fluoroallylic amines, isolated yields. ^*a*^The *m* : *d* ratios are determined by ^1^H NMR. Reaction conditions: a (0.20 mmol), b (0.60 mmol), Pd catalyst (5 mol%) with XPhos ligand (12.5 mol%), K_3_PO_4_ (2 equiv.) in *p*-xylene (2.0 mL) at 110 °C for 12 h. ^*b*^a : b = 1 : 1.

We then turned our attention to the new di-2-fluoroallylic amines and their synthesis (Scheme 3, d1–d17), affording generally good yields and selectivities (*d* : *m* > 20 : 1). Interestingly, even sterically hindered 2-methyl aniline converted to the di-functionalized product (d18), although with a reaction time extended to 24 h, affording 82% yield and an encouraging di-selectivity (*d* : *m* = 9 : 1). In addition, multifunctional and heterocyclic anilines were also found to be competent (d19–d24), in addition to a broad range of bioactive and natural fragments (d25–d29). In order to further explore the synthetic utility of the method, a gram scale reaction was conducted for mono-functionalized product c1 (Scheme 4). This target was thus obtained in a remarkably preserved 80% isolated yield (0.96 g), with high mono-selectivity (*m* : *d* > 20 : 1). Furthermore, we verified that diphenylamine reacts in a similar fashion to the primary anilines described in this study, with 1a, affording indeed 95% isolated yield of the corresponding product (e1). Moreover, scaffold e1 could also be obtained from c1 in likewise excellent 95% yield with a classical Buchwald–Hartwig coupling reaction.^39^ Finally, unsymmetrical di-2-fluoroallylic product d30, with two different allylic arms, could be accessed from c1 reacting with a different *gem*-difluorocyclopropane, under otherwise similar reaction conditions (64%, Scheme 4). This demonstrates the feasibility of attaching two different fluoroallyl functional groups on primary anilines in a sequential fashion.

**Scheme 3:**
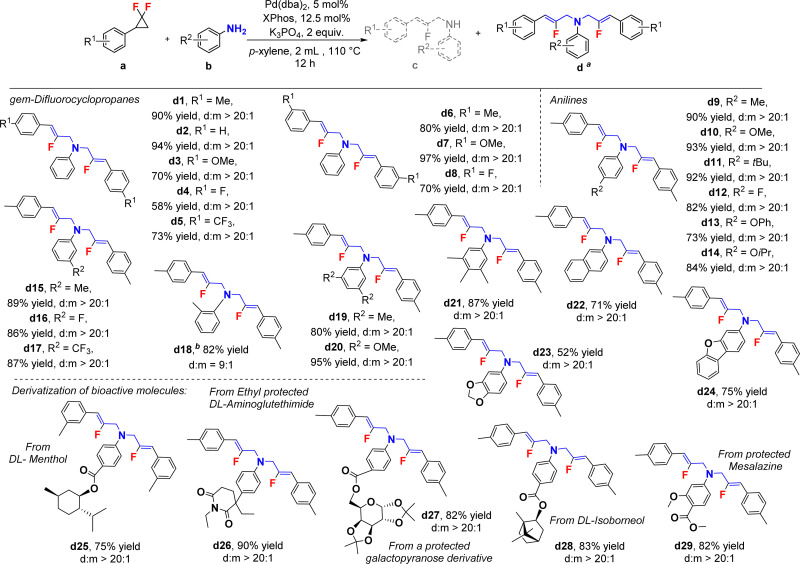
Substrate scope for the di-2-fluoroallylic amines, isolated yields. ^*a*^The d : m ratios are determined by ^1^H NMR. Reaction conditions: a (0.60 mmol), b (0.20 mmol), Pd catalyst (5 mol %) with XPhos ligand (12.5 mol%), K_3_PO_4_ (2 equiv.) in *p*-xylene (2.0 mL) at 110 °C for 12 h. ^*b*^Reaction time: 24 h.

**Scheme 4:**
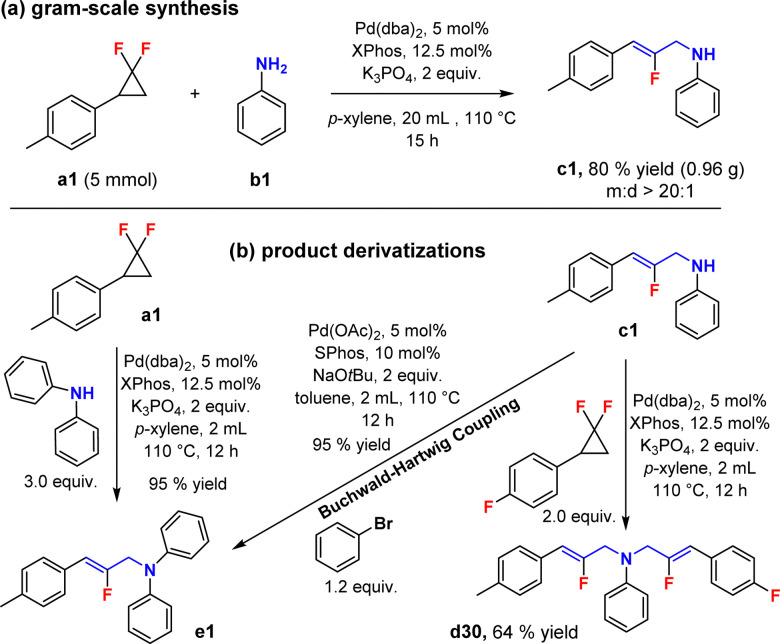
Synthetic utility (a) and further developments (b).

Based on previous literature,^26^ we envisioned a possible mechanism as outlined in Scheme 5. First Pd(0) would activate the strained C–C bond of the *gem*-difluorocyclopropane to form intermediate I, followed by β-F elimination^40^ to give π-allylpalladium species II. Intermediate II would then be attacked by the N-nucleophile to give species III. Finally, the product would be obtained by C–N bond reductive elimination, thus regenerating the Pd(0) active catalyst. Experimentally, we observed first order kinetics with respect to the *gem*-difluorocyclopropane building block a1 in the 0.05 to 0.40 M concentration range. In contrast, the reaction has an approximatively zeroth order in aniline substrate b1 (see the ESI† for details). These results suggest an early rate limiting step, such as the strained C–C bond activation, or the subsequent β-F elimination step towards intermediate II.

**Scheme 5:**
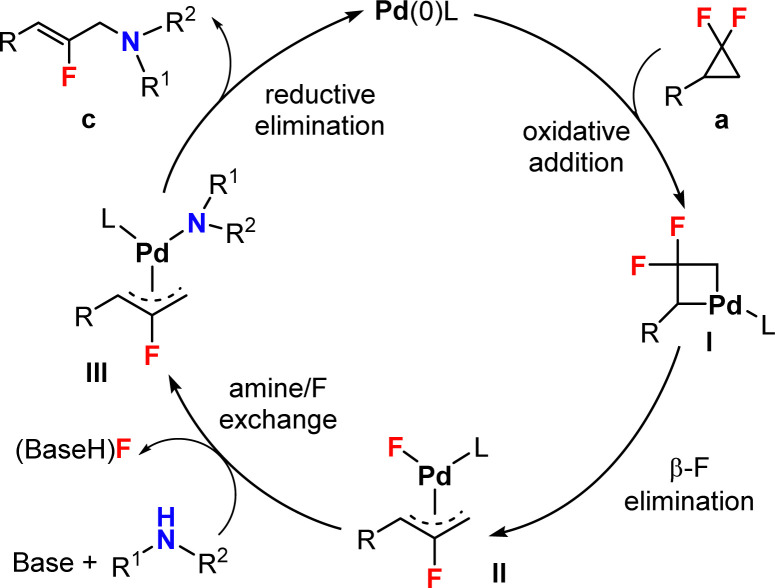
Proposed mechanism.

In summary, we have developed a Pd-catalyzed highly selective synthesis of mono- and di- 2-fluoroallylic amines from primary anilines and *gem*-difluorocyclopropanes. In addition to the newly opened chemical space in terms of potentially interesting fluorinated drug candidates, these results should encourage the further development of cross coupling methods based on the very versatile *gem*-difluorocyclopropane^41–43^ and related strained building blocks.

The ERC project 716136:2O2ACTIVATION (FWP) and the Chinese Scholarship Council (XW, No. 202008330337) are gratefully acknowledged for financial support.

## Conflicts of interest

There are no conflicts to declare.

## Supplementary Material

CC-059-D2CC05844H-s001

## References

[cit1] Roughley S. D., Jordan A. M. (2011). J. Med. Chem..

[cit2] Trowbridge A., Walton S. M., Gaunt M. J. (2020). Chem. Rev..

[cit3] Vitaku E., Smith D. T., Njardarson J. T. (2014). J. Med. Chem..

[cit4] Petranyi G., Ryder N. S., Stütz A. (1984). Science.

[cit5] Kanno H., Taylor R. J. K. (2002). Tetrahedron Lett..

[cit6] Olesen J. (1991). J. Neurol..

[cit7] Marın M. T., Margarit M. V., Salcedo G. E. (2002). Il Farmaco.

[cit8] Mei H., Han J., Fustero S., Medio-Simon M., Sedgwick D. M., Santi C., Ruzziconi R., Soloshonok V. A. (2019). Chem. – Eur. J..

[cit9] Chen H., Viel S., Ziarelli F., Peng L. (2013). Chem. Soc. Rev..

[cit10] Yoder N. C., Kumar K. (2002). Chem. Soc. Rev..

[cit11] Salwiczek M., Nyakatura E. K., Gerling U. I. M., Ye S., Koksch B. (2012). Chem. Soc. Rev..

[cit12] Marsh E. N. G. (2014). Acc. Chem. Res..

[cit13] Ohshima T., Miyamoto Y., Ipposhi J., Nakahara Y., Utsunomiya M., Mashima K. (2009). J. Am. Chem. Soc..

[cit14] Das K., Shibuya R., Nakahara Y., Germain N., Ohshima T., Mashima K. (2012). Angew. Chem., Int. Ed..

[cit15] Knöfel N. D., Rothfuss H., Willenbacher J., Barner-Kowollik C., Roesky P. W. (2017). Angew. Chem., Int. Ed..

[cit16] Šolić I., Reich D., Lim J., Bates R. W. (2017). Asian J. Org. Chem..

[cit17] Ozawa F., Okamoto H., Kawagishi S., Yamamoto S., Minami T., Yoshifuji M. (2002). J. Am. Chem. Soc..

[cit18] Sawadjoon S., Samec J. S. M. (2011). Org. Biomol. Chem..

[cit19] Bahena E. N., Griffin S. E., Schafer L. L. (2020). J. Am. Chem. Soc..

[cit20] Cai A., Guo W., Martínez-Rodríguez L., Kleij A. W. (2016). J. Am. Chem. Soc..

[cit21] Skucas E., Ngai M. Y., Komanduri V., Krische M. J. (2007). Acc. Chem. Res..

[cit22] Ali S. Z., Budaitis B. G., Fontaine D. F. A., Pace A. L., Garwin J. A., White M. C. (2022). Science.

[cit23] Seyferth D., Dertouzos H., Suzuki R., Mui J. Y. P. (1967). J. Org. Chem..

[cit24] Thankachan A. P., Sindhu K. S., Krishnan K. K., Anilkumar G. (2015). Org. Biomol. Chem..

[cit25] Volochnyuk D. M. and Grygorenko O. O., Emerging Fluorinated Motifs: Synthesis, Properties, and Applications, ed. D. Cahard and J.-A. Ma, Wiley, 2020, vol. 1, p. 135

[cit26] Xu J., Ahmed E. A., Xiao B., Lu Q. Q., Wang Y. L., Yu C. G., Fu Y. (2015). Angew. Chem., Int. Ed..

[cit27] Lv L., Qian H., Ma Y., Huang S., Yan X., Li Z. (2021). Chem. Sci..

[cit28] Wenz J., Rettenmeier C. A., Wadepohl H., Gade L. H. (2016). Chem. Commun..

[cit29] Ahmed E. A. M. A., Suliman A. M. Y., Gong T. J., Fu Y. (2019). Org. Lett..

[cit30] Ahmed E. A. M. A., Suliman A. M. Y., Gong T. J., Fu Y. (2020). Org. Lett..

[cit31] Suliman A. M. Y., Ahmed E. A. M. A., Gong T. J., Fu Y. (2021). Org. Lett..

[cit32] Zhou P. X., Yang X., Wang J., Ge C., Feng W., Liang Y. M., Zhang Y. (2021). Org. Lett..

[cit33] Suliman A. M. Y., Ahmed E. A. M. A., Gong T. J., Fu Y. (2021). Chem. Commun..

[cit34] Lv L., Li C. J. (2021). Angew. Chem., Int. Ed..

[cit35] Fu Z., Zhu J., Guo S., Lin A. (2021). Chem. Commun..

[cit36] Xiong B., Chen X., Liu J., Zhang X., Xia Y., Lian Z. (2021). ACS Catal..

[cit37] Jiang Z. T., Huang J., Zeng Y., Hu F., Xia Y. (2021). Angew. Chem., Int. Ed..

[cit38] Xie J., Yu J., Rudolph M., Rominger F., Hashmi A. S. K. (2016). Angew. Chem., Int. Ed..

[cit39] Topchiy M. A., Asachenko A. F., Nechaev M. S. (2014). Eur. J. Org. Chem..

[cit40] Fujita T., Fuchibe K., Ichikawa J. (2019). Angew. Chem., Int. Ed..

[cit41] Zhu Y., Zeng Y., Jiang Z.-T., Xia Y. (2022). Synlett.

[cit42] Lv L., Qian H., Crowell A. B., Chen S., Li Z. (2022). ACS Catal..

[cit43] Yuan W., Li X., Qi Z., Li X. (2022). Org. Lett..

