# Supplementary material for: Pd-catalyzed access to mono- and di-fluoroallylic amines from primary anilines
Source: Chem Commun (Camb). 2022 Dec 16;59(4):486–9. doi: 10.1039/d2cc05844h (PMC9814328; doi:10.1039/d2cc05844h)
Supplement: CC-059-D2CC05844H-s001 [file CC-059-D2CC05844H-s001.pdf]

Electronic Supplementary Material (ESI) for Chemical Communications.
This journal is © The Royal Society of Chemistry 2022

Supporting Information

Pd-catalyzed access to mono- and di-fluoroallylic amines from primary anilines

Xingben Wang, Frederic W. Patureau *

Institute of Organic Chemistry, RWTH Aachen University, Landoltweg 1, 52074 Aachen, Germany.
E-mail: Frederic.Patureau@rwth-aachen.de

Table of Contents

1. General information (S2)
2. Optimization (S2)
3. Preparation of some starting materials (S3)
4. General procedure for **c** (S3)
5. Product characterization of **c** (S3)
6. General procedure for **d** (S17)
7. Product characterization of **d** (S18)
8. Transformation of products (S30)
9. Kinetic orders (S32)
10. References (S34)
11. Copies of ^{1}H, ^{13}C, and ^{19}F spectra (S35)

1. General information

NMR spectra were obtained on an Agilent VNMRS 400 or a Bruker Av 600 using $CDCl_3$ as solvents. Chemical shifts are given in ppm and coupling constants (*J*) in Hz. ^{1}H spectra were calibrated in relation to the reference measurement of TMS (0.00 ppm). ^{13}C spectra were calibrated in relation to the deuterated solvent, namely $CDCl_3$ (77.16 ppm). The following abbreviations were used for ^{1}H NMR spectra to indicate the signal multiplicity: s (singlet), d (doublet), t (triplet), q (quartet) and m (multiplet) as well as combinations of them. Flash chromatography was performed on silica gel (60 M, 0.04-0.063 mm) by standard technique. All the chemicals used for synthesis were purchased from Sigma Aldrich, abcr, Alfa Aesar, TCI, Fisher, or chemPUR. $Pd(dba)_2$ was purchased from Sigma Aldrich. High resolution mass spectra (HRMS) were obtained on a Thermo Scientific LTQ Orbitrap XL spectrometer.

2. Optimization

Table S1 Optimization towards mono- or di-2-fluoroallylic amines.[a]

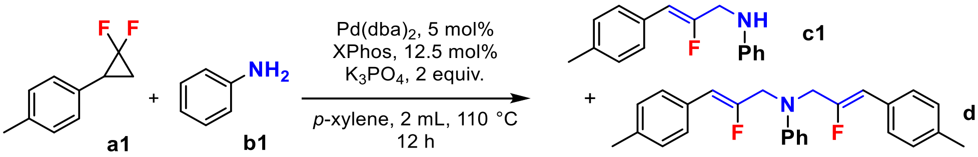

Entry	**a1**:**b1**	**c1** yield (%) [b]	**d1** yield (%) [b]	**c1**:**d1** [b]
1	1:1	29	4	7.25:1
2	1:2	54	2	>20:1
3	1:3	90(89)[c]	2	>20:1
4 [d]	1:3	< 10	-	-
5	2:1	1	75	<1:20
6	3:1	1	90(90)[c]	<1:20
7 [e]	1:2	18	<5	-
8 [e]	3:1	6	<5	-

[a] Reaction conditions: **a1** (0.20 mmol), **b1** (0.20 mmol), catalyst (5 mol%), XPhos ligand (12.5 mol %), K_3PO_4 (2 equiv.) in *p*-xylene (2.0 mL) at 110 °C for 12 h. [b] determined by ^{1}H NMR, using CH_2Br_2 as an internal standard. [c] isolated yields. [d] XPhos loading reduced to 5 mol%. [e] Fu's typical conditions: $Pd(OTFA)_2$ (10 mol%), *t*Bu-XPhos (10 mol%), K_2CO_3 (2 equiv.), CH_3CN (1 mL), 80 °C, N_2 atm, 16 h.

3. Preparation of some starting materials

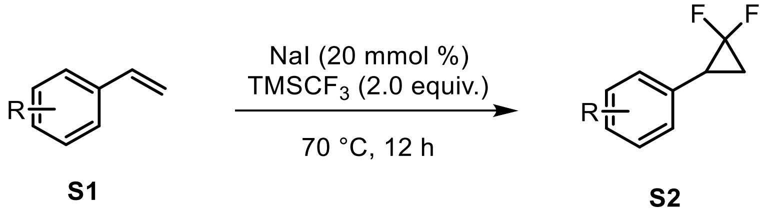

According to a known procedure,[1-2] a schlenk-tube (50 mL) was charged with **S1** (10 mmol), NaI (2 mmol, 300 mg) and $TMSCF_3$ (20 mmol). The mixture was heated up to 70 °C. the reaction was stirred (12 h) and simultaneously allowed to acclimatize to room temperature. The reaction was quenched with water (20 mL), extracted with ethyl acetate (3x 10 mL), dried over $MgSO_4$ and concentrated under reduced pressure. The crude was purified by SiO_2 gel column chromatography to afford **S2**.

4. General procedure for c

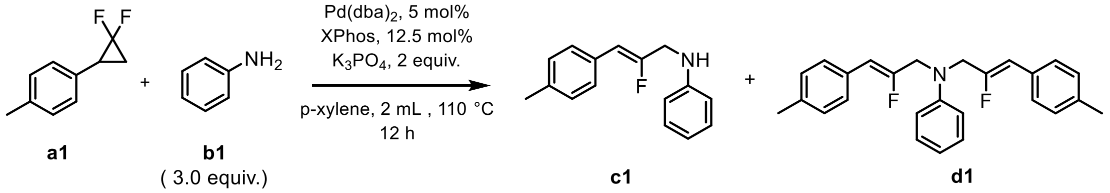

Under N_2 atmosphere, XPhos (11.9 mg, 0.025 mmol) and $Pd(dba)_2$ (5.7 mg, 0.01 mmol), gem-difluorocyclopropanes **a1** (0.2 mmol), aniline[3] **b1** (0.6 mmol), K_3PO_4 (2.0 equiv., 84.8 mg) were dissolved in 2 mL p-xylene, then the mixture was stirred at 110 °C for about 12 h to the starting material was consumed (monitored by TLC), the mixture was filtered through celite and the filtrate was concentrated to dryness. A portion of the residue was analyzed with ^1H NMR to determine selectivity and recovered. The crude was purified by column chromatography to give the products **c**.

5. Product characterization of c

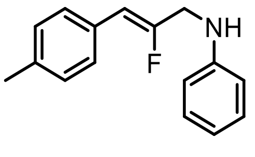

(*Z*)-*N*-(2-fluoro-3-(*p*-tolyl)allyl)aniline

c1: The crude mixture was purified by SiO_2 gel column chromatography with pentane/EA (from 60:1). 43 mg product was obtained by 89% isolated yield as yellow solid.

^{1}H NMR (600 MHz, Chloroform-d) δ 7.43 (d, *J* = 7.9 Hz, 2H), 7.25 (t, *J* = 7.9 Hz, 2H), 7.18 (d, *J* = 7.8 Hz, 2H), 6.81 (t, *J* = 7.3 Hz, 1H), 6.74 (d, *J* = 7.9 Hz, 2H), 5.77 (d, *J* = 39.6 Hz, 1H), 4.05 (s, 1H) 4.04 (d, *J* = 9.8 Hz, 2H), 2.38 (s, 3H).

^{19}F NMR (565 MHz, Chloroform-d) δ -110.50 (dt, *J* = 39.7 Hz, *J* = 9.9 Hz).

^{13}C NMR (151 MHz, Chloroform-d) δ 156.6 (d, *J* = 266.5 Hz), 147.3, 137.1 (d, *J* = 2.4 Hz), 130.1 (d, *J*

= 2.7 Hz), 129.3, 129.2, 128.5 (d, *J* = 6.9 Hz), 118.3, 113.2, 106.8 (d, *J* = 6.7 Hz), 45.6 (d, *J* = 33.4 Hz), 21.3.

IR (neat, cm^{-1}): ṽ: 3416, 2918, 1694, 1599, 1501, 1433, 1312, 1249, 1153, 1103, 989, 862, 744.

ESI-HRMS: mass spectrometry: m/z calcd for $C_{16}H_{17}NF$ $[M+H]^+$ 242.13395, measured 242.13329.

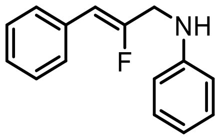

(*Z*)-*N*-(2-fluoro-3-phenylallyl)aniline

c2: The crude mixture was purified by SiO_2 gel column chromatography with pentane/EA (from 60:1). 39 mg product was obtained by 86% isolated yield as yellow oil.

^{1}H NMR (600 MHz, Chloroform-*d*) δ 7.39 (d, *J* = 6.9 Hz, 2H), 7.23 (t, *J* = 7.7 Hz, 2H), 7.16 – 7.09 (m, 3H), 6.68 (t, *J* = 7.4 Hz, 1H), 6.60 (d, *J* = 7.5 Hz, 2H), 5.67 (d, *J* = 39.5 Hz, 1H), 3.92 (s, 1H), 3.91 (d, *J* = 10.3 Hz, 2H).

^{19}F NMR (565 MHz, Chloroform-*d*) δ -109.53 (dt, *J* = 39.4 Hz, *J* = 9.7 Hz).

^{13}C NMR (151 MHz, Chloroform-*d*) δ 157.2 (d, *J* = 267.7 Hz), 147.2, 133.0 (d, *J* = 2.8 Hz), 129.4, 128.6 (d, *J* = 7.0 Hz), 128.5, 127.3 (d, *J* = 1.8 Hz), 118.3, 113.2, 106.8 (d, *J* = 6.6 Hz), 45.5 (d, *J* = 33.8 Hz).

IR (neat, cm^{-1}): ṽ: 3418, 2920, 1694, 1601, 1504, 1439, 1315, 1263, 1105, 870, 749.

ESI-HRMS: mass spectrometry: m/z calcd for $C_{15}H_{15}NF$ $[M+H]^+$ 228.11830, measured 228.11794.

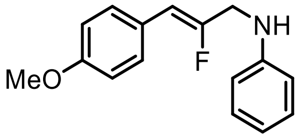

(*Z*)-*N*-(2-fluoro-3-(4-methoxyphenyl)allyl)aniline

c3: The crude mixture was purified by SiO_2 gel column chromatography with pentane/EA (from 40:1). 36 mg product was obtained by 70% isolated yield as brown solid.

^{1}H NMR (600 MHz, Chloroform-*d*) δ 7.34 (d, *J* = 8.7 Hz, 2H), 7.15 – 7.07 (m, 2H), 6.77 (d, *J* = 8.8 Hz, 2H), 6.68 (t, *J* = 7.4 Hz, 1H), 6.61 (d, *J* = 7.6 Hz, 2H), 5.62 (d, *J* = 39.7 Hz, 1H), 3.94 (broad s, 1H), 3.91 (d, *J* = 10.6 Hz, 2H), 3.72 (s, 3H).

^{19}F NMR (565 MHz, Chloroform-*d*) δ -112.41 (dt, *J* = 39.9 Hz, *J* = 10.5 Hz).

^{13}C NMR (151 MHz, Chloroform-*d*) δ 158.7 (d, *J* = 2.7 Hz), 155.8 (d, *J* = 265.1 Hz), 147.3, 129.8 (d, *J* = 7.2 Hz), 129.3, 125.7 (d, *J* = 2.8 Hz), 118.2, 113.9, 113.2, 106.4 (d, *J* = 6.8 Hz), 55.3, 45.6 (d, *J* = 33.4 Hz).

IR (neat, cm^{-1}): ṽ: 3416, 2929, 1692, 1601, 1504, 1434, 1305, 1248, 1154, 1105, 1033, 854, 742.

ESI-HRMS: mass spectrometry: m/z calcd for $C_{16}H_{15}ONF$ $[M-H]^+$ 256.11322, measured 256.11249.

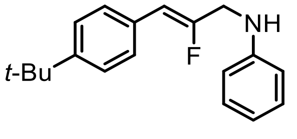

(*Z*)-*N*-(3-(4-(*tert*-butyl)phenyl)-2-fluoroallyl)aniline

c4: The crude mixture was purified by SiO_2 gel column chromatography with pentane/EA (from 60:1). 43 mg product was obtained by 76% isolated yield as yellow oil.

^{1}H NMR (600 MHz, Chloroform-*d*) δ 7.34 (d, *J* = 8.4 Hz, 2H), 7.26 (d, *J* = 8.4 Hz, 2H), 7.13 – 7.08 (m, 2H), 6.67 (t, *J* = 7.3 Hz, 1H), 6.60 (d, *J* = 7.4 Hz, 2H), 5.65 (d, *J* = 39.7 Hz, 1H), 3.95 – 3.88 (m, 3H), 1.23 (s, 9H).

^{19}F NMR (565 MHz, Chloroform-*d*) δ -110.50 (dt, *J* = 39.5 Hz, *J* = 10.0 Hz).

^{13}C NMR (151 MHz, Chloroform-*d*) δ 156.7 (d, *J* = 266.7 Hz), 150.3 (d, *J* = 2.1 Hz), 147.3, 130.2 (d, *J* = 2.4 Hz), 129.3, 128.3 (d, *J* = 7.1 Hz), 125.4, 118.3, 113.2, 106.7 (d, *J* = 6.7 Hz), 45.6 (d, *J* = 33.7 Hz), 34.6, 31.3.

IR (neat, cm^{-1}): ṽ: 3420, 2960, 1694, 1602, 1506, 1437, 1364, 1264, 1105, 986, 863, 747.

ESI-HRMS: mass spectrometry: m/z calcd for $C_{19}H_{23}NF$ $[M+H]^+$ 284.18090, measured 284.18045.

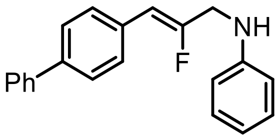

(*Z*)-*N*-(3-([1,1'-biphenyl]-4-yl)-2-fluoroallyl)aniline

c5: The crude mixture was purified by SiO_2 gel column chromatography with pentane/EA (from 60:1). 52 mg product was obtained by 86% isolated yield as brown solid.

^{1}H NMR (600 MHz, Chloroform-*d*) δ 7.53 – 7.45 (m, 6H), 7.35 (t, *J* = 7.6 Hz, 2H), 7.26 (t, *J* = 7.4 Hz, 1H), 7.13 (t, *J* = 7.9 Hz, 2H), 6.69 (t, *J* = 7.3 Hz, 1H), 6.63 (d, *J* = 8.0 Hz, 2H), 5.73 (d, *J* = 39.4 Hz, 1H), 4.0 (broad s, 1H), 3.95 (d, *J* = 9.7 Hz, 2H).

^{19}F NMR (565 MHz, Chloroform-*d*) δ -109.04 (dt, *J* = 39.6 Hz, *J* = 9.6 Hz).

^{13}C NMR (151 MHz, Chloroform-*d*) δ 157.4 (d, *J* = 268.1 Hz), 147.2, 140.7, 139.9 (d, *J* = 2.5 Hz), 132.1 (d, *J* = 2.4 Hz), 129.4, 129.0 (d, *J* = 7.2 Hz), 128.8, 127.3, 127.1, 127.0, 118.4, 113.2, 106.5 (d, *J* = 6.6 Hz), 45.6 (d, *J* = 33.7 Hz).

IR (neat, cm^{-1}): ṽ: 3399, 2920, 1596, 1503, 1311, 1157, 1067, 982, 908, 867, 751, 688.

ESI-HRMS: mass spectrometry: m/z calcd for $C_{21}H_{18}NFNa$ $[M+Na]^+$ 326.13155, measured 326.13123.

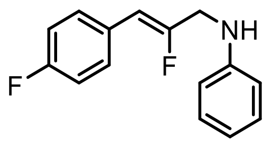

(*Z*)-*N*-(2-fluoro-3-(4-fluorophenyl)allyl)aniline

c6: The crude mixture was purified by SiO_2 gel column chromatography with pentane/EA (from 60:1). 45 mg product was obtained by 92% isolated yield as yellow oil.

^{1}H NMR (600 MHz, Chloroform-*d*) δ 7.36 (dd, *J* = 8.4, 5.5 Hz, 2H), 7.12 (t, *J* = 7.6 Hz, 2H), 6.91 (t, *J* = 8.5 Hz, 2H), 6.68 (t, *J* = 7.3 Hz, 1H), 6.60 (d, *J* = 7.9 Hz, 2H), 5.64 (d, *J* = 39.1 Hz, 1H), 3.94 (broad s, 1H), 3.91 (d, *J* = 9.7 Hz, 2H).

^{19}F NMR (565 MHz, Chloroform-*d*) δ -110.74 (dt, *J* = 39.1, 9.8 Hz, 1F), -114.18 (m, 1F).

^{13}C NMR (151 MHz, Chloroform-*d*) δ 161.8 (dd, *J* = 247.1, 3.2 Hz), 156.9 (dd, *J* = 267.5, 2.3 Hz), 147.1, 130.2 (t, *J* = 7.6 Hz), 129.4, 129.1 (t, *J* = 3.0 Hz), 118.4, 115.4 (d, *J* = 21.4 Hz), 113.2, 105.7 (d, *J* = 6.7 Hz), 45.4 (d, *J* = 33.8 Hz).

IR (neat, cm^{-1}): ṽ: 3418, 2920, 1695, 1601, 1504, 1436, 1378, 1228, 1157, 1106, 985, 749.

APCI-HRMS: mass spectrometry: m/z calcd for $C_{15}H_{14}NF_2$ $[M+H]^+$ 246.10888, measured 246.10889.

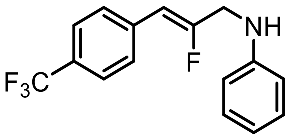

(*Z*)-*N*-(2-fluoro-3-(4-(trifluoromethyl)phenyl)allyl)aniline

c7: The crude mixture was purified by SiO_2 gel column chromatography with pentane/EA (from 40:1). 45 mg product was obtained by 76% isolated yield as brown oil.

^{1}H NMR (600 MHz, Chloroform-*d*) δ 7.47 (s, 4H), 7.15 – 7.11 (m, 2H), 6.70 (t, *J* = 7.4 Hz, 1H), 6.61 (d, *J* = 7.4 Hz, 2H), 5.73 (d, *J* = 38.7 Hz, 1H), 3.97 (broad s, 1H), 3.95 (d, *J* = 8.5 Hz, 2H).

^{19}F NMR (564 MHz, Chloroform-*d*) δ -62.60 (s, 3F), -106.34 (dt, *J* = 38.8, 8.5 Hz, 1F).

^{13}C NMR (151 MHz, Chloroform-*d*) δ 159.1 (d, *J* = 271.2 Hz), 147.0, 136.5, 129.4, 128.95 (qd, *J* = 32.6 Hz, *J* = 2.5 Hz), 128.7 (d, *J* = 7.4 Hz), 125.4 (q, *J* = 3.8 Hz), 124.1 (q, *J* = 271.8 Hz), 118.5, 113.1, 105.5 (d, *J* = 6.0 Hz), 45.3 (d, *J* = 34.1 Hz).

IR (neat, cm^{-1}): ṽ: 3418, 2922, 1694, 1603, 1506, 1415, 1322, 1263, 1163, 1066, 1017, 865, 750.

EI-HRMS: mass spectrometry: m/z calcd for $C_{16}H_{13}NF_4$ $[M]^+$ 295.09786, measured 295.09788.

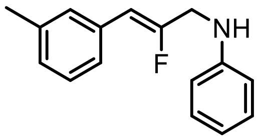

(*Z*)-*N*-(2-fluoro-3-(*m*-tolyl)allyl)aniline

c8: The crude mixture was purified by SiO_2 gel column chromatography with pentane/EA (from 60:1). 40 mg product was obtained by 83% isolated yield as brown oil.

^{1}H NMR (600 MHz, Chloroform-*d*) δ 7.21 (s, 2H), 7.15 – 7.09 (m, 3H), 6.96 (d, *J* = 7.5 Hz, 1H), 6.68 (t, *J* = 7.3 Hz, 1H), 6.60 (d, *J* = 7.5 Hz, 2H), 5.64 (d, *J* = 39.6 Hz, 1H), 3.94 (broad s, 1H), 3.91 (d, *J* = 9.9 Hz, 2H), 2.25 (s, 3H).

^{19}F NMR (565 MHz, Chloroform-*d*) δ -109.63 (dt, *J* = 39.9 Hz, *J* = 9.9 Hz).

^{13}C NMR (151 MHz, Chloroform-*d*) δ 157.0 (d, *J* = 267.4 Hz), 147.2, 138.0, 132.9 (d, *J* = 2.8 Hz), 129.3, 129.3 (d, *J* = 6.7 Hz), 128.4, 128.0 (d, *J* = 2.3 Hz), 125.7 (d, *J* = 7.3 Hz), 118.3, 113.2, 106.9 (d, *J* = 6.3 Hz), 45.5 (d, *J* = 33.6 Hz), 21.4.

IR (neat, cm^{-1}): ṽ: 3446, 2922, 1693, 1603, 1504, 1436, 1277, 1243, 1102, 984, 847, 740, 688.

APCI-HRMS: mass spectrometry: m/z calcd for $C_{16}H_{17}NF$ $[M+H]^+$ 242.13395, measured 242.13414.

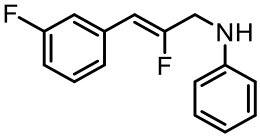

(*Z*)-*N*-(2-fluoro-3-(3-fluorophenyl)allyl)aniline

c9: The crude mixture was purified by SiO_2 gel column chromatography with pentane/EA (from 60:1). 46 mg product was obtained by 94% isolated yield as yellow oil.

^{1}H NMR (600 MHz, Chloroform-*d*) δ 7.20 – 7.08 (m, 5H), 6.84 (tdd, *J* = 8.5 Hz, *J* = 2.7 Hz, *J* = 1.0 Hz, 1H), 6.69 (tt, *J* = 7.3 Hz, *J* = 1.0 Hz, 1H), 6.60 (dm, *J* = 7.4 Hz, 2H), 5.67 (d, *J* = 38.6 Hz, 1H), 3.95 (broad s, 1H) 3.93 (d, *J* = 9.3 Hz, 2H).

^{19}F NMR (565 MHz, Chloroform-*d*) δ -107.49 (dt, *J* = 38.8 Hz, *J* = 8.9 Hz, 1F), -113.29 (m, 1F).

^{13}C NMR (151 MHz, Chloroform-*d*) δ 162.8 (d, *J* = 244.6 Hz), 158.2 (d, *J* = 269.7 Hz), 147.0, 135.0 (dd, *J* = 8.5, 2.4 Hz), 129.8 (d, *J* = 8.4 Hz), 129.4, 124.3 (dd, *J* = 6.4, 2.8 Hz), 118.4, 115.2 (dd, *J* = 22.7, 8.6 Hz), 114.1 (dd, *J* = 23.3 Hz, *J* = 2.2 Hz), 113.1, 105.8 (dd, *J* = 6.2, 2.7 Hz), 45.4 (d, *J* = 33.9 Hz).

IR (neat, cm^{-1}): ṽ: 3418, 2921, 2326, 1692, 1602, 1505, 1438, 1310, 1246, 1152, 963, 875, 750.

APCI-HRMS: mass spectrometry: m/z calcd for $C_{15}H_{14}NF_2$ $[M+H]^+$ 246.10888, measured 246.10929.

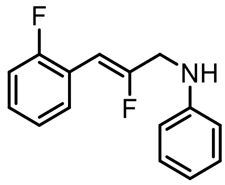

(*Z*)-*N*-(2-fluoro-3-(2-fluorophenyl)allyl)aniline

c10: The crude mixture was purified by SiO_2 gel column chromatography with pentane/EA (from 60:1). 30 mg product was obtained by 61% isolated yield as yellow oil.

^{1}H NMR (600 MHz, Chloroform-*d*) δ 7.70 (td, *J* = 7.7 Hz, *J* = 1.8 Hz, 1H), 7.16 – 7.09 (m, 3H), 7.03 (t, *J* = 7.7 Hz, 1H), 6.94 (m, 1H), 6.69 (t, *J* = 7.3 Hz, 1H), 6.62 (d, *J* = 7.5 Hz, 2H), 5.96 (d, *J* = 39.1 Hz, 1H), 3.96 (d, *J* = 10.4 Hz, 2H), 3.95 (broad s, 1H).

^{19}F NMR (565 MHz, Chloroform-*d*) δ -108.03 (dtd, *J* = 38.6 Hz, *J* = 10.7 Hz, *J* = 4.6 Hz, 1F), -116.85 (m, 1F).

^{13}C NMR (151 MHz, Chloroform-*d*) δ 159.4 (d, *J* = 249.3 Hz), 158.5 (dd, *J* = 269.6, 2.2 Hz), 147.1, 130.1 (dd, *J* = 12.2, 2.9 Hz), 129.4, 128.7 (dd, *J* = 8.5 Hz, *J* = 1.5 Hz), 124.1 (d, *J* = 3.6 Hz), 120.8 (dd, *J* = 12.1, 2.6 Hz), 118.4, 115.2 (d, *J* = 22.2 Hz), 113.2, 98.6 (t, *J* = 6.6 Hz), 45.6 (d, *J* = 32.8 Hz).

IR (neat, cm^{-1}): ṽ: 3418, 2921, 1695, 1602, 1503, 1452, 1313, 1253, 1152, 1112, 985, 824, 750.

ESI-HRMS: mass spectrometry: m/z calcd for $C_{15}H_{14}NF_2$ $[M+H]^+$ 246.10888, measured 246.10830.

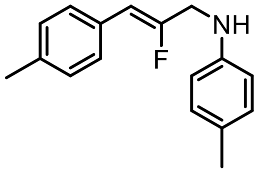

(*Z*)-*N*-(2-fluoro-3-(*p*-tolyl)allyl)-4-methylaniline

c11: The crude mixture was purified by SiO_2 gel column chromatography with pentane/EA (from 60:1). 50 mg product was obtained by 98% isolated yield as brown oil.

^{1}H NMR (600 MHz, Chloroform-*d*) δ 7.28 (d, *J* = 8.0 Hz, 2H), 7.03 (d, *J* = 7.9 Hz, 2H), 6.92 (d, *J* = 8.1 Hz, 2H), 6.52 (d, *J* = 8.4 Hz, 2H), 5.62 (d, *J* = 39.7 Hz, 1H), 3.87 (d, *J* = 10.3 Hz, 2H), 3.84 – 3.69 (broad s, 1H), 2.24 (s, 3H), 2.16 (s, 3H).

^{19}F NMR (565 MHz, Chloroform-*d*) δ -110.40 (dt, *J* = 39.8 Hz, *J* = 10.3 Hz).

^{13}C NMR (151 MHz, Chloroform-*d*) δ 156.8 (d, *J* = 266.8 Hz), 145.0, 137.0 (d, *J* = 2.3 Hz), 130.2 (d, *J* = 2.4 Hz), 129.8, 129.2, 128.5 (d, *J* = 7.2 Hz), 127.5, 113.4, 106.7 (d, *J* = 6.7 Hz), 45.9 (d, *J* = 33.3 Hz), 21.3, 20.4.

IR (neat, cm^{-1}): ṽ: 3400, 2913, 1691, 1613, 1515, 1449, 1340, 1245, 1156, 1091, 987, 878, 808.

APCI-HRMS: mass spectrometry: m/z calcd for $C_{17}H_{19}NF$ $[M+H]^+$ 256.14960, measured 256.14968.

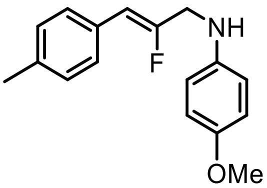

(*Z*)-*N*-(2-fluoro-3-(*p*-tolyl)allyl)-4-methoxyaniline

c12: The crude mixture was purified by SiO_2 gel column chromatography with pentane/EA (from 40:1). 49 mg product was obtained by 90% isolated yield as black solid.

^{1}H NMR (600 MHz, Chloroform-*d*) δ 7.29 (d, *J* = 8.1 Hz, 2H), 7.04 (d, *J* = 7.9 Hz, 2H), 6.74 – 6.68 (2nd order m, 2H), 6.60 – 6.53 (2nd order m, 2H), 5.62 (d, *J* = 39.8 Hz, 1H), 3.85 (d, *J* = 10.8 Hz, 2H), 3.71 (broad s, 1H), 3.66 (s, 3H), 2.24 (s, 3H).

^{19}F NMR (565 MHz, Chloroform-*d*) δ -110.40 (dt, *J* = 39.6 Hz, *J* = 10.7 Hz).

^{13}C NMR (151 MHz, Chloroform-*d*) δ 156.9 (d, *J* = 266.4 Hz), 152.7, 141.4, 137.0 (d, *J* = 2.6 Hz), 130.2 (d, *J* = 2.4 Hz), 129.2, 128.5 (d, *J* = 7.0 Hz), 114.9, 114.7, 106.8 (d, *J* = 6.8 Hz), 55.8, 46.6 (d, *J* = 33.0 Hz), 21.2.

IR (neat, cm^{-1}): ṽ: 3398, 2919, 1691, 1613, 1507, 1462, 1343, 1233, 1154, 1035, 873, 816, 761.

EI-HRMS: mass spectrometry: m/z calcd for $C_{17}H_{18}ONF$ $[M]^+$ 271.13669, measured 271.13661.

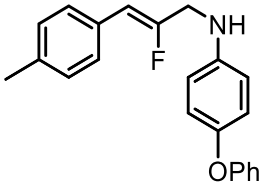

(*Z*)-*N*-(2-fluoro-3-(*p*-tolyl)allyl)-4-phenoxyaniline

c13: The crude mixture was purified by SiO_2 gel column chromatography with pentane/EA (from 40:1). 58 mg product was obtained by 87% isolated yield as yellow oil.

^{1}H NMR (600 MHz, Chloroform-*d*) δ 7.43 (d, *J* = 7.8 Hz, 2H), 7.32 (t, *J* = 7.7 Hz, 2H), 7.18 (d, *J* = 7.8 Hz, 2H), 7.05 (t, *J* = 7.3 Hz, 1H), 6.97 (t, *J* = 8.8 Hz, 4H), 6.72 (d, *J* = 8.6 Hz, 2H), 5.77 (d, *J* = 39.6 Hz, 1H), 4.02 (d, *J* = 11.0 Hz, 2H), 4.00 (broad s, 1H), 2.38 (s, 3H).

^{19}F NMR (565 MHz, Chloroform-*d*) δ -110.54 (dt, *J* = 39.4 Hz, *J* = 10.8 Hz).

^{13}C NMR (151 MHz, Chloroform-*d*) δ 158.9, 156.5 (d, *J* = 266.5 Hz), 148.4, 143.8, 137.1 (d, *J* = 2.3 Hz), 130.1 (d, *J* = 2.4 Hz), 129.5, 129.2, 128.5 (d, *J* = 7.0 Hz), 122.1, 121.2, 117.3, 114.3, 107.0 (d, *J* = 6.8 Hz), 46.1 (d, *J* = 33.1 Hz), 21.3.

IR (neat, cm^{-1}): ṽ: 3424, 2915, 1691, 1589, 1508, 1407, 1333, 1229, 1151, 1074, 989, 869, 747.

ESI-HRMS: mass spectrometry: m/z calcd for $C_{22}H_{20}ONFK$ $[M+K]^+$ 372.11605, measured 372.11599.

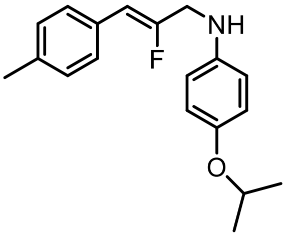

(*Z*)-*N*-(2-fluoro-3-(*p*-tolyl)allyl)-4-isopropoxyaniline

c14: The crude mixture was purified by SiO_2 gel column chromatography with pentane/EA (from 40:1). 58 mg product was obtained by 97% isolated yield as brown oil.

^{1}H NMR (600 MHz, Chloroform-*d*) δ 7.29 (d, *J* = 8.1 Hz, 2H), 7.04 (d, *J* = 7.9 Hz, 2H), 6.70 (d, *J* = 8.9 Hz, 2H), 6.54 (d, *J* = 8.8 Hz, 2H), 5.63 (d, *J* = 39.7 Hz, 1H), 4.28 (sept, *J* = 6.1 Hz, 1H), 3.85 (d, *J* = 10.6 Hz, 2H), 3.69 (broad s, 1H), 2.24 (s, 3H), 1.20 (d, *J* = 6.1 Hz, 6H).

^{19}F NMR (565 MHz, Chloroform-*d*) δ -110.33 (dt, *J* = 39.6 Hz, *J* = 10.7 Hz).

^{13}C NMR (151 MHz, Chloroform-*d*) δ 156.9 (d, *J* = 266.6 Hz), 150.7, 141.6, 137.0 (d, *J* = 2.3 Hz), 130.2 (d, *J* = 2.5 Hz), 129.2, 128.5 (d, *J* = 7.0 Hz), 117.9, 114.5, 106.8 (d, *J* = 6.8 Hz), 71.1, 46.5 (d, *J* = 33.0 Hz), 22.2, 21.2.

IR (neat, cm^{-1}): ṽ: 3418, 2978, 1693, 1507, 1454, 1374, 1337, 1227, 1116, 946, 860, 814.

EI-HRMS: mass spectrometry: m/z calcd for $C_{19}H_{22}ONF$ $[M]^+$ 299.16799, measured 299.16793.

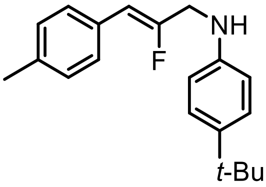

(*Z*)-4-(*tert*-butyl)-*N*-(2-fluoro-3-(*p*-tolyl)allyl)aniline

c15: The crude mixture was purified by SiO_2 gel column chromatography with pentane/EA (from 60:1). 58 mg product was obtained by 98% isolated yield as yellow oil.

^{1}H NMR (600 MHz, Chloroform-*d*) δ 7.29 (d, *J* = 8.0 Hz, 2H), 7.14 (d, *J* = 8.6 Hz, 2H), 7.04 (d, *J* = 7.9 Hz, 2H), 6.56 (d, *J* = 8.6 Hz, 2H), 5.65 (d, *J* = 39.7 Hz, 1H), 3.88 (d, *J* = 10.3 Hz, 2H), 3.83 (broad s, 1H), 2.24 (s, 3H), 1.20 (s, 9H).

^{19}F NMR (565 MHz, Chloroform-*d*) δ -110.30 (dt, *J* = 39.9 Hz, *J* = 10.1 Hz).

^{13}C NMR (151 MHz, Chloroform-*d*) δ 156.9 (d, *J* = 266.4 Hz), 144.9, 141.0, 137.0 (d, *J* = 2.3 Hz), 130.2 (d, *J* = 2.5 Hz), 129.2, 128.5 (d, *J* = 6.9 Hz), 126.1, 112.9, 106.6 (d, *J* = 6.7 Hz), 45.8 (d, *J* = 33.4 Hz), 33.9, 31. 6, 21.3.

IR (neat, cm^{-1}): ṽ: 3383, 2956, 1695, 1614, 1516, 1455, 1360, 1300, 1191, 1158, 1079, 992, 816.

ESI-HRMS: mass spectrometry: m/z calcd for $C_{20}H_{24}NFNa$ $[M+Na]^+$ 320.17850, measured 320.17773.

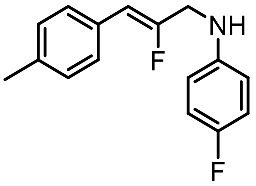

(*Z*)-4-fluoro-*N*-(2-fluoro-3-(*p*-tolyl)allyl)aniline

c16: The crude mixture was purified by SiO_2 gel column chromatography with pentane/EA (from

60:1). 43 mg product was obtained by 83% isolated yield as brown solid.

^{1}H NMR (600 MHz, Chloroform-*d*) δ 7.28 (d, *J* = 8.0 Hz, 2H), 7.05 (d, *J* = 7.9 Hz, 2H), 6.85 – 6.79 (m, 2H), 6.56 – 6.50 (m, 2H), 5.62 (d, *J* = 39.6 Hz, 1H), 3.86 (d, *J* = 10.7 Hz, 2H), 3.82 (broad s, 1H), 2.25 (s, 3H).

^{19}F NMR (564 MHz, Chloroform-*d*) δ -110.72 (dt, *J* = 39.7, 10.7 Hz, 1F), -127.06 (m, 1F).

^{13}C NMR (151 MHz, Chloroform-*d*) δ 156.4 (d, *J* = 266.5 Hz), 156.3 (d, *J* = 236.0 Hz), 143.5 (d, *J* = 1.9 Hz), 137.2 (d, *J* = 2.3 Hz), 130.0 (d, *J* = 2.5 Hz), 129.2, 128.5 (d, *J* = 7.2 Hz), 115.8 (d, *J* = 22.3 Hz), 114.2 (d, *J* = 7.4 Hz), 107.0 (d, *J* = 6.7 Hz), 46.2 (d, *J* = 32.9 Hz), 21.2.

IR (neat, cm^{-1}): ṽ: 3391, 2920, 1691, 1610, 1510, 1343, 1221, 1155, 1111, 989, 874, 785.

EI-HRMS: mass spectrometry: m/z calcd for $C_{16}H_{15}NF_2$ [M]$^+$ 259.11671, measured 59.11663.

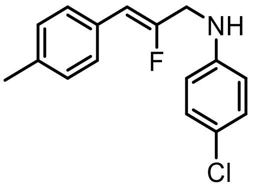

(*Z*)-4-chloro-*N*-(2-fluoro-3-(*p*-tolyl)allyl)aniline

c17: The crude mixture was purified by SiO_2 gel column chromatography with pentane/EA (from 60:1). 27 mg product was obtained by 49% isolated yield as brown solid.

^{1}H NMR (600 MHz, Chloroform-*d*) δ 7.28 (d, *J* = 7.9 Hz, 2H), 7.11 – 7.02 (m, 4H), 6.52 (d, *J* = 8.8 Hz, 2H), 5.61 (d, *J* = 39.5 Hz, 1H), 3.98 (broad s, 1H), 3.88 (d, *J* = 10.5 Hz, 2H), 2.25 (s, 3H).

^{19}F NMR (565 MHz, Chloroform-*d*) δ -110.84 (dt, *J* = 39.8 Hz, *J* = 10.4 Hz).

^{13}C NMR (151 MHz, Chloroform-*d*) δ 156.0 (d, *J* = 266.5 Hz), 145.8, 137.2 (d, *J* = 2.3 Hz), 129.9 (d, *J* = 2.5 Hz), 129.2, 129.1, 128.5 (d, *J* = 7.0 Hz), 122.9, 114.3, 107.1 (d, *J* = 6.8 Hz), 45.6 (d, *J* = 33.4 Hz), 21.2.

IR (neat, cm^{-1}): ṽ: 3390, 2917, 1691, 1599, 1495, 1340, 1239, 1156, 1083, 993, 874, 814, 711.

EI-HRMS: mass spectrometry: m/z calcd for $C_{16}H_{15}NClF$ [M]$^+$ 275.08716, measured 275.08730.

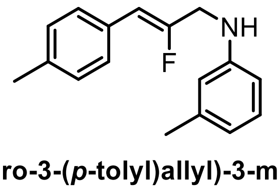

(*Z*)-*N*-(2-fluoro-3-(*p*-tolyl)allyl)-3-methylaniline

c18: The crude mixture was purified by SiO_2 gel column chromatography with pentane/EA (from 60:1). 48 mg product was obtained by 94% isolated yield as brown solid.

^{1}H NMR (600 MHz, Chloroform-*d*) δ 7.29 (d, *J* = 7.9 Hz, 2H), 7.04 (d, *J* = 7.9 Hz, 2H), 7.00 (t, *J* = 8.0 Hz, 1H), 6.50 (d, *J* = 7.5 Hz, 1H), 6.41 (d, *J* = 6.5 Hz, 2H), 5.63 (d, *J* = 39.8 Hz, 1H), 3.88 (d, *J* = 10.1 Hz, 2H), 3.86 (broad s, 1H), 2.24 (s, 3H), 2.20 (s, 3H).

^{19}F NMR (565 MHz, Chloroform-*d*) δ -110.39 (dt, *J* = 39.5 Hz, *J* = 10.1 Hz).

^{13}C NMR (151 MHz, Chloroform-*d*) δ 156.8 (d, *J* = 266.8 Hz), 147.3, 139.2, 137.0 (d, *J* = 2.3 Hz), 130.2 (d, *J* = 2.5 Hz), 129.22, 129.18, 128.5 (d, *J* = 7.0 Hz), 119.2, 114.0, 110.3, 106.7 (d, *J* = 6.7 Hz), 45.6 (d, *J* = 33.5 Hz), 21.7, 21.3.

IR (neat, cm^{-1}): ṽ: 3415, 2919, 1694, 1603, 1510, 1443, 1325, 1269, 1178, 1104, 988, 857, 769.

ESI-HRMS: mass spectrometry: m/z calcd for $C_{17}H_{18}NFNa$ [M+Na]$^+$ 278.13155, measured 278.13071.

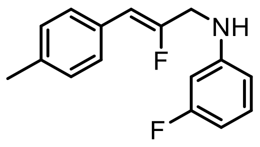

(*Z*)-3-fluoro-*N*-(2-fluoro-3-(*p*-tolyl)allyl)aniline

c19: The crude mixture was purified by SiO_2 gel column chromatography with pentane/EA (from 60:1). 48 mg product was obtained by 93% isolated yield as yellow oil.

^{1}H NMR (600 MHz, Chloroform-*d*) δ 7.29 (d, *J* = 7.9 Hz, 2H), 7.07 – 6.99 (m, 3H), 6.37 – 6.32 (m, 2H), 6.28 (d, *J* = 11.4 Hz, 1H), 5.62 (d, *J* = 39.5 Hz, 1H), 4.04 (very broad t, *J* = 5.5 Hz, 1H), 3.88 (dd, *J* = 10.7, 5.3 Hz, 2H), 2.25 (s, 3H).

^{19}F NMR (565 MHz, Chloroform-*d*) δ -110.84 (dt, *J* = 39.4 Hz, *J* = 10.8 Hz, 1F), -112.57 (m, 1F).

^{13}C NMR (151 MHz, Chloroform-*d*) δ 164.1 (d, *J* = 243.3 Hz), 155.9 (d, *J* = 266.5 Hz), 149.0 (d, *J* = 10.5 Hz), 137.2 (d, *J* = 2.4 Hz), 130.4 (d, *J* = 10.2 Hz), 129.9 (d, *J* = 2.7 Hz), 129.2, 128.5 (d, *J* = 7.1 Hz), 109.0 (d, *J* = 2.4 Hz), 107.1 (d, *J* = 6.8 Hz), 104.7 (d, *J* = 21.7 Hz), 100.0 (d, *J* = 25.5 Hz), 45.4 (d, *J* = 33.4 Hz), 21.2.

IR (neat, cm^{-1}): ṽ: 3429, 2926, 1694, 1592, 1506, 1438, 1336, 1285, 1144, 1102, 967, 863, 757.

EI-HRMS: mass spectrometry: m/z calcd for $C_{16}H_{15}NF_2$ $[M]^+$ 259.11671, measured 259.11645.

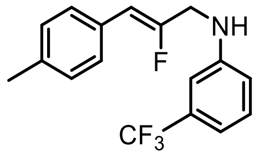

(Z)-N-(2-fluoro-3-(p-tolyl)allyl)-3-(trifluoromethyl)aniline

c20: The crude mixture was purified by SiO_2 gel column chromatography with pentane/EA (from 40:1). 55 mg product was obtained by 89% isolated yield as yellow oil.

^{1}H NMR (600 MHz, Chloroform-*d*) δ 7.29 (d, *J* = 7.9 Hz, 2H), 7.19 (t, *J* = 8.0 Hz, 1H), 7.05 (d, *J* = 7.8 Hz, 2H), 6.90 (d, *J* = 7.7 Hz, 1H), 6.79 (s, 1H), 6.72 (d, *J* = 8.3 Hz, 1H), 5.63 (d, *J* = 39.3 Hz, 1H), 4.11 (broad t, *J* = 5.2 Hz, 1H), 3.92 (dd, *J* = 11.1, 6.0 Hz, 2H), 2.24 (s, 3H).

^{19}F NMR (564 MHz, Chloroform-*d*) δ -62.87 (s, 3F), -111.06 (dt, *J* = 39.4 Hz, *J* = 11.0 Hz, 1F).

^{13}C NMR (151 MHz, Chloroform-*d*) δ 155.7 (d, *J* = 266.4 Hz), 147.4, 137.3 (d, *J* = 2.5 Hz), 131.7 (q, *J* = 31.8 Hz), 129.8 (d, *J* = 2.8 Hz), 129.8, 129.2, 128.5 (d, *J* = 6.9 Hz), 124.3 (q, *J* = 272.4 Hz), 116.0, 114.7 (q, *J* = 3.8 Hz), 109.5 (q, *J* = 4.1 Hz), 107.4 (d, *J* = 6.7 Hz), 45.3 (d, *J* = 33.2 Hz), 21.2.

IR (neat, cm^{-1}): ṽ: 3425, 2924, 1694, 1614, 1513, 1444, 1335, 1256, 1160, 1068, 993, 861, 696.

EI-HRMS: mass spectrometry: m/z calcd for $C_{17}H_{15}NF_4$ $[M]^+$ 309.11351, measured 309.11331.

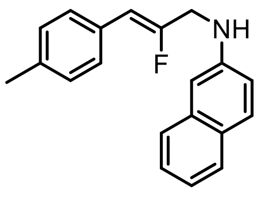

(*Z*)-*N*-(2-fluoro-3-(*p*-tolyl)allyl)naphthalen-2-amine

c21: The crude mixture was purified by SiO_2 gel column chromatography with pentane/EA (from 40:1). 47 mg product was obtained by 81% isolated yield as black solid.

^{1}H NMR (600 MHz, Chloroform-*d*) δ 7.59 (d, *J* = 8.2 Hz, 1H), 7.57 (d, *J* = 8.8 Hz, 1H), 7.53 (d, *J* = 8.3 Hz, 1H), 7.32 – 7.24 (m, 3H), 7.14 (d, *J* = 6.6 Hz, 1H), 7.03 (d, *J* = 7.9 Hz, 2H), 6.84 (dd, *J* = 8.8 Hz, *J* = 2.4 Hz, 1H), 6.80 (d, *J* = 2.3 Hz, 1H), 5.67 (d, *J* = 39.6 Hz, 1H), 4.07 (broad s, 1H), 3.99 (d, *J* = 10.3 Hz, 2H), 2.23 (s, 3H).
^{19}F NMR (565 MHz, Chloroform-*d*) δ -110.43 (dt, *J* = 39.9 Hz, *J* = 10.8 Hz).
^{13}C NMR (151 MHz, Chloroform-*d*) δ 156.3 (d, *J* = 266.5 Hz), 144.9, 137.1 (d, *J* = 2.3 Hz), 135.0, 130.1 (d, *J* = 2.6 Hz), 129.2, 129.1, 128.5 (d, *J* = 7.1 Hz), 127.9, 127.7, 126.5, 126.1, 122.4, 117.7, 107.0 (d, *J* = 6.7 Hz), 105.4, 45.6 (d, *J* = 33.4 Hz), 21.3.
IR (neat, cm^{-1}): ṽ: 3400, 2916, 1685, 1625, 1503, 1428, 1306, 1220, 1148, 953, 806, 705.
ESI-HRMS: mass spectrometry: m/z calcd for $C_{20}H_{19}NF$ $[M+H]^+$ 292.14960, measured 292.14928.

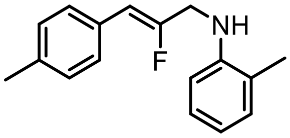

(*Z*)-*N*-(2-fluoro-3-(*p*-tolyl)allyl)-2-methylaniline

c22: The crude mixture was purified by SiO_2 gel column chromatography with pentane/EA (from 60:1). 48 mg product was obtained by 94% isolated yield as yellow oil.
^{1}H NMR (600 MHz, Chloroform-*d*) δ 7.43 (d, *J* = 8.0 Hz, 2H), 7.18 (d+m, *J* = 8.1 Hz, 3H), 7.14 (d, *J* = 7.3 Hz, 1H), 6.76 (t, *J* = 7. 4 Hz, 1H), 6.73 (d, *J* = 8.0 Hz, 1H), 5.78 (d, *J* = 39.6 Hz, 1H), 4.09 (d, *J* = 10.4 Hz, 2H), 3.94 (broad s, 1H), 2.38 (s, 3H), 2.25 (s, 3H).
^{19}F NMR (565 MHz, Chloroform-*d*) δ -110.49 (dt, *J* = 39.9 Hz, *J* = 10.2 Hz).
^{13}C NMR (151 MHz, Chloroform-*d*) δ 156.6 (d, *J* = 266.3 Hz), 145.2, 137.1 (d, *J* = 2.4 Hz), 130.3, 130.1 (d, *J* = 2.5 Hz), 129.2, 128.5 (d, *J* = 6.9 Hz), 127.2, 122.3, 117.9, 110.3, 106.8 (d, *J* = 6.7 Hz), 45.6 (d, *J* = 33.3 Hz), 21.3, 17.5.
IR (neat, cm^{-1}): ṽ: 3440, 2919, 1694, 1604, 1510, 1448, 1312, 1262, 1125, 1051, 983, 860, 746.
ESI-HRMS: mass spectrometry: m/z calcd for $C_{17}H_{19}NF$ $[M+H]^+$ 256.14960, measured 256.14956.

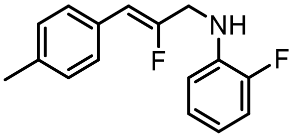

(*Z*)-2-fluoro-*N*-(2-fluoro-3-(*p*-tolyl)allyl)aniline

c23: The crude mixture was purified by SiO_2 gel column chromatography with pentane/EA (from 60:1). 42 mg product was obtained by 81% isolated yield as yellow oil.
^{1}H NMR (600 MHz, Chloroform-*d*) δ 7.29 (d, *J* = 7.9 Hz, 2H), 7.05 (d, *J* = 7.8 Hz, 2H), 6.95 – 6.88 (m, 2H), 6.69 (t, *J* = 8.3 Hz, 1H), 6.62 – 6.56 (m, 1H), 5.64 (d, *J* = 39.5 Hz, 1H), 4.22 (broad s, 1H), 3.94 (d, *J* = 10.2 Hz, 2H), 2.25 (s, 3H).
^{19}F NMR (564 MHz, Chloroform-*d*) δ -110.76 (dt, *J* = 39.4 Hz, *J* = 10.3 Hz, 1F), -136.27 (m, 1F).
^{13}C NMR (151 MHz, Chloroform-*d*) δ 156.1 (d, *J* = 266.4 Hz), 151.7 (d, *J* = 238.6 Hz), 137.2 (d, *J* = 2.4 Hz), 135.7 (d, *J* = 11.5 Hz), 130.0 (d, *J* = 2.8 Hz), 129.2, 128.5 (d, *J* = 7.2 Hz), 124.6 (d, *J* = 3.6 Hz), 117.6 (d, *J* = 7.1 Hz), 114.6 (d, *J* = 18.6 Hz), 112.6 (d, *J* = 3.0 Hz), 106.9 (d, *J* = 6.7 Hz), 45.1 (d, *J* = 34.0 Hz), 21.2.
IR (neat, cm^{-1}): ṽ: 3434, 2921, 1694, 1620, 1515, 1450, 1336, 1256, 1189, 1112, 1036, 860, 740.

EI-HRMS: mass spectrometry: m/z calcd for $C_{16}H_{15}NF_2$ $[M]^+$ 259.11671, measured 259.11664.

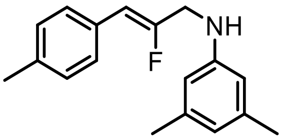

(*Z*)-*N*-(2-fluoro-3-(*p*-tolyl)allyl)-3,5-dimethylaniline

c24: The crude mixture was purified by SiO_2 gel column chromatography with pentane/EA (from 60:1). 50 mg product was obtained by 93% isolated yield as yellow oil.

^{1}H NMR (600 MHz, Chloroform-*d*) δ 7.29 (d, *J* = 7.9 Hz, 2H), 7.04 (d, *J* = 7.9 Hz, 2H), 6.34 (s, 1H), 6.24 (s, 2H), 5.64 (d, *J* = 39.8 Hz, 1H), 3.88 (d, *J* = 9.9 Hz, 2H), 3.81 (broad s, 1H), 2.25 (s, 3H), 2.16 (s, 6H).

^{19}F NMR (565 MHz, Chloroform-*d*) δ -110.30 (dt, *J* = 40.0 Hz, *J* = 9.9 Hz).

^{13}C NMR (151 MHz, Chloroform-*d*) δ 156.9 (d, *J* = 266.8 Hz), 147.4, 139.0, 137.0 (d, *J* = 2.3 Hz), 130.2 (d, *J* = 2.7 Hz), 129.2, 128.5 (d, *J* = 6.9 Hz), 120.3, 111.1, 106.5 (d, *J* = 6.9 Hz), 45.6 (d, *J* = 33.8 Hz), 21.5, 21.2.

IR (neat, cm^{-1}): ṽ: 3412, 2918, 1694, 1601, 1512, 1474, 1334, 1189, 1034, 858, 820, 689.

ESI-HRMS: mass spectrometry: m/z calcd for $C_{18}H_{20}NFK$ $[M+K]^+$ 308.12114, measured 308.12103.

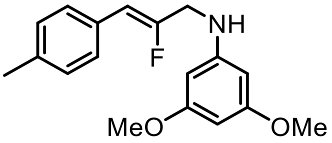

(*Z*)-*N*-(2-fluoro-3-(*p*-tolyl)allyl)-3,5-dimethoxyaniline

c25: The crude mixture was purified by SiO_2 gel column chromatography with pentane/EA (from 40:1). 45 mg product was obtained by 75% isolated yield as yellow oil.

^{1}H NMR (600 MHz, Chloroform-*d*) δ 7.41 (d, *J* = 7.8 Hz, 2H), 7.16 (d, *J* = 7.9 Hz, 2H), 5.96 (s, 1H), 5.91 (s, 2H), 5.75 (d, *J* = 39.5 Hz, 1H), 4.08 (broad s, 1H), 3.99 (d, *J* = 10.6 Hz, 2H), 3.78 (s, 6H), 2.37 (s, 3H).

^{19}F NMR (565 MHz, Chloroform-*d*) δ -110.53 (dt, *J* = 39.8 Hz, *J* = 10.4 Hz).

^{13}C NMR (151 MHz, Chloroform-*d*) δ 161.8, 156.4 (d, *J* = 266.5 Hz), 149.2, 137.1 (d, *J* = 2.4 Hz), 130.1 (d, *J* = 2.6 Hz), 129.2, 128.5 (d, *J* = 7.1 Hz), 106.9 (d, *J* = 6.8 Hz), 92.1, 90.5, 55.2, 45.5 (d, *J* = 33.3 Hz), 21.2.

IR (neat, cm^{-1}): ṽ: 3410, 2933, 1693, 1598, 1514, 1456, 1338, 1201, 1149, 1065, 861, 808, 681.

ESI-HRMS: mass spectrometry: m/z calcd for $C_{18}H_{20}O_2NFNa$ $[M+Na]^+$ 324.13703, measured 324.13641.

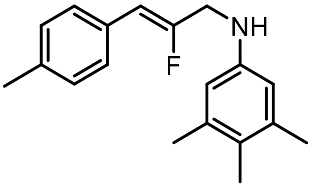

(*Z*)-*N*-(2-fluoro-3-(*p*-tolyl)allyl)-3,4,5-trimethylaniline

c26: The crude mixture was purified by SiO_2 gel column chromatography with pentane/EA (from 60:1). 56 mg product was obtained by 99% isolated yield as yellow oil.

^{1}H NMR (600 MHz, Chloroform-*d*) δ 7.29 (d, *J* = 7.8 Hz, 2H), 7.03 (d, *J* = 7.8 Hz, 2H), 6.30 (s, 2H), 5.63 (d, *J* = 39.8 Hz, 1H), 3.85 (d, *J* = 9.8 Hz, 2H), 3.67 (broad s, 1H), 2.24 (s, 3H), 2.13 (s, 6H), 1.98 (s, 3H).

^{19}F NMR (565 MHz, Chloroform-*d*) δ -110.17 (dt, *J* = 39.9 Hz, *J* = 9.6 Hz).

^{13}C NMR (151 MHz, Chloroform-*d*) δ 157.2 (d, *J* = 266.5 Hz), 144.8, 137.4, 136.9 (d, *J* = 2.3 Hz), 130.3 (d, *J* = 2.4 Hz), 129.2, 128.5 (d, *J* = 7.0 Hz), 124.9, 112.8, 106.4 (d, *J* = 6.6 Hz), 45.9 (d, *J* = 33.7 Hz), 21.3, 20.9, 14.5.

IR (neat, cm^{-1}): ṽ: 3408, 2918, 1907, 1694, 1609, 1500, 1443, 1328, 1216, 1136, 991, 837, 702.

ESI-HRMS: mass spectrometry: m/z calcd for $C_{19}H_{23}NF$ $[M+H]^+$ 284.18090, measured 284.18088.

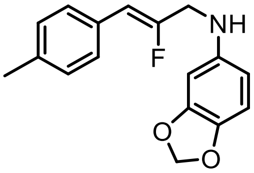

(*Z*)-*N*-(2-fluoro-3-(*p*-tolyl)allyl)benzo[*d*][1,3]dioxol-5-amine

c27: The crude mixture was purified by SiO_2 gel column chromatography with pentane/EA (from 40:1). 47 mg product was obtained by 82% isolated yield as brown oil.

^{1}H NMR (600 MHz, Chloroform-*d*) δ 7.30 (d, *J* = 7.9 Hz, 2H), 7.05 (d, *J* = 7.9 Hz, 2H), 6.59 (d, *J* = 8.3 Hz, 1H), 6.24 (d, *J* = 2.3 Hz, 1H), 6.05 (dd, *J* = 8.3, 2.3 Hz, 1H), 5.79 (s, 2H), 5.63 (d, *J* = 39.6 Hz, 1H), 3.85 (d, *J* = 10.9 Hz, 2H), 3.75 (broad s, 1H), 2.26 (s, 3H).

^{19}F NMR (565 MHz, Chloroform-*d*) δ -110.59 (dt, *J* = 39.7 Hz, *J* = 11.0 Hz).

^{13}C NMR (151 MHz, Chloroform-*d*) δ 156.5 (d, *J* = 266.5 Hz), 148.4, 142.8, 140.3, 137.1 (d, *J* = 2.2 Hz), 130.1 (d, *J* = 2.3 Hz), 129.2, 128.5 (d, *J* = 7.1 Hz), 108.6, 107.0 (d, *J* = 6.8 Hz), 105.1, 100.7, 96.5, 46.6 (d, *J* = 33.1 Hz), 21.2.

IR (neat, cm^{-1}): ṽ: 3417, 2884, 1693, 1624, 1493, 1292, 1202, 1106, 1037, 934, 809, 730.

ESI-HRMS: mass spectrometry: m/z calcd for $C_{17}H_{17}NF$ $[M+H]^+$ 286.12378, measured 286.12306.

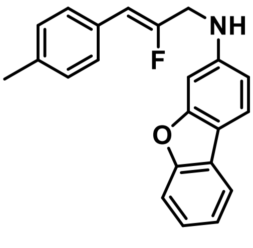

(*Z*)-*N*-(2-fluoro-3-(*p*-tolyl)allyl)dibenzo[*b*,*d*]furan-3-amine

c28: The crude mixture was purified by SiO_2 gel column chromatography with pentane/EA (from 40:1). 63 mg product was obtained by 95% isolated yield as yellow solid.

^{1}H NMR (600 MHz, Chloroform-*d*) δ 7.69 (d, *J* = 7.5 Hz, 1H), 7.61 (d, *J* = 8.3 Hz, 1H), 7.38 (d, *J* = 8.0 Hz, 1H), 7.29 (d, *J* = 8.0 Hz, 2H), 7.22 (t, *J* = 7.7 Hz, 1H), 7.17 (t, *J* = 7.4 Hz, 1H), 7.03 (d, *J* = 8.0 Hz, 2H), 6.74 (d, *J* = 2.0 Hz, 1H), 6.59 (dd, *J* = 8.4, 2.1 Hz, 1H), 5.66 (d, *J* = 39.5 Hz, 1H), 4.19 (broad s, 1H), 3.96 (d, *J* = 10.7 Hz, 2H), 2.23 (s, 3H).

^{19}F NMR (565 MHz, Chloroform-*d*) δ -110.67 (dt, *J* = 39.5 Hz, *J* = 10.8 Hz).

^{13}C NMR (151 MHz, Chloroform-*d*) δ 158.2, 156.1 (d, *J* = 266.5 Hz), 155.9, 147.7, 137.2 (d, *J* = 2.4 Hz), 130.0 (d, *J* = 2.6 Hz), 129.2, 128.5 (d, *J* = 7.1 Hz), 125.1, 124.9, 122.6, 121.2, 119.3, 115.3, 111.2, 110.2, 107.2 (d, *J* = 6.7 Hz), 95.1, 45.8 (d, *J* = 33.2 Hz), 21.3.

IR (neat, cm^{-1}): ṽ: 3417, 2917, 1693, 1636, 1499, 1424, 1339, 1257, 1159, 1011, 872, 747.

EI-HRMS: mass spectrometry: m/z calcd for $C_{22}H_{18}ONF$ $[M]^+$ 331.13669, measured 331.13666.

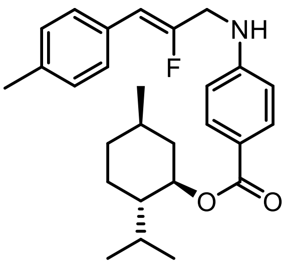

From DL- Menthol

(1*R*,2*S*,5*R*)-2-isopropyl-5-methylcyclohexyl 4-(((*Z*)-2-fluoro-3-(*p*-tolyl)allyl)amino)benzoate

c29: The crude mixture was purified by SiO_2 gel column chromatography with pentane/EA (from 20:1). 55 mg product was obtained by 65% isolated yield as yellow solid.

^{1}H NMR (600 MHz, Chloroform-*d*) δ 7.81 (d, *J* = 8.3 Hz, 2H), 7.27 (d, *J* = 7.8 Hz, 2H), 7.03 (d, *J* = 7.8 Hz, 2H), 6.55 (d, *J* = 8.4 Hz, 2H), 5.60 (d, *J* = 39.4 Hz, 1H), 4.79 (td, *J* = 10.9 Hz, *J* = 4.3 Hz, 1H), 4.43 (broad s, 1H), 3.93 (d, *J* = 10.1 Hz, 2H), 2.23 (s, 3H), 2.02 (broad d, *J* = 12.0 Hz, 1H), 1.88 (m, 1H), 1.62 (broad d, *J* = 11.4 Hz, 2H), 1.50 – 1.38 (m, 2H), 1.09 – 0.93 (m, 2H), 0.82 (dd+m, *J* = 6.7 Hz, *J* = 3.0 Hz, 7H), 0.70 (d, *J* = 7.0 Hz, 3H).

^{19}F NMR (565 MHz, Chloroform-*d*) δ -110.89 (dt, *J* = 39.7 Hz, *J* = 10.2 Hz).

^{13}C NMR (151 MHz, Chloroform-*d*) δ 166.2, 155.6 (d, *J* = 266.8 Hz), 150.9, 137.3 (d, *J* = 2.3 Hz), 131.5, 129.9 (d, *J* = 2.5 Hz), 129.2, 128.5 (d, *J* = 7.1 Hz), 120.1, 111.9, 107.2 (d, *J* = 6.7 Hz), 74.1, 47.4, 44.9 (d, *J* = 33.9 Hz), 41.2, 34.4, 31.5, 26.5, 23.8, 22.1, 21.3, 20.8, 16.6.

IR (neat, cm^{-1}): ṽ: 3363, 2952, 1672, 1599, 1527, 1338, 1267, 1172, 1112, 965, 903, 838, 727.

ESI-HRMS: mass spectrometry: m/z calcd for $C_{27}H_{34}O_2NFNa$ $[M+Na]^+$ 446.24658, measured 446.24504.

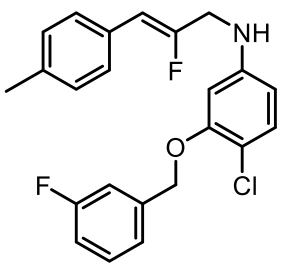

(*Z*)-4-chloro-*N*-(2-fluoro-3-(*p*-tolyl)allyl)-3-((3-fluorobenzyl)oxy)aniline

c30: The crude mixture was purified by SiO_2 gel column chromatography with pentane/EA (from 20:1). 21 mg product was obtained by 26% isolated yield as brown oil.

^{1}H NMR (600 MHz, Chloroform-*d*) δ 7.28 (d, *J* = 8.0 Hz, 2H), 7.24 (m, 1H), 7.13 – 7.08 (m, 2H), 7.05 (d, *J* = 7.9 Hz, 2H), 6.91 (td, *J* = 8.6 Hz, *J* = 2.5 Hz, 1H), 6.73 (d, *J* = 8.8 Hz, 1H), 6.66 (d, *J* = 2.9 Hz, 1H), 6.41 (dd, *J* = 8.9, 2.8 Hz, 1H), 5.61 (d, *J* = 39.5 Hz, 1H), 4.94 (s, 2H), 3.84 (d, *J* = 10.9 Hz, 2H), 3.78 (broad s, 1H), 2.25 (s, 3H).

^{19}F NMR (565 MHz, Chloroform-*d*) δ -110.75 (dt, *J* = 39.3, 10.9 Hz, 1F), -112.91 (m, 1F).

^{13}C NMR (151 MHz, Chloroform-*d*) δ 163.0 (d, *J* = 245.9 Hz), 156.1 (d, *J* = 266.7 Hz), 146.7, 142.7, 139.7 (d, *J* = 7.3 Hz), 137.2 (d, *J* = 2.3 Hz), 130.0 (d, *J* = 8.4 Hz), 130.0 (d, *J* = 2.5 Hz), 129.2, 128.5 (d, *J* = 7.0 Hz), 124.8, 122.7 (d, *J* = 2.9 Hz), 117.0, 115.3, 114.7 (d, *J* = 21.2 Hz), 114.2 (d, *J* = 22.2 Hz), 112.3, 107.1 (d, *J* = 6.8 Hz), 71.5 (d, *J* = 1.9 Hz), 46.0 (d, *J* = 33.1 Hz), 21.2.

IR (neat, cm^{-1}): ṽ: 3419, 2922, 2329, 1693, 1592, 1503, 1225, 1140, 1056, 907, 860, 783.

ESI-HRMS: mass spectrometry: m/z calcd for $C_{23}H_{20}ONClF_2Na$ $[M+Na]^+$ 422.10937, measured 422.10796.

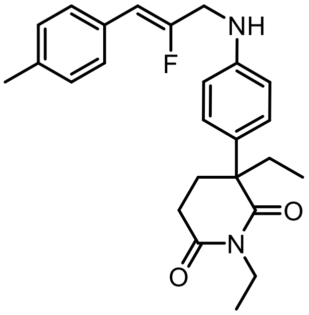

(*Z*)-1,3-diethyl-3-(4-((2-fluoro-3-(*p*-tolyl)allyl)amino)phenyl)piperidine-2,6-dione

c31: The crude mixture was purified by SiO_2 gel column chromatography with pentane/EA (from 5:1). 81 mg product was obtained by 99% isolated yield as brown oil.[4]

^{1}H NMR (600 MHz, Chloroform-*d*) δ 7.29 (d, *J* = 8.2 Hz, 2H), 7.04 (d, *J* = 7.9 Hz, 2H), 6.93 (d, *J* = 8.7 Hz, 2H), 6.55 (d, *J* = 8.7 Hz, 2H), 5.62 (d, *J* = 39.5 Hz, 1H), 4.03 (broad s, 1H), 3.88 (d, *J* = 10.4 Hz, 2H), 3.85 – 3.72 (m, 2H), 2.52 (dm, *J* = 17.8 Hz, 1H), 2.44 – 2.35 (m, 1H), 2.24 (s, 3H), 2.14 (dm, *J* = 14.0 Hz, 1H), 2.02 (td, *J* = 13.7 Hz, *J* = 4.7 Hz, 1H), 1.96 – 1.88 (m, 1H), 1.80 – 1.72 (m, 1H), 1.05 (t, *J* = 7.0 Hz, 3H), 0.75 (t, *J* = 7.4 Hz, 3H).

^{19}F NMR (565 MHz, Chloroform-*d*) δ -110.56 (dt, *J* = 39.4, 10.6 Hz).

^{13}C NMR (151 MHz, Chloroform-*d*) δ 175.4, 172.2, 156.3 (d, *J* = 266.5 Hz), 146.4, 137.1 (d, *J* = 2.3 Hz), 130.0 (d, *J* = 2.4 Hz), 129.2, 128.7, 128.5 (d, *J* = 6.9 Hz), 127.1, 113.3, 106.9 (d, *J* = 6.6 Hz), 50.5, 45.4 (d, *J* = 33.3 Hz), 35.2, 33.8, 30.0, 25.9, 21.3, 13.3, 9.1.

IR (neat, cm^{-1}): ṽ: 3396, 2972, 1910, 1666, 1529, 1455, 1355, 1214, 1122, 1045, 908, 861, 730.

ESI-HRMS: mass spectrometry: m/z calcd for $C_{25}H_{29}O_2N_2FNa$ $[M+Na]^+$ 431.21053, measured 431.20950.

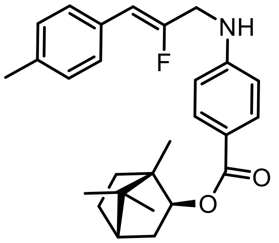

From DL-Isoborneol

(1*S*,2*S*,4*S*)-1,7,7-trimethylbicyclo[2.2.1]heptan-2-yl 4-(((*Z*)-2-fluoro-3-(*p*-tolyl)allyl)amino)benzoate

c32: The crude mixture was purified by SiO_2 gel column chromatography with pentane/EA (from 10:1). 70 mg product was obtained by 83% isolated yield as yellow solid.

^{1}H NMR (600 MHz, Chloroform-*d*) δ 7.83 (d, *J* = 8.3 Hz, 2H), 7.28 (d, *J* = 7.8 Hz, 2H), 7.04 (d, *J* = 7.8 Hz, 2H), 6.57 (d, *J* = 8.3 Hz, 2H), 5.61 (d, *J* = 39.4 Hz, 1H), 4.99 (d, *J* = 9.9 Hz, 1H), 4.42 (broad t, *J* = 5.3 Hz, 1H), 3.95 (dd, *J* = 10.4, 5.8 Hz, 2H), 2.41 – 2.32 (m, 1H), 2.24 (s, 3H), 2.08 – 2.00 (m, 1H), 1.75 – 1.66 (m, 1H), 1.63 (t, *J* = 4.6 Hz, 1H), 1.36 – 1.25 (m, 1H), 1.24 – 1.18 (m, 1H), 1.01 (dd, *J* = 13.8, 3.4 Hz, 1H), 0.87 (s, 3H), 0.81 (d, *J* = 5.4 Hz, 6H).
^{19}F NMR (565 MHz, Chloroform-*d*) δ -110.96 (dt, *J* = 39.4, 10.3 Hz).
^{13}C NMR (151 MHz, Chloroform-*d*) δ 166.9, 155.6 (d, *J* = 266.8 Hz), 150.9, 137.3 (d, *J* = 2.1 Hz), 131.5, 129.8 (d, *J* = 2.5 Hz), 129.2, 128.5 (d, *J* = 7.1 Hz), 120.2, 112.0, 107.3 (d, *J* = 6.6 Hz), 79.7, 49.1, 47.8, 45.1, 44.9 (d, *J* = 33.7 Hz), 37.0, 28.1, 27.5, 21.3, 19.8, 19.0, 13.6.
IR (neat, cm^{-1}): ṽ: 3360, 2953, 1907, 1671, 1530, 1450, 1341, 1285, 1230, 1170, 1114, 984, 770.
ESI-HRMS: mass spectrometry: m/z calcd for $C_{27}H_{32}O_2NFNa$ $[M+Na]^+$ 444.23093, measured 444.22995.

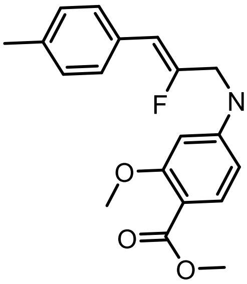

methyl (*Z*)-4-((2-fluoro-3-(*p*-tolyl)allyl)amino)-2-methoxybenzoate

c33: The crude mixture was purified by SiO_2 gel column chromatography with pentane/EA (from 10:1). 58 mg product was obtained by 88% isolated yield as yellow oil.
^{1}H NMR (600 MHz, Chloroform-*d*) δ 7.29 (d, *J* = 8.0 Hz, 2H), 7.08 (d, *J* = 3.0 Hz, 1H), 7.05 (d, *J* = 7.9 Hz, 2H), 6.79 (d, *J* = 8.9 Hz, 1H), 6.74 (dd, *J* = 8.9, 3.0 Hz, 1H), 5.63 (d, *J* = 39.6 Hz, 1H), 3.89 (d, *J* = 11.1 Hz, 2H), 3.80 (s, 3H), 3.75 (s, 3H), 2.25 (s, 3H).
^{19}F NMR (565 MHz, Chloroform-*d*) δ -110.71 (dt, *J* = 39.4 Hz, *J* = 11.0 Hz).
^{13}C NMR (151 MHz, Chloroform-*d*) δ 166.8, 156.4 (d, *J* = 266.5 Hz), 152.2, 140.8, 137.1 (d, *J* = 2.3 Hz), 130.0 (d, *J* = 2.6 Hz), 129.2, 128.5 (d, *J* = 7.2 Hz), 120.8, 118.4, 116.4, 114.3, 107.1 (d, *J* = 6.9 Hz), 56.9, 52.1, 46.2 (d, *J* = 32.8 Hz), 21.2.
IR (neat, cm^{-1}): ṽ: 3373, 2925, 1799, 1687, 1617, 1500, 1436, 1298, 1225, 1180, 1088, 1021, 877, 733.
ESI-HRMS: mass spectrometry: m/z calcd for $C_{19}H_{20}O_3NFNa$ $[M+Na]^+$ 352.13194, measured 352.13203.

6. General procedure for d

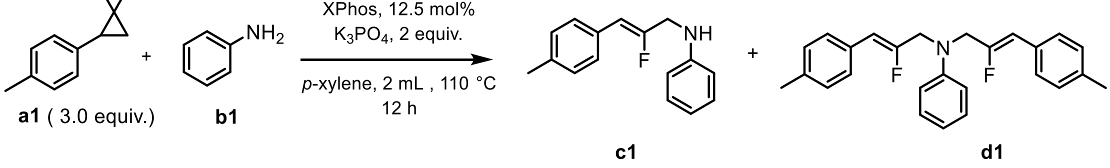

Under N_2 atmosphere, XPhos (11.9 mg, 0.025 mmol) and $Pd(dba)_2$ (5.7 mg, 0.01 mmol), gem-difluorocyclopropanes **a1** (0.6 mmol), aniline **b1** (0.2 mmol) , K_3PO_4 (2.0 equiv., 84.8 mg) were dissolved in 2 mL *p*-xylene, then the mixture was stirred at 110 ^{o}C for about 12 h to the starting

material was consumed (monitored by TLC), the mixture was filtered through celite and the filtrate was concentrated to dryness. A portion of the residue was analyzed with ^1H NMR to determine selectivity and recovered. The crude was purified by column chromatography to give the products **d**.

7. Product characterization of d

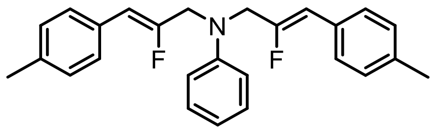

***N*,*N*-bis((*Z*)-2-fluoro-3-(*p*-tolyl)allyl)aniline**

d1: The crude mixture was purified by SiO_2 gel column chromatography with pentane/EA (from 100:1). 70 mg product was obtained by 90% isolated yield as yellow oil.

^{1}H NMR (600 MHz, Chloroform-d) δ 7.28 (d, *J* = 8.0 Hz, 4H), 7.16 (broad t, *J* = 7.7 Hz, 2H), 7.03 (d, *J* = 7.9 Hz, 4H), 6.81 (d, *J* = 8.2 Hz, 2H), 6.71 (t, *J* = 7.3 Hz, 1H), 5.55 (d, *J* = 39.7 Hz, 2H), 4.12 (d, *J* = 7.8 Hz, 4H), 2.24 (s, 6H).

^{19}F NMR (565 MHz, Chloroform-d) δ -110.48 (dt, *J* = 39.8 Hz, *J* = 7.9 Hz).

^{13}C NMR (151 MHz, Chloroform-d) δ 155.4 (d, *J* = 269.2 Hz), 148.0, 137.2 (d, *J* = 2.2 Hz), 130.0 (d, *J* = 2.3 Hz), 129.4, 129.2, 128.6 (d, *J* = 7.1 Hz), 118.2, 113.2, 107.1 (d, *J* = 6.2 Hz), 51.5 (d, *J* = 34.2 Hz), 21.3.

IR (neat, cm^{-1}): ṽ: 3409, 2920, 1909, 1691, 1589, 1504, 1378, 1218, 1129, 951, 906, 733.

ESI-HRMS: mass spectrometry: m/z calcd for $C_{26}H_{25}NF_2Na$ $[M+Na]^+$ 412.18473, measured 412.18445.

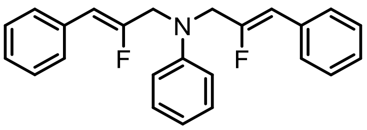

***N*,*N*-bis((*Z*)-2-fluoro-3-phenylallyl)aniline**

d2: The crude mixture was purified by SiO_2 gel column chromatography with pentane/EA (from 100:1). 68 mg product was obtained by 94% isolated yield as brown solid.

^{1}H NMR (600 MHz, Chloroform-*d*) δ 7.40 (d, *J* = 8.0 Hz, 4H), 7.24 (t, *J* = 7.7 Hz, 4H), 7.21 – 7.17 (m, 2H), 7.17 – 7.14 (m, 2H), 6.84 (d, *J* = 8.4 Hz, 2H), 6.74 (t, *J* = 7.3 Hz, 1H), 5.60 (d, *J* = 39.5 Hz, 2H), 4.16 (d, *J* = 7.5 Hz, 4H).

^{19}F NMR (565 MHz, Chloroform-*d*) δ -109.57 (dt, *J* = 39.4 Hz, *J* = 7.8 Hz).

^{13}C NMR (151 MHz, Chloroform-*d*) δ 155.9 (d, *J* = 270.2 Hz), 147.8, 132.8 (d, *J* = 2.3 Hz), 129.4, 128.6 (d, *J* = 7.2 Hz), 128.5, 127.4 (d, *J* = 2.1 Hz), 118.3, 113.2, 107.1 (d, *J* = 6.0 Hz), 51.5 (d, *J* = 34.3 Hz).

IR (neat, cm^{-1}): ṽ:3028, 2923, 1910, 1690, 1598, 1499, 1263, 1276, 1178, 1126, 959, 861, 747.

EI-HRMS: mass spectrometry: m/z calcd for $C_{24}H_{21}NF_2$ $[M]^+$ 361.16366, measured 361.16350.

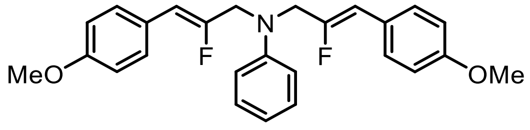

***N*,*N*-bis((*Z*)-2-fluoro-3-(4-methoxyphenyl)allyl)aniline**

d3: The crude mixture was purified by SiO_2 gel column chromatography with pentane/EA (from 50:1). 59 mg product was obtained by 70% isolated yield as yellow solid.

^{1}H NMR (600 MHz, Chloroform-*d*) δ 7.34 (d, *J* = 8.6 Hz, 4H), 7.17 (t, *J* = 7.8 Hz, 2H), 6.82 (d, *J* = 8.2 Hz, 2H), 6.77 (d, *J* = 8.5 Hz, 4H), 6.72 (t, *J* = 7.3 Hz, 1H), 5.53 (d, *J* = 39.8 Hz, 2H), 4.13 (d, *J* = 8.0 Hz, 4H), 3.71 (s, 6H).

^{19}F NMR (565 MHz, Chloroform-*d*) δ -112.36 (dt, *J* = 39.9 Hz, *J* = 8.1 Hz).

^{13}C NMR (151 MHz, Chloroform-*d*) δ 158.8 (d, *J* = 2.5 Hz), 154.5 (d, *J* = 267.8 Hz), 148.0, 129.9 (d, *J* = 7.2 Hz), 129.3, 125.6 (d, *J* = 2.2 Hz), 118.1, 113.9, 113.2, 106.7 (d, *J* = 6.4 Hz), 55.3, 51.4 (d, *J* = 34.1 Hz).

IR (neat, cm^{-1}): ṽ: 3046, 2930, 1900, 1692, 1601, 1504, 1350, 1247, 1175, 1030, 951, 858, 745.

ESI-HRMS: mass spectrometry: m/z calcd for $C_{26}H_{25}O_2NF_2Na$ $[M+Na]^+$ 444.17456, measured 444.17416.

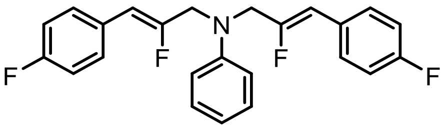

***N*,*N*-bis((*Z*)-2-fluoro-3-(4-fluorophenyl)allyl)aniline**

d4: The crude mixture was purified by SiO_2 gel column chromatography with pentane/EA (from 100:1). 46 mg product was obtained by 58% isolated yield as yellow solid.

^{1}H NMR (600 MHz, Chloroform-*d*) δ 7.36 (dd, *J* = 8.6 Hz, *J* = 5.5 Hz, 4H), 7.19 (t, *J* = 7.3 Hz, 2H), 6.92 (t, *J* = 8.7 Hz, 4H), 6.82 (d, *J* = 8.2 Hz, 2H), 6.74 (t, *J* = 7.3 Hz, 1H), 5.56 (d, *J* = 39.1 Hz, 2H), 4.14 (d, *J* = 7.5 Hz, 4H).

^{19}F NMR (565 MHz, Chloroform-*d*) δ -110.77 (dt, *J* = 39.2 Hz, *J* = 7.7 Hz, 2F), -113.89 (m, 2F).

^{13}C NMR (151 MHz, Chloroform-*d*) δ 161.8 (dd, *J* = 247.5, 3.2 Hz), 155.5 (dd, *J* = 269.7, 2.5 Hz), 147.7, 130.3 (t, *J* = 7.6 Hz), 129.4, 128.9 (t, *J* = 3.0 Hz), 118.4, 115.4 (d, *J* = 21.3 Hz), 113.1, 106.1 (d, *J* = 6.2 Hz), 51.5 (d, *J* = 34.1 Hz).

IR (neat, cm^{-1}): ṽ: 3382, 2922, 1895, 1692, 1598, 1504, 1379, 1225, 1129, 1017, 956, 857, 744.

APCI-HRMS: mass spectrometry: m/z calcd for $C_{24}H_{20}NF_4$ $[M+H]^+$ 398.15264, measured 398.15215.

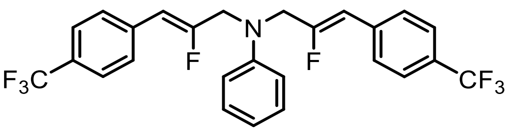

***N*,*N*-bis((*Z*)-2-fluoro-3-(4-(trifluoromethyl)phenyl)allyl)aniline**

d5: The crude mixture was purified by SiO_2 gel column chromatography with pentane/EA (from 50:1). 73 mg product was obtained by 73% isolated yield as brown solid.

^{1}H NMR (600 MHz, Chloroform-*d*) δ 7.47 (s, 8H), 7.22 – 7.18 (m, 2H), 6.82 (d, *J* = 7.8 Hz, 1H), 6.77 (t, *J* = 7.3 Hz, 1H), 5.65 (d, *J* = 38.6 Hz, 2H), 4.18 (d, *J* = 6.9 Hz, 4H).

^{19}F NMR (564 MHz, Chloroform-*d*) δ -62.63 (s, 6F), -106.46 (dt, *J* = 38.7 Hz, *J* = 7.1 Hz, 2F).

^{13}C NMR (151 MHz, Chloroform-*d*) δ 157.6 (d, *J* = 273.6 Hz), 147.4, 136.2, 129.5, 129.2 (qd, *J* = 32.2 Hz, *J* = 1.7 Hz), 128.7 (d, *J* = 7.3 Hz), 125.4 (q, *J* = 3.7 Hz), 124.1 (q, *J* = 271.9 Hz), 118.8, 113.2, 106.1 (d, *J* = 5.6 Hz), 51.7 (d, *J* = 34.2 Hz).

IR (neat, cm^{-1}): ṽ: 2924, 1932, 1691, 1599, 1502, 1415, 1381, 1322, 1215, 1114, 1016, 946, 862, 752.

ESI-HRMS: mass spectrometry: m/z calcd for $C_{26}H_{20}NF_8$ $[M+H]^+$ 498.14625, measured 498.14532.

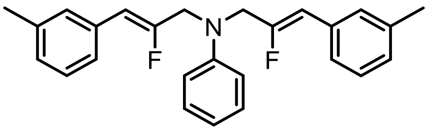

***N*,*N*-bis((*Z*)-2-fluoro-3-(*m*-tolyl)allyl)aniline**

d6: The crude mixture was purified by SiO_2 gel column chromatography with pentane/EA (from 100:1). 62 mg product was obtained by 80% isolated yield as brown oil.

^{1}H NMR (600 MHz, Chloroform-*d*) δ 7.23 – 7.20 (m, 4H), 7.20 – 7.16 (m, 2H), 7.13 (t, *J* = 7.9 Hz, 2H), 6.97 (d, *J* = 7.6 Hz, 2H), 6.83 (d, *J* = 7.6 Hz, 2H), 6.73 (t, *J* = 7.4 Hz, 1H), 5.57 (d, *J* = 39.7 Hz, 2H), 4.15 (d, *J* = 7.6 Hz, 4H), 2.25 (s, 6H).

^{19}F NMR (565 MHz, Chloroform-*d*) δ -109.63 (dt, *J* = 39.4 Hz, *J* = 7.9 Hz).

^{13}C NMR (151 MHz, Chloroform-*d*) δ 155.7 (d, *J* = 270.2 Hz), 147.9, 138.1, 132.7 (d, *J* = 2.4 Hz), 129.4, 129.3, 128.4, 128.1 (d, *J* = 2.0 Hz), 125.7 (d, *J* = 7.4 Hz), 118.3, 113.2, 107.2 (d, *J* = 6.0 Hz), 51.5 (d, *J* = 34.2 Hz), 21.4.

IR (neat, cm^{-1}): ṽ: 3384, 2921, 1925, 1690, 1598, 1501, 1379, 1302, 1219, 1129, 991, 949, 780.

APCI-HRMS: mass spectrometry: m/z calcd for $C_{26}H_{26}NF_2$ $[M+H]^+$ 390.20278, measured 390.20290.

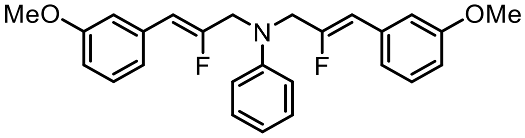

***N*,*N*-bis((*Z*)-2-fluoro-3-(3-methoxyphenyl)allyl)aniline**

d7: The crude mixture was purified by SiO_2 gel column chromatography with pentane/EA (from 50:1). 82 mg product was obtained by 97% isolated yield as yellow solid.

^{1}H NMR (600 MHz, Chloroform-*d*) δ 7.20 – 7.16 (m, 2H), 7.14 (t, *J* = 7.9 Hz, 2H), 6.99 – 6.94 (m, 4H), 6.82 (d, *J* = 8.3 Hz, 2H), 6.75 – 6.69 (m, 3H), 5.57 (d, *J* = 39.2 Hz, 2H), 4.13 (d, *J* = 7.5 Hz, 4H), 3.69 (s, 6H).

^{19}F NMR (565 MHz, Chloroform-*d*) δ -108.74 (dt, *J* = 39.2 Hz, *J* = 7.8 Hz).

^{13}C NMR (151 MHz, Chloroform-*d*) δ 159.7, 156.1 (d, *J* = 270.9 Hz), 147.8, 134.1 (d, *J* = 2.3 Hz), 129.5, 129.4, 121.3 (d, *J* = 6.9 Hz), 118.4, 113.8 (d, *J* = 7.6 Hz), 113.4 (d, *J* = 1.4 Hz), 113.2, 107.1 (d, *J* = 5.5 Hz), 55.2, 51.5 (d, *J* = 34.1 Hz).

IR (neat, cm^{-1}): ṽ: 3003, 2936, 1921, 1690, 1596, 1498, 1432, 1380, 1293, 1221, 1162, 1045, 907, 778.

ESI-HRMS: mass spectrometry: m/z calcd for $C_{26}H_{26}O_2NF_2$ $[M+H]^+$ 422.19261, measured 422.19217.

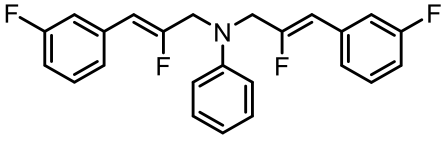

***N*,*N*-bis((*Z*)-2-fluoro-3-(3-fluorophenyl)allyl)aniline**

d8: The crude mixture was purified by SiO_2 gel column chromatography with pentane/EA (from 100:1). 56 mg product was obtained by 70% isolated yield as brown oil.

^{1}H NMR (600 MHz, Chloroform-*d*) δ 7.22 – 7.13 (m, 6H), 7.10 (d, *J* = 7.8 Hz, 2H), 6.85 (td, *J* = 8. 4 Hz, *J* = 2.5 Hz, 2H), 6.81 (d, *J* = 8.2 Hz, 2H), 6.75 (t, *J* = 7.3 Hz, 1H), 5.58 (d, *J* = 38.6 Hz, 2H), 4.15 (d, *J* = 7.2 Hz, 4H).
^{19}F NMR (565 MHz, Chloroform-*d*) δ -107.57 (dt, *J* = 38.4 Hz, *J* = 7.3 Hz, 2F), -113.11 (m, 2F).
^{13}C NMR (151 MHz, Chloroform-*d*) δ 162.8 (d, *J* = 244.8 Hz), 156.8 (d, *J* = 272.1 Hz), 147.5, 134.8 (dd, *J* = 8.5, 2.1 Hz), 129.9 (d, *J* = 8.4 Hz), 129.5, 124.4 (dd, *J* = 6.5, 2.9 Hz), 118.6, 115.3 (dd, *J* = 22.7, 8.6 Hz), 114.3 (dd, *J* = 20.9 Hz, *J* = 1.4 Hz), 113.2, 106.3 (dd, *J* = 5.7, 2.6 Hz), 51.6 (d, *J* = 34.2 Hz).
IR (neat, cm^{-1}): ṽ: 3383, 2922, 1923, 1691, 1584, 1441, 1380, 1281, 1221, 1151, 1079, 966, 779.
APCI-HRMS: mass spectrometry: m/z calcd for $C_{24}H_{20}NF_4$ $[M+H]^+$ 398.15264, measured 398.15290.

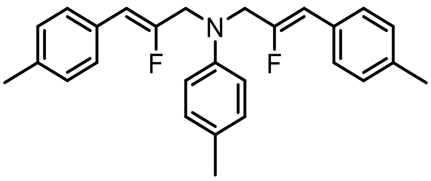

***N*,*N*-bis((*Z*)-2-fluoro-3-(*p*-tolyl)allyl)-4-methylaniline**

d9: The crude mixture was purified by SiO_2 gel column chromatography with pentane/EA (from 100:1). 73 mg product was obtained by 90% isolated yield as brown oil.
^{1}H NMR (600 MHz, Chloroform-*d*) δ 7.28 (d, *J* = 8.0 Hz, 4H), 7.03 (d, *J* = 7.9 Hz, 4H), 6.97 (d, *J* = 8.3 Hz, 2H), 6.73 (d, *J* = 8.6 Hz, 2H), 5.54 (d, *J* = 39.8 Hz, 2H), 4.09 (d, *J* = 7.9 Hz, 4H), 2.24 (s, 6H), 2.17 (s, 3H).
^{19}F NMR (565 MHz, Chloroform-*d*) δ -110.29 (dt, *J* = 39.9 Hz, *J* = 8.0 Hz).
^{13}C NMR (151 MHz, Chloroform-*d*) δ 155.6 (d, *J* = 269.4 Hz), 145.8, 137.1 (d, *J* = 2.2 Hz), 130.1 (d, *J* = 2.0 Hz), 129.9, 129.2, 128.5 (d, *J* = 6.9 Hz), 127.5, 113.5, 107.0 (d, *J* = 6.1 Hz), 51.7 (d, *J* = 34.0 Hz), 21.3, 20.3.
IR (neat, cm^{-1}): ṽ: 3385, 2920, 1906, 1692, 1615, 1515, 1377, 1214, 1131, 949, 906, 804, 730.
APCI-HRMS: mass spectrometry: m/z calcd for $C_{27}H_{28}NF_2$ $[M+H]^+$ 404.21843, measured 404.21861.

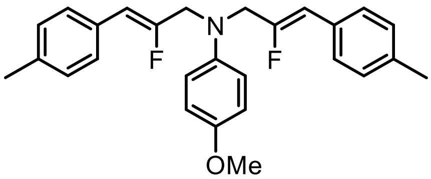

***N*,*N*-bis((*Z*)-2-fluoro-3-(*p*-tolyl)allyl)-4-methoxyaniline**

d10: The crude mixture was purified by SiO_2 gel column chromatography with pentane/EA (from 80:1). 78 mg product was obtained by 93% isolated yield as brown solid.
^{1}H NMR (600 MHz, Chloroform-*d*) δ 7.28 (d, *J* = 8.1 Hz, 4H), 7.03 (d, *J* = 7.9 Hz, 4H), 6.80 – 6.72 (second order m, 4H), 5.54 (d, *J* = 39.8 Hz, 2H), 4.05 (d, *J* = 9.0 Hz, 4H), 3.65 (s, 3H), 2.23 (s, 6H).
^{19}F NMR (565 MHz, Chloroform-*d*) δ -109.90 (dt, *J* = 39.9 Hz, *J* = 8.9 Hz).
^{13}C NMR (151 MHz, Chloroform-*d*) δ 155.9 (d, *J* = 269.5 Hz), 152.7, 142.5, 137.1 (d, *J* = 2.0 Hz), 130.1 (d, *J* = 2.2 Hz), 129.2, 128.5 (d, *J* = 7.0 Hz), 115.4, 114.8, 107.3 (d, *J* = 6.2 Hz), 55.7, 52.3 (d, *J* = 33.2 Hz), 21.3.
IR (neat, cm^{-1}): ṽ: 3374, 2914, 1912, 1690, 1611, 1508, 1377, 1286, 1243, 1153, 1030, 909, 814.
ESI-HRMS: mass spectrometry: m/z calcd for $C_{27}H_{28}ONF_2$ $[M+H]^+$ 420.21335, measured 420.21279.

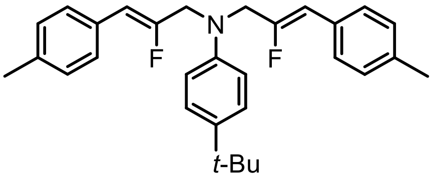

4-(*tert*-butyl)-*N*,*N*-bis((*Z*)-2-fluoro-3-(*p*-tolyl)allyl)aniline

d11: The crude mixture was purified by SiO_2 gel column chromatography with pentane/EA (from 100:1). 82 mg product was obtained by 92% isolated yield as brown oil.

^{1}H NMR (600 MHz, Chloroform-*d*) δ 7.29 (d, *J* = 8.2 Hz, 4H), 7.19 (d, *J* = 8.8 Hz, 2H), 7.03 (d, *J* = 7.9 Hz, 4H), 6.77 (d, *J* = 8.8 Hz, 2H), 5.57 (d, *J* = 39.8 Hz, 2H), 4.10 (d, *J* = 7.9 Hz, 4H), 2.24 (s, 6H), 1.20 (s, 9H).

^{19}F NMR (565 MHz, Chloroform-*d*) δ -110.28 (dt, *J* = 40.0 Hz, *J* = 8.0 Hz).

^{13}C NMR (151 MHz, Chloroform-*d*) δ 155.6 (d, *J* = 269.4 Hz), 145.7, 140.8, 137.1 (d, *J* = 1.9 Hz), 130.1 (d, *J* = 2.0 Hz), 129.2, 128.6 (d, *J* = 7.0 Hz), 126.2, 112.9, 107.0 (d, *J* = 6.0 Hz), 51.6 (d, *J* = 34.1 Hz), 33.9, 31.5, 21.3.

IR (neat, cm^{-1}): ṽ: 3390, 2958, 1907, 1692, 1612, 1516, 1452, 1367, 1208, 1130, 954, 906, 810.

ESI-HRMS: mass spectrometry: m/z calcd for $C_{30}H_{33}NF_2Na$ $[M+Na]^+$ 468.24733, measured 468.24661.

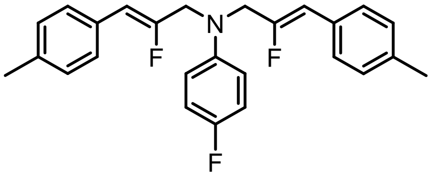

4-fluoro-*N*,*N*-bis((*Z*)-2-fluoro-3-(*p*-tolyl)allyl)aniline

d12: The crude mixture was purified by SiO_2 gel column chromatography with pentane/EA (from 100:1). 67 mg product was obtained by 82% isolated yield as brown oil.

^{1}H NMR (600 MHz, Chloroform-*d*) δ 7.29 (d, *J* = 8.0 Hz, 4H), 7.05 (d, *J* = 7.9 Hz, 4H), 6.86 (t, *J* = 8.7 Hz, 2H), 6.78 – 6.73 (m, 2H), 5.54 (d, *J* = 39.6 Hz, 2H), 4.08 (d, *J* = 8.9 Hz, 4H), 2.25 (s, 6H).

^{19}F NMR (564 MHz, Chloroform-*d*) δ -110.43 (dt, *J* = 39.7 Hz, *J* = 8.8 Hz, 2F), -126.96 (m, 1F).

^{13}C NMR (151 MHz, Chloroform-*d*) δ 156.3 (d, *J* = 237.2 Hz), 155.3 (d, *J* = 269.3 Hz), 144.6 (d, *J* = 2.2 Hz), 137.3 (d, *J* = 2.3 Hz), 129.9 (d, *J* = 2.3 Hz), 129.2, 128.5 (d, *J* = 7.1 Hz), 115.7 (d, *J* = 22.2 Hz), 114.8 (d, *J* = 7.5 Hz), 107.4 (d, *J* = 6.3 Hz), 52.1 (d, *J* = 33.3 Hz), 21.3.

IR (neat, cm^{-1}): ṽ: 3380, 2019, 1906, 1687, 1611, 1508, 1438, 1367, 1218, 1142, 937, 811, 734.

EI-HRMS: mass spectrometry: m/z calcd for $C_{26}H_{24}NF_3$ $[M]^+$ 407.18554, measured 407.18525.

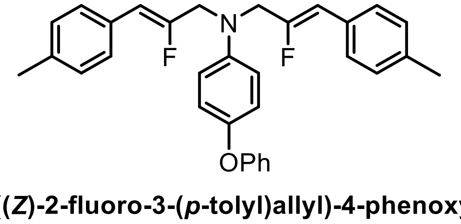

***N*,*N*-bis((*Z*)-2-fluoro-3-(*p*-tolyl)allyl)-4-phenoxyaniline**

d13: The crude mixture was purified by SiO_2 gel column chromatography with pentane/EA (from 80:1). 70 mg product was obtained by 73% isolated yield as brown oil.

¹H NMR (600 MHz, Chloroform-*d*) δ 7.45 (d, *J* = 8.0 Hz, 4H), 7.37 – 7.32 (m, 2H), 7.20 (d, *J* = 7.9 Hz, 4H), 7.08 (t, *J* = 7.3 Hz, 1H), 7.04 – 6.99 (m, 4H), 6.97 – 6.92 (m, 2H), 5.72 (d, *J* = 39.6 Hz, 2H), 4.25 (d, *J* = 8.9 Hz, 4H), 2.40 (s, 6H).

¹⁹F NMR (565 MHz, Chloroform-*d*) δ -110.27 (dt, *J* = 39.4 Hz, *J* = 8.9 Hz).

¹³C NMR (151 MHz, Chloroform-*d*) δ 158.7, 155.4 (d, *J* = 269.2 Hz), 148.6, 144.6, 137.3 (d, *J* = 2.1 Hz), 130.0 (d, *J* = 2.1 Hz), 129.6, 129.3, 128.6 (d, *J* = 7.2 Hz), 122.3, 120.8, 117.6, 114.8, 107.4 (d, *J* = 6.2 Hz), 52.0 (d, *J* = 33.3 Hz), 21.3.

IR (neat, cm^{-1}): ṽ: 3030, 2921, 1906, 1691, 1591, 1508, 1378, 1287, 1234, 1131, 1023, 952, 731.

ESI-HRMS: mass spectrometry: m/z calcd for $C_{32}H_{30}ONF_2$ $[M+H]^+$ 482.22900, measured 482.22859.

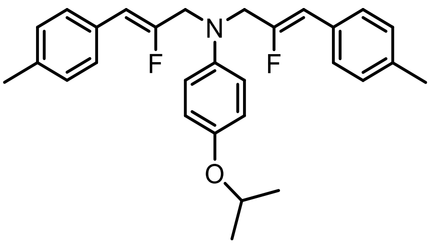

***N*,*N*-bis((*Z*)-2-fluoro-3-(*p*-tolyl)allyl)-4-isopropoxyaniline**

d14: The crude mixture was purified by SiO_2 gel column chromatography with pentane/EA (from 80:1). 75 mg product was obtained by 84% isolated yield as brown oil.

¹H NMR (600 MHz, Chloroform-*d*) δ 7.29 (d, *J* = 8.1 Hz, 4H), 7.04 (d, *J* = 7.9 Hz, 4H), 6.77 – 6.70 (m, 4H), 5.55 (d, *J* = 39.7 Hz, 2H), 4.30 (sept, *J* = 6.1 Hz, 1H), 4.05 (d, *J* = 8.8 Hz, 4H), 2.24 (s, 6H), 1.21 (d, *J* = 6.1 Hz, 6H).

¹⁹F NMR (565 MHz, Chloroform-*d*) δ -109.93 (dt, *J* = 39.8 Hz, *J* = 8.9 Hz).

¹³C NMR (151 MHz, Chloroform-*d*) δ 155.8 (d, *J* = 269.5 Hz), 150.8, 142.6, 137.1 (d, *J* = 2.2 Hz), 130.1 (d, *J* = 2.1 Hz), 129.2, 128.5 (d, *J* = 7.1 Hz), 117.5, 115.2, 107.2 (d, *J* = 6.1 Hz), 70.8, 52.2 (d, *J* = 33.3 Hz), 22.3, 21.3.

IR (neat, cm^{-1}): ṽ: 3384, 2975, 1908, 1691, 1612, 1509, 1449, 1375, 1238, 1126, 1040, 953, 860, 731.

EI-HRMS: mass spectrometry: m/z calcd for $C_{29}H_{31}ONF_2$ $[M]^+$ 447.23682, measured 447.23659.

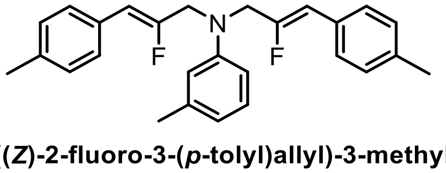

***N*,*N*-bis((*Z*)-2-fluoro-3-(*p*-tolyl)allyl)-3-methylaniline**

d15: The crude mixture was purified by SiO_2 gel column chromatography with pentane/EA (from 100:1). 72 mg product was obtained by 89% isolated yield as brown oil.

¹H NMR (600 MHz, Chloroform-*d*) δ 7.45 (d, *J* = 7.9 Hz, 4H), 7.23 – 7.17 (m, 5H), 6.81 – 6.77 (m, 2H), 6.70 (d, *J* = 7.5 Hz, 1H), 5.71 (d, *J* = 39.8 Hz, 2H), 4.27 (d, *J* = 7.5 Hz, 4H), 2.40 (s, 6H), 2.39 (s, 3H).

¹⁹F NMR (565 MHz, Chloroform-*d*) δ -110.40 (dt, *J* = 39.7 Hz, *J* = 7.6 Hz).

¹³C NMR (151 MHz, Chloroform-*d*) δ 155.5 (d, *J* = 269.2 Hz), 148.1, 139.1, 137.2 (d, *J* = 2.1 Hz), 130.1 (d, *J* = 2.5 Hz), 129.2, 128.6 (d, *J* = 7.1 Hz), 119.2, 113.9, 110.5, 107.0 (d, *J* = 6.1 Hz), 51.4 (d, *J* = 34.4 Hz), 22.0, 21.3.

IR (neat, cm^{-1}): ṽ: 3388, 2920, 1907, 1691, 1602, 1497, 1374, 1241, 1179, 1129, 952, 858, 730.

ESI-HRMS: mass spectrometry: m/z calcd for $C_{27}H_{27}NF_2Na$ $[M+Na]^+$ 426.20038, measured 426.19959.

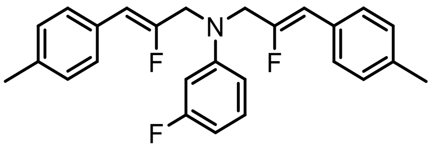

3-fluoro-*N*,*N*-bis((*Z*)-2-fluoro-3-(*p*-tolyl)allyl)aniline

d16: The crude mixture was purified by SiO_2 gel column chromatography with pentane/EA (from 100:1). 70 mg product was obtained by 86% isolated yield as brown oil.

^{1}H NMR (600 MHz, Chloroform-*d*) δ 7.29 (d, *J* = 7.9 Hz, 4H), 7.09 (≈q, *J* = 7.6 Hz, 1H), 7.05 (d, *J* = 7.9 Hz, 4H), 6.57 (d, *J* = 8.4 Hz, 1H), 6.52 (d, *J* = 12.4 Hz, 1H), 6.41 (t, *J* = 8.2 Hz, 1H), 5.55 (d, *J* = 39.5 Hz, 2H), 4.12 (d, *J* = 8.5 Hz, 4H), 2.25 (s, 6H).

^{19}F NMR (565 MHz, Chloroform-*d*) δ -110.81 (dt, *J* = 39.2 Hz, *J* = 8.8 Hz, 2F), -111.91 (dt, *J* = 12.4 Hz, *J* = 7.5 Hz, 1F).

^{13}C NMR (151 MHz, Chloroform-*d*) δ 164.0 (d, *J* = 243.2 Hz), 154.8 (d, *J* = 268.9 Hz), 149.7 (d, *J* = 10.4 Hz), 137.3 (d, *J* = 2.2 Hz), 130.4 (d, *J* = 10.2 Hz), 129.8 (d, *J* = 2.3 Hz), 129.2, 128.5 (d, *J* = 7.1 Hz), 108.8 (d, *J* = 2.3 Hz), 107.4 (d, *J* = 6.3 Hz), 104.7 (d, *J* = 21.3 Hz), 100.5 (d, *J* = 26.2 Hz), 51.5 (d, *J* = 33.9 Hz), 21.3.

IR (neat, cm^{-1}): ṽ: 3027, 2922, 1906, 1691, 1615, 1498, 1376, 1249, 1131, 1042, 994, 906, 860.

ESI-HRMS: mass spectrometry: m/z calcd for $C_{26}H_{24}NF_3Na$ $[M+Na]^+$ 430.17531, measured 430.17443.

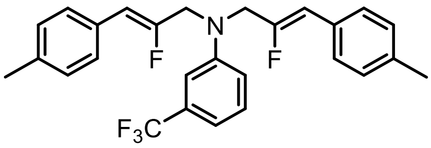

***N*,*N*-bis((*Z*)-2-fluoro-3-(*p*-tolyl)allyl)-3-(trifluoromethyl)aniline**

d17: The crude mixture was purified by SiO_2 gel column chromatography with pentane/EA (from 80:1). 80 mg product was obtained by 87% isolated yield as brown oil.

^{1}H NMR (600 MHz, Chloroform-*d*) δ 7.29 (d, *J* = 7.9 Hz, 4H), 7.24 (t, *J* = 8.0 Hz, 1H), 7.04 (d+m, *J* = 8.2 Hz, 5H), 6.95 (d, *J* = 8.2 Hz, 2H), 5.56 (d, *J* = 39.3 Hz, 2H), 4.15 (d, *J* = 9.3 Hz, 4H), 2.24 (s, 6H).

^{19}F NMR (564 MHz, Chloroform-*d*) δ -62.73 (s, 3F), -110.91 (dt, *J* = 39.4 Hz, *J* = 9.4 Hz, 2F).

^{13}C NMR (151 MHz, Chloroform-*d*) δ 154.6 (d, *J* = 268.8 Hz), 148.2, 137.5 (d, *J* = 2.2 Hz), 131.7 (q, *J* = 31.7 Hz), 129.8, 129.7 (d, *J* = 2.4 Hz), 129.3, 128.6 (d, *J* = 7.1 Hz), 124.3 (q, *J* = 272.8 Hz), 116.3, 114.7 (q, *J* = 4.0 Hz), 109.6 (q, *J* = 3.8 Hz), 107.8 (d, *J* = 6.4 Hz), 51.5 (d, *J* = 33.3 Hz), 21.3.

IR (neat, cm^{-1}): ṽ: 3418, 2923, 1914, 1686, 1611, 1505, 1433, 1323, 1223, 1161, 1040, 949, 863, 775.

ESI-HRMS: mass spectrometry: m/z calcd for $C_{27}H_{24}NF_5Na$ $[M+Na]^+$ 480.17211, measured 480.17195.

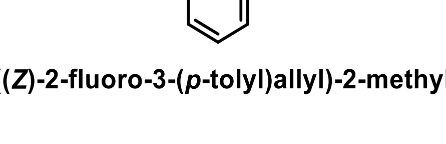

***N*,*N*-bis((*Z*)-2-fluoro-3-(*p*-tolyl)allyl)-2-methylaniline**

d18: The crude mixture was purified by SiO_2 gel column chromatography with pentane/EA (from 100:1). 66 mg product was obtained by 82% isolated yield as yellow oil.

^{1}H NMR (600 MHz, Chloroform-*d*) δ 7.27 (d, *J* = 7.8 Hz, 4H), 7.11 (d, *J* = 7.6 Hz, 1H), 7.07 – 7.02 (m, 6H), 6.96 – 6.90 (m, 1H), 5.51 (d, *J* = 38.9 Hz, 2H), 3.82 (d, *J* = 16.1 Hz, 4H), 2.31 (s, 3H), 2.24 (s, 6H).

^{19}F NMR (565 MHz, Chloroform-*d*) δ -106.80 (dt, *J* = 38.9 Hz, *J* = 16.2 Hz).

^{13}C NMR (151 MHz, Chloroform-*d*) δ 156.6 (d, *J* = 269.0 Hz), 149.1, 137.1 (d, *J* = 2.1 Hz), 134.4, 131.2, 130.3 (d, *J* = 2.3 Hz), 129.2, 128.5 (d, *J* = 7.3 Hz), 126.4, 124.5, 123.2, 108.9 (d, *J* = 6.8 Hz), 54.6 (d, *J* = 28.0 Hz), 21.3, 18.2.

IR (neat, cm^{-1}): ṽ: 3381, 2921, 1905, 1688, 1600, 1493, 1372, 1213, 1154, 1035, 939, 858, 766.

ESI-HRMS: mass spectrometry: m/z calcd for $C_{27}H_{28}NF_2$ $[M+H]^+$ 404.21843, measured 404.21841.

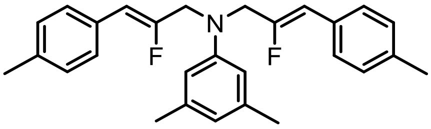

***N*,*N*-bis((*Z*)-2-fluoro-3-(*p*-tolyl)allyl)-3,5-dimethylaniline**

d19: The crude mixture was purified by SiO_2 gel column chromatography with pentane/EA (from 100:1). 67 mg product was obtained by 80% isolated yield as brown oil.

^{1}H NMR (600 MHz, Chloroform-*d*) δ 7.44 (d, *J* = 7.9 Hz, 4H), 7.19 (d, *J* = 7.9 Hz, 4H), 6.59 (s, 2H), 6.54 (s, 1H), 5.69 (d, *J* = 39.8 Hz, 2H), 4.25 (d, *J* = 7.2 Hz, 4H), 2.39 (s, 6H), 2.33 (s, 6H).

^{19}F NMR (565 MHz, Chloroform-*d*) δ -110.34 (dt, *J* = 39.9 Hz, *J* = 7.3 Hz).

^{13}C NMR (151 MHz, Chloroform-*d*) δ 155.5 (d, *J* = 269.4 Hz), 148.2, 139.0, 137.1 (d, *J* = 2.1 Hz), 130.1 (d, *J* = 2.2 Hz), 129.2, 128.5 (d, *J* = 7.2 Hz), 120.3, 111.2, 106.8 (d, *J* = 6.1 Hz), 51.3 (d, *J* = 34.6 Hz), 21.8, 21.3.

IR (neat, cm^{-1}): ṽ: 3414, 2019, 1904, 1691, 1597, 1511, 1372, 1185, 1129, 1036, 954, 906, 730.

ESI-HRMS: mass spectrometry: m/z calcd for $C_{28}H_{29}NF_2Na$ $[M+Na]^+$ 440.21603, measured 440.21506.

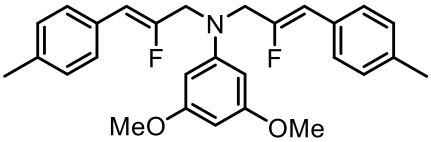

***N*,*N*-bis((*Z*)-2-fluoro-3-(*p*-tolyl)allyl)-3,5-dimethoxyaniline**

d20: The crude mixture was purified by SiO_2 gel column chromatography with pentane/EA (from 60:1). 85 mg product was obtained by 95% isolated yield as brown solid.

^{1}H NMR (600 MHz, Chloroform-*d*) δ 7.29 (d, *J* = 8.0 Hz, 4H), 7.04 (d, *J* = 8.0 Hz, 4H), 6.01 (d, *J* = 2.0 Hz, 2H), 5.90 (s, 1H), 5.56 (d, *J* = 39.6 Hz, 2H), 4.11 (d, *J* = 8.0 Hz, 4H), 3.67 (s, 6H), 2.25 (s, 6H).

^{19}F NMR (565 MHz, Chloroform-*d*) δ -110.49 (dt, *J* = 39.8 Hz, *J* = 8.1 Hz).

^{13}C NMR (151 MHz, Chloroform-*d*) δ 161.7, 155.2 (d, *J* = 269.0 Hz), 145.0, 137.2 (d, *J* = 2.2 Hz), 123.0 (d, *J* = 2.3 Hz), 129.2, 128.5 (d, *J* = 7.0 Hz), 107.2 (d, *J* = 6.2 Hz), 92.7, 90.2, 55.2, 51.5 (d, *J* = 34.2 Hz), 21.3.

IR (neat, cm^{-1}): ṽ: 3005, 2929, 1911, 1693, 1593, 1485, 1370, 1293, 1198, 1070, 969, 903, 804.

ESI-HRMS: mass spectrometry: m/z calcd for $C_{28}H_{29}NF_2Na$ $[M+Na]^+$ 472.20586, measured 420.20497.

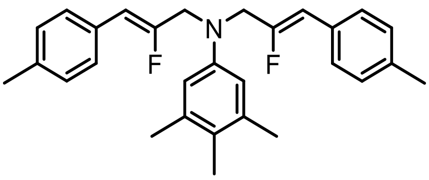

N,N-bis((Z)-2-fluoro-3-(p-tolyl)allyl)-3,4,5-trimethylaniline

d21: The crude mixture was purified by SiO_2 gel column chromatography with pentane/EA (from 100:1). 75 mg product was obtained by 87% isolated yield as brown oil.

^{1}H NMR (600 MHz, Chloroform-d) δ 7.29 (d, J = 7.8 Hz, 4H), 7.03 (d, J = 7.8 Hz, 4H), 6.50 (s, 2H), 5.55 (d, J = 39.9 Hz, 2H), 4.08 (d, J = 7.2 Hz, 4H), 2.24 (s, 6H), 2.16 (s, 6H), 2.00 (s, 3H).

^{19}F NMR (565 MHz, Chloroform-d) δ -110.13 (dt, J = 39.7 Hz, J = 7.6 Hz).

^{13}C NMR (151 MHz, Chloroform-d) δ 155.8 (d, J = 269.5 Hz), 145.7, 137.4, 137.1 (d, J = 2.0 Hz), 130.2 (d, J = 2.2 Hz), 129.2, 128.6 (d, J = 7.1 Hz), 125.0, 112.9, 106.8 (d, J = 6.2 Hz), 51.4 (d, J = 34.4 Hz), 21.3, 21.2, 14.5.

IR (neat, cm^{-1}): ṽ: 2918, 1906, 1688, 1605, 1496, 1366, 1222, 1179, 1153, 999, 906, 861, 729.

ESI-HRMS: mass spectrometry: m/z calcd for $C_{29}H_{32}NF_2$ $[M+H]^+$ 432.24973, measured 432.24983.

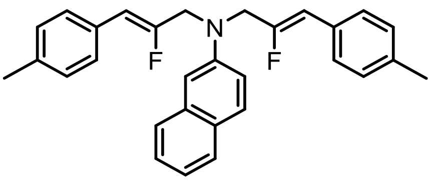

N,N-bis((Z)-2-fluoro-3-(p-tolyl)allyl)naphthalen-2-amine

d22: The crude mixture was purified by SiO_2 gel column chromatography with pentane/EA (from 100:1). 62 mg product was obtained by 71% isolated yield as brown oil.

^{1}H NMR (600 MHz, Chloroform-d) δ 7.65 (d, J = 9.0 Hz, 1H), 7.62 (d, J = 8.1 Hz, 1H), 7.57 (d, J = 8.4 Hz, 1H), 7.29 (d+m, J = 8.1 Hz, 5H), 7.17 – 7.14 (m, 2H), 7.07 (d, J = 2.6 Hz, 1H), 7.04 (d, J = 7.9 Hz, 4H), 5.60 (d, J = 39.7 Hz, 2H), 4.24 (d, J = 7.9 Hz, 4H), 2.24 (s, 6H).

^{19}F NMR (565 MHz, Chloroform-d) δ -110.37 (dt, J = 39.9 Hz, J = 8.0 Hz).

^{13}C NMR (151 MHz, Chloroform-d) δ 155.3 (d, J = 269.2 Hz), 145.8, 137.2 (d, J = 2.2 Hz), 134.7, 130.0 (d, J = 2.3 Hz), 129.22, 129.16, 128.6 (d, J = 7.1 Hz), 127.6, 127.4, 126.48, 126.46, 122.8, 116.1, 107.7, 107.3 (d, J = 6.2 Hz), 51.6 (d, J = 34.1 Hz), 21.3.

IR (neat, cm^{-1}): ṽ: 3857, 2922, 1908, 1691, 1628, 1510, 1382, 1213, 1184, 1129, 1040, 955, 831.

ESI-HRMS: mass spectrometry: m/z calcd for $C_{30}H_{27}NF_2Na$ $[M+Na]^+$ 462.20038, measured 462.19924.

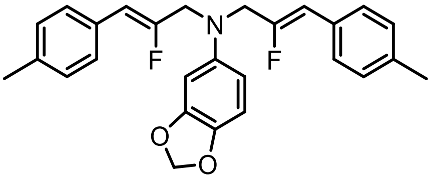

N,N-bis((Z)-2-fluoro-3-(p-tolyl)allyl)benzo[d][1,3]dioxol-5-amine

d23: The crude mixture was purified by SiO_2 gel column chromatography with pentane/EA (from 60:1). 45 mg product was obtained by 52% isolated yield as brown solid.

^{1}H NMR (600 MHz, Chloroform-*d*) δ 7.29 (d, *J* = 7.9 Hz, 4H), 7.05 (d, *J* = 7.9 Hz, 4H), 6.62 (d, *J* = 8.5 Hz, 1H), 6.47 (d, *J* = 2.5 Hz, 1H), 6.26 (dd, *J* = 8.6, 2.5 Hz, 1H), 5.79 (s, 2H), 5.55 (d, *J* = 39.7 Hz, 2H), 4.05 (d, *J* = 9.0 Hz, 4H), 2.25 (s, 6H).
^{19}F NMR (565 MHz, Chloroform-*d*) δ -110.08 (dt, *J* = 39.5 Hz, *J* = 9.0 Hz).
^{13}C NMR (151 MHz, Chloroform-*d*) δ 155.5 (d, *J* = 269.3 Hz), 148.5, 144.1, 140.4, 137.2 (d, *J* = 2.1 Hz), 130.0 (d, *J* = 2.3 Hz), 129.2, 128.5 (d, *J* = 7.1 Hz), 108.5, 107.4 (d, *J* = 6.1 Hz), 106.3, 100.9, 97.3, 52.5 (d, *J* = 33.2 Hz), 21.3.
IR (neat, cm^{-1}): ṽ: 2921, 1911, 1695, 1631, 1502, 1431, 1371, 1274, 1204, 1127, 1034, 965, 807, 731.
ESI-HRMS: mass spectrometry: m/z calcd for $C_{27}H_{26}O_2NF_2$ $[M+H]^+$ 434.19261, measured 434.19126.

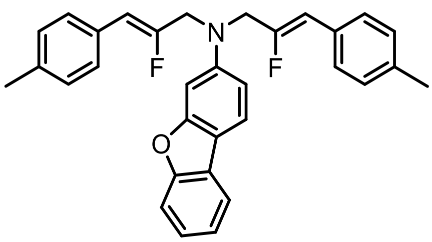

***N*,*N*-bis((*Z*)-2-fluoro-3-(*p*-tolyl)allyl)dibenzo[*b*,*d*]furan-3-amine**

d24: The crude mixture was purified by SiO_2 gel column chromatography with pentane/EA (from 60:1). 72 mg product was obtained by 75% isolated yield as brown solid.
^{1}H NMR (600 MHz, Chloroform-*d*) δ 7.72 (d, *J* = 8.0 Hz, 1H), 7.69 (d, *J* = 8.6 Hz, 1H), 7.39 (d, *J* = 8.1 Hz, 1H), 7.31 (d, *J* = 8.0 Hz, 4H), 7.24 (t, *J* = 7.6 Hz, 1H), 7.19 (t, *J* = 7.5 Hz, 1H), 7.05 (d, *J* = 8.0 Hz, 4H), 7.01 (d, *J* = 2.3 Hz, 1H), 6.86 (dd, *J* = 8.6, 2.3 Hz, 1H), 5.61 (d, *J* = 39.6 Hz, 2H), 4.24 (d, *J* = 8.3 Hz, 4H), 2.25 (s, 6H).
^{19}F NMR (565 MHz, Chloroform-*d*) δ -110.64 (dt, *J* = 39.8 Hz, *J* = 8.2 Hz).
^{13}C NMR (151 MHz, Chloroform-*d*) δ 158.1, 156.1, 155.0 (d, *J* = 269.2 Hz), 148.4, 137.3 (d, *J* = 2.1 Hz), 129.8 (d, *J* = 2.4 Hz), 129.2, 128.6 (d, *J* = 7.1 Hz), 125.3, 124.6, 122.6, 121.1, 119.5, 115.3, 111.2, 109.5, 107.5 (d, *J* = 6.3 Hz), 96.2, 52.0 (d, *J* = 33.9 Hz), 21.2.
IR (neat, cm^{-1}): ṽ: 3021, 2920, 1916, 1690, 1601, 1503, 1456, 1377, 1239, 1156, 1010, 943, 811, 721.
ESI-HRMS: mass spectrometry: m/z calcd for $C_{32}H_{28}ONF_2$ $[M+H]^+$ 480.21335, measured 480.21187.

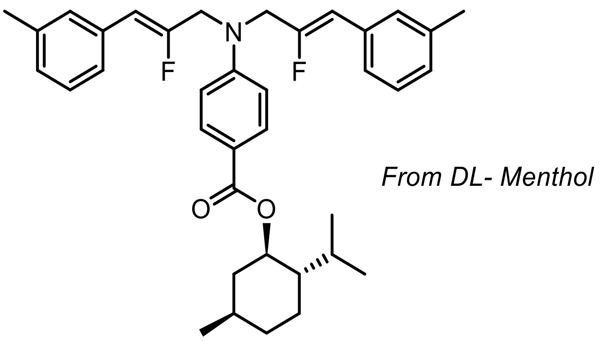

(1*R*,2*S*,5*R*)-2-isopropyl-5-methylcyclohexyl 4-(bis((*Z*)-2-fluoro-3-(*m*-tolyl)allyl)amino)benzoate

d25: The crude mixture was purified by SiO_2 gel column chromatography with pentane/EA (from 50:1). 86 mg product was obtained by 75% isolated yield as yellow oil.

^{1}H NMR (600 MHz, Chloroform-*d*) δ 7.87 (d, *J* = 8.7 Hz, 2H), 7.20 (d+m, *J* = 8.5 Hz, 4H), 7.13 (t, *J* = 7.6 Hz, 2H), 6.97 (d, *J* = 7.5 Hz, 2H), 6.80 (d, *J* = 8.7 Hz, 2H), 5.54 (d, *J* = 39.3 Hz, 2H), 4.80 (td, *J* = 10.9 Hz, *J* = 4.4 Hz, 1H), 4.19 (d, *J* = 7.9 Hz, 4H), 2.24 (s, 6H), 2.03 (dm, *J* = 12.2 Hz, 1H), 1.88 (sept.d, *J* = 7.0 Hz, *J* = 2.6 Hz, 1H), 1.66 – 1.58 (m, 2H), 1.48 – 1.40 (m, 2H), 1.06 – 0.93 (m, 2H), 0.84 – 0.80 (m, 7H), 0.70 (d, *J* = 6.9 Hz, 3H).
^{19}F NMR (565 MHz, Chloroform-*d*) δ -110.12 (dt, *J* = 39.3 Hz, *J* = 8.2 Hz).
^{13}C NMR (151 MHz, Chloroform-*d*) δ 166.1, 154.8 (d, *J* = 269.8 Hz), 151.1, 138.1, 132.4 (d, *J* = 2.3 Hz), 131.4, 129.4 (d, *J* = 6.7 Hz), 128.5, 128.4 (d, *J* = 1.6 Hz), 125.8 (d, *J* = 7.2 Hz), 120.3, 112.0, 107.8 (d, *J* = 5.9 Hz), 74.1, 51.4 (d, *J* = 34.1 Hz), 47.4, 41.2, 34.4, 31.5, 26.5, 23.8, 22.1, 21.4, 20.8, 16.6.
IR (neat, cm^{-1}): ṽ: 3380, 2926, 1937, 1693, 1604, 1519, 1453, 1378, 1279, 1185, 1115, 1039, 961, 732.
ESI-HRMS: mass spectrometry: m/z calcd for $C_{37}H_{43}O_2NF_2Na$ $[M+Na]^+$ 594.31541, measured 594.31439.

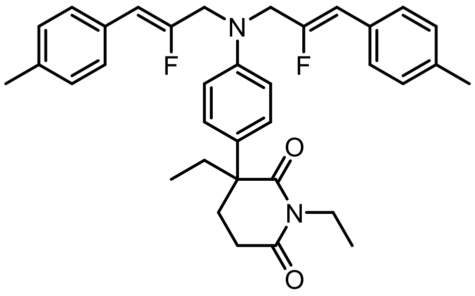

3-(4-(bis((*Z*)-2-fluoro-3-(*p*-tolyl)allyl)amino)phenyl)-1,3-diethylpiperidine-2,6-dione

d26: The crude mixture was purified by SiO_2 gel column chromatography with pentane/EA (from 10:1). 101 mg product was obtained by 90% isolated yield as brown oil.
^{1}H NMR (600 MHz, Chloroform-*d*) δ 7.29 (d, *J* = 7.9 Hz, 4H), 7.04 (d, *J* = 7.8 Hz, 4H), 6.98 (d, *J* = 8.6 Hz, 2H), 6.76 (d, *J* = 8.5 Hz, 2H), 5.54 (d, *J* = 39.6 Hz, 2H), 4.11 (d, *J* = 8.2 Hz, 4H), 3.86 – 3.70 (m, 2H), 2.51 (dm, *J* = 17.2 Hz, 1H), 2.44 – 2.36 (m, 1H), 2.24 (s, 6H), 2.14 (dm, *J* = 13.4 Hz, 1H), 2.02 (td, *J* = 13.8 Hz, *J* = 4.7 Hz, 1H), 1.98 – 1.90 (m, 1H), 1.80 – 1.71 (m, 1H), 1.05 (t, *J* = 7.1 Hz, 3H), 0.76 (t, *J* = 7.4 Hz, 3H).
^{19}F NMR (565 MHz, Chloroform-*d*) δ -110.59 (dt, *J* = 39.6 Hz, *J* = 8.6 Hz).
^{13}C NMR (151 MHz, Chloroform-*d*) δ 175.3, 172.2, 155.1 (d, *J* = 269.1 Hz), 147.0, 137.3 (d, *J* = 1.7 Hz), 129.9 (d, *J* = 2.2 Hz), 129.2, 128.7, 128.6 (d, *J* = 6.9 Hz), 127.2, 113.3, 107.3 (d, *J* = 6.3 Hz), 51.4 (d, *J* = 33.9 Hz), 50.5, 35.3, 33.8, 30.0, 25.9, 21.3, 13.3, 9.2.
IR (neat, cm^{-1}): ṽ: 3374, 2972, 1904, 1669, 1516, 1453, 1357, 1212, 1123, 1045, 908, 862, 809, 730.
ESI-HRMS: mass spectrometry: m/z calcd for $C_{35}H_{38}O_2N_2F_2Na$ $[M+Na]^+$ 579.27936, measured 579.27783.

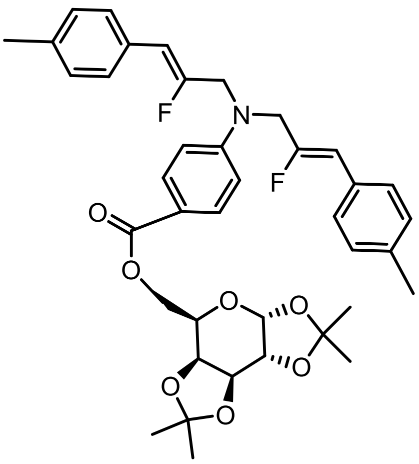

((3a*R*,5*R*,5a*S*,8a*S*,8b*R*)-2,2,7,7-tetramethyltetrahydro-5*H*-bis([1,3]dioxolo)[4,5-*b*:4',5'-*d*]pyran-5-yl)methyl 4-(bis((*Z*)-2-fluoro-3-(*p*-tolyl)allyl)amino)benzoate

d27: The crude mixture was purified by SiO_2 gel column chromatography with pentane/EA (from 5:1). 111 mg product was obtained by 82% isolated yield as brown oil.

^{1}H NMR (600 MHz, Chloroform-*d*) δ 7.87 (d, *J* = 8.6 Hz, 2H), 7.28 (d, *J* = 7.9 Hz, 4H), 7.04 (d, *J* = 7.9 Hz, 4H), 6.79 (d, *J* = 8.7 Hz, 2H), 5.53 (d, *J* = 39.4 Hz, 2H), 5.47 (d, *J* = 4.9 Hz, 1H), 4.55 (dd, *J* = 7.9, 2.4 Hz, 1H), 4.40 (dd, *J* = 11.4 Hz, *J* = 5.1 Hz, 1H), 4.30 (dd, *J* = 11.4 Hz, *J* = 7.3 Hz, 1H), 4.27 – 4.21 (m, 2H), 4.18 (d, *J* = 8.2 Hz, 4H), 4.10 – 4.05 (m, 1H), 2.24 (s, 6H), 1.43 (s, 3H), 1.39 (s, 3H), 1.27 (s, 3H), 1.24 (s, 3H).

^{19}F NMR (565 MHz, Chloroform-*d*) δ -111.04 (dt, *J* = 39.4 Hz, *J* = 8.4 Hz).

^{13}C NMR (151 MHz, Chloroform-*d*) δ 166.3, 154.3 (d, *J* = 268.4 Hz), 151.4, 137.4 (d, *J* = 1.7 Hz), 131.6, 129.6 (d, *J* = 2.2 Hz), 129.3, 128.6 (d, *J* = 7.0 Hz), 119.3, 112.0, 109.6, 108.8, 107.6 (d, *J* = 6.2 Hz), 96.4, 71.2, 70.8, 70.6, 66.2, 63.3, 51.2 (d, *J* = 34.1 Hz), 26.1, 26.0, 25.0, 24.5, 21.3.

IR (neat, cm^{-1}): ṽ: 2986, 1908, 1703, 1604, 1518, 1378, 1323, 1278, 1185, 1105, 1069, 906, 767.

ESI-HRMS: mass spectrometry: m/z calcd for $C_{39}H_{43}O_7N_1F_2Na$ $[M+Na]^+$ 698.28998, measured 698.28961.

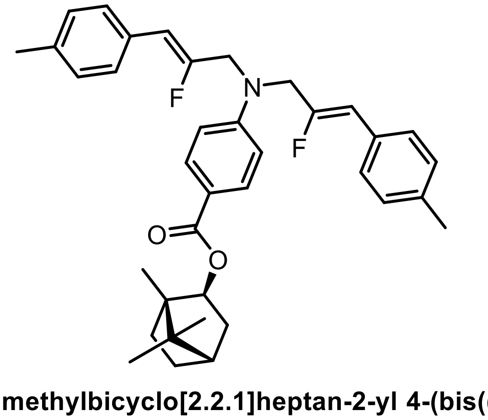

From DL-Isoborneol

(1*S*,2*S*,4*S*)-1,7,7-trimethylbicyclo[2.2.1]heptan-2-yl 4-(bis((*Z*)-2-fluoro-3-(*p*-tolyl)allyl)amino)benzoate

d28: The crude mixture was purified by SiO_2 gel column chromatography with pentane/EA (from 10:1). 95 mg product was obtained by 83% isolated yield as brown oil.

^{1}H NMR (600 MHz, Chloroform-*d*) δ 7.89 (d, *J* = 8.5 Hz, 2H), 7.28 (d, *J* = 7.9 Hz, 4H), 7.05 (d, *J* = 7.8 Hz, 4H), 6.82 (d, *J* = 8.6 Hz, 2H), 5.54 (d, *J* = 39.5 Hz, 2H), 4.99 (d, *J* = 9.4 Hz, 1H), 4.19 (d, *J* = 8.1

Hz, 4H), 2.40 – 2.33 (m, 1H), 2.25 (s, 6H), 2.09 – 2.01 (m, 1H), 1.74 – 1.65 (m, 1H), 1.63 (t, *J* = 4.6 Hz, 1H), 1.33 – 1.26 (m, 1H), 1.24 – 1.15 (m, 1H), 1.01 (dd, *J* = 13.8, 3.5 Hz, 1H), 0.87 (s, 3H), 0.82 (s, 3H), 0.81 (s, 3H).

^{19}F NMR (565 MHz, Chloroform-*d*) δ -111.07 (dt, *J* = 39.6 Hz, *J* = 8.4 Hz).

^{13}C NMR (151 MHz, Chloroform-*d*) δ 166.8, 154.4 (d, *J* = 268.7 Hz), 151.2, 137.4 (d, *J* = 2.1 Hz), 131.4, 129.7 (d, *J* = 2.2 Hz), 129.3, 128.6 (d, *J* = 7.0 Hz), 120.2, 112.0, 107.6 (d, *J* = 6.2 Hz), 79.8, 51.3 (d, *J* = 34.1 Hz), 49.1, 47.9, 45.1, 37.0, 28.1, 27.4, 21.3, 19.8, 19.0, 13.6.

IR (neat, cm^{-1}): ṽ: 2954, 1907, 1696, 1604, 1517, 1450, 1377, 1281, 1224, 1185, 1117, 908, 834, 731.

ESI-HRMS: mass spectrometry: m/z calcd for $C_{37}H_{42}O_2N_1F_2$ [M+H]$^+$ 570.31781, measured 570.31685.

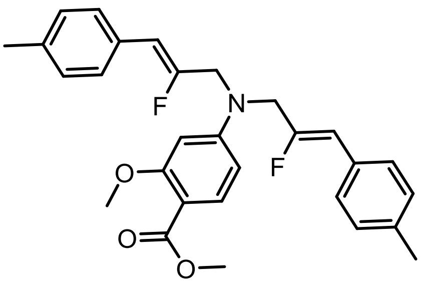

methyl 4-(bis((*Z*)-2-fluoro-3-(*p*-tolyl)allyl)amino)-2-methoxybenzoate

d29: The crude mixture was purified by SiO_2 gel column chromatography with pentane/EA (from 10:1). 78 mg product was obtained by 82% isolated yield as brown oil.

^{1}H NMR (600 MHz, Chloroform-*d*) δ 7.31 – 7.26 (m, 5H), 7.04 (d, *J* = 7.9 Hz, 4H), 6.94 (dd, *J* = 9.1, 3.3 Hz, 1H), 6.81 (d, *J* = 9.1 Hz, 1H), 5.55 (d, *J* = 39.5 Hz, 2H), 4.08 (d, *J* = 9.7 Hz, 4H), 3.79 (s, 3H), 3.74 (s, 3H), 2.24 (s, 6H).

^{19}F NMR (565 MHz, Chloroform-*d*) δ -110.11 (dt, *J* = 39.8 Hz, *J* = 9.9 Hz).

^{13}C NMR (151 MHz, Chloroform-*d*) δ 166.9, 155.3 (d, *J* = 269.0 Hz), 152.2, 141.9, 137.3 (d, *J* = 2.0 Hz), 129.9 (d, *J* = 2.3 Hz), 129.2, 128.5 (d, *J* = 7.1 Hz), 120.7, 119.3, 117.1, 114.0, 107.7 (d, *J* = 6.2 Hz), 56.8, 52.13, 52.10 (d, *J* = 32.8 Hz), 21.3.

IR (neat, cm^{-1}): ṽ: 2948, 1909, 1724, 1613, 1504, 1436, 1294, 1242, 1182, 1082, 1022, 861, 807.

ESI-HRMS: mass spectrometry: m/z calcd for $C_{29}H_{30}O_3N_1F_2$ [M+H]$^+$ 478.21883, measured 478.21875.

8. Transformation of products

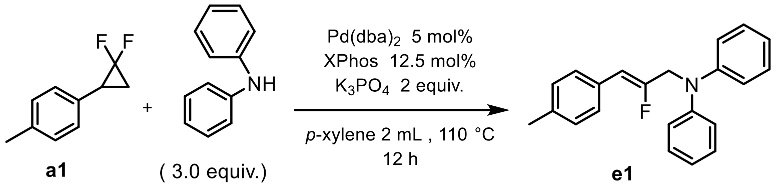

Under N_2 atmosphere, XPhos (11.9 mg, 0.025 mmol) and Pd(dba)$_2$ (5.7 mg, 0.01 mmol), gem-difluorocyclopropane **a1** (0.2 mmol), diphenylamine (0.6 mmol), K_3PO_4 (2.0 equiv., 84.8 mg) were dissolved in 2 mL *p*-xylene, then the mixture was stirred at 110 °C for about 12 h to the starting material was consumed (monitored by TLC), the mixture was filtered through celite and the filtrate was concentrated to dryness. The crude was purified by column chromatography to give the product **e1** (95% yield, 60 mg).

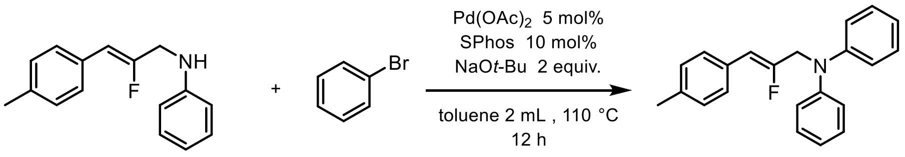

c1 (1.2 equiv.) **e1**

Under N_2 atmosphere, SPhos (8.2 mg, 0.02 mmol) and $Pd(OAc)_2$ (2.2 mg, 0.01 mmol), product **c1** (0.2 mmol), bromobenzene (0.24 mmol), NaO*t*-Bu (2.0 equiv., 38.4 mg) were dissolved in 2 mL toluene, then the mixture was stirred at 110 °C for about 12 h to the starting material was consumed (monitored by TLC), the mixture was filtered through celite and the filtrate was concentrated to dryness. The crude was purified by column chromatography to give the product **e1** (95% yield, 60 mg).

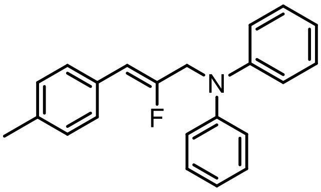

(*Z*)-*N*-(2-fluoro-3-(*p*-tolyl)allyl)-*N*-phenylaniline

e1: The crude mixture was purified by SiO_2 gel column chromatography with pentane/EA (from 100:1). 60 mg product was obtained by 95% isolated yield as yellow oil.

^{1}H NMR (600 MHz, Chloroform-*d*) δ 7.25 (d, *J* = 8.0 Hz, 2H), 7.19 (≈ t, *J* ≈ 7.9 Hz, 4H), 7.03 – 6.99 (m, 6H), 6.90 (t, *J* = 7.3 Hz, 2H), 5.61 (d, *J* = 39.9 Hz, 1H), 4.46 (d, *J* = 6.9 Hz, 2H), 2.23 (s, 3H).

^{19}F NMR (565 MHz, Chloroform-*d*) δ -109.93 (dt, *J* = 40.1, 7.0 Hz).

^{13}C NMR (151 MHz, Chloroform-*d*) δ 155.7 (d, *J* = 269.0 Hz), 147.5, 137.0 (d, *J* = 2.3 Hz), 130.2 (d, *J* = 2.3 Hz), 129.4, 129.1, 128.5 (d, *J* = 7.1 Hz), 121.9, 120.9, 107.1 (d, *J* = 6.2 Hz), 53.4 (d, *J* = 35.0 Hz), 21.2.

IR (neat, cm^{-1}): ṽ: 3398, 2920, 2325, 2904, 1693, 1588, 1359, 1216, 1134, 1096, 994, 864, 746.

EI-HRMS: mass spectrometry: m/z calcd for $C_{22}H_{20}NF$ $[M]^+$ 317.15743, measured 317.15730.

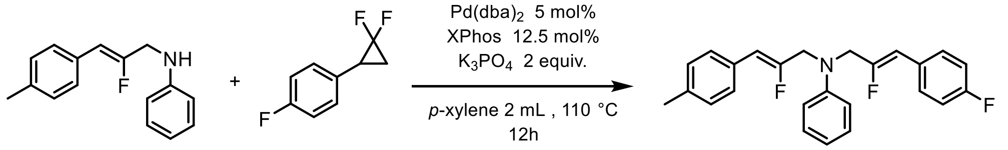

c1 (2.0 equiv.) **d30**

Under N_2 atmosphere, XPhos (11.9 mg, 0.025 mmol) and $Pd(dba)_2$ (5.7 mg, 0.01 mmol), product **c1** (0.2 mmol), 1-(2,2-difluorocyclopropyl)-4-fluorobenzene (0.4 mmol), K_3PO_4 (2.0 equiv., 84.8 mg) were dissolved in 2 mL *p*-xylene, then the mixture was stirred at 110 °C for about 12 h to the starting material was consumed (monitored by TLC), the mixture was filtered through celite and the filtrate was concentrated to dryness. A portion of the residue was analyzed with ^{1}H NMR to determine selectivity and recovered. The crude was purified by column chromatography to give the products **d30** (64% yield, 50 mg).

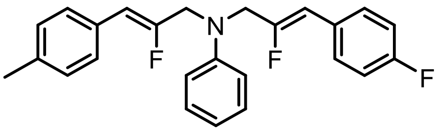

N-((Z)-2-fluoro-3-(4-fluorophenyl)allyl)-N-((Z)-2-fluoro-3-(p-tolyl)allyl)aniline

d30: The crude mixture was purified by SiO_2 gel column chromatography with pentane/EA (from 100:1). 50 mg product was obtained by 64% isolated yield as brown oil.

^{1}H NMR (600 MHz, Chloroform-*d*) δ 7.38 – 7.33 (m, 2H), 7.29 (d, *J* = 8.1 Hz, 2H), 7.18 (≈ dd, *J* = 8.8, 7.2 Hz, 2H), 7.04 (d, *J* = 7.9 Hz, 2H), 6.92 (≈ t, *J* = 8.7 Hz, 2H), 6.82 (d, *J* = 7.8 Hz, 2H), 6.73 (t, *J* = 7.3 Hz, 1H), 5.56 (d, *J* = 39.4 Hz, 2H), 4.16 – 4.12 (m, 4H), 2.25 (s, 3H).

^{19}F NMR (565 MHz, Chloroform-*d*) δ -110.52 (dt, *J* = 39.5, 8.1 Hz, 1F), -110.75 (dt, *J* = 39.2, 7.4 Hz, 1F), -114.00 (m, 1F).

^{13}C NMR (151 MHz, Chloroform-*d*) δ 161.8 (dd, *J* = 247.3, 3.2 Hz), 155.6 (dd, *J* = 269.6, 2.5 Hz), 155.2 (d, *J* = 269.3 Hz), 147.8, 137.2 (d, *J* = 2.2 Hz), 130.3 (t, *J* = 7.6 Hz), 129.9 (d, *J* = 2.4 Hz), 129.4, 129.2, 129.0 (t, *J* = 2.9 Hz), 128.5 (d, *J* = 6.9 Hz), 118.3, 115.4 (d, *J* = 21.4 Hz), 113.2, 107.2 (d, *J* = 6.1 Hz), 106.0 (d, *J* = 6.1 Hz), 51.6 (d, *J* = 22.7 Hz), 51.4 (d, *J* = 23.0 Hz), 21.3.

IR (neat, cm^{-1}): ṽ: 3389, 2922, 1896, 1692, 1598, 1504, 1378, 1224, 1129, 1023, 955, 859, 745.

EI-HRMS: mass spectrometry: m/z calcd for $C_{25}H_{22}NF_3$ $[M]^+$ 393.16989, measured 393.16976.

9. Kinetic orders

9.1. Kinetic order in *gem*-difluorocyclopropane **a1**:

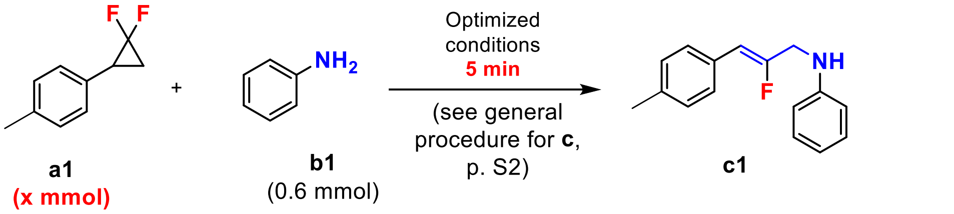

a1	c1
0.1 mmol	0,036 mmol
0,2 mmol	0,058 mmol
0,4 mmol	0,144 mmol
0,8 mmol	0,144 mmol

(determined by ^{1}H NMR analysis, 1,3,5-trimethoxybenzene as an internal standard)

X-axis : Ln(**a1**), Y-axis : Ln(**c1**)

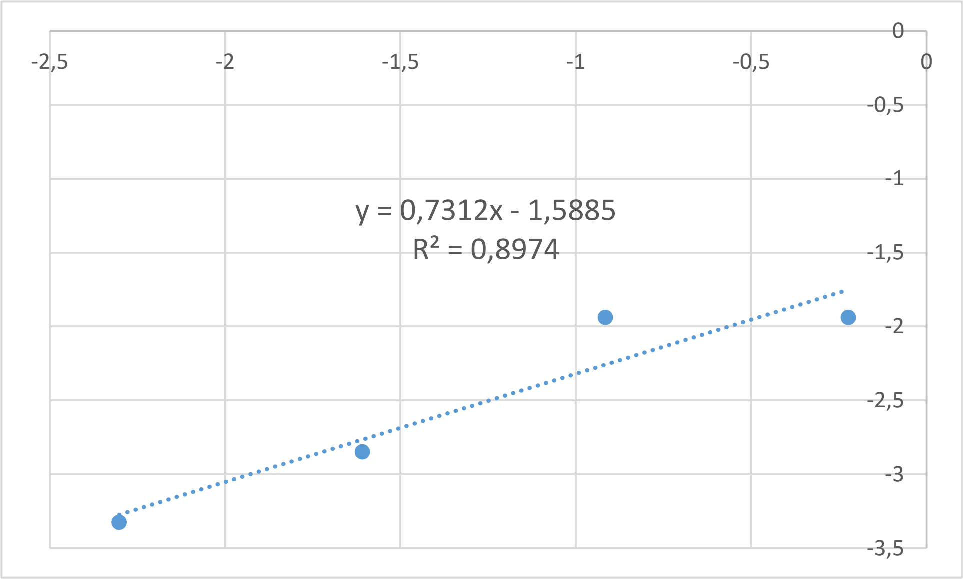

X-axis : Ln(**a1**), Y-axis : Ln(**c1**), first 3 points only.

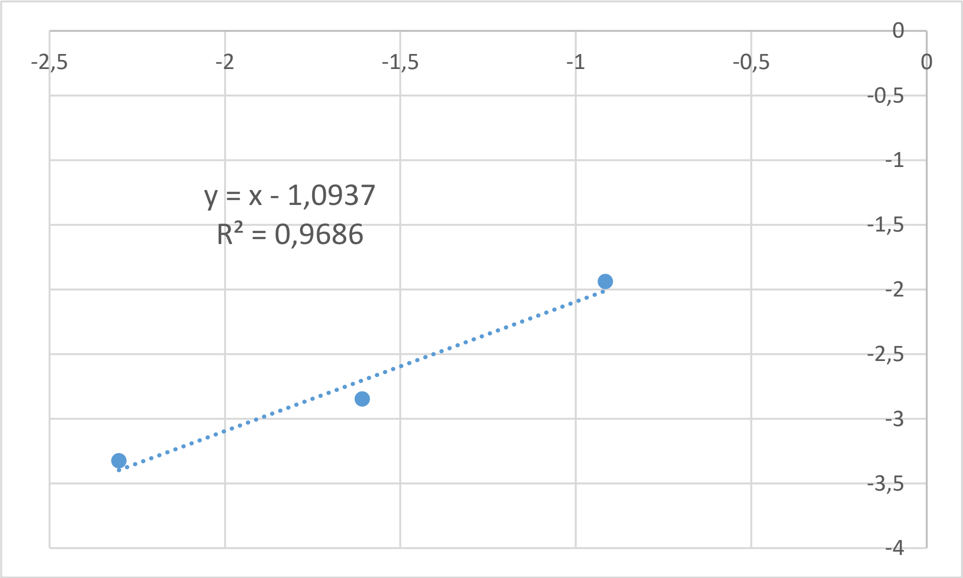

Result: the reaction is first order in *gem*-difluorocyclopropane **a1**.

9.2. Kinetic order in aniline **b1**:

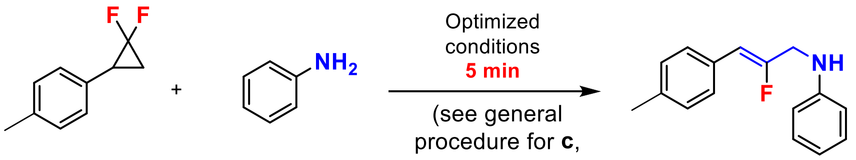

a1 (0.2 mmol) + **b1** (**x mmol**) → **c1**

b1	c1
**0.1 mmol**	**0,064 mmol**
**0,2 mmol**	**0,074 mmol**
**0,4 mmol**	**0,076 mmol**
**0,8 mmol**	**0,072 mmol**

(determined by ^{1}H NMR analysis, 1,3,5-trimethoxybenzene as an internal standard)

Result: the reaction is approximatively zeroth order in aniline **b1**.

10. References

[1] J. Xu, E. A. Ahmed, B. Xiao, Q. Q. Lu, Y. L. Wang, C. G. Yu, Y. Fu, *Angew. Chem. Int. Ed.* **2015**, *54*, 8231.

[2] L. Y. Lv, C.-J. Li, *Angew. Chem. Int. Ed.* **2021**, *60*, 13098.

[3] S. Q. Chen, S. Cao, C. Y. Liu, B. X. Wang, X. R. Ren, H. Huang, Z. H. Peng, X.Wang, *Org. Lett.* **2021**, *23*, 7428.

[4] W. Liu, L. Cao, Z.-Y. Zhang, G. Zhang, S.-Q. Huang, L.-W. Huang, P. Zhao, X. Y. Yan, *Org. Lett.* **2020**, *22*, 2210.

11. Copies of ^{1}H and ^{13}C Spectra

^{1}H NMR (**c1**)

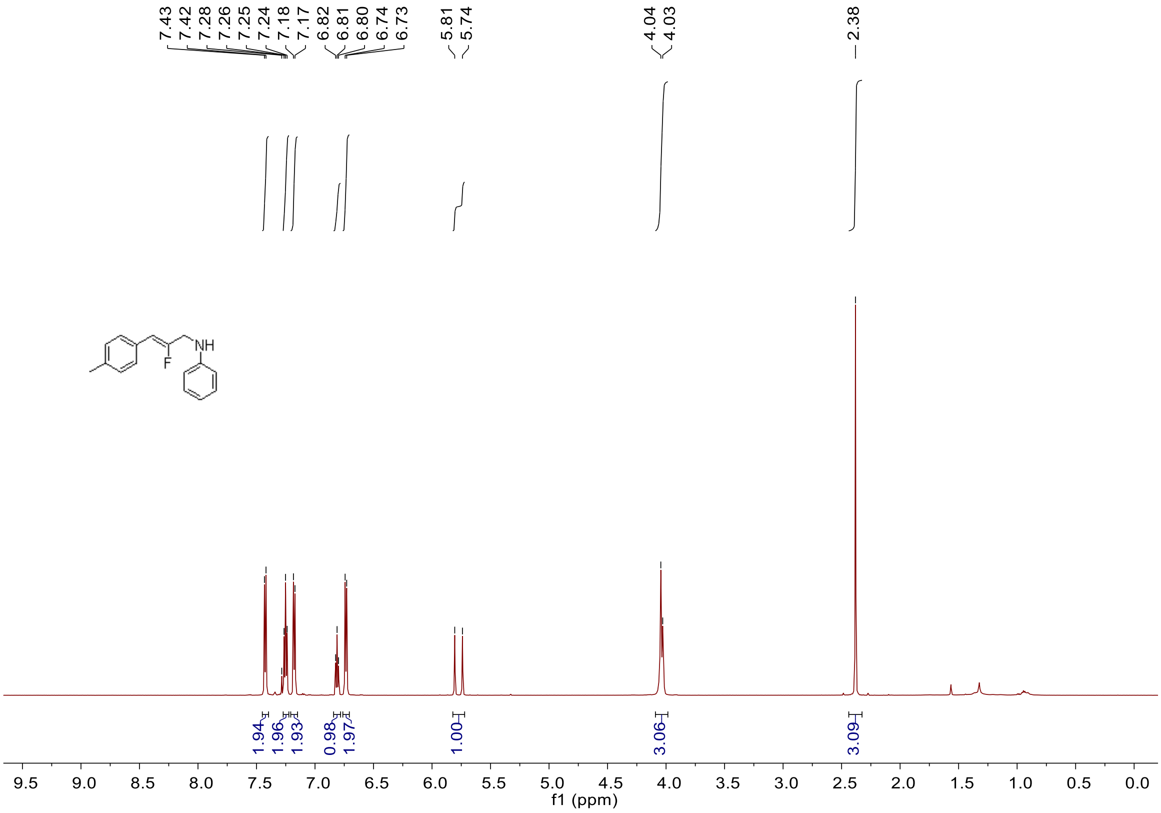

^{19}F NMR (**c1**)

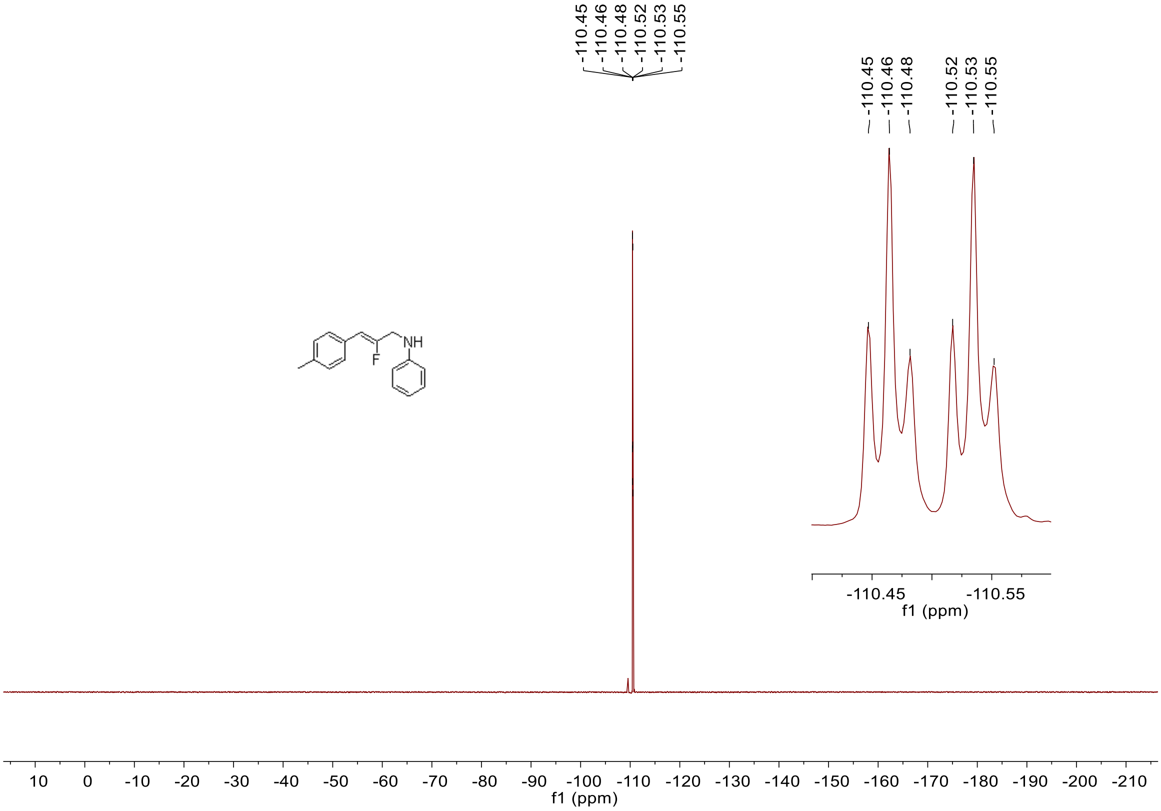

^{13}C NMR (**c1**)

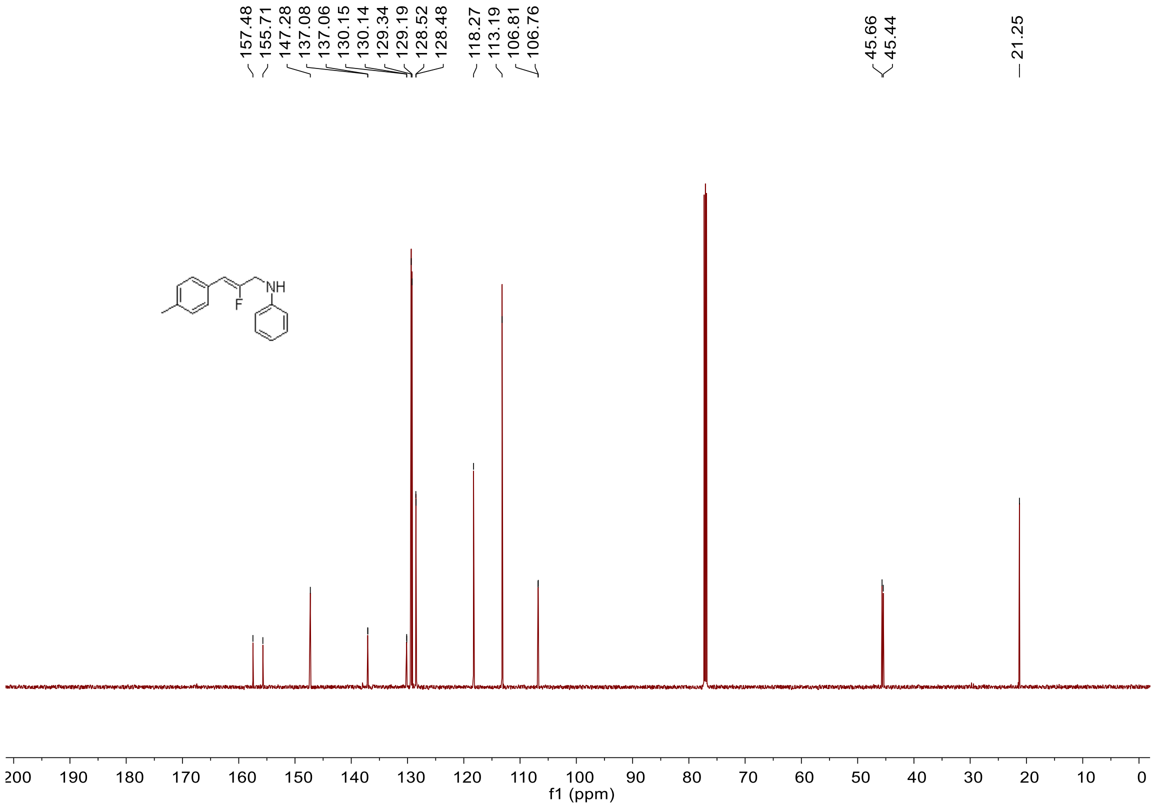

^{1}H NMR (**c2**)

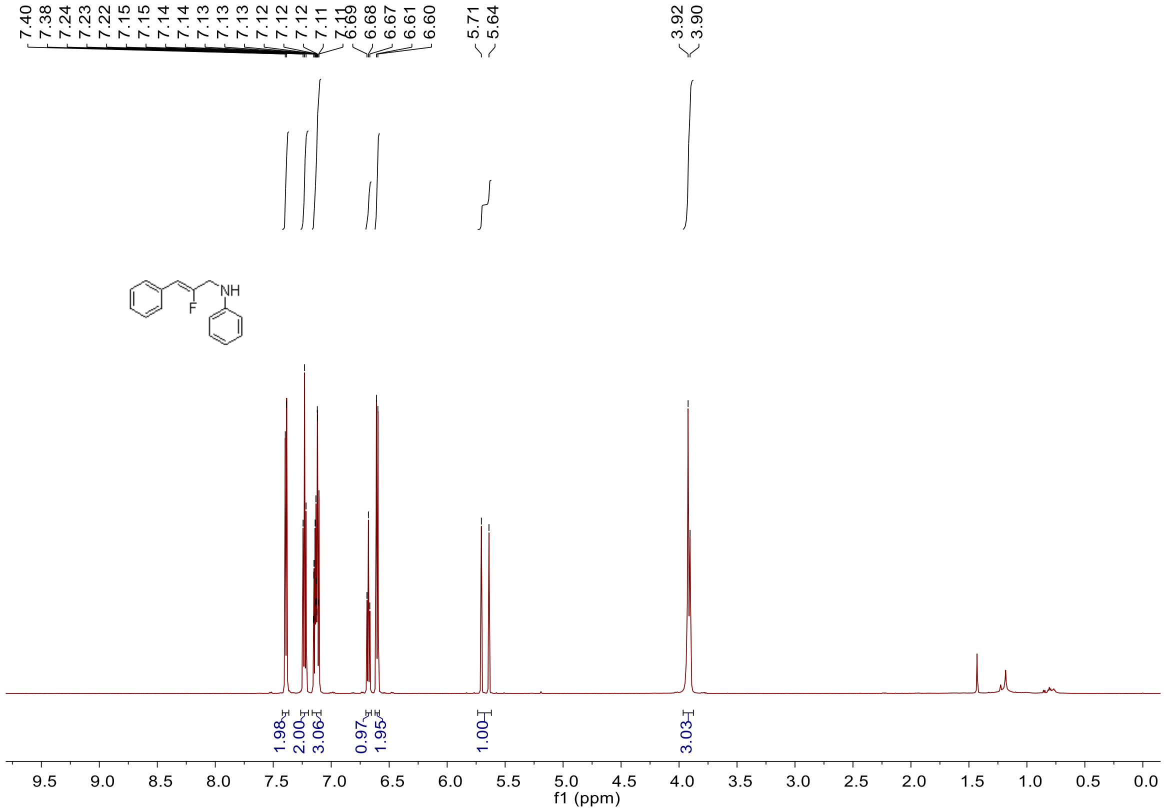

^{19}F NMR (**c2**)

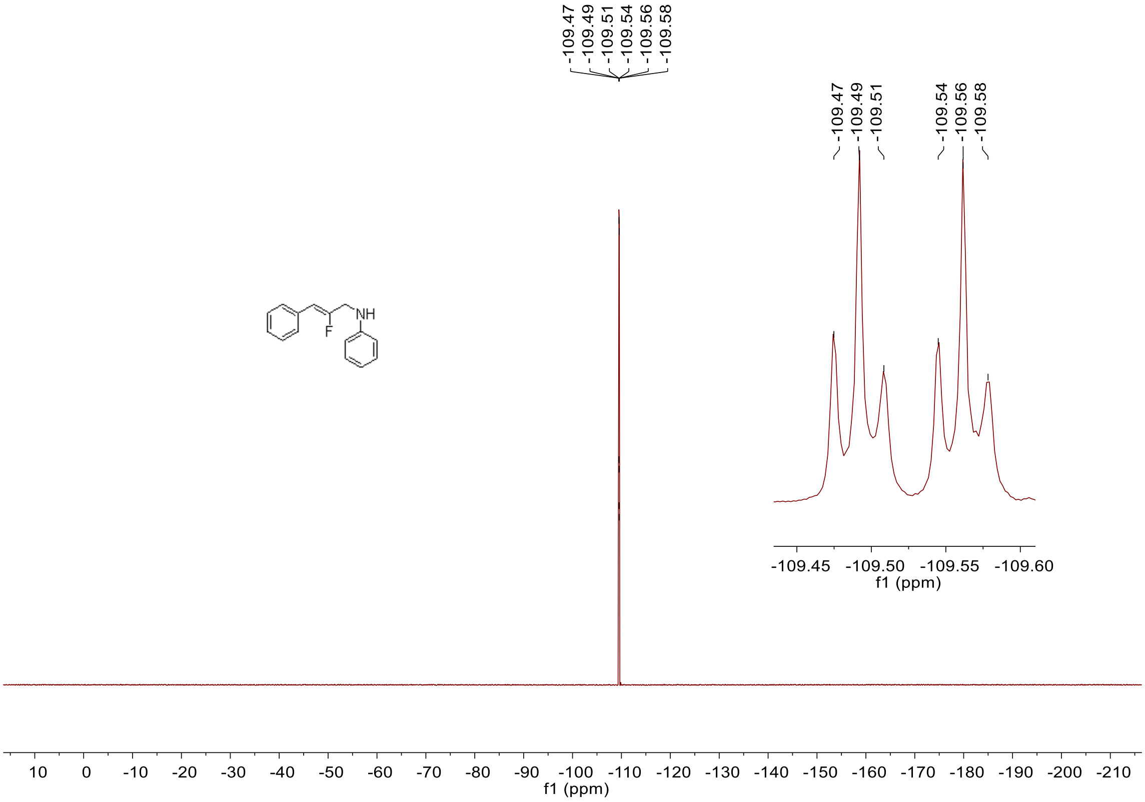

^{13}C NMR (**c2**)

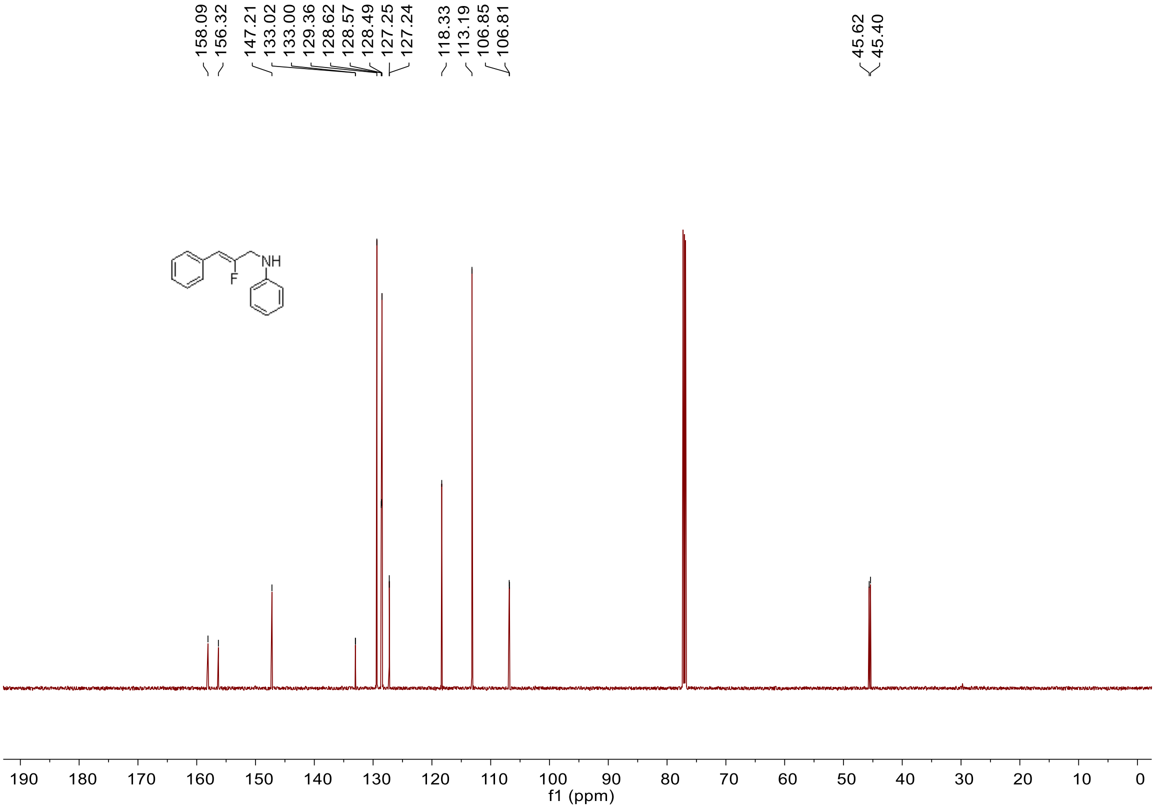

^{1}H NMR (**c3**)

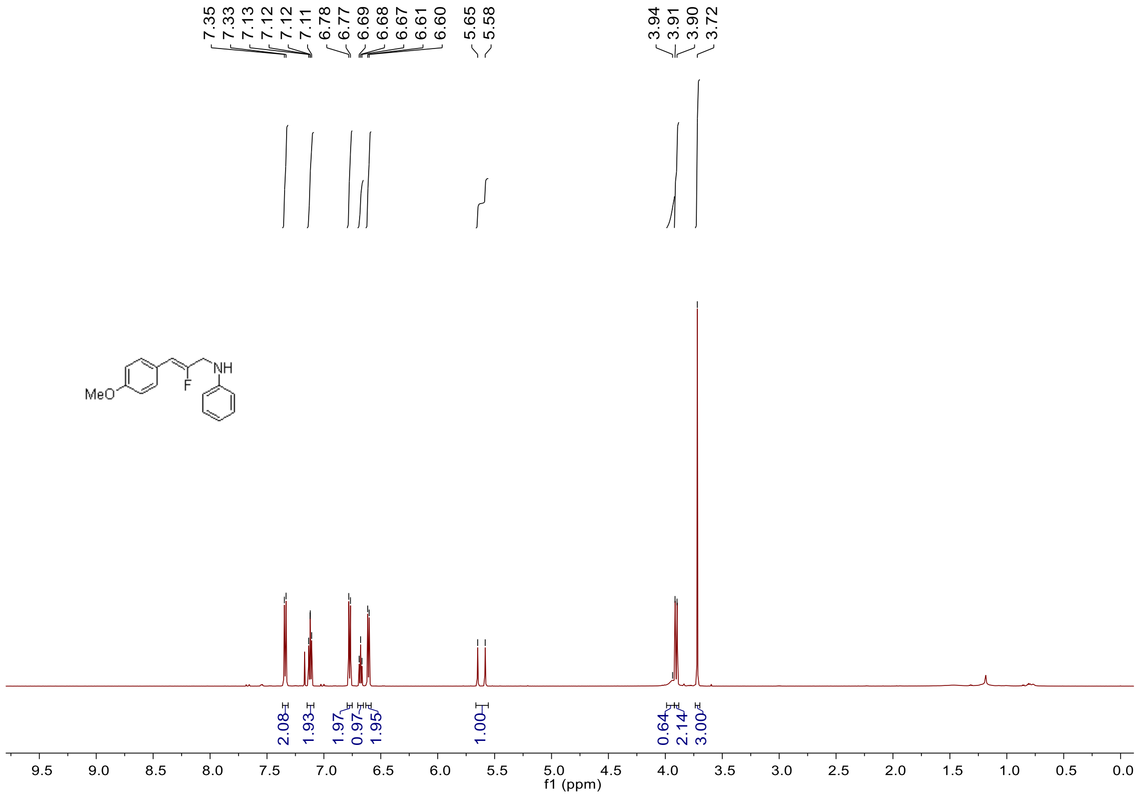

^{19}F NMR (**c3**)

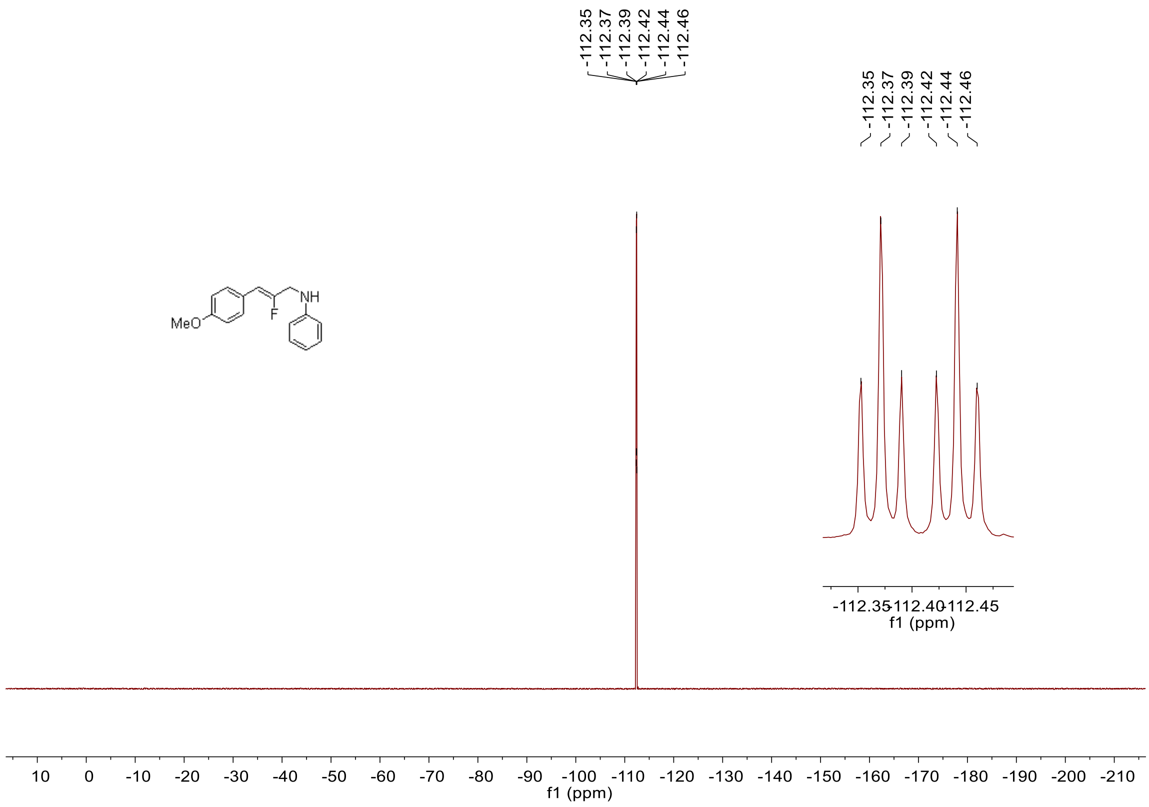

^{13}C NMR (**c3**)

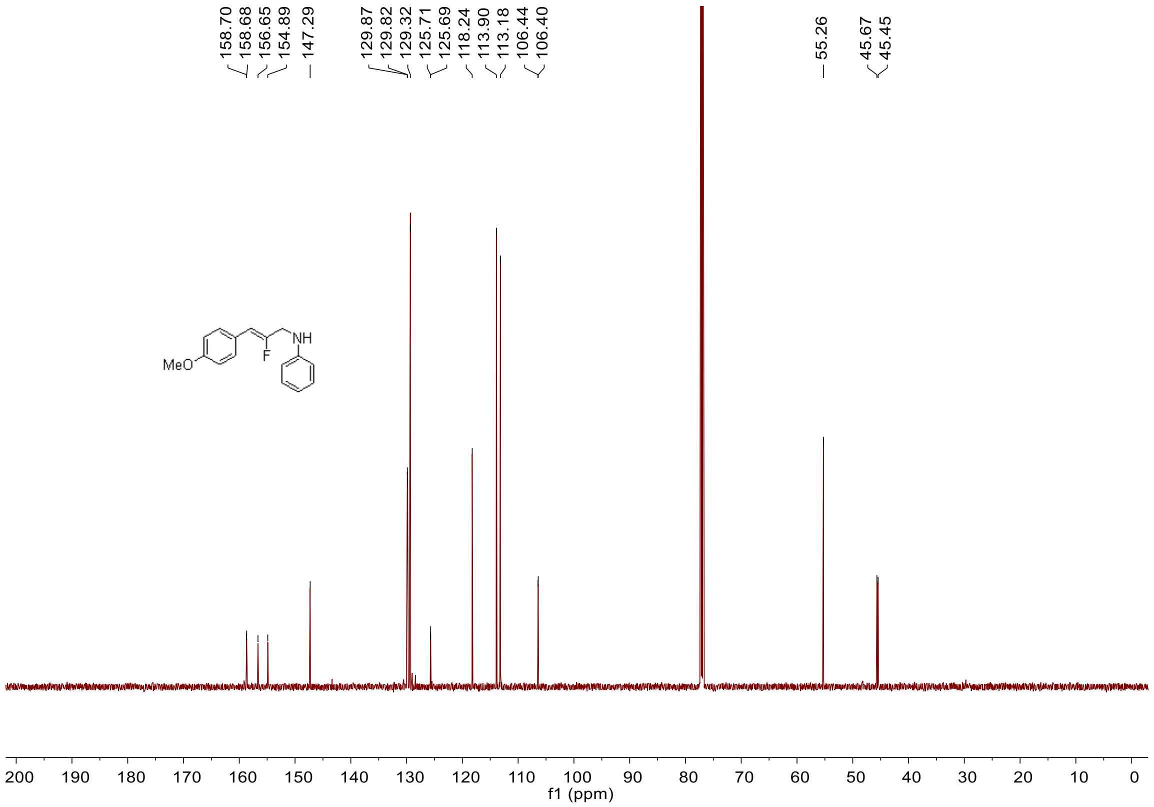

^{1}H NMR (**c4**)

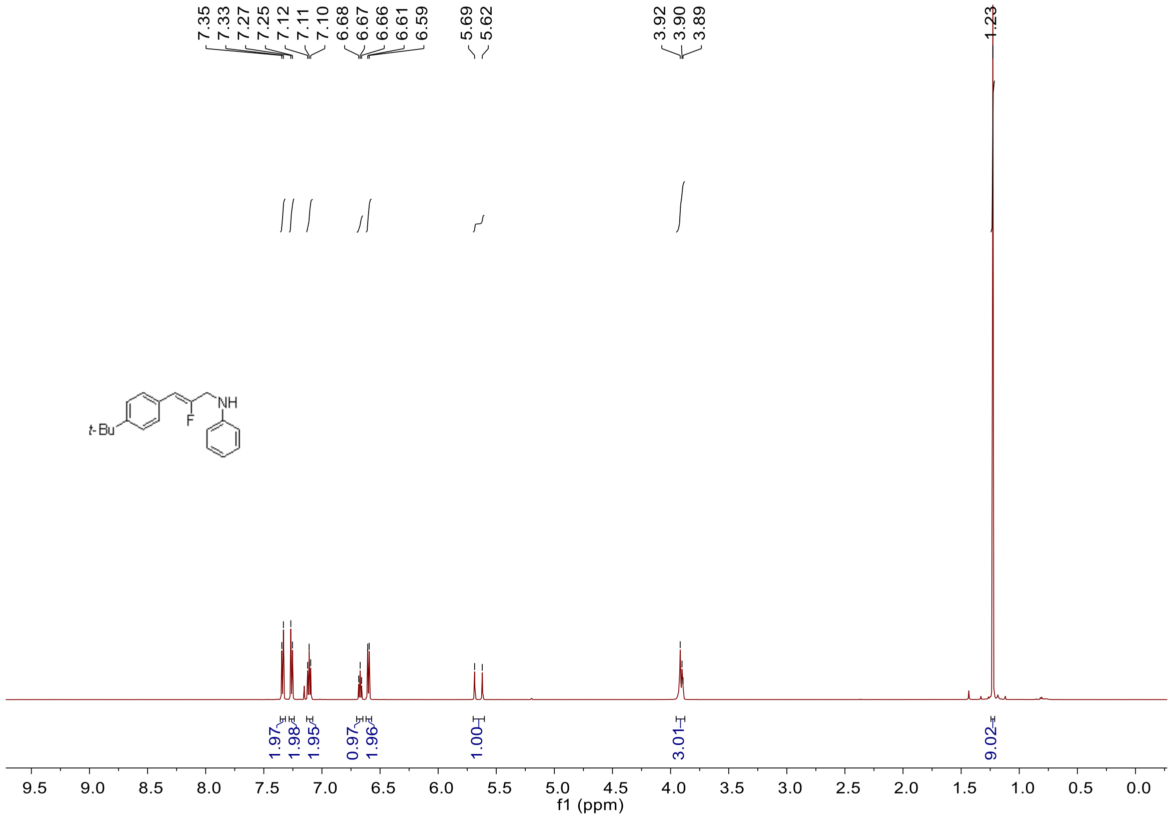

^{19}F NMR (**c4**)

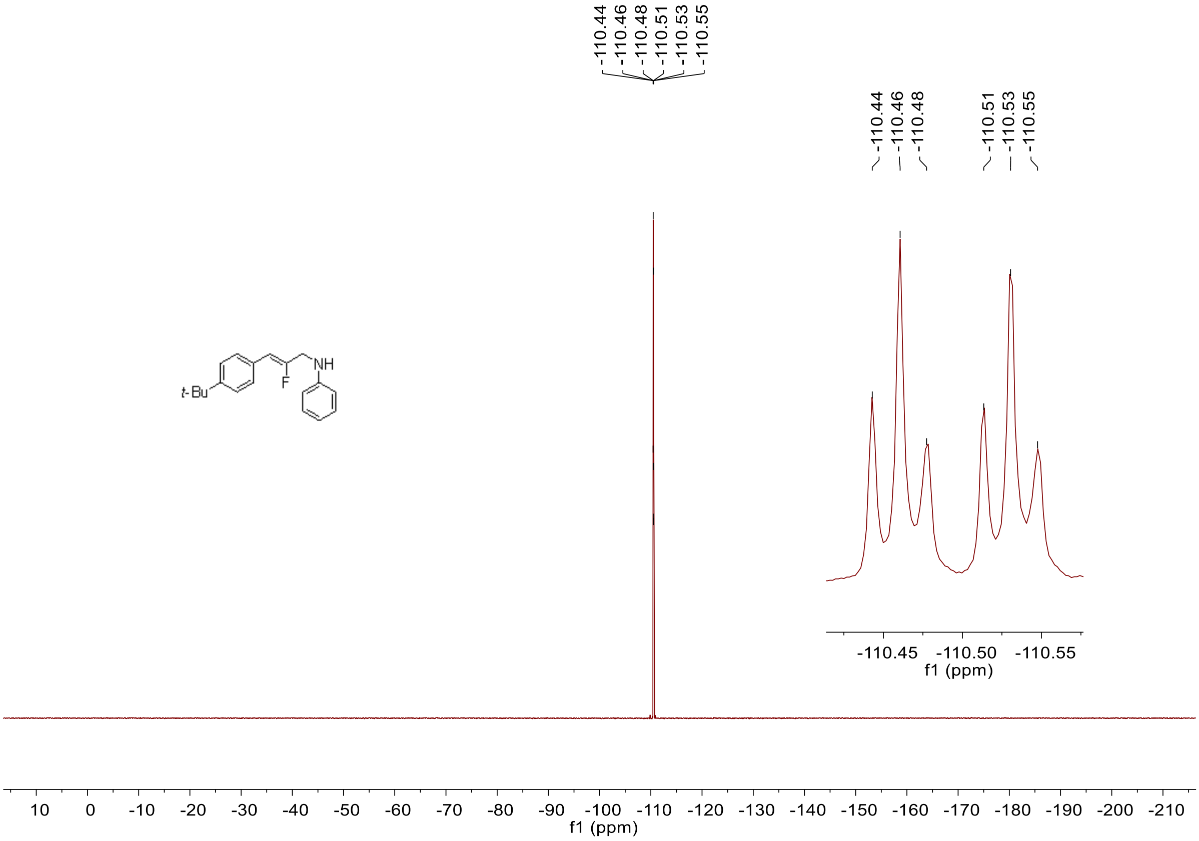

^{13}C NMR (**c4**)

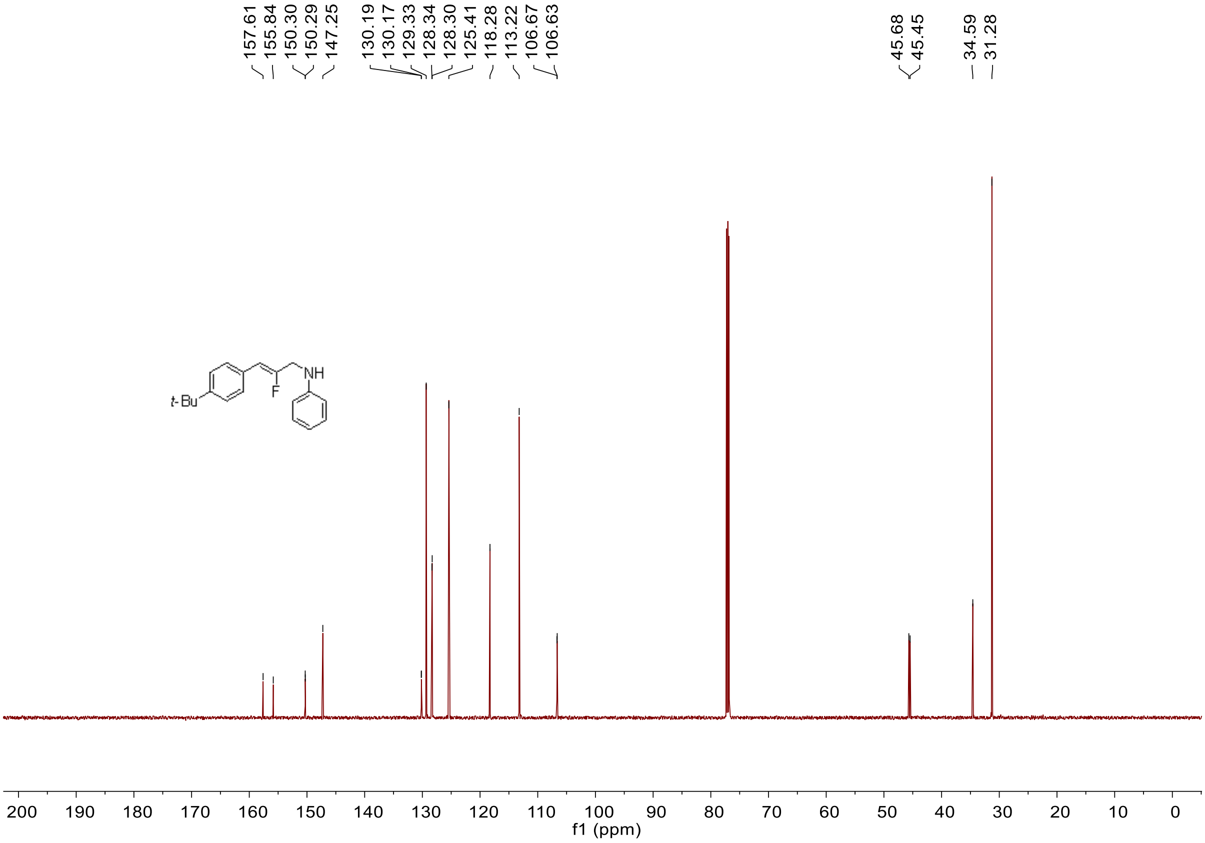

^{1}H NMR (**c5**)

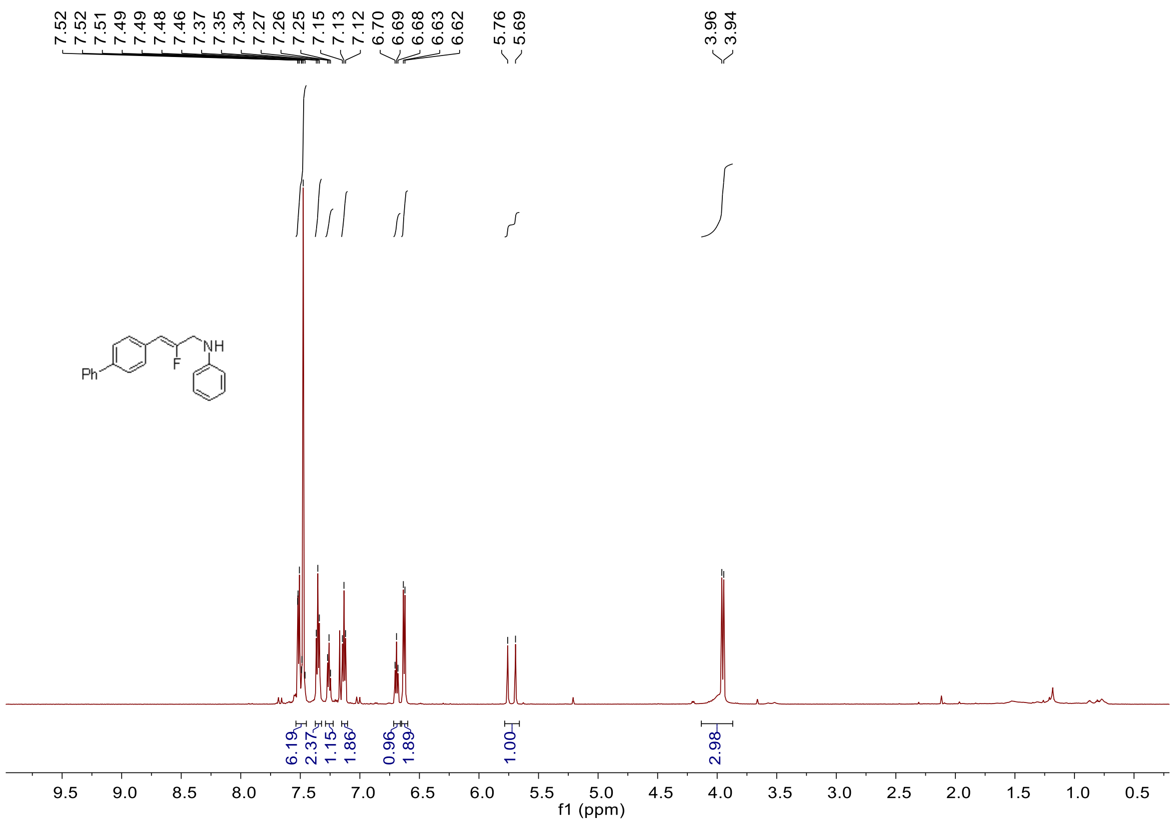

^{19}F NMR (**c5**)

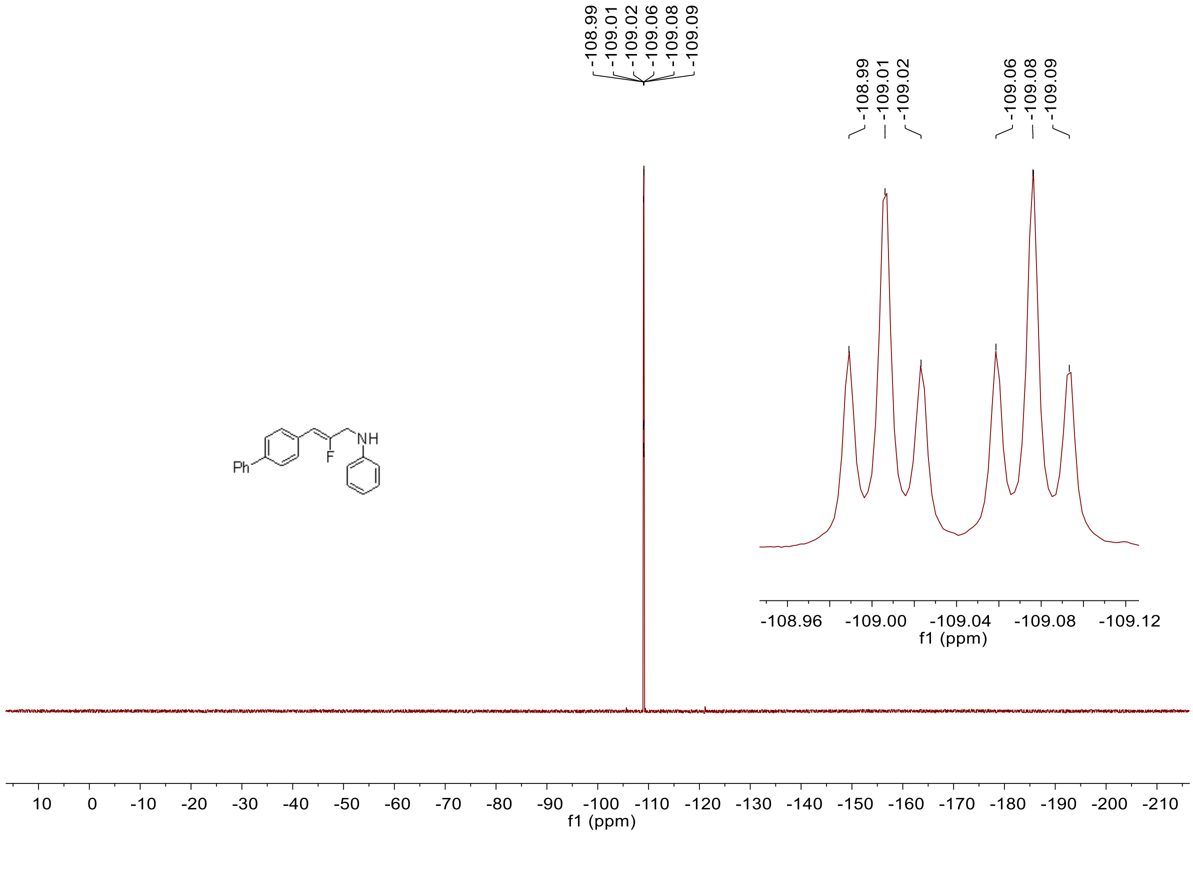

^{13}C NMR (**c5**)

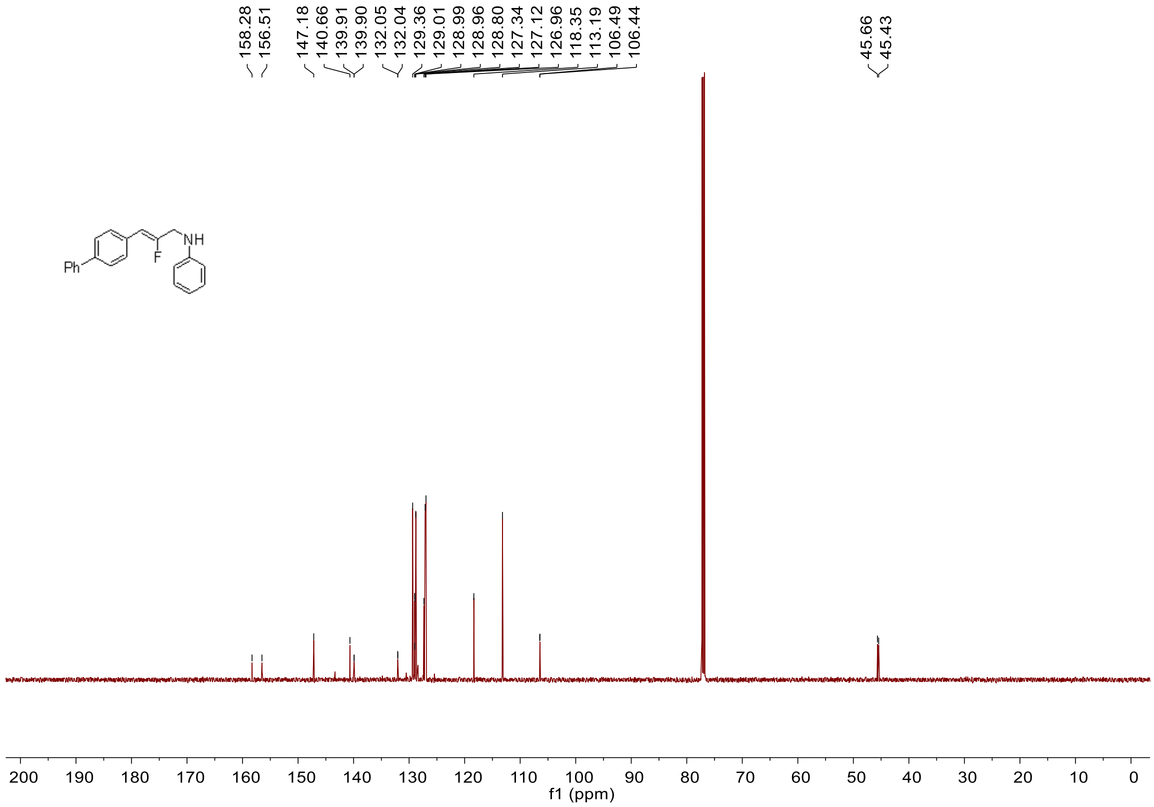

^{1}H NMR (**c6**)

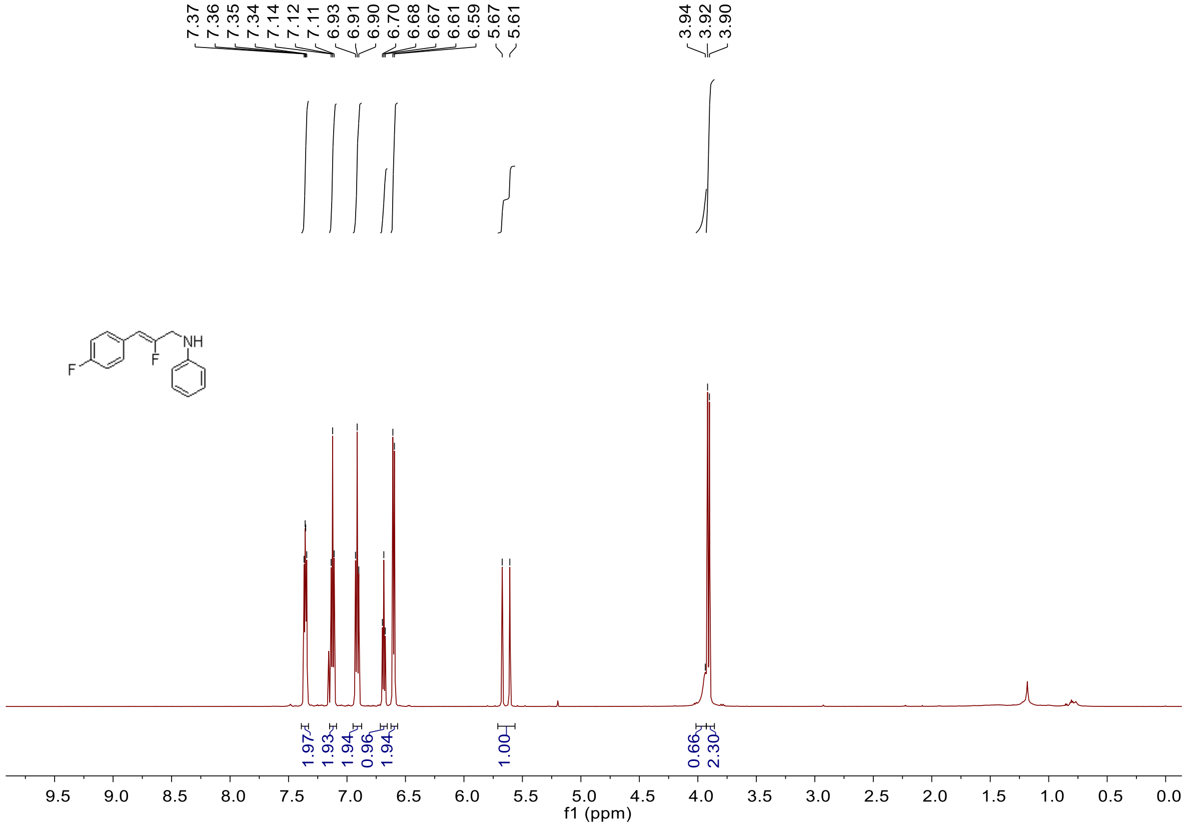

^{19}F NMR (**c6**)

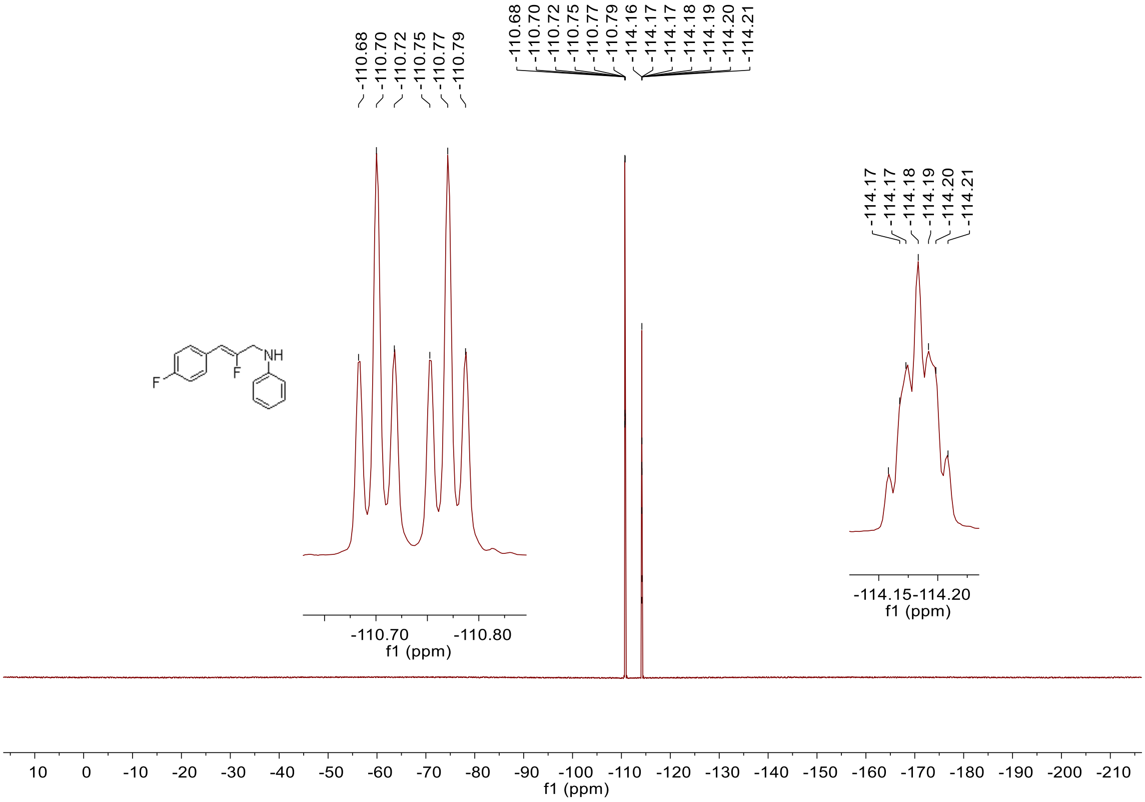

^{13}C NMR (**c6**)

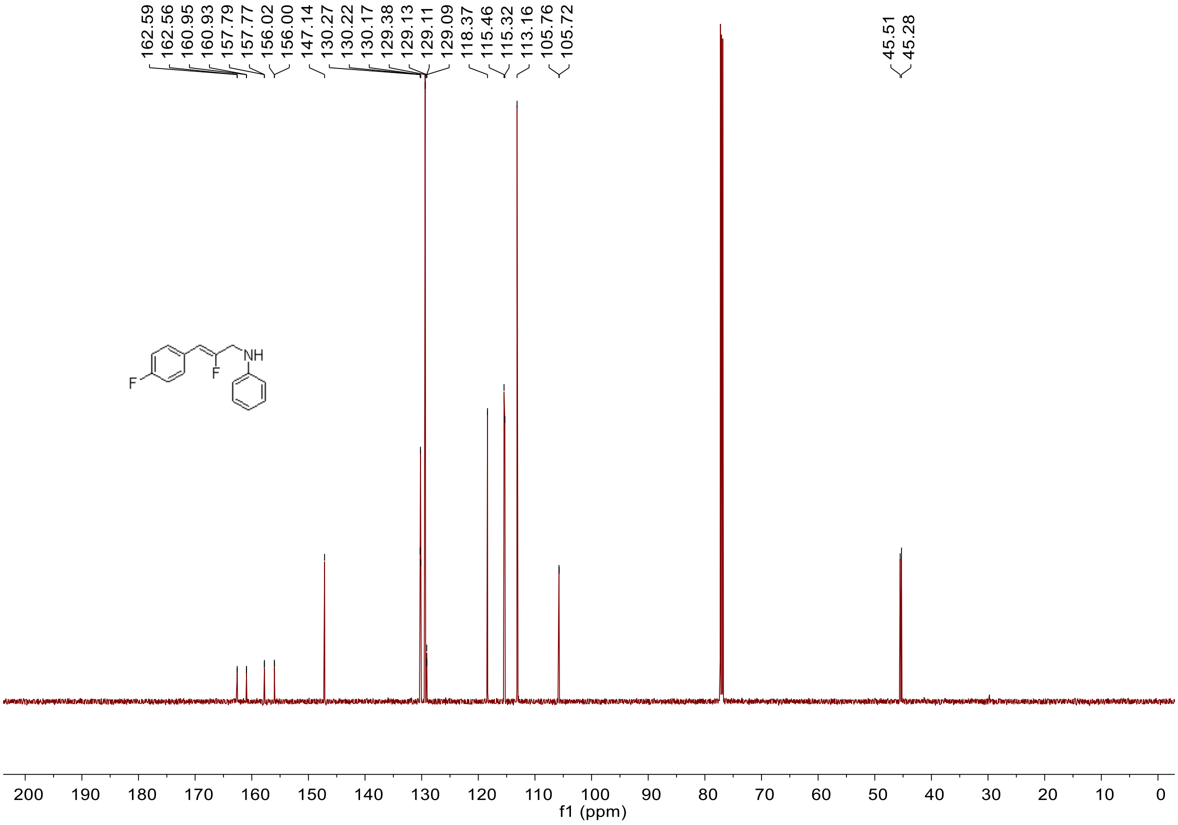

^{1}H NMR (**c7**)

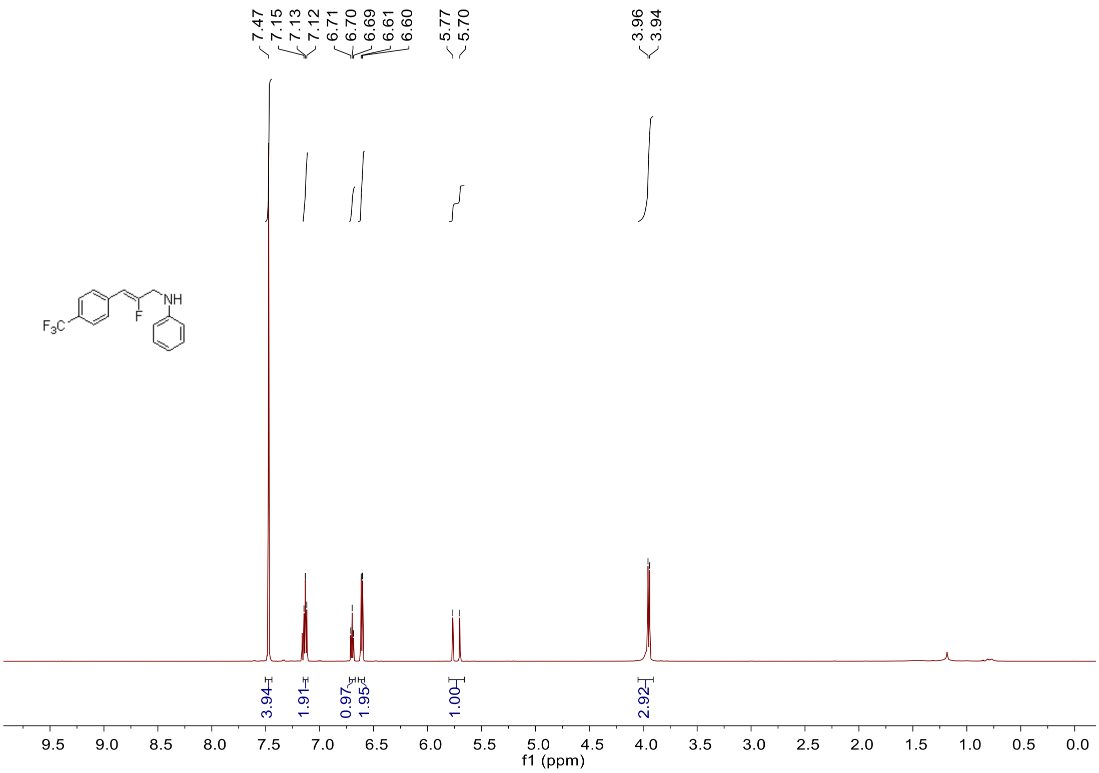

^{19}F NMR (**c7**)

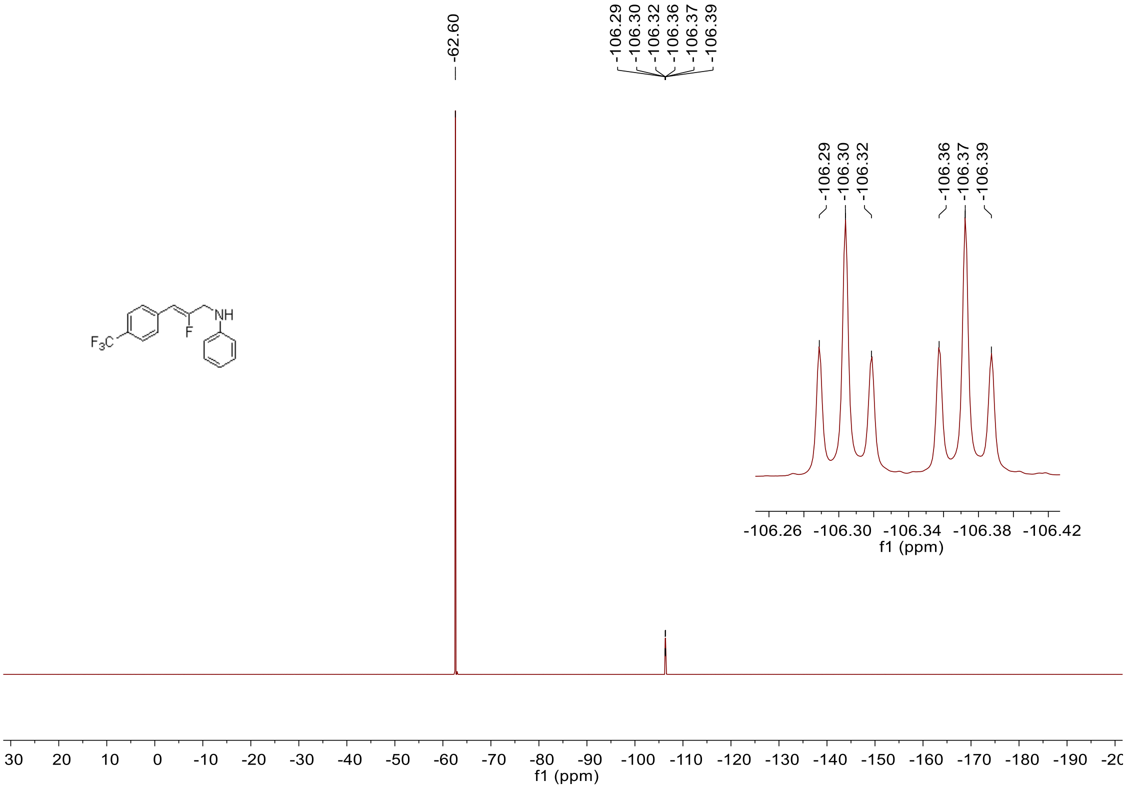

^{13}C NMR (**c7**)

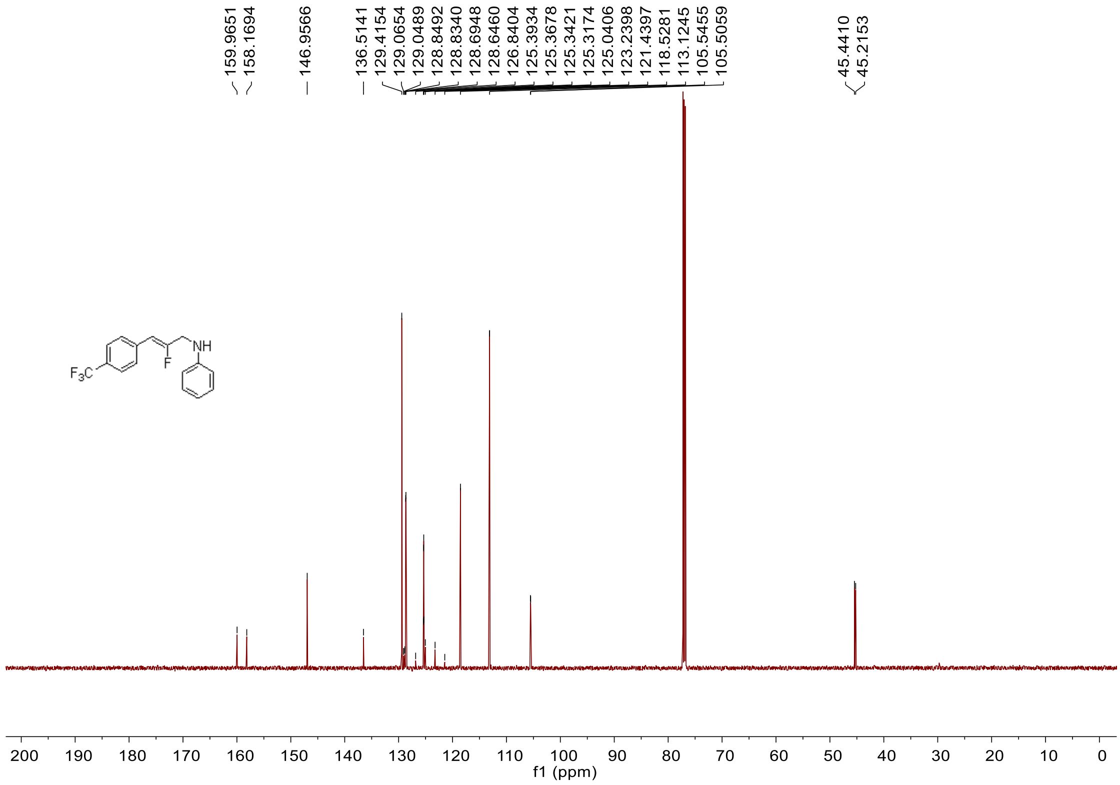

^{1}H NMR (**c8**)

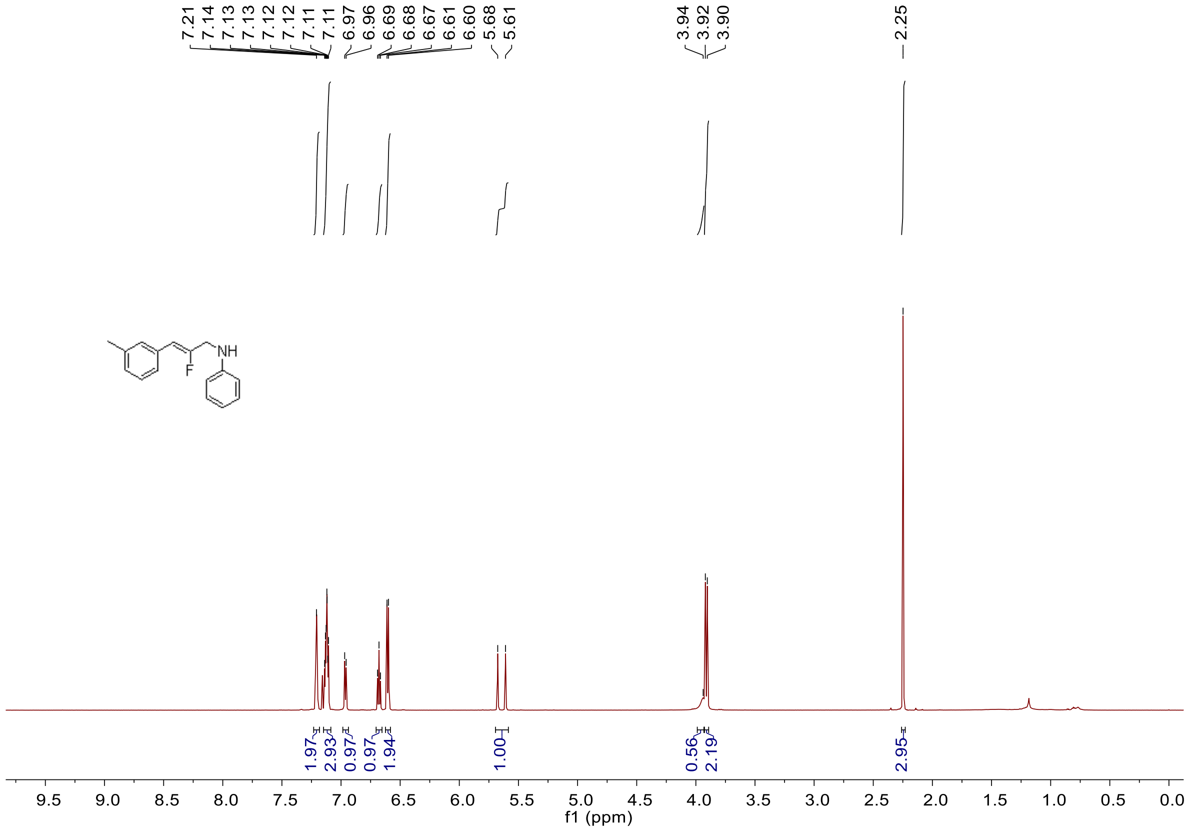

^{19}F NMR (**c8**)

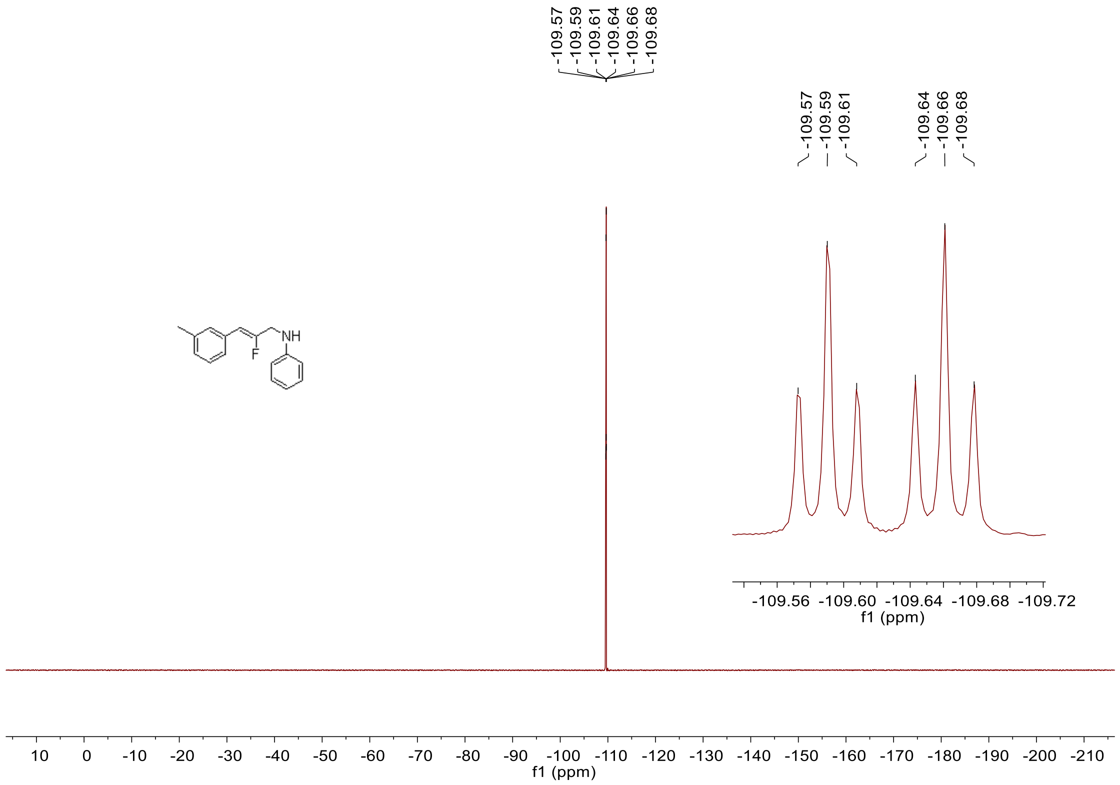

^{13}C NMR (**c8**)

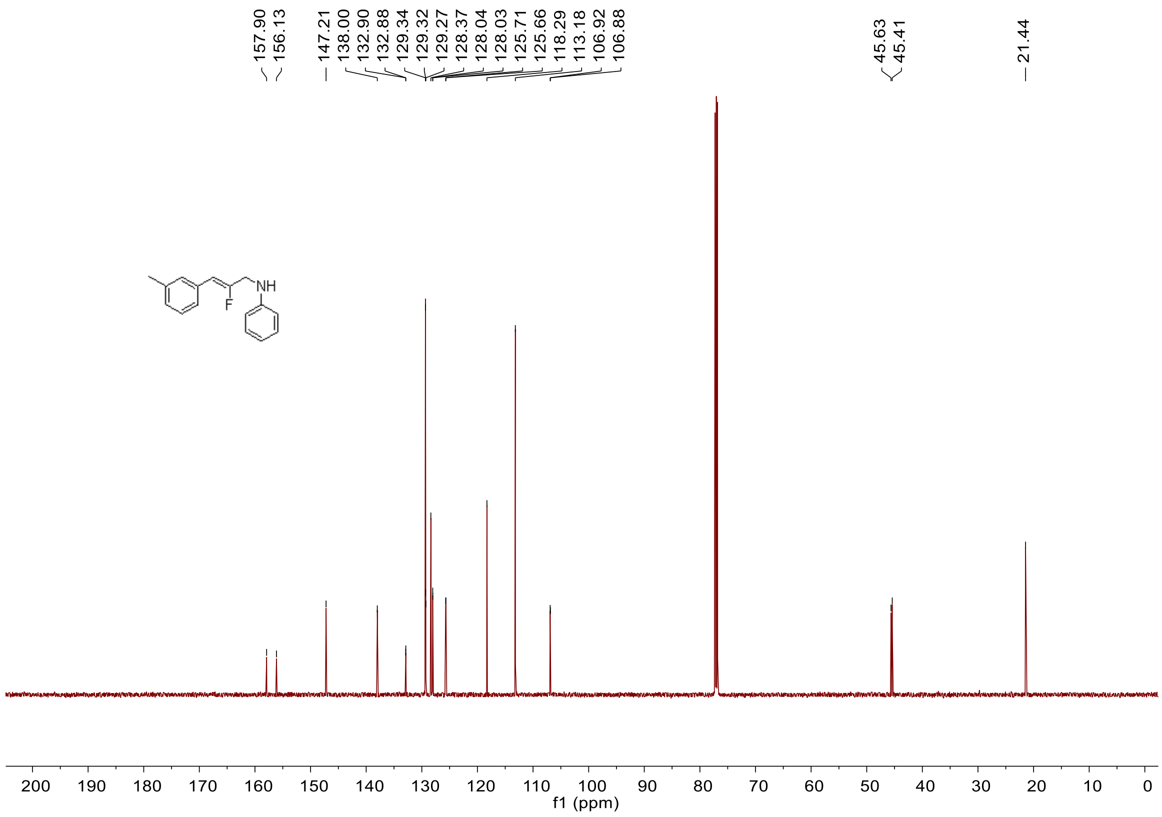

^{1}H NMR (**c9**)

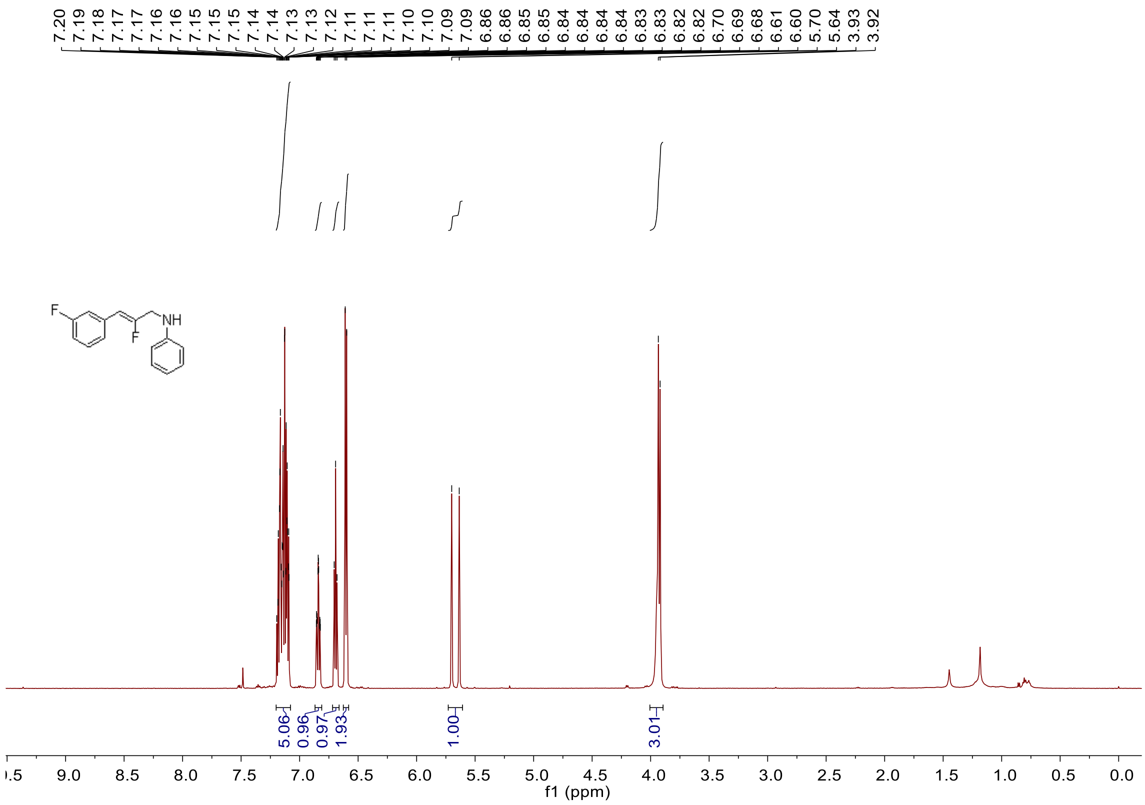

^{19}F NMR (**c9**)

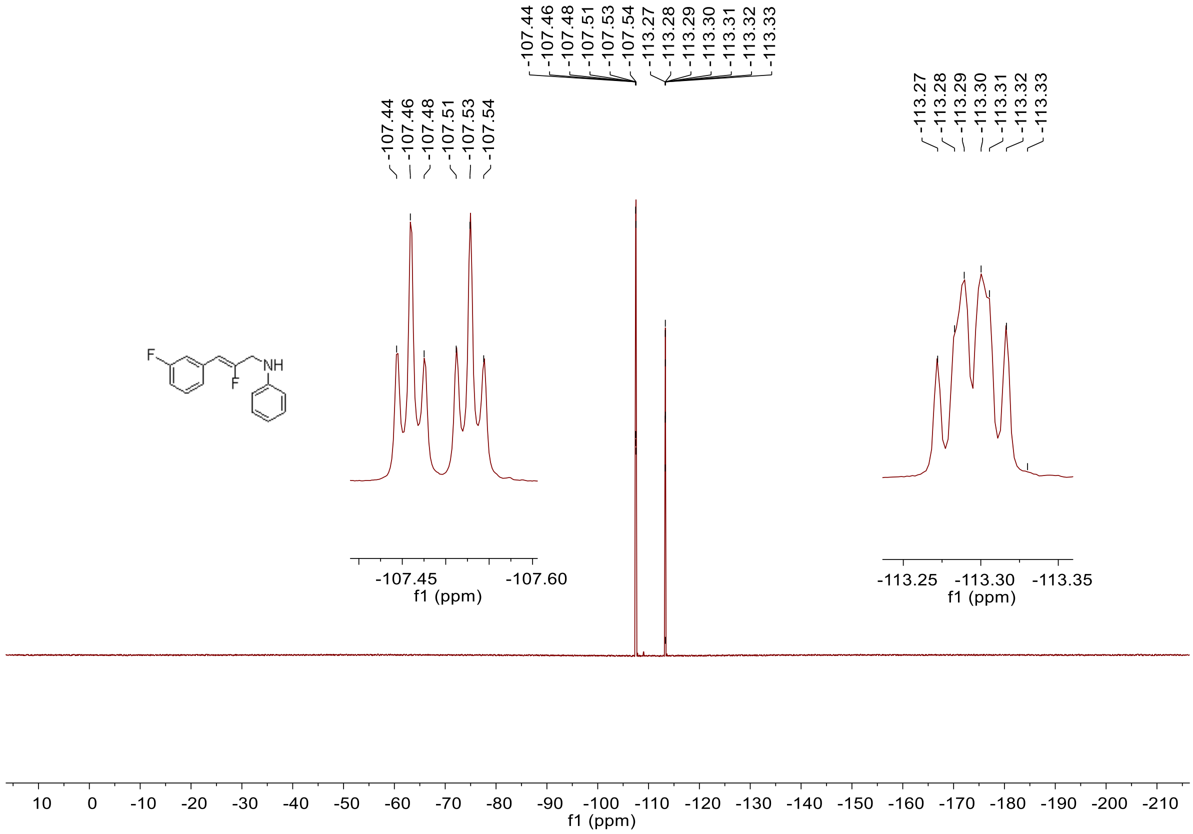

^{13}C NMR (**c9**)

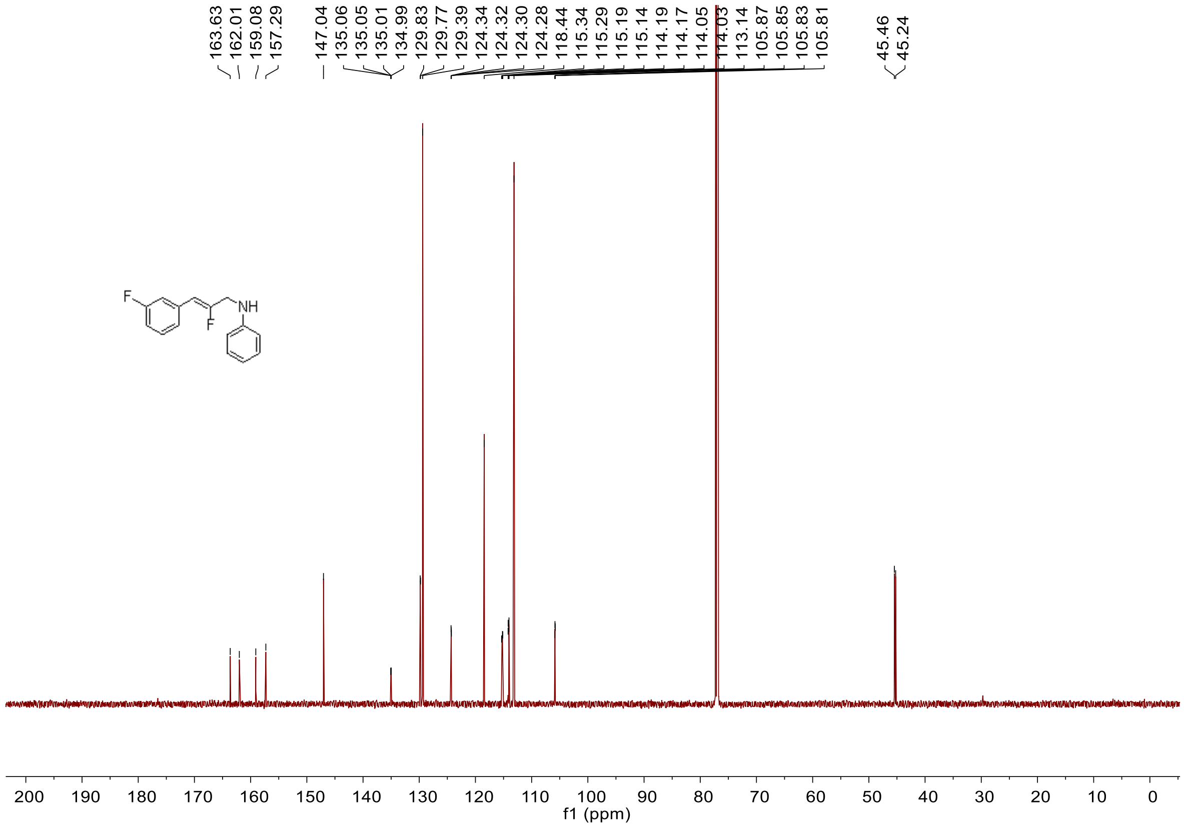

^{1}H NMR (**c10**)

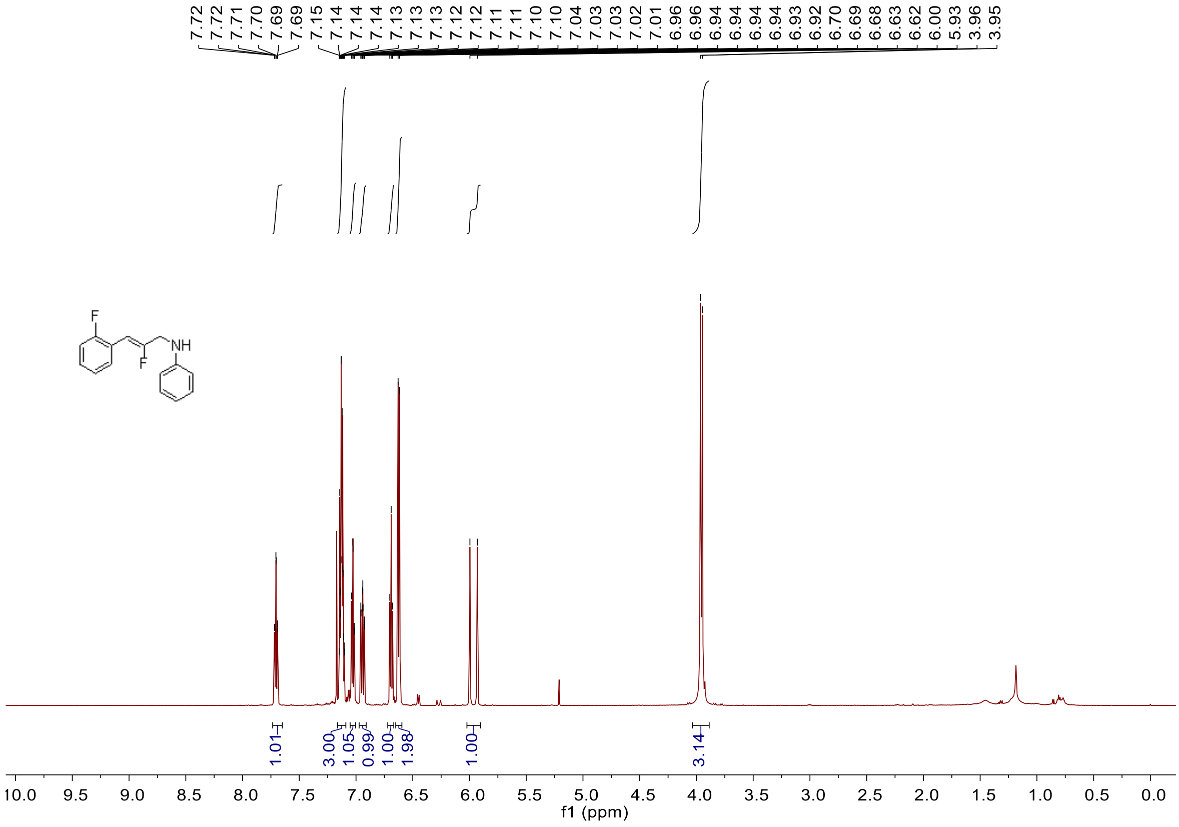

^{19}F NMR (**c10**)

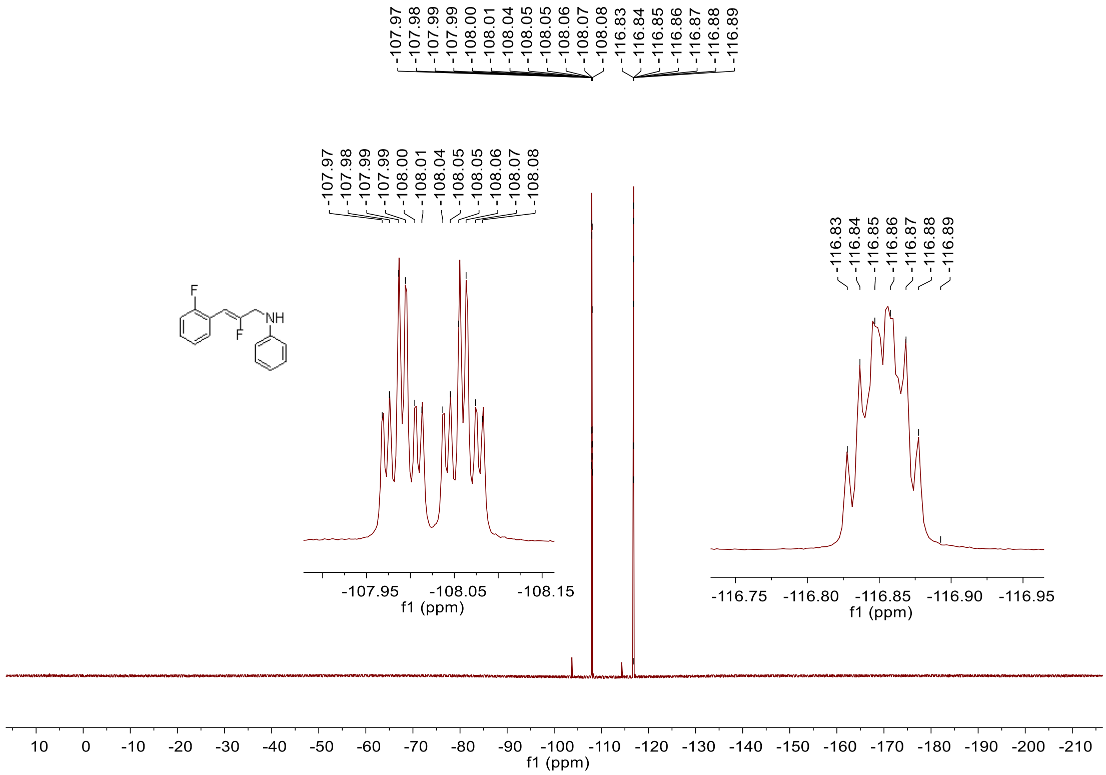

^{13}C NMR (**c10**)

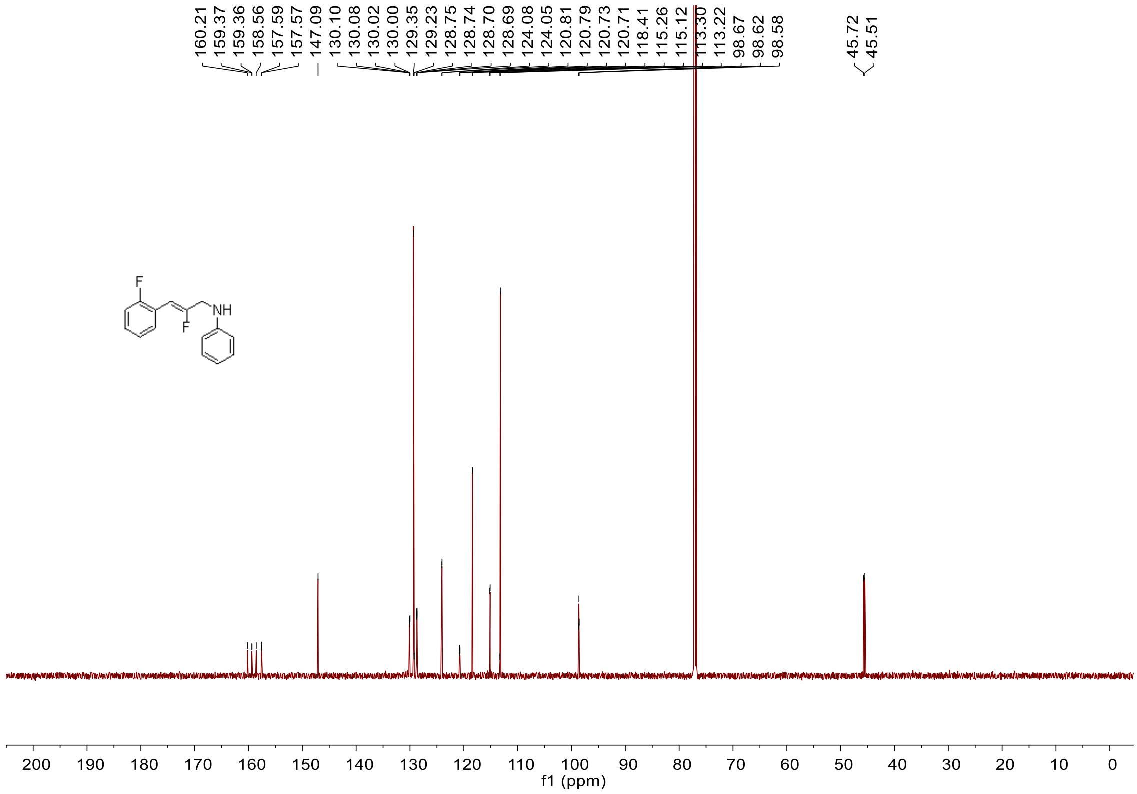

^{1}H NMR (**c11**)

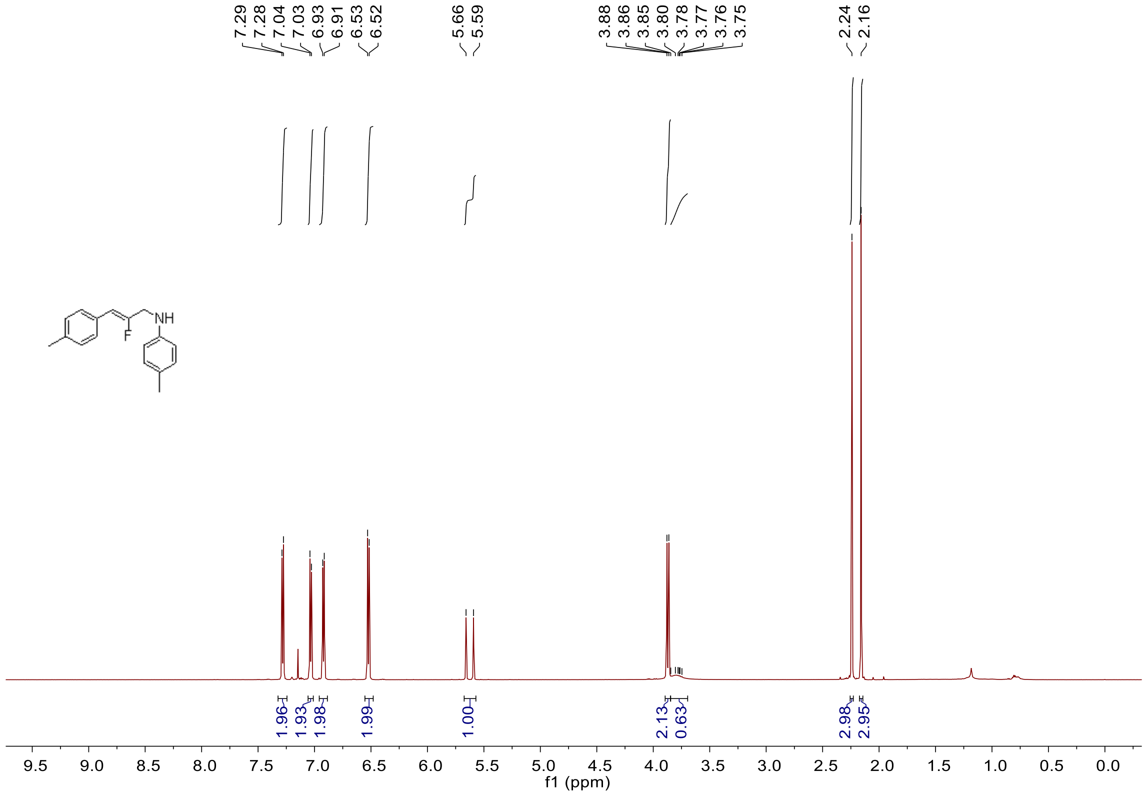

^{19}F NMR (**c11**)

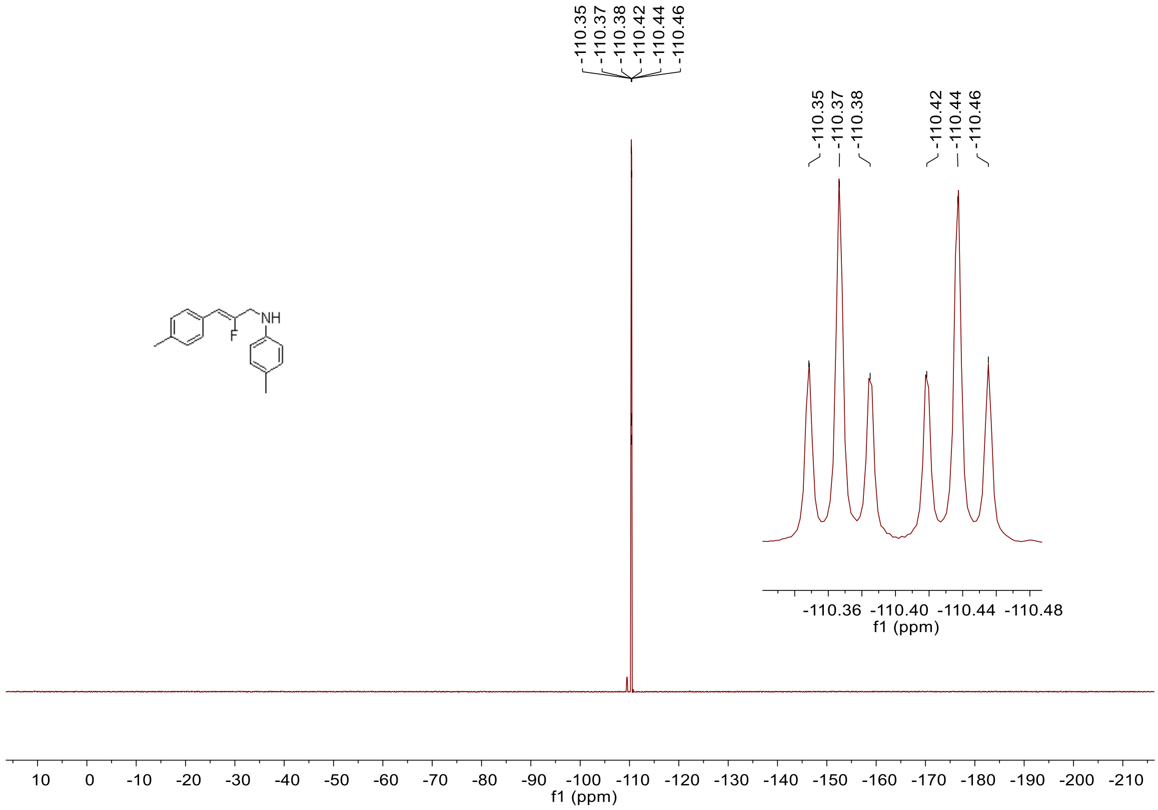

^{13}C NMR (**c11**)

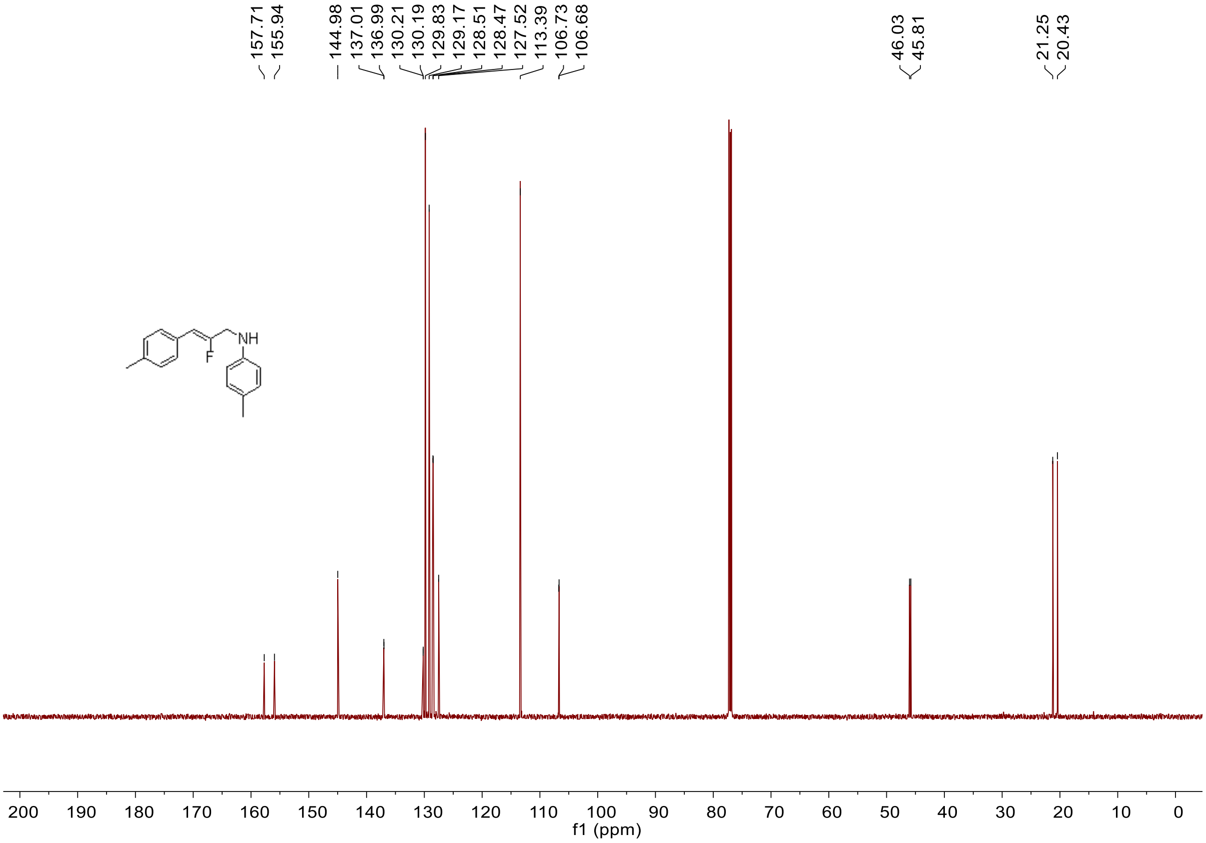

^{1}H NMR (**c12**)

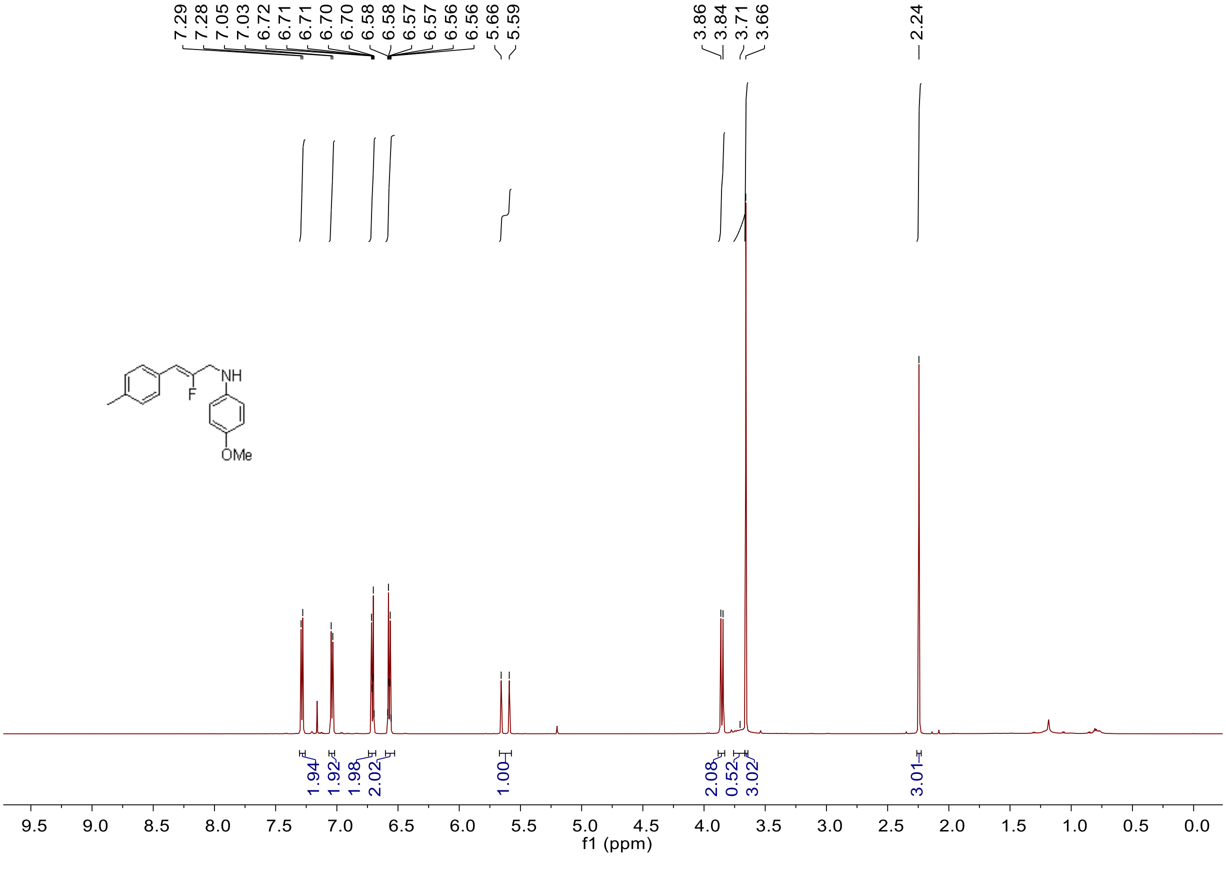

^{19}F NMR (**c12**)

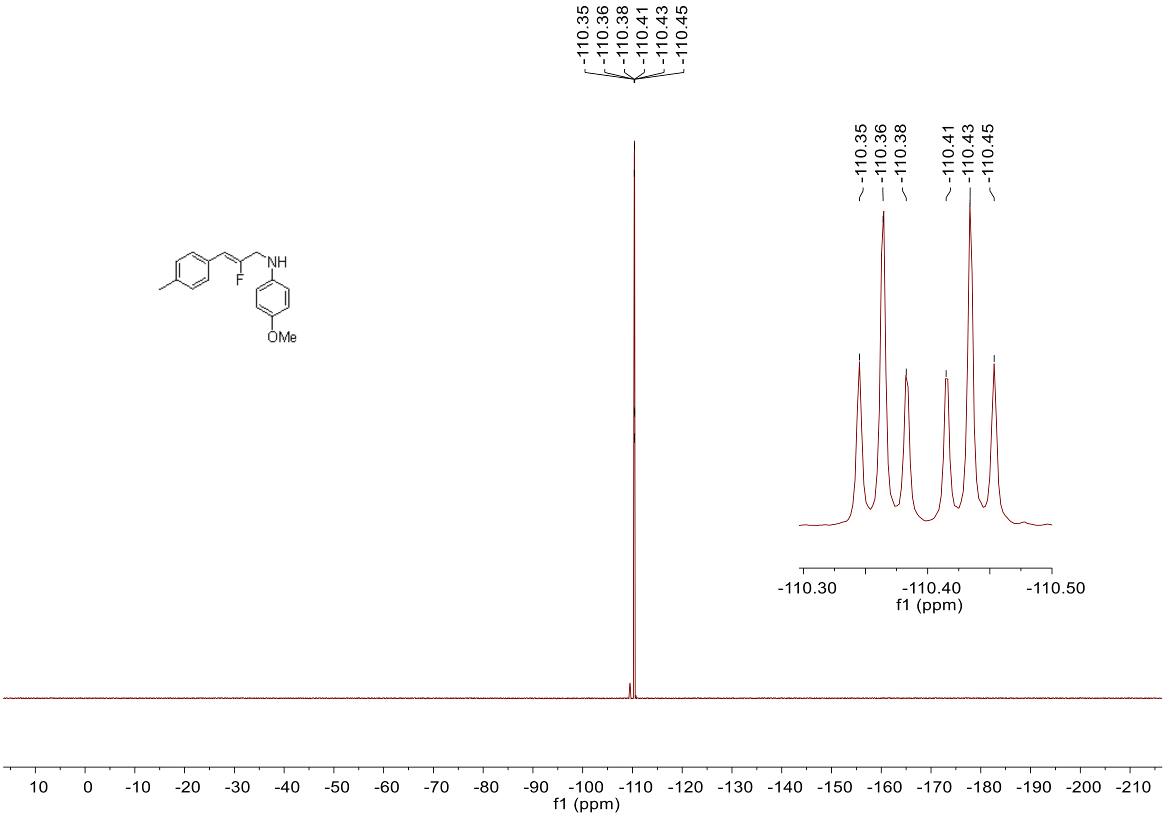

^{13}C NMR (**c12**)

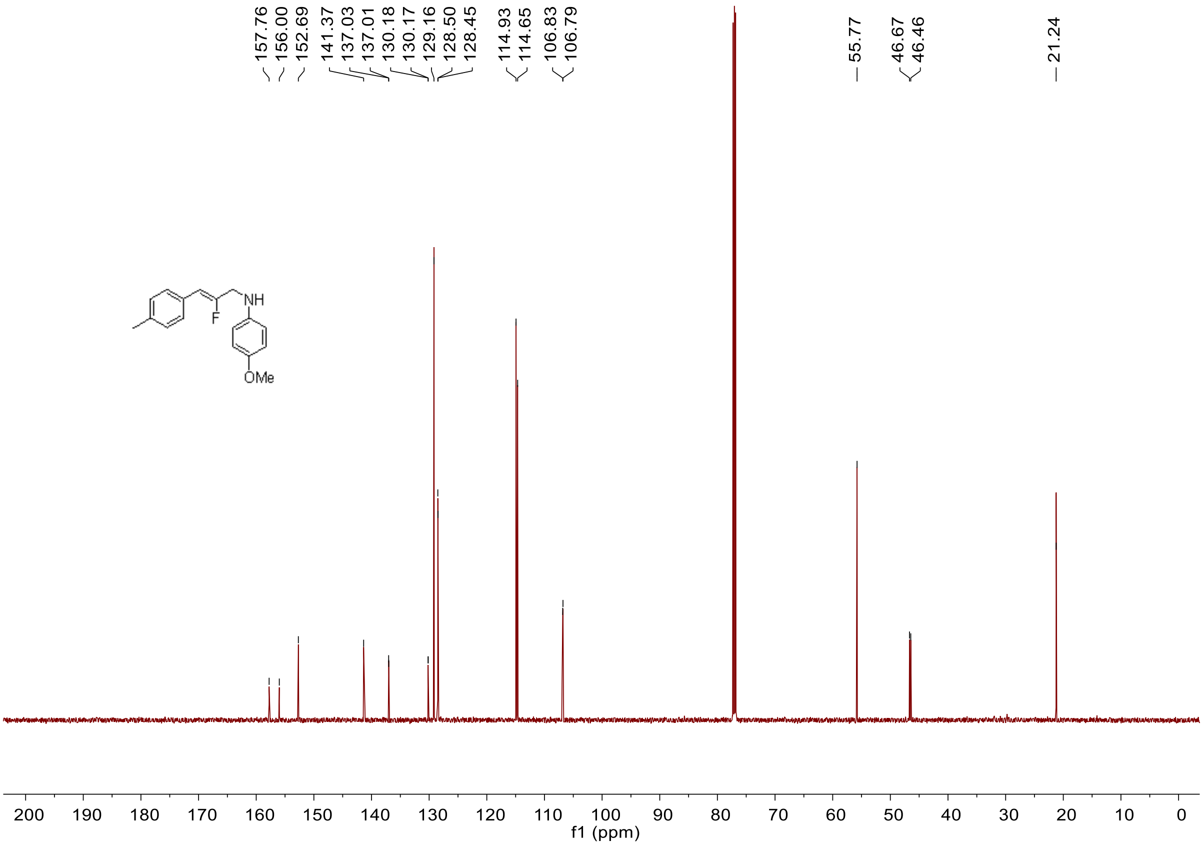

^{1}H NMR (**c13**)

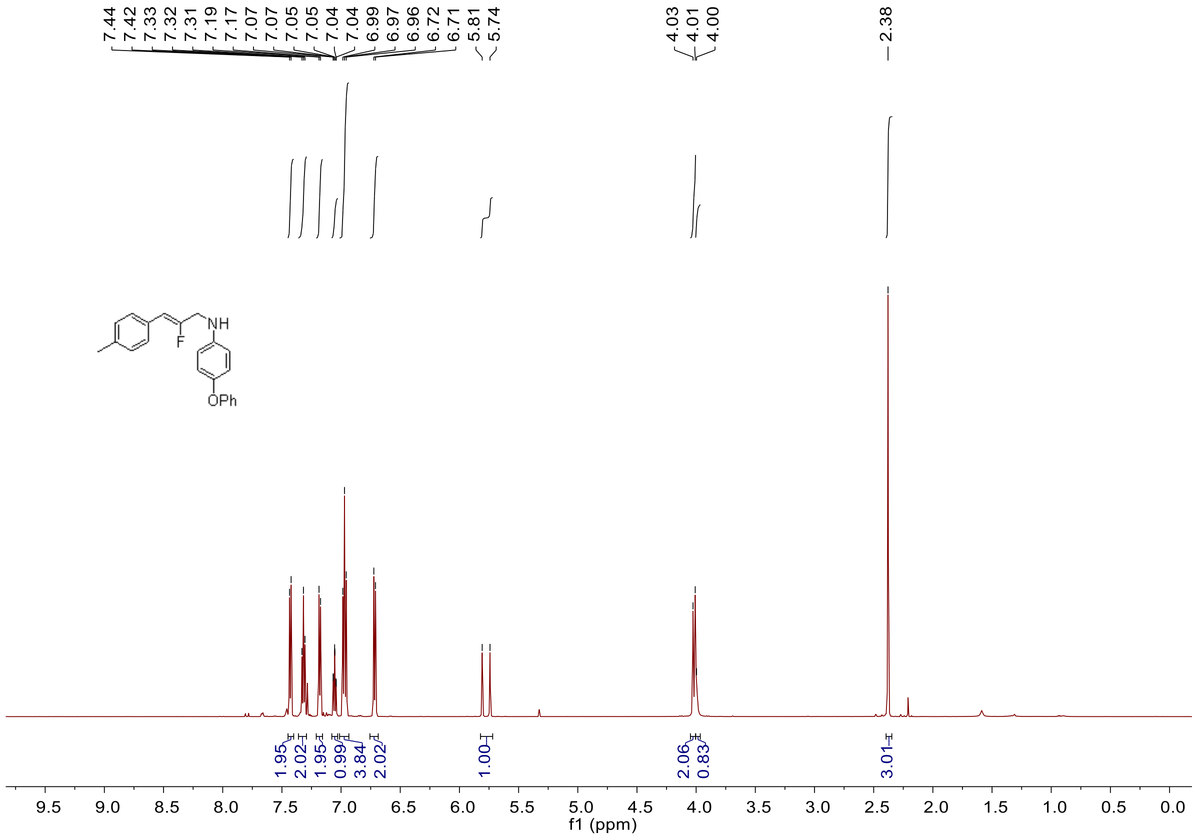

^{19}F NMR (**c13**)

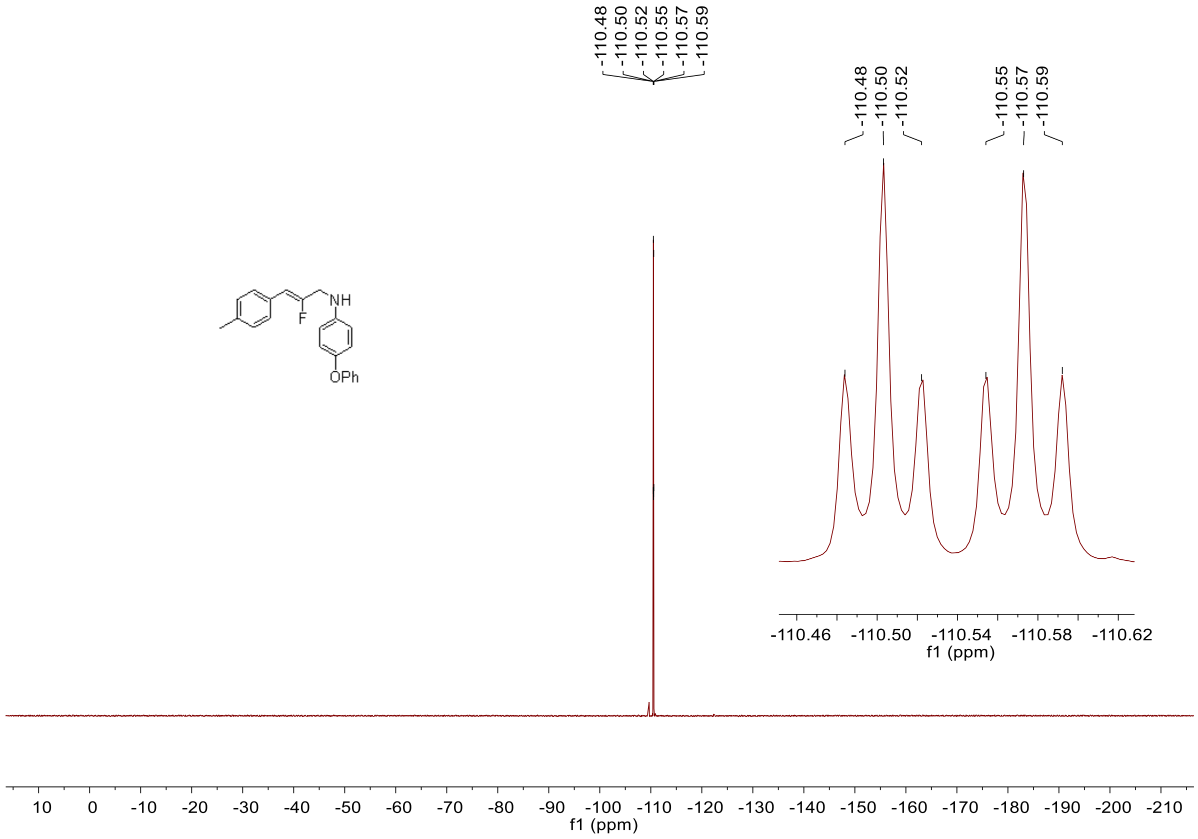

^{13}C NMR (**c13**)

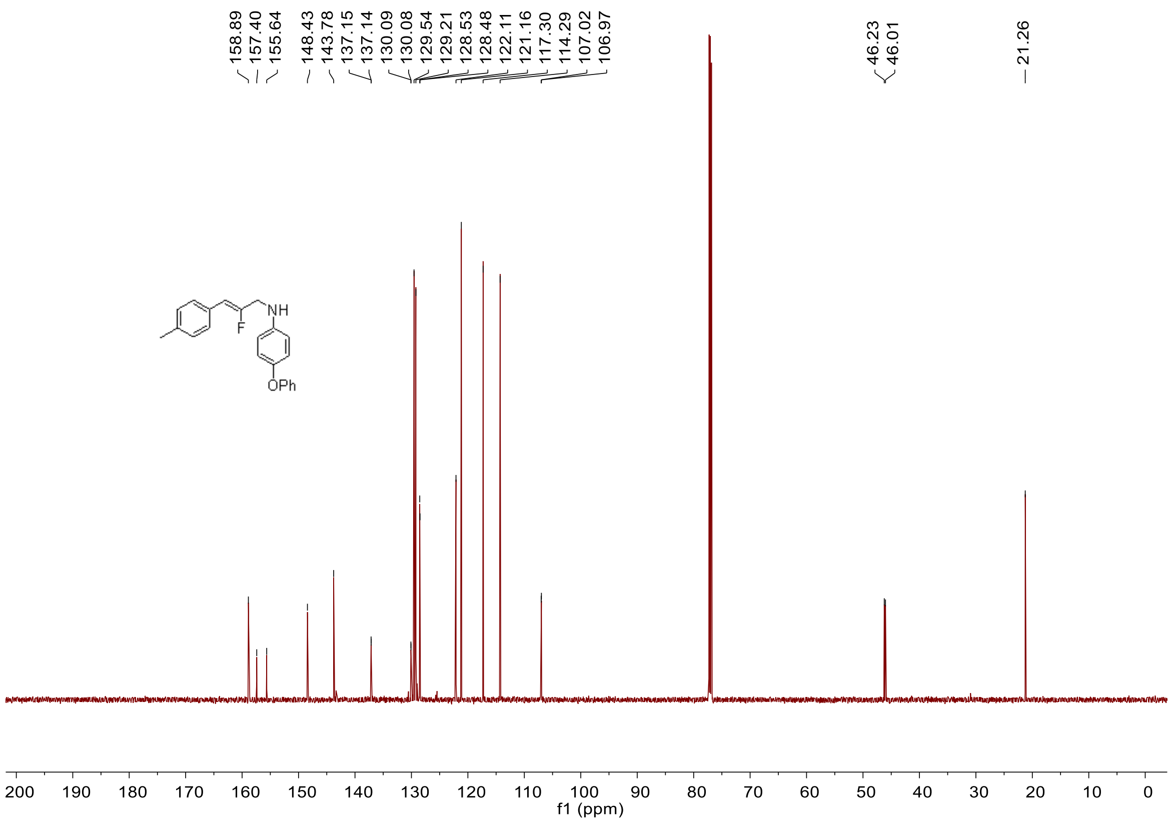

^{1}H NMR (**c14**)

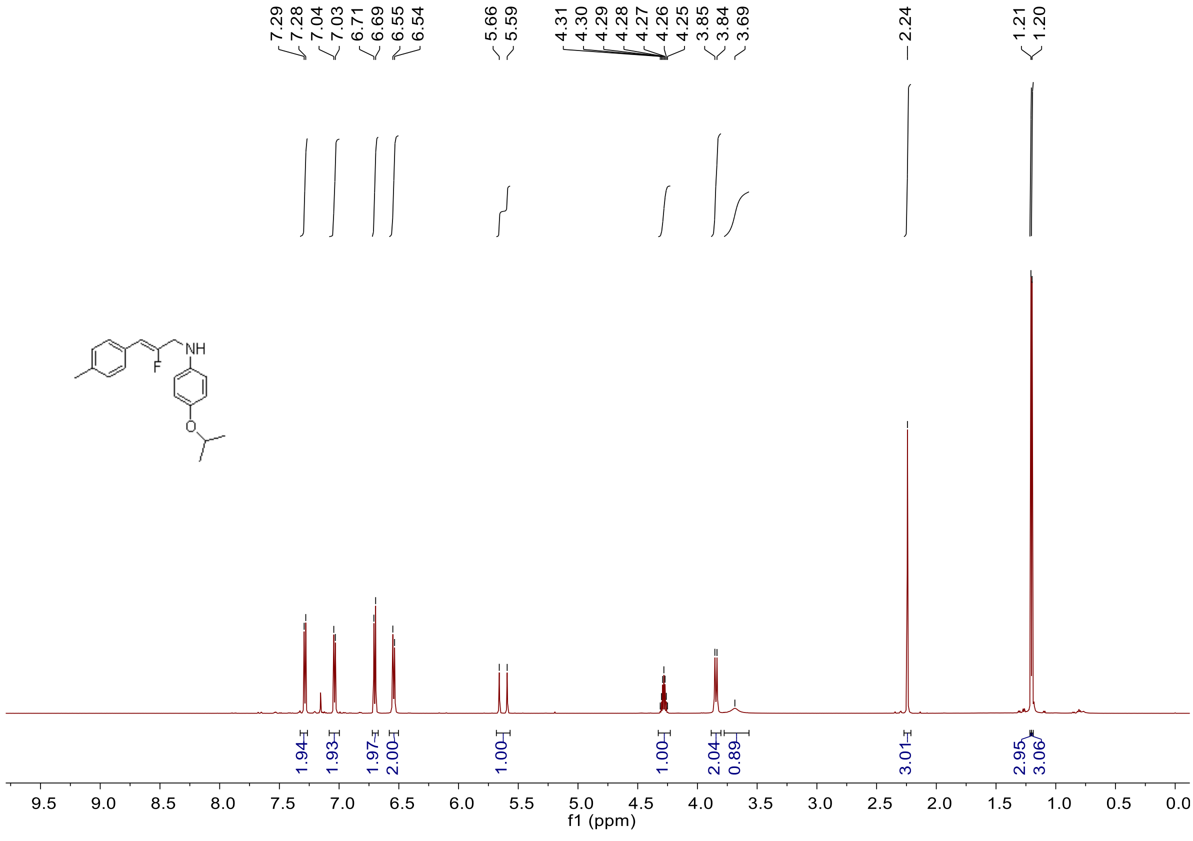

^{19}F NMR (**c14**)

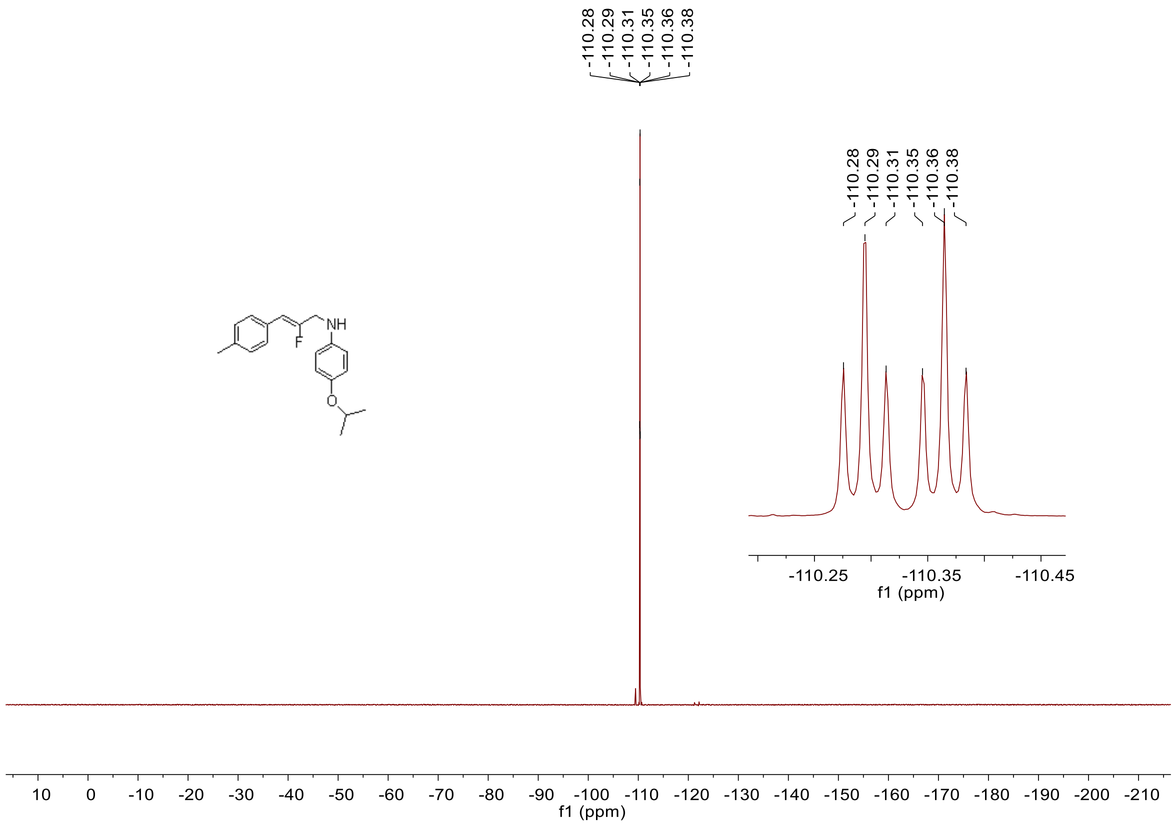

^{13}C NMR (**c14**)

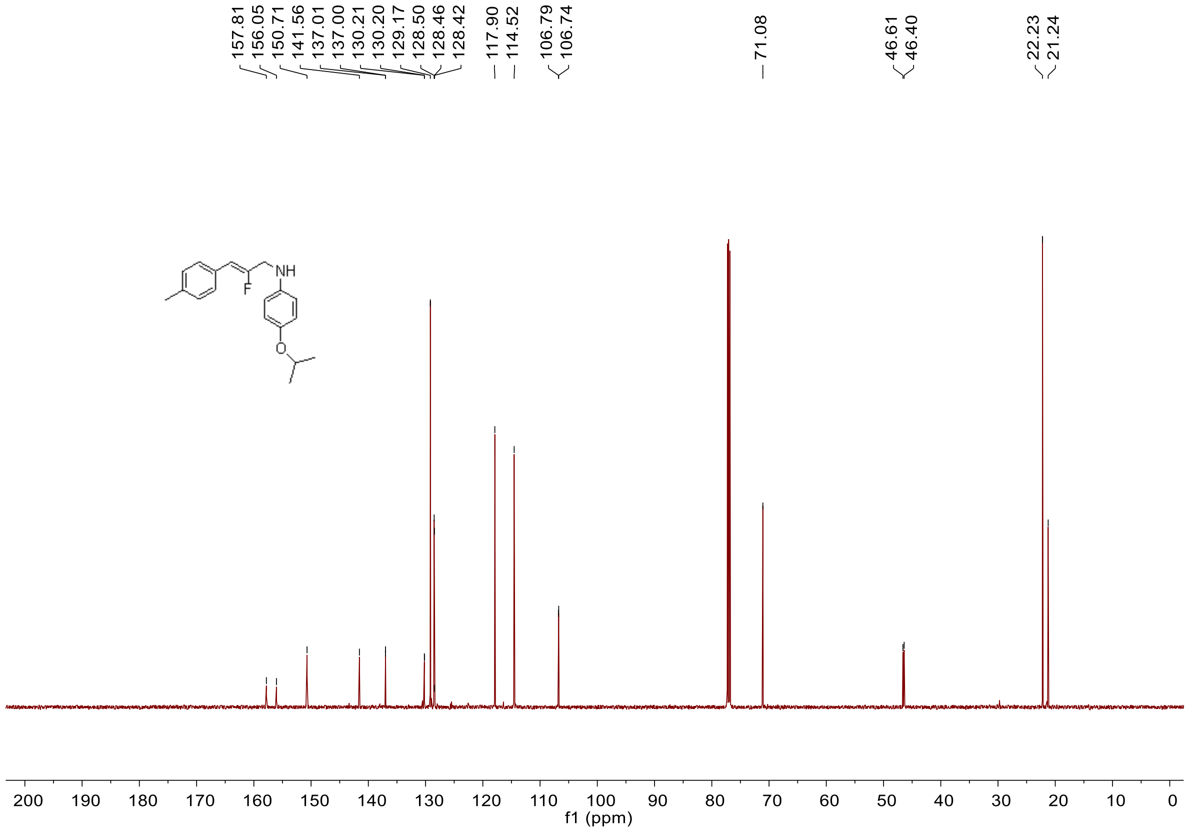

^{1}H NMR (**c15**)

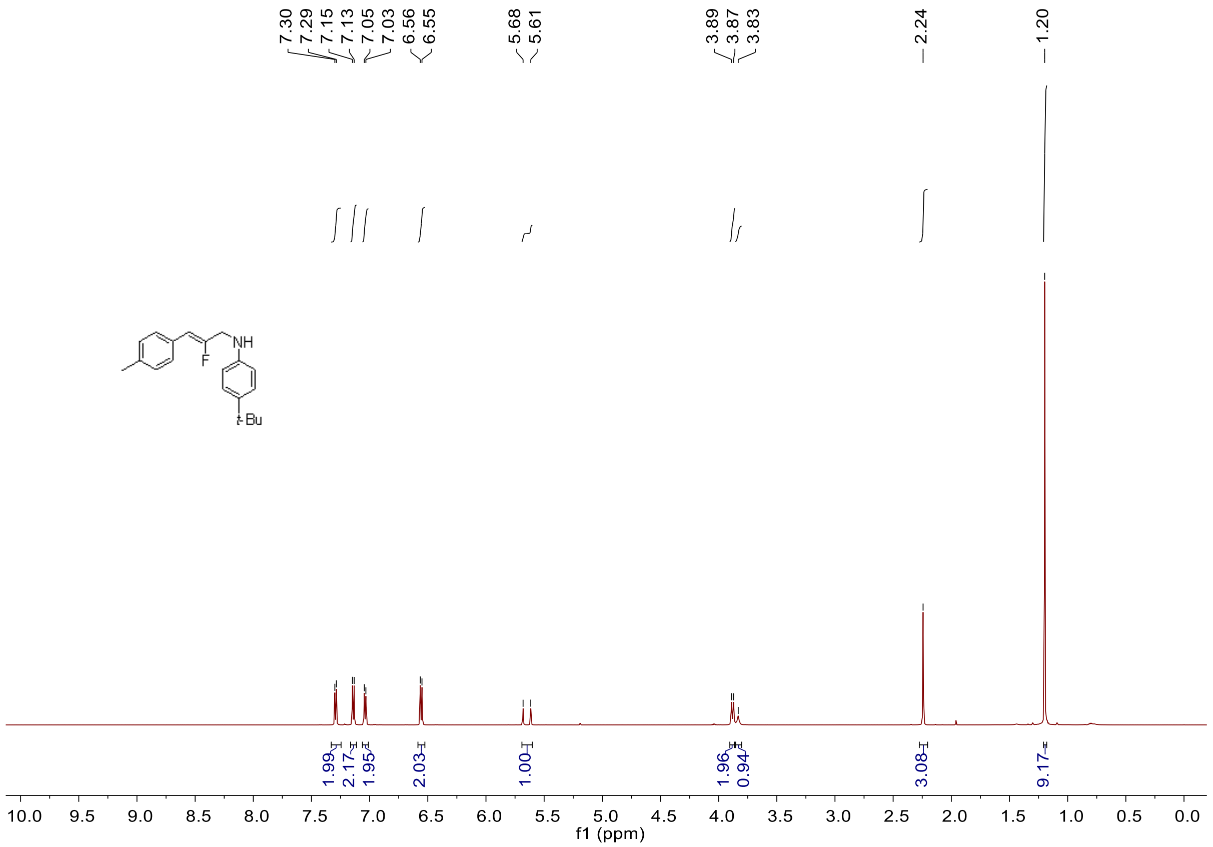

^{19}F NMR (**c15**)

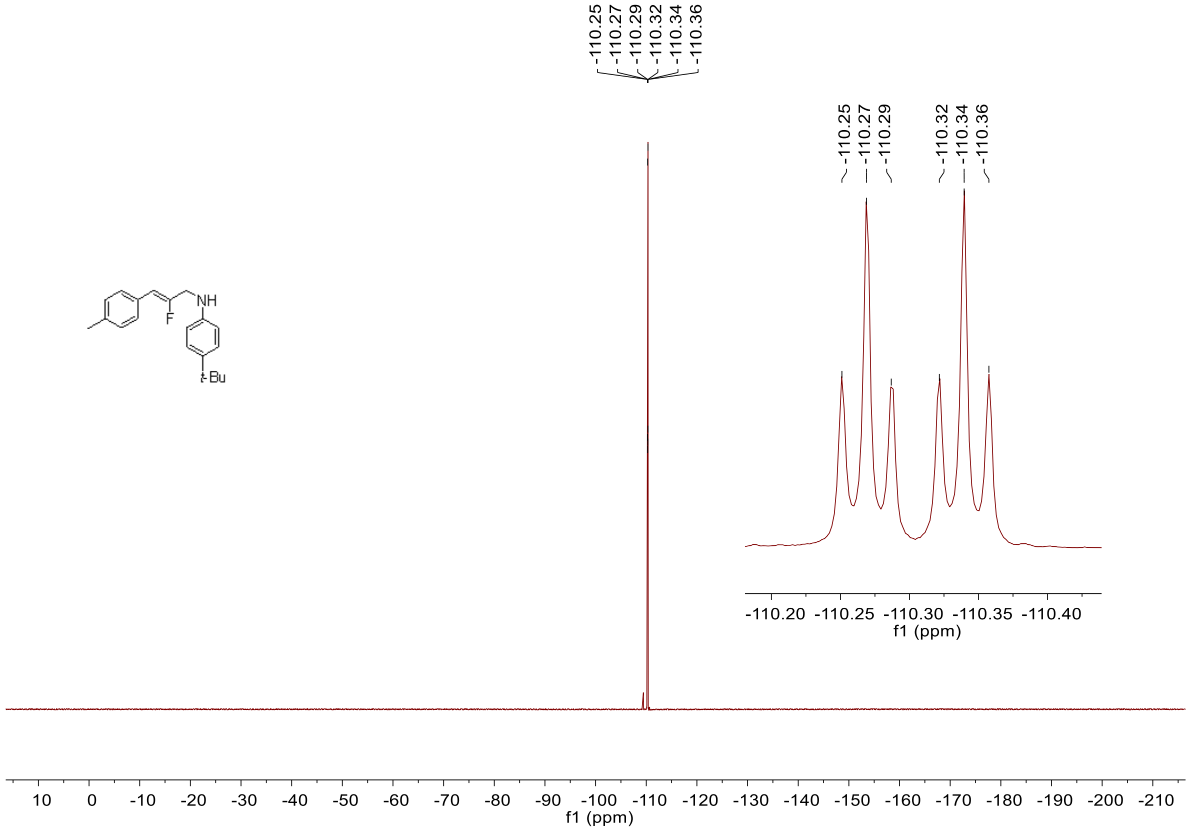

^{13}C NMR (**c15**)

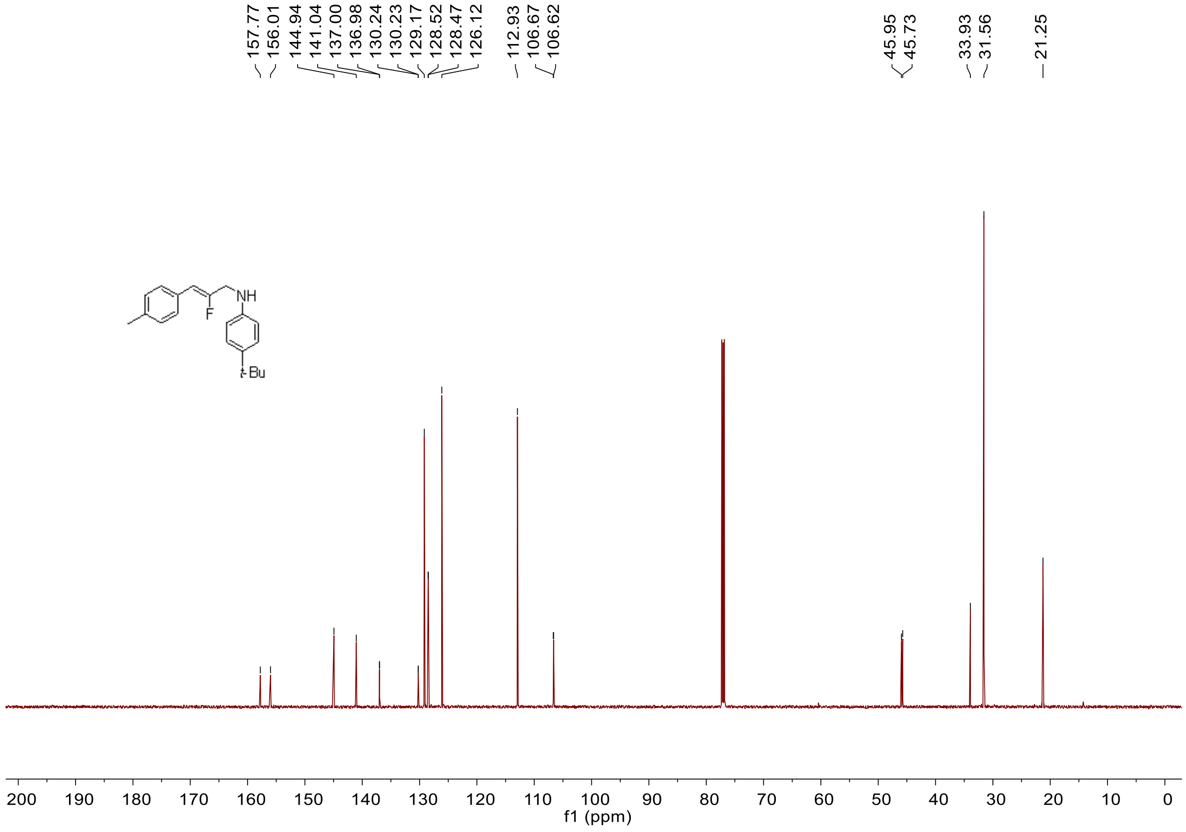

^{1}H NMR (**c16**)

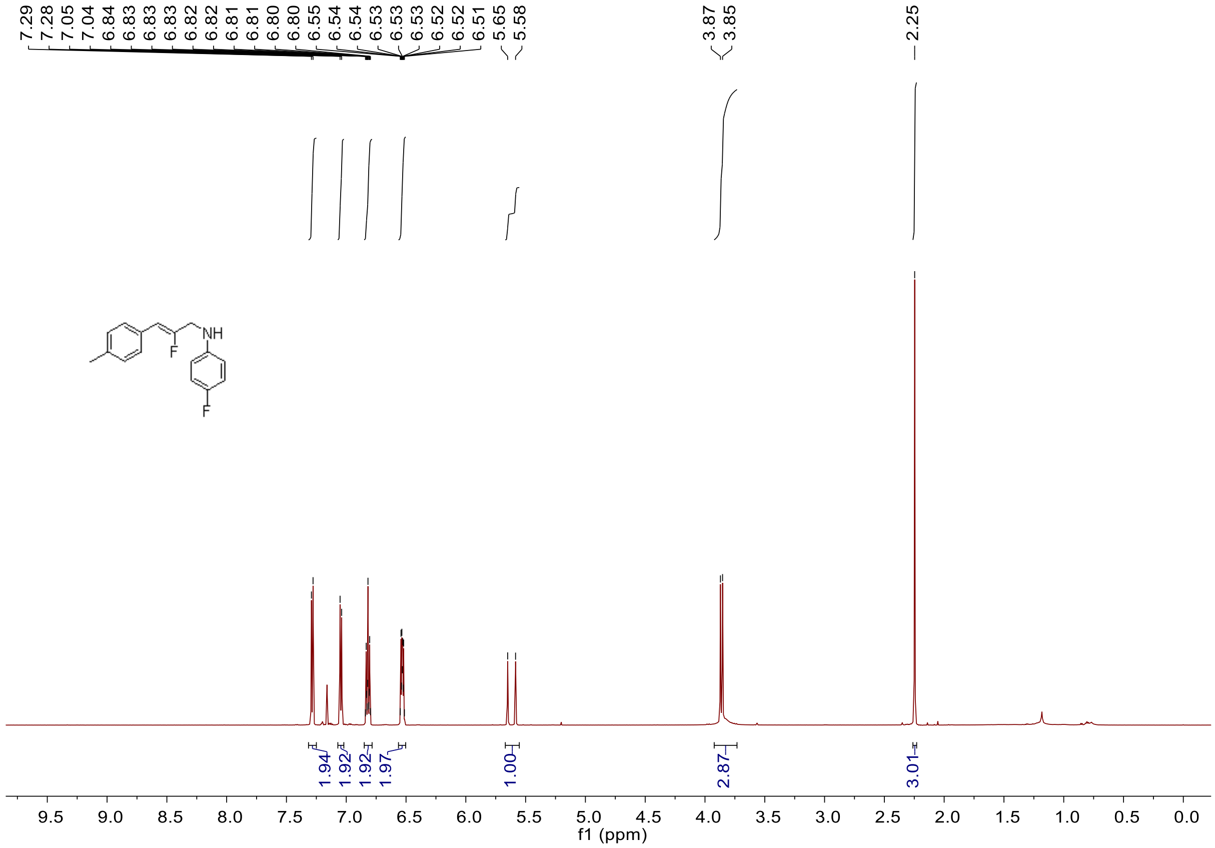

^{19}F NMR (**c16**)

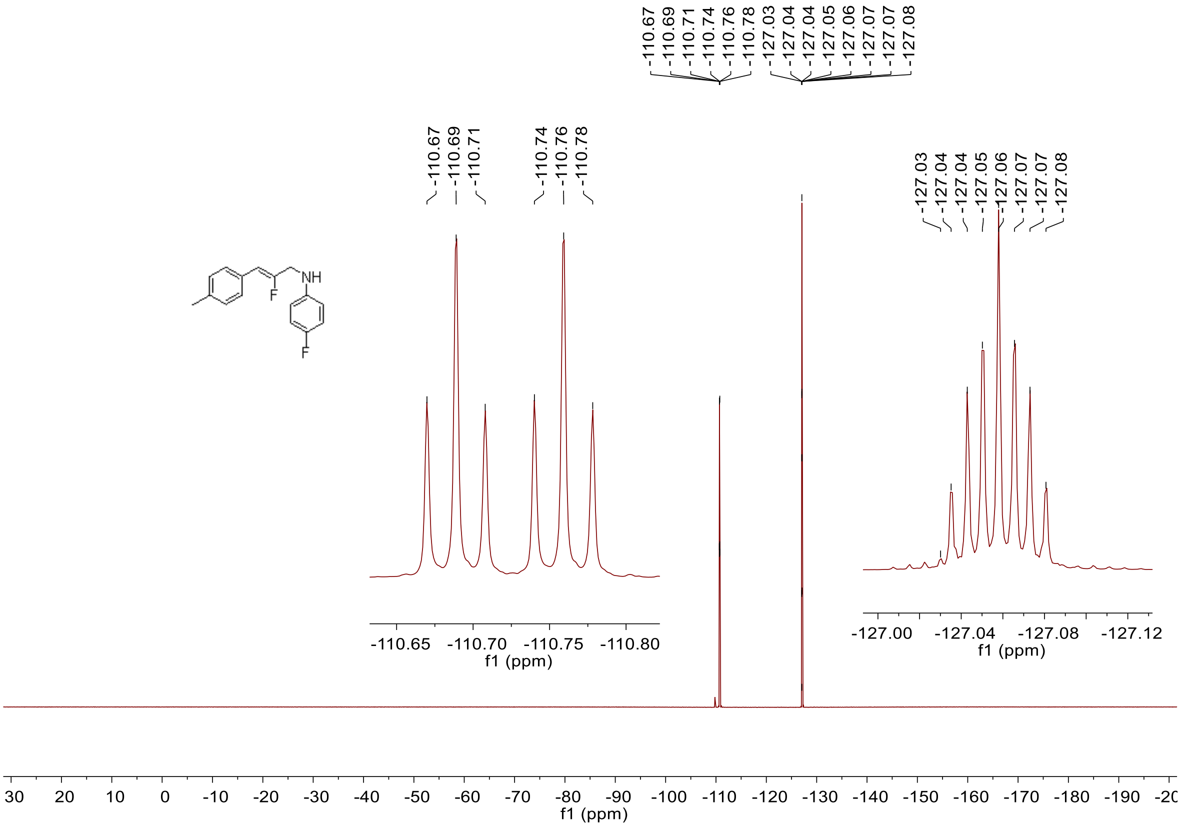

^{13}C NMR (**c16**)

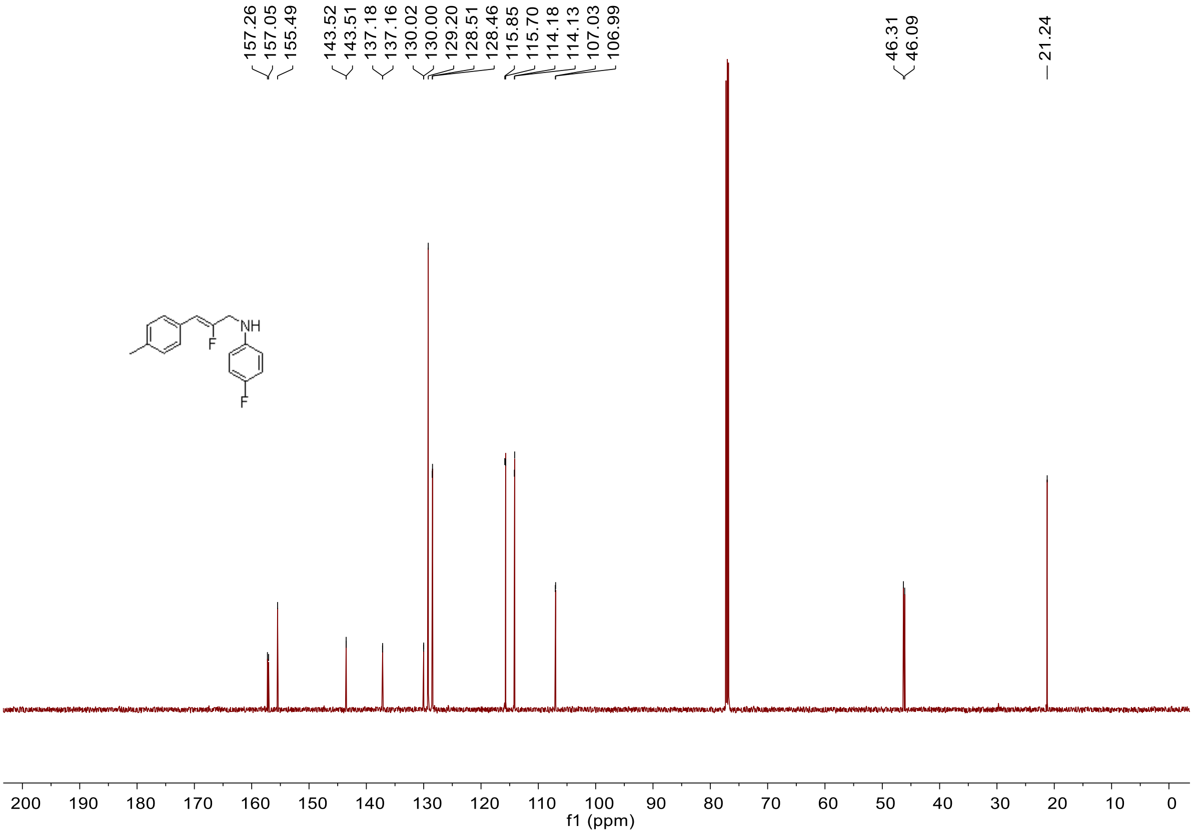

^{1}H NMR (**c17**)

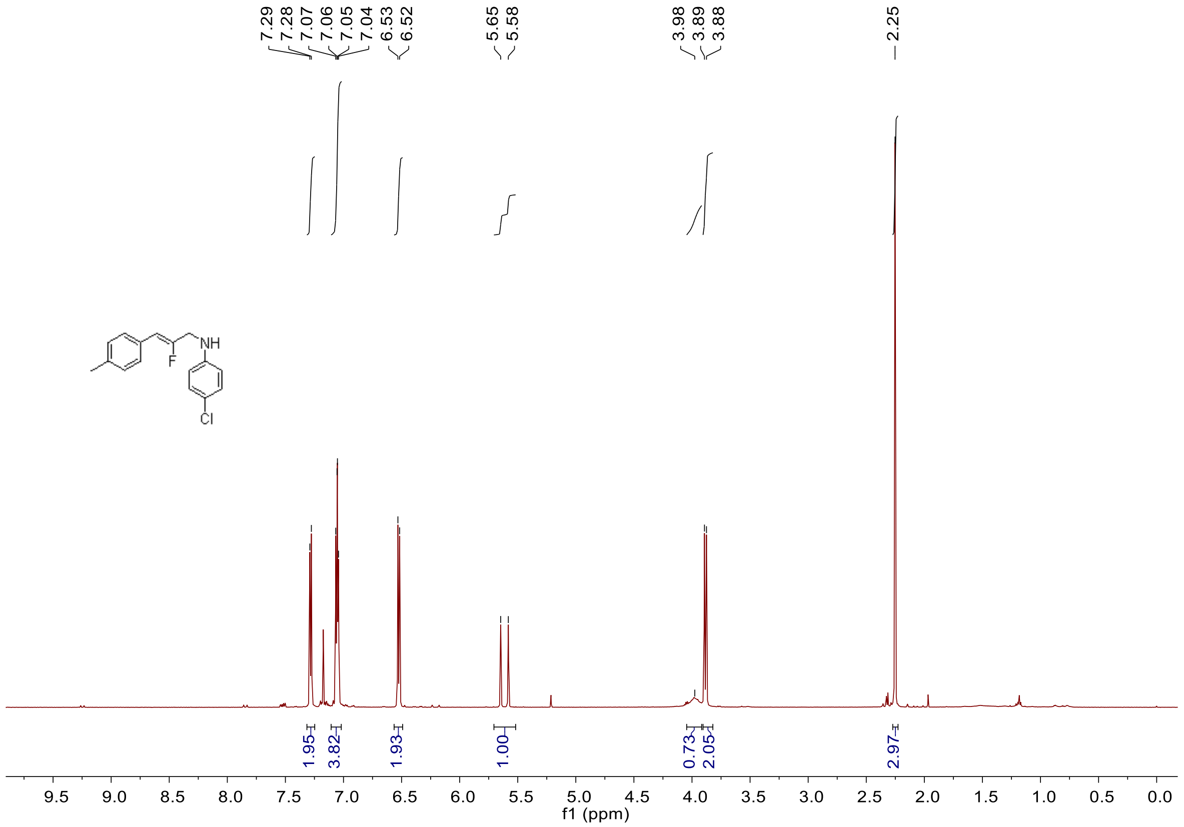

^{19}F NMR (**c17**)

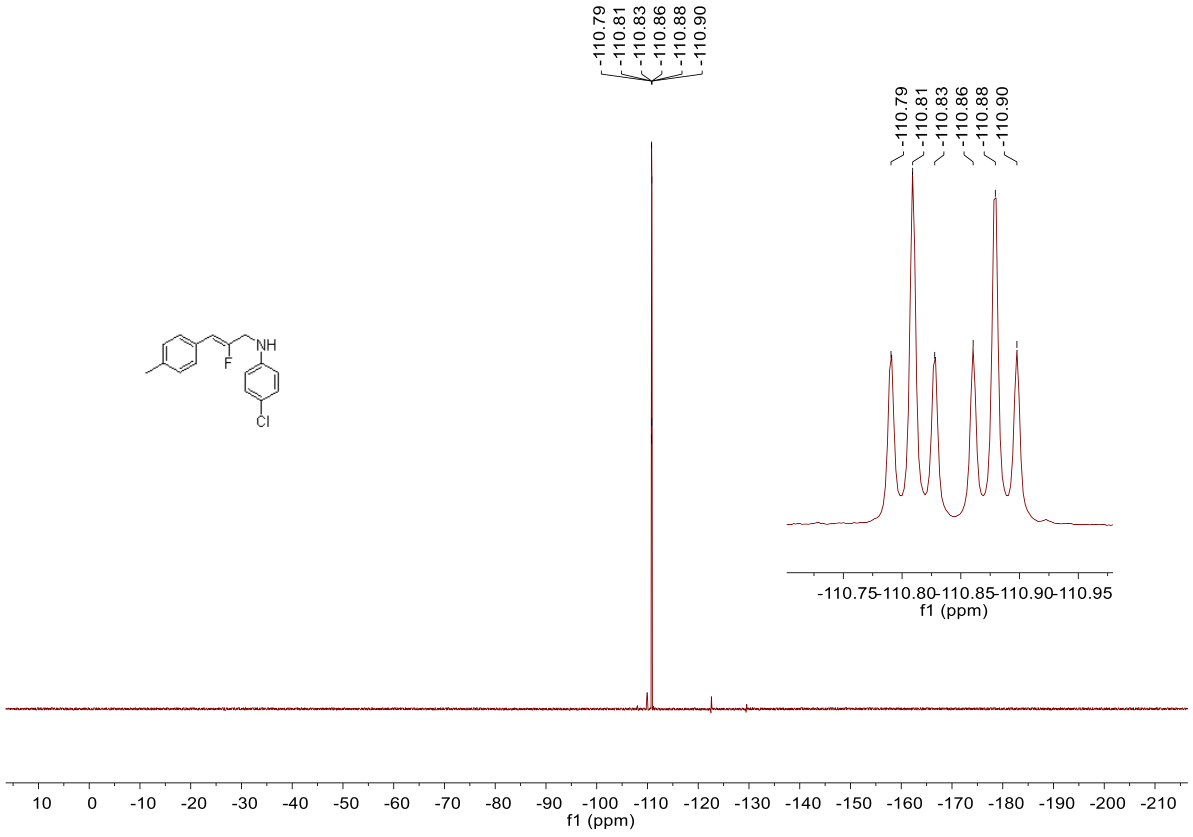

^{13}C NMR (**c17**)

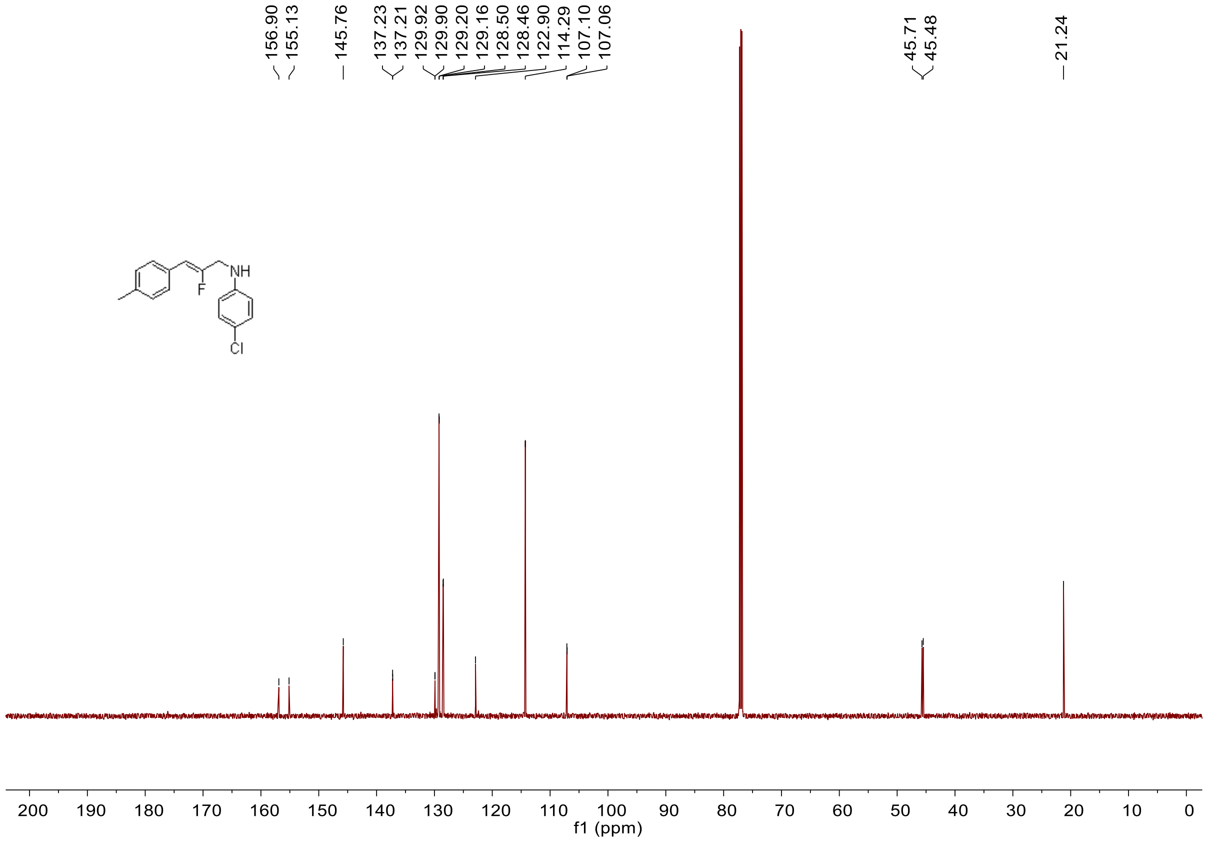

^{1}H NMR (**c18**)

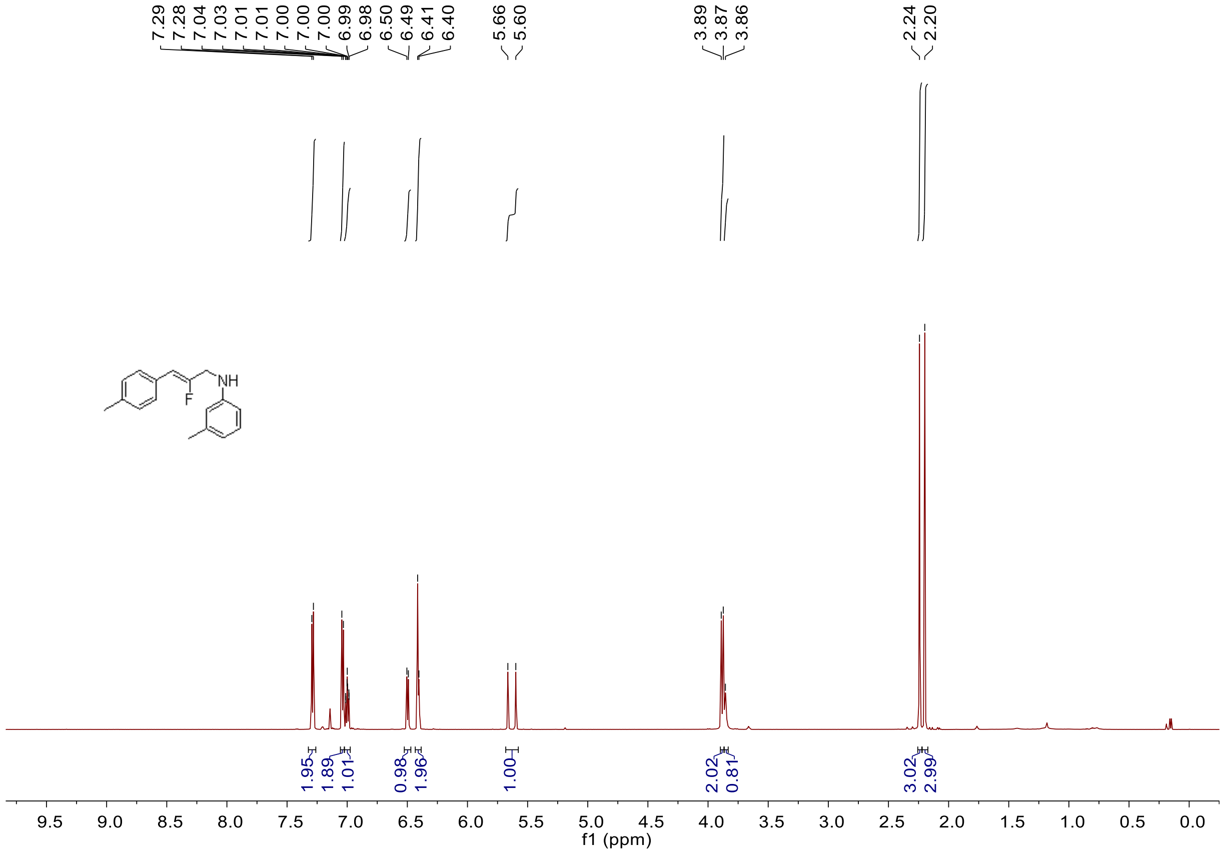

^{19}F NMR (**c18**)

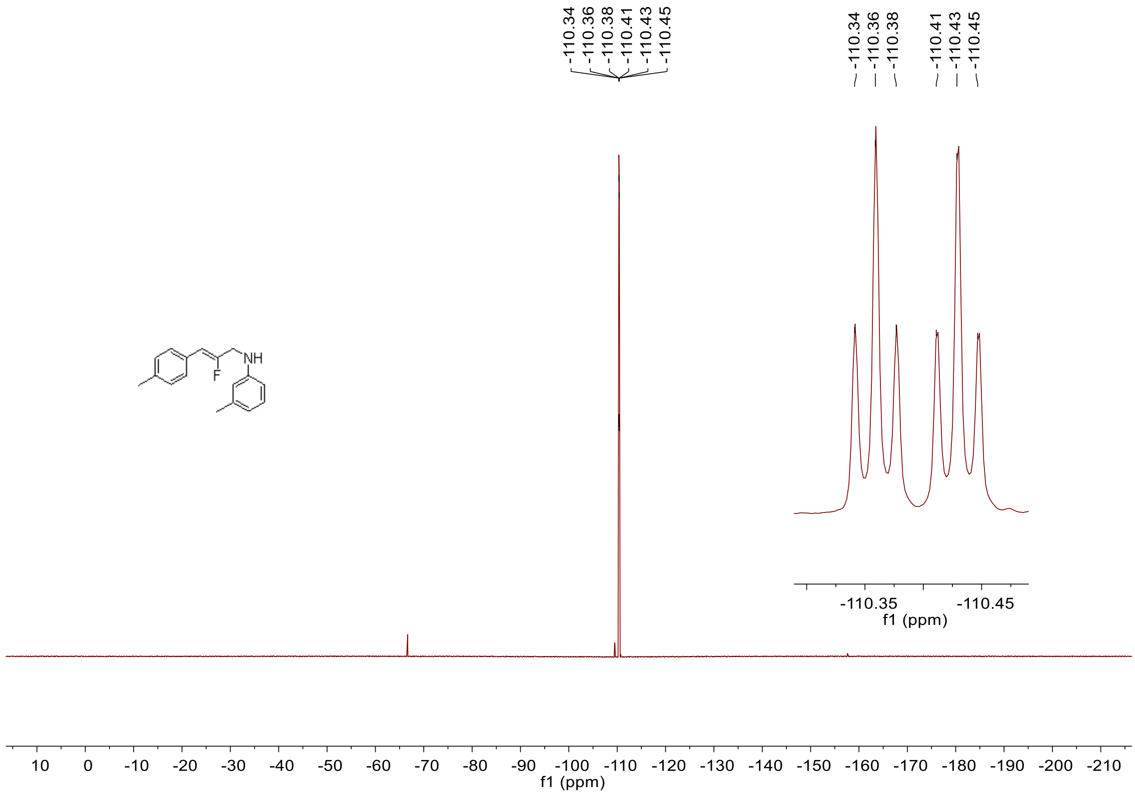

^{13}C NMR (**c18**)

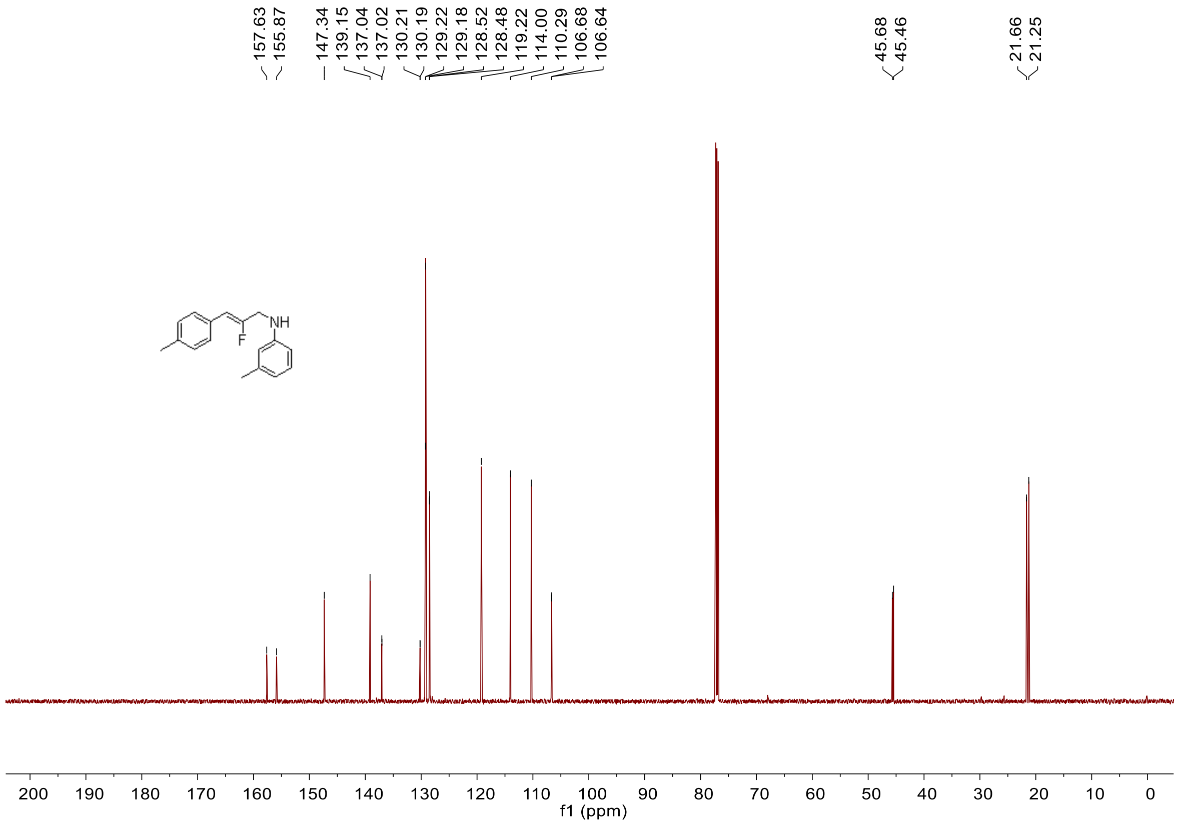

^{1}H NMR (**c19**)

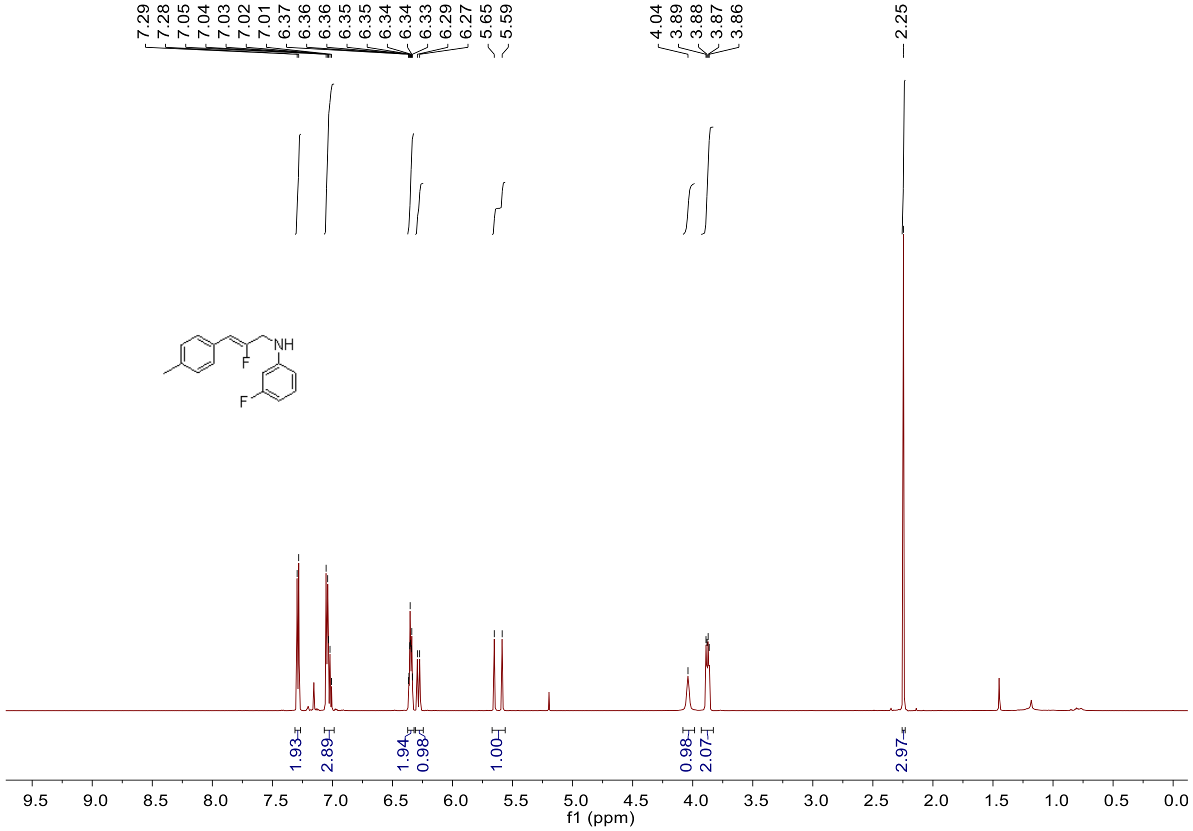

^{19}F NMR (**c19**)

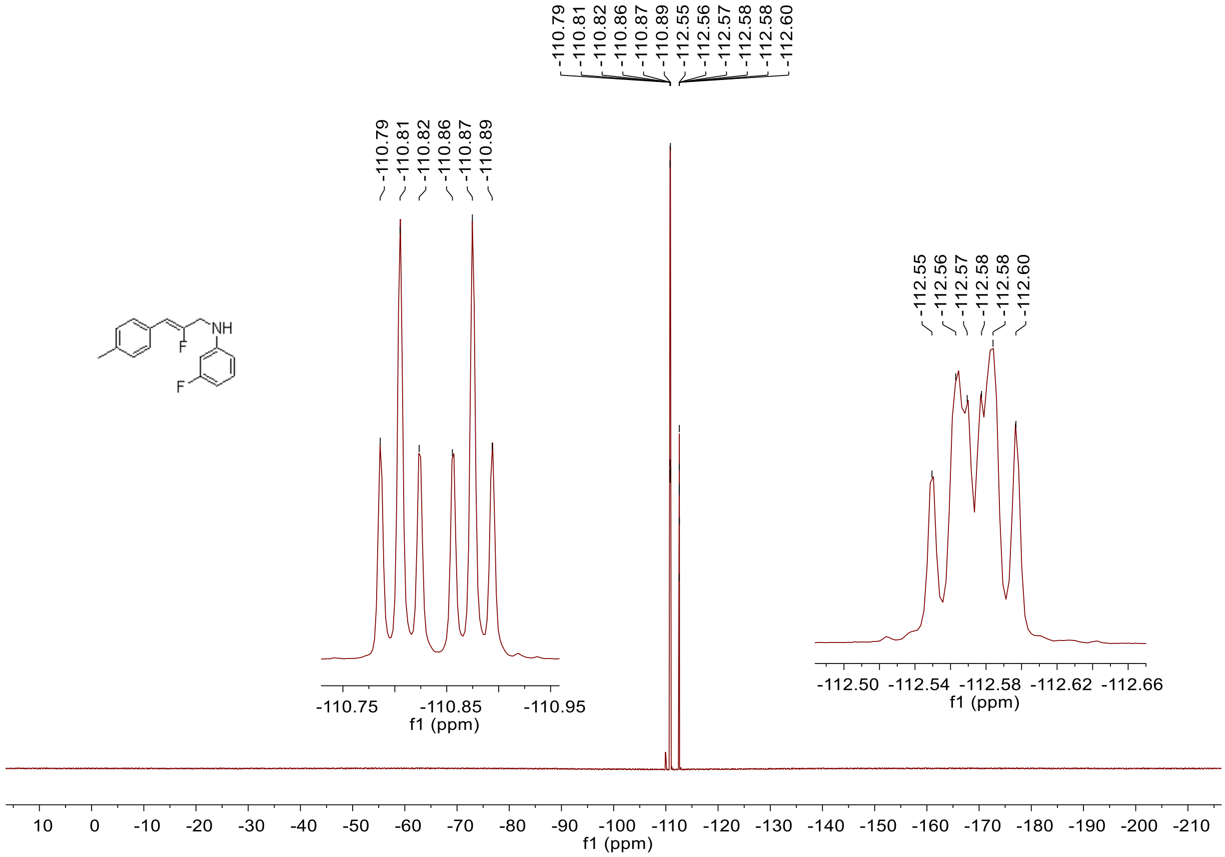

^{13}C NMR (**c19**)

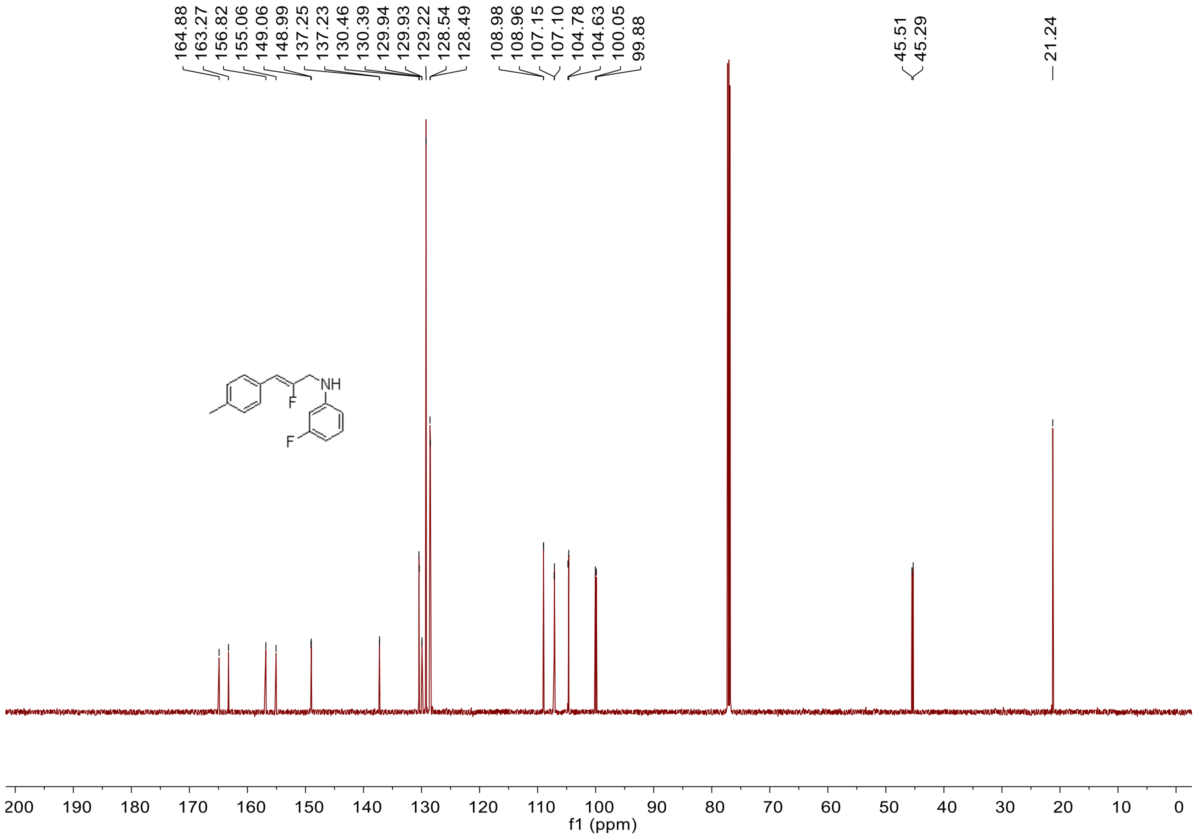

^{1}H NMR (**c20**)

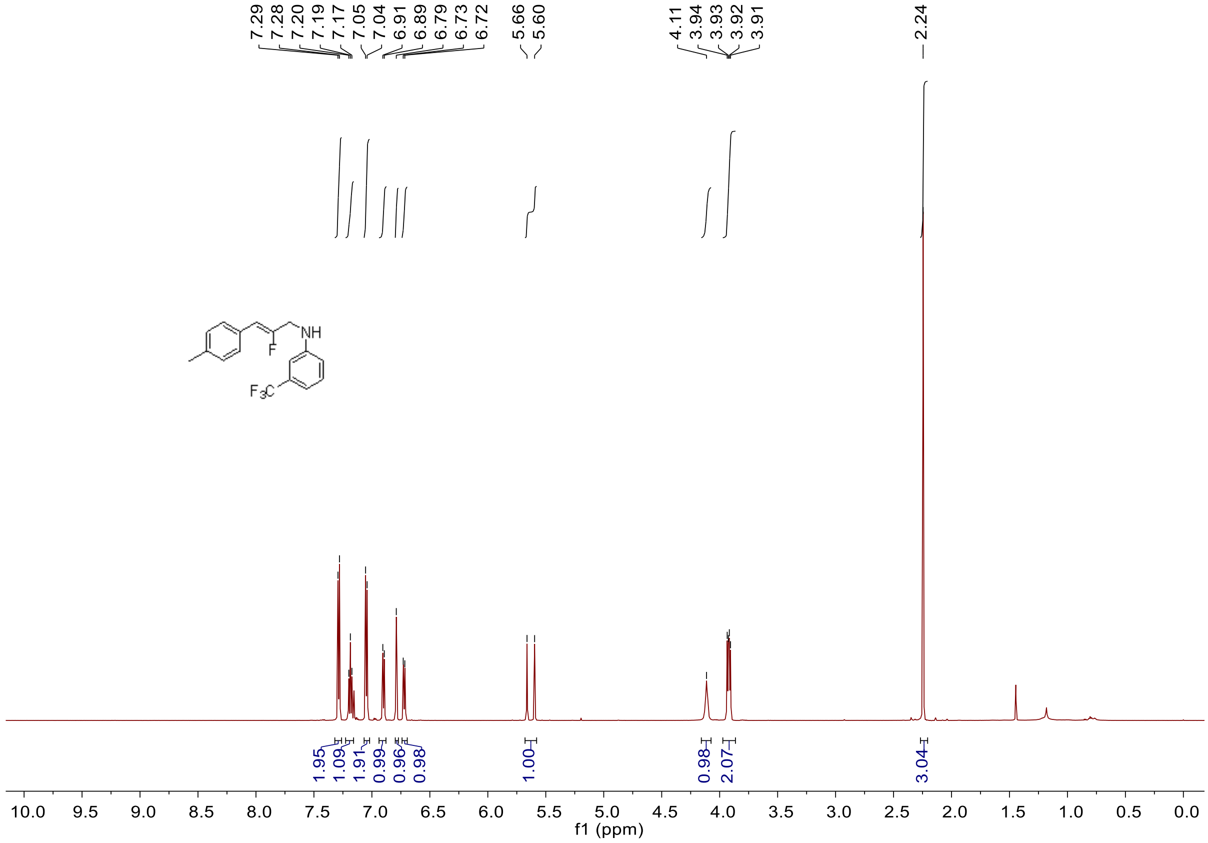

^{19}F NMR (**c20**)

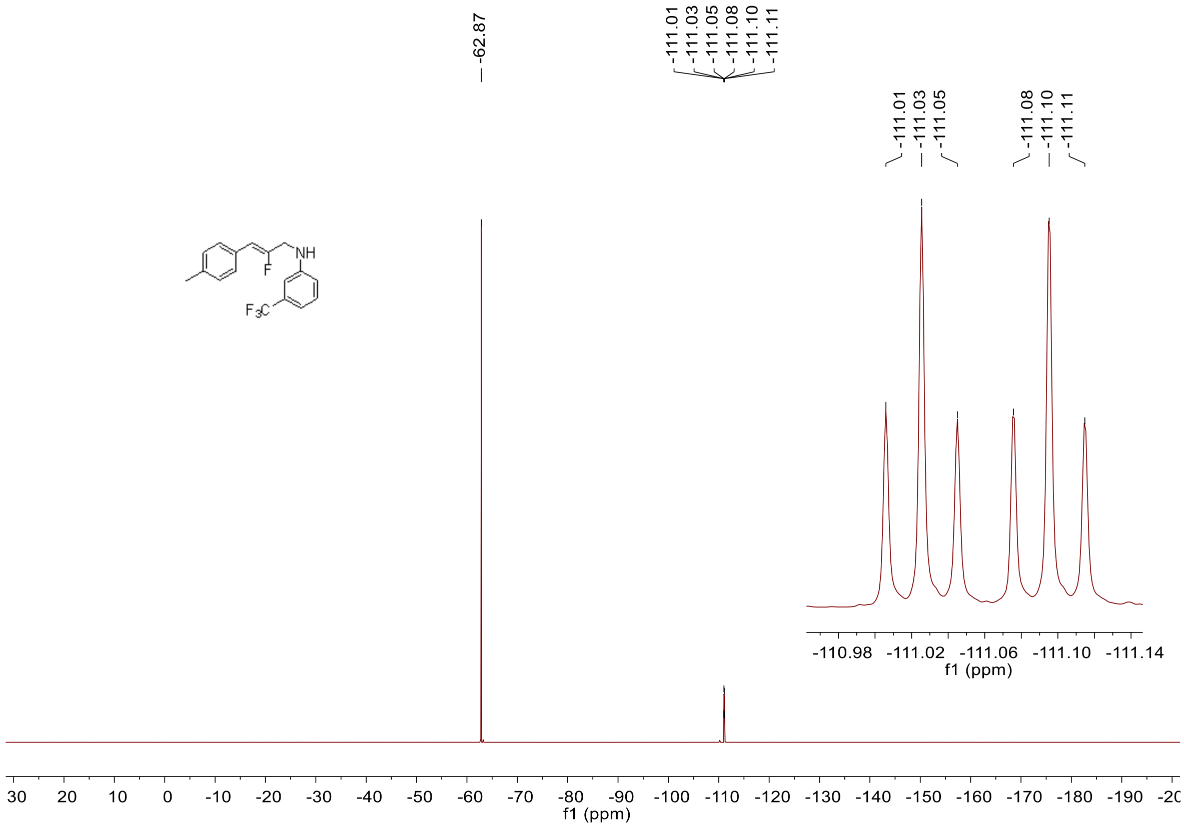

^{13}C NMR (**c20**)

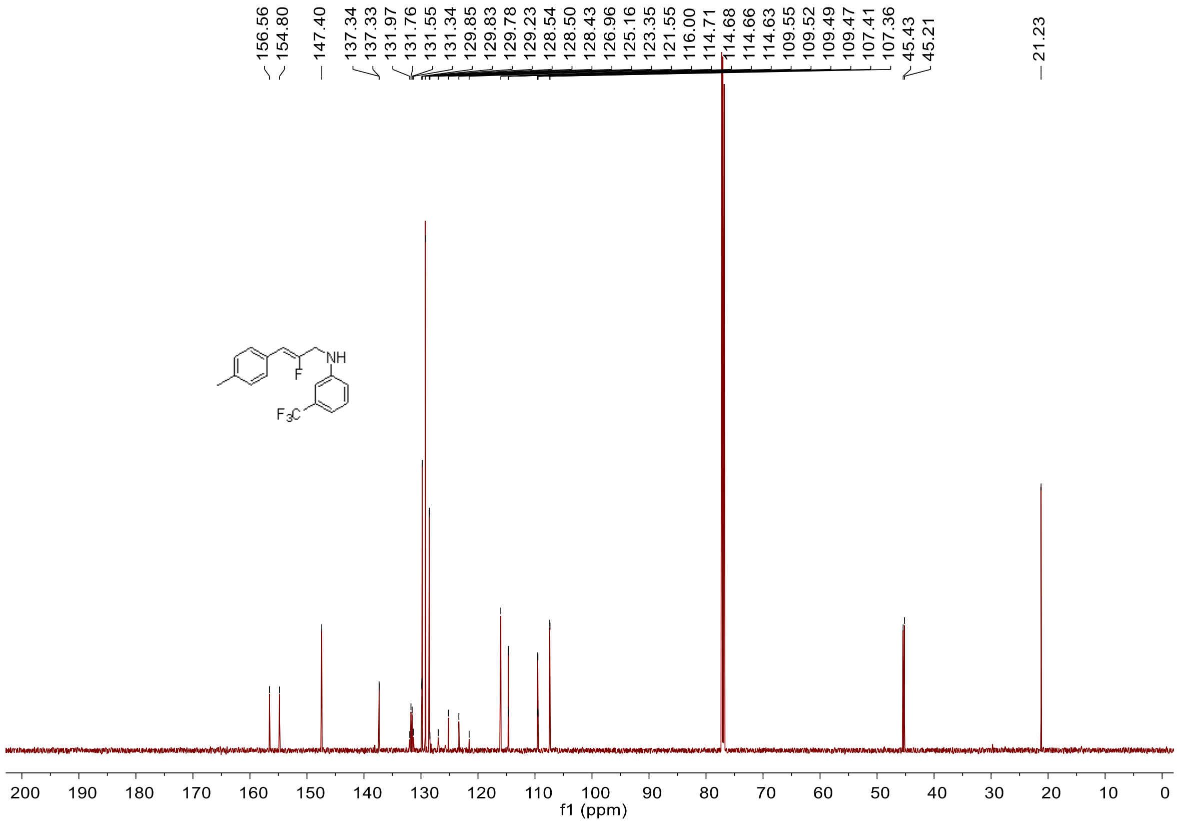

^{1}H NMR (**c21**)

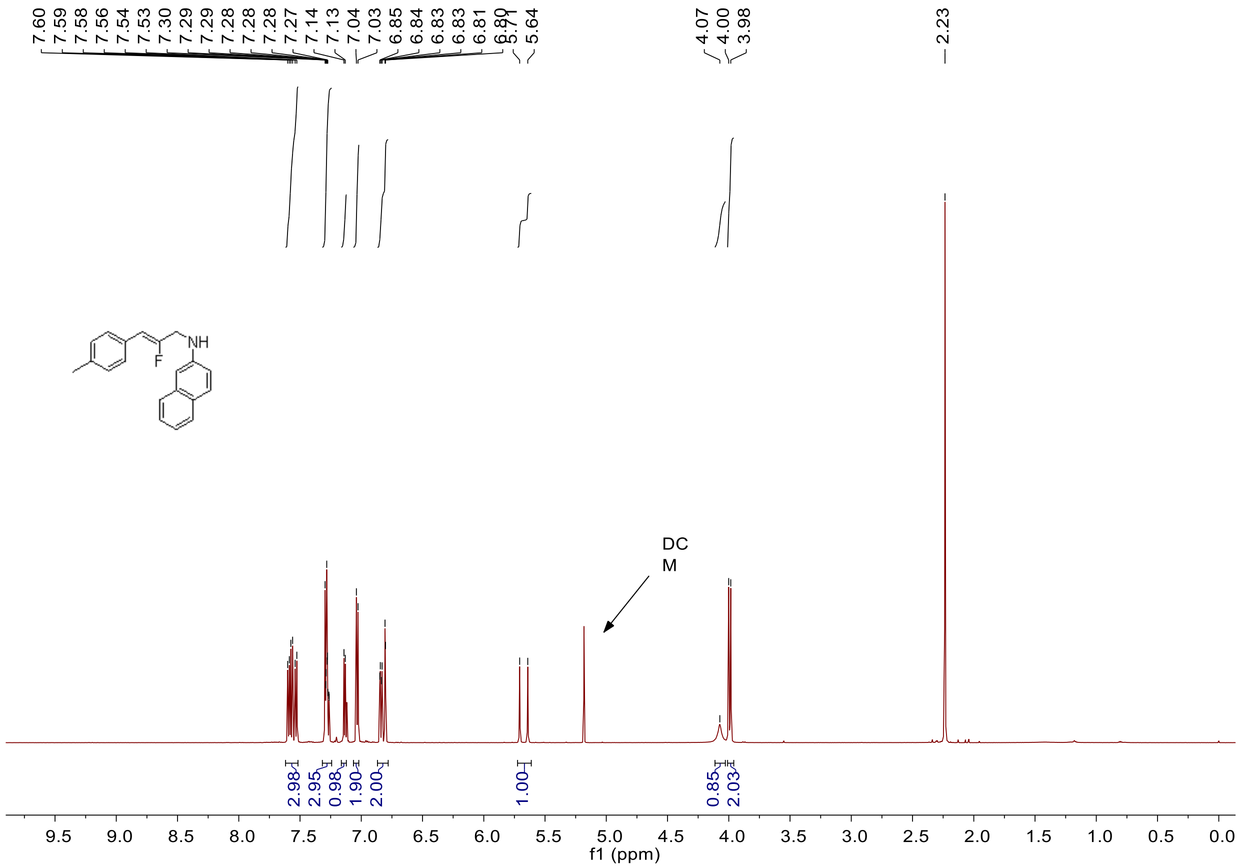

^{19}F NMR (**c21**)

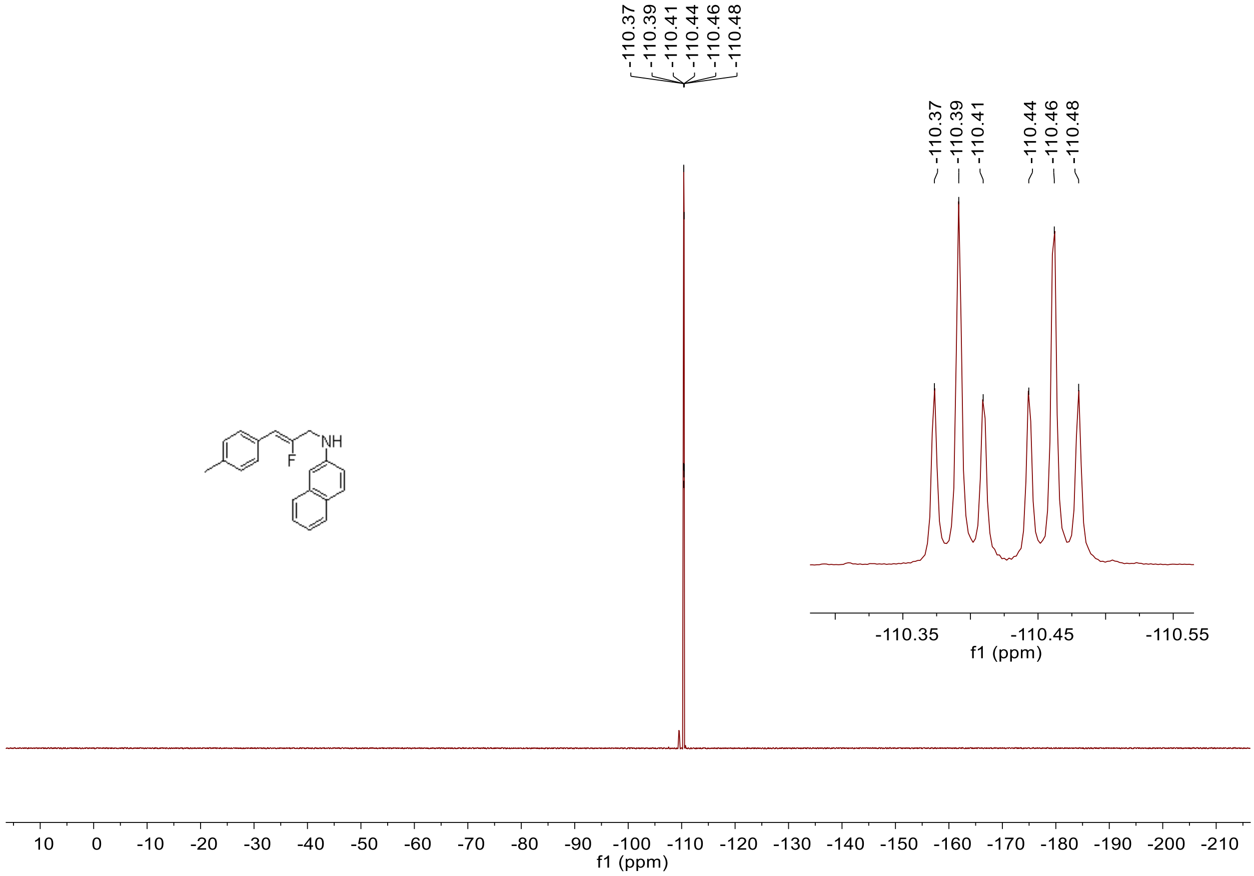

^{13}C NMR (**c21**)

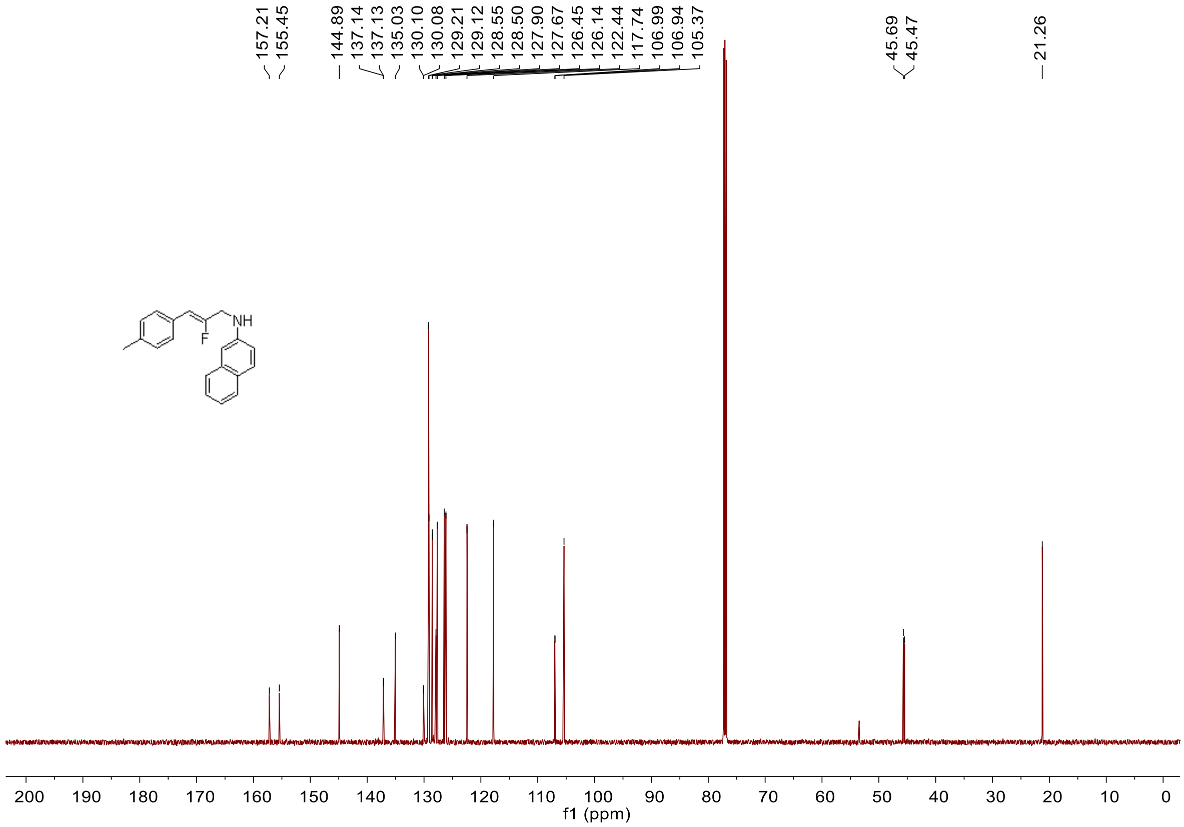

^{1}H NMR (**c22**)

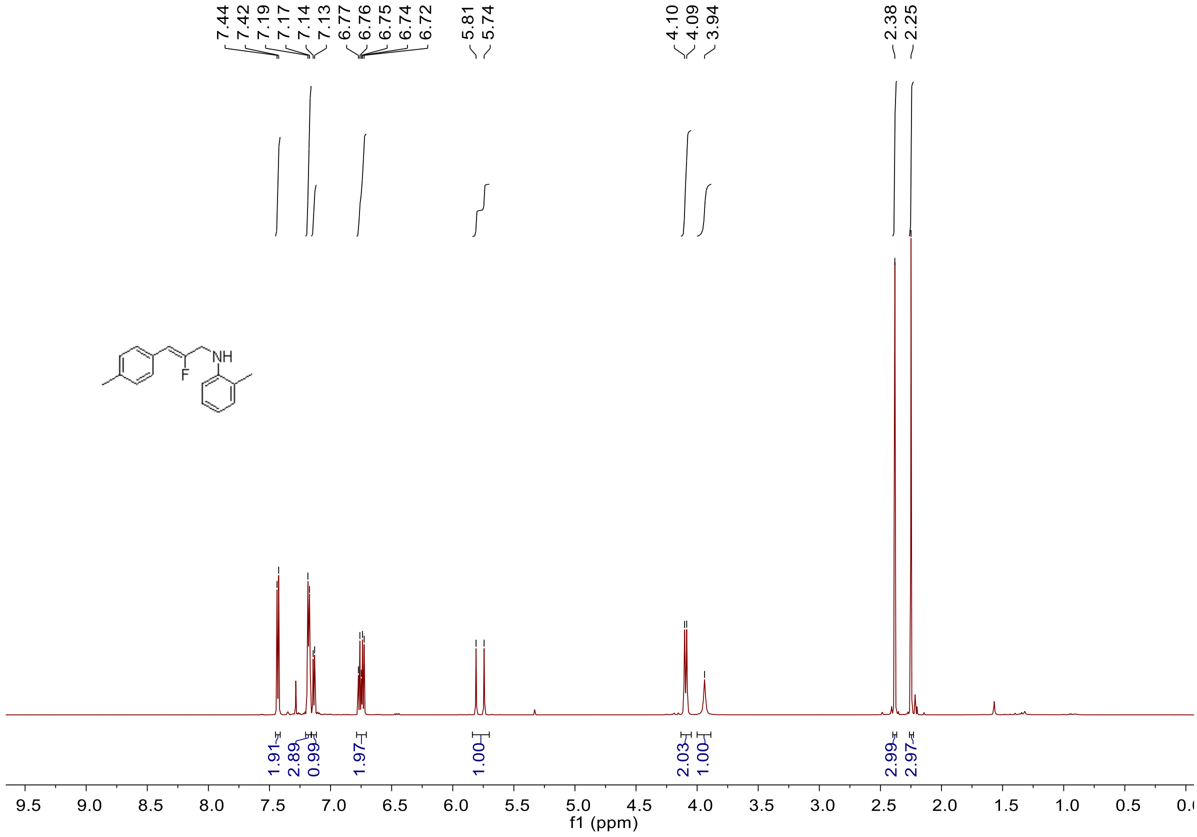

^{19}F NMR (**c22**)

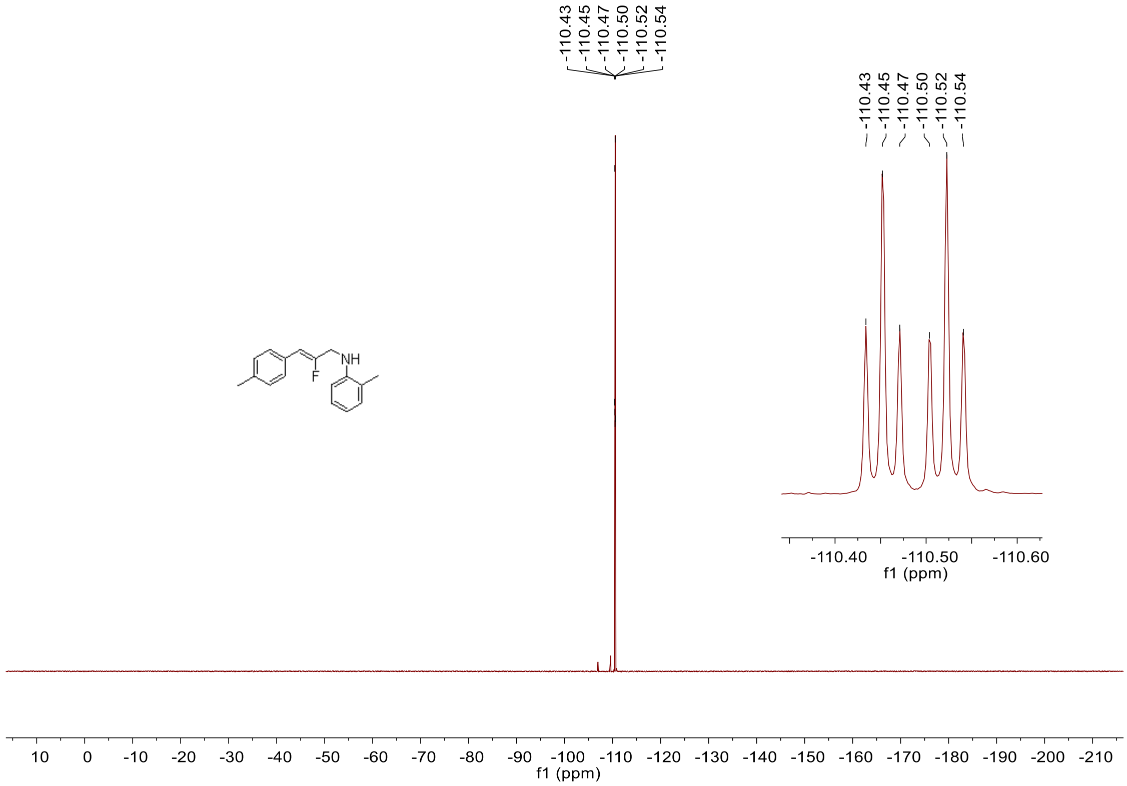

^{13}C NMR (**c22**)

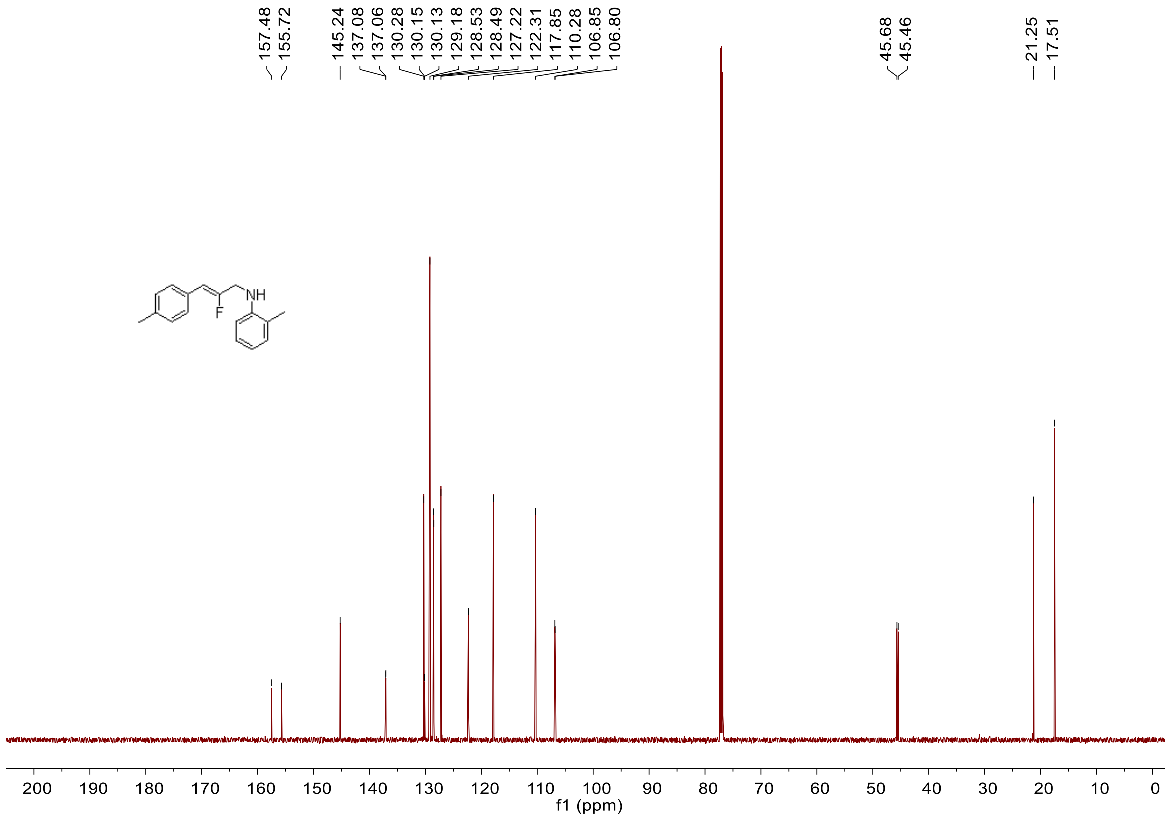

^{1}H NMR (**c23**)

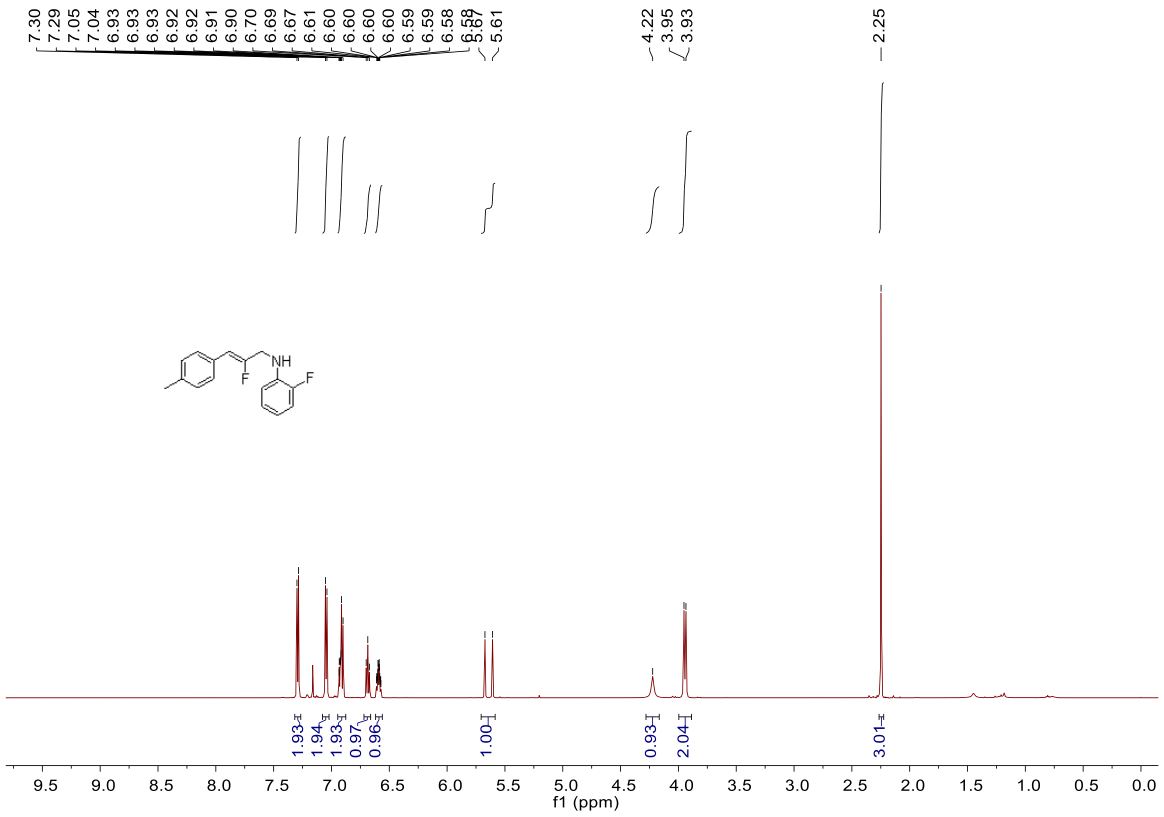

^{19}F NMR (**c23**)

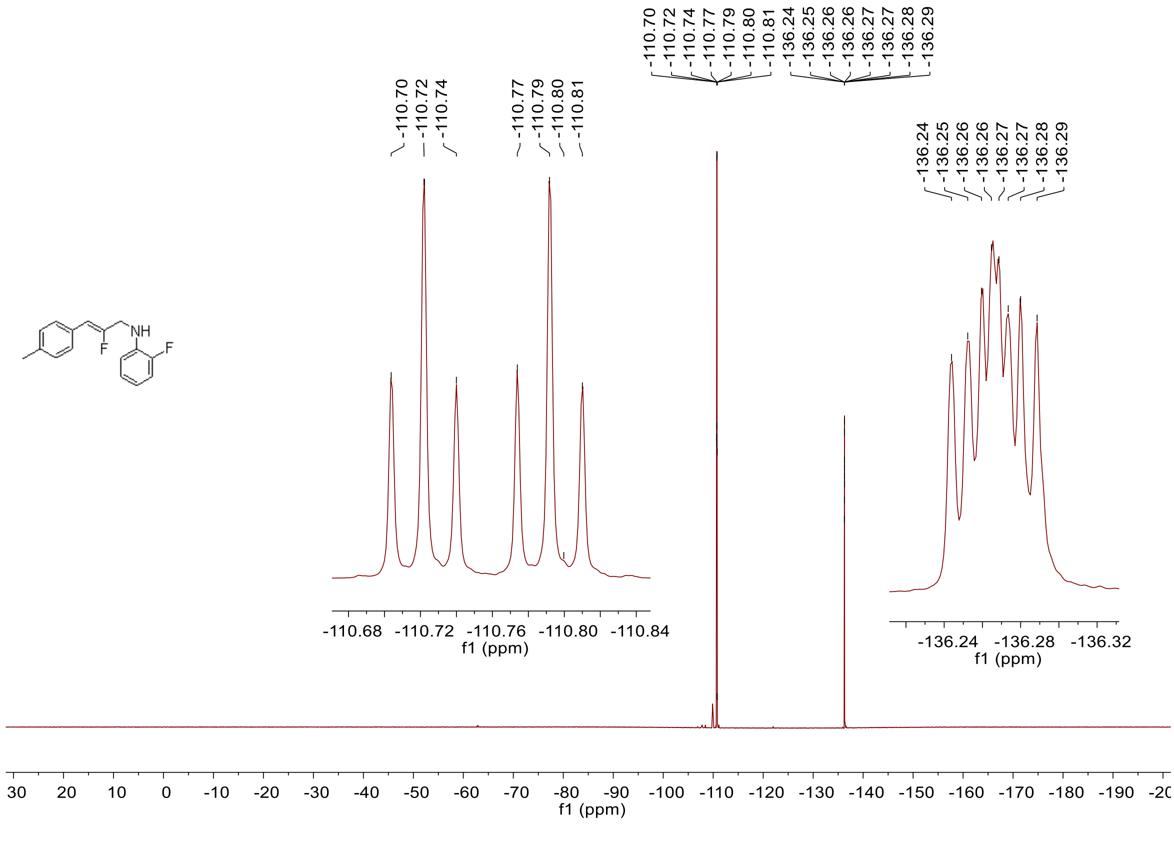

^{13}C NMR (**c23**)

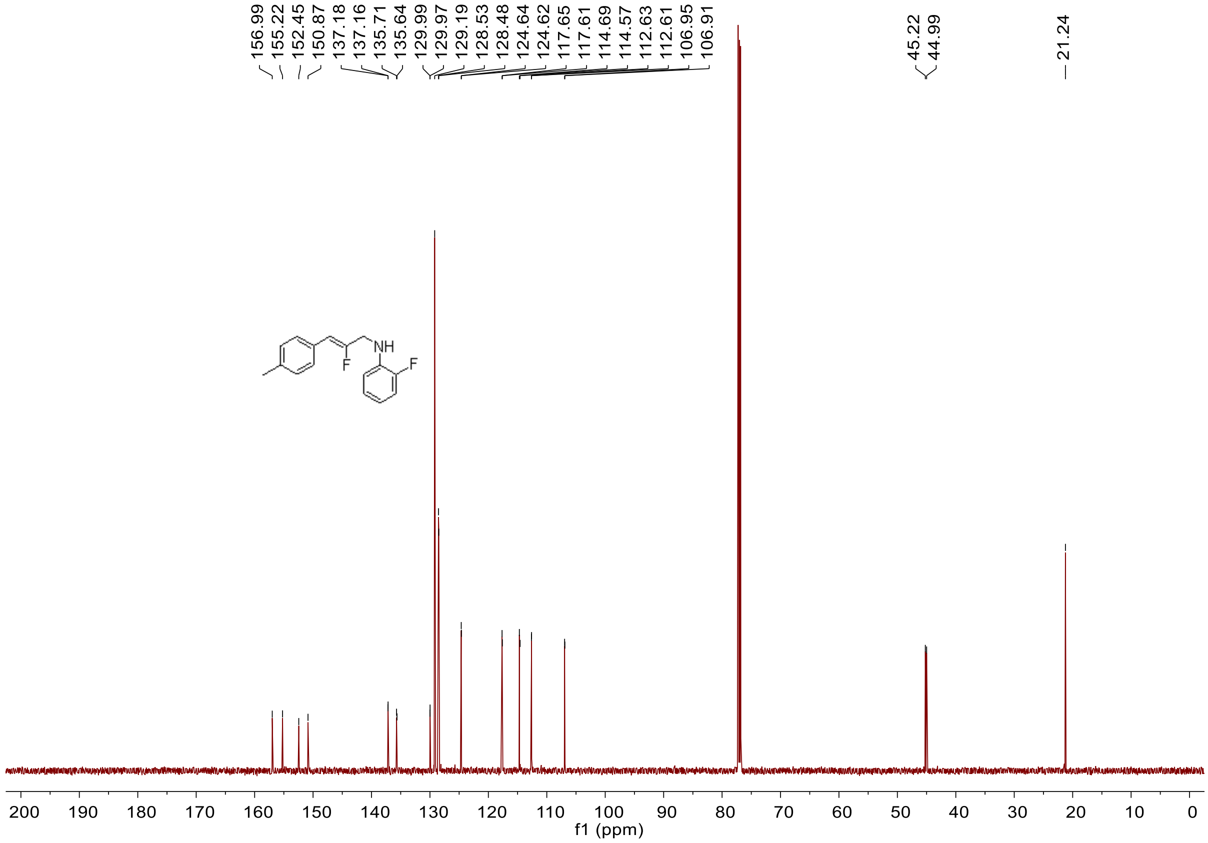

^{1}H NMR (**c24**)

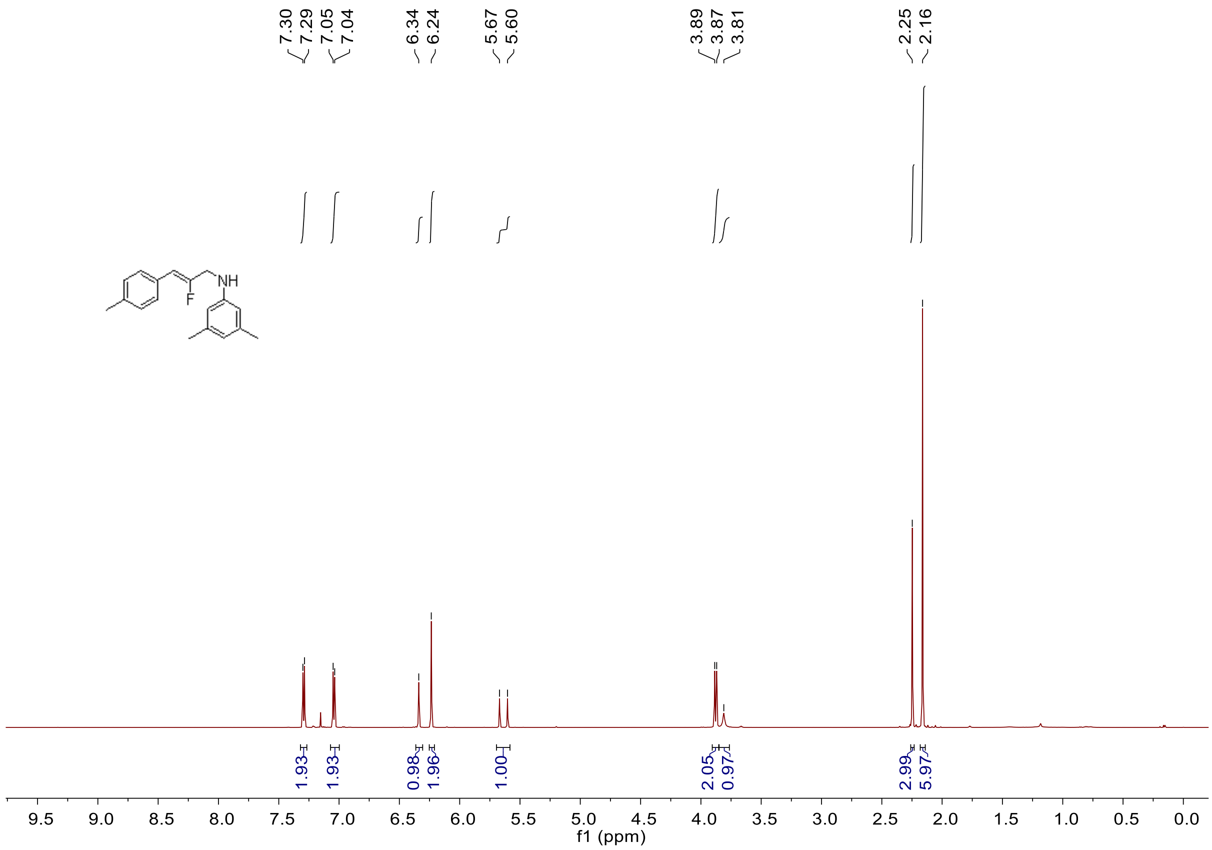

^{19}F NMR (**c24**)

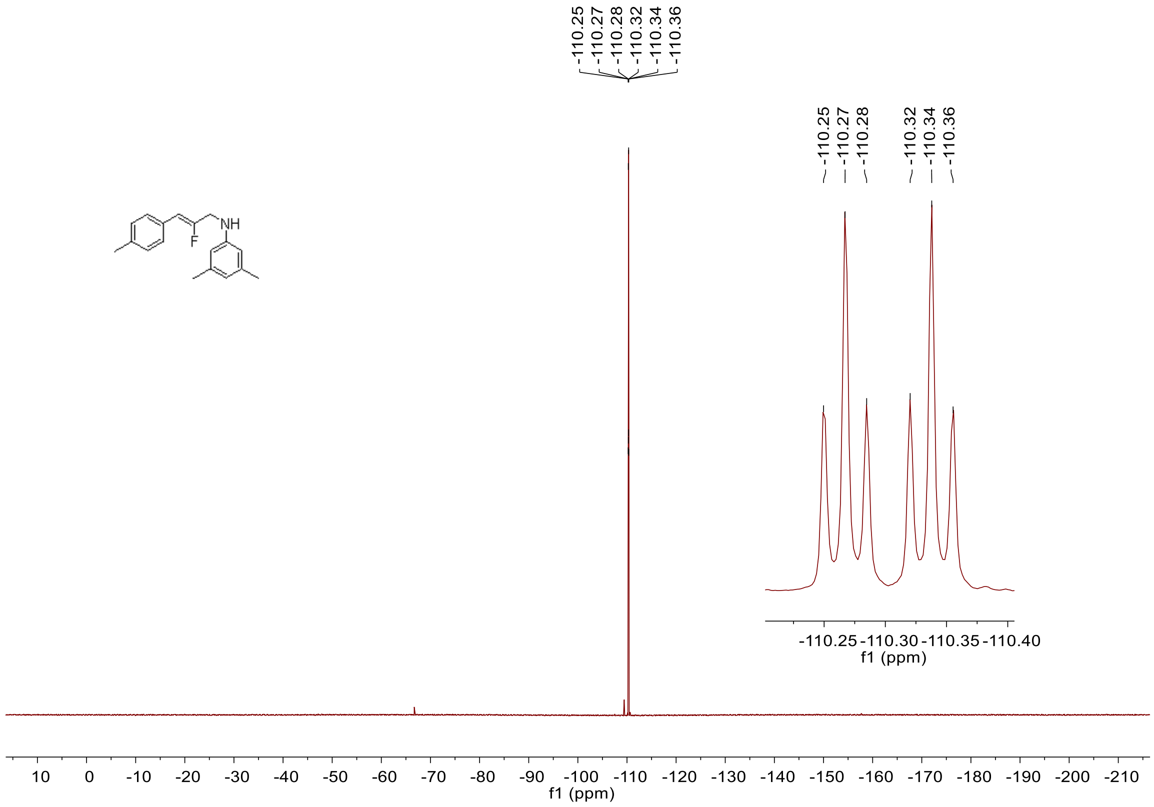

^{13}C NMR (**c24**)

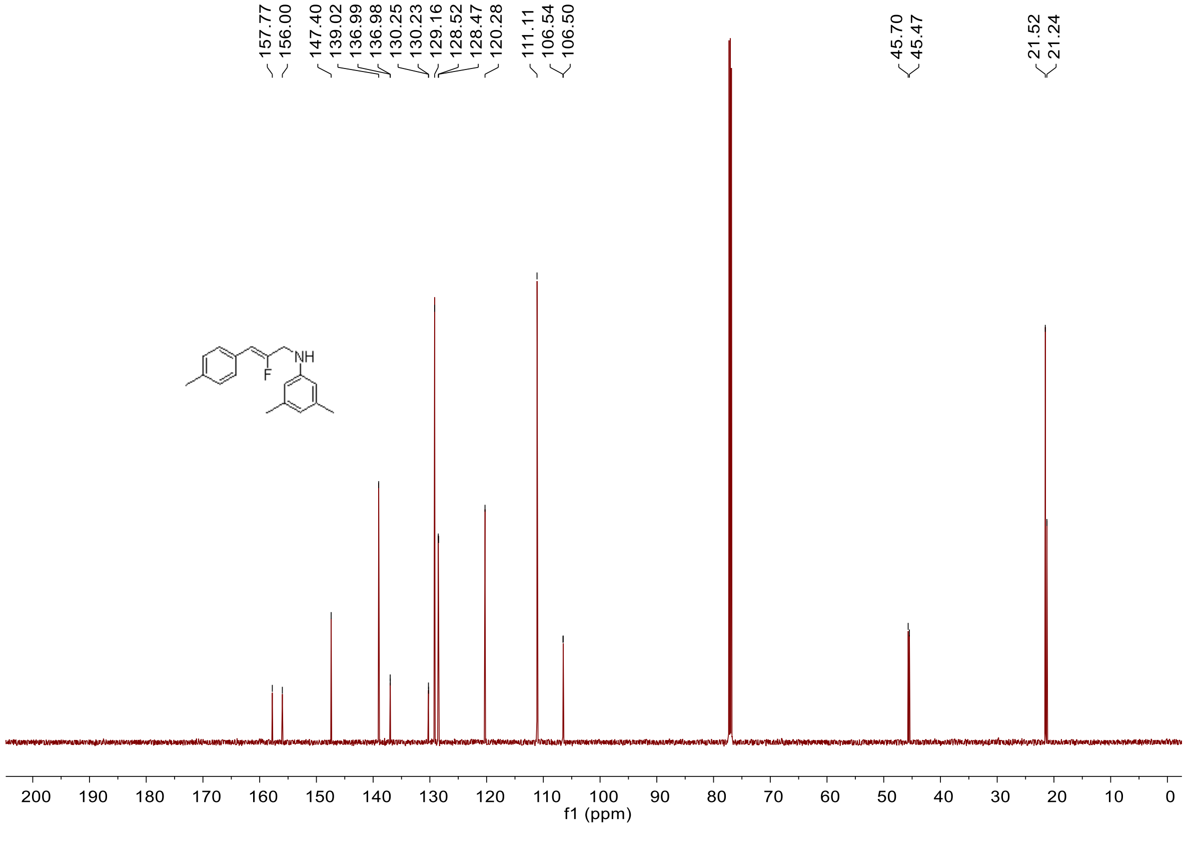

^{1}H NMR (**c25**)

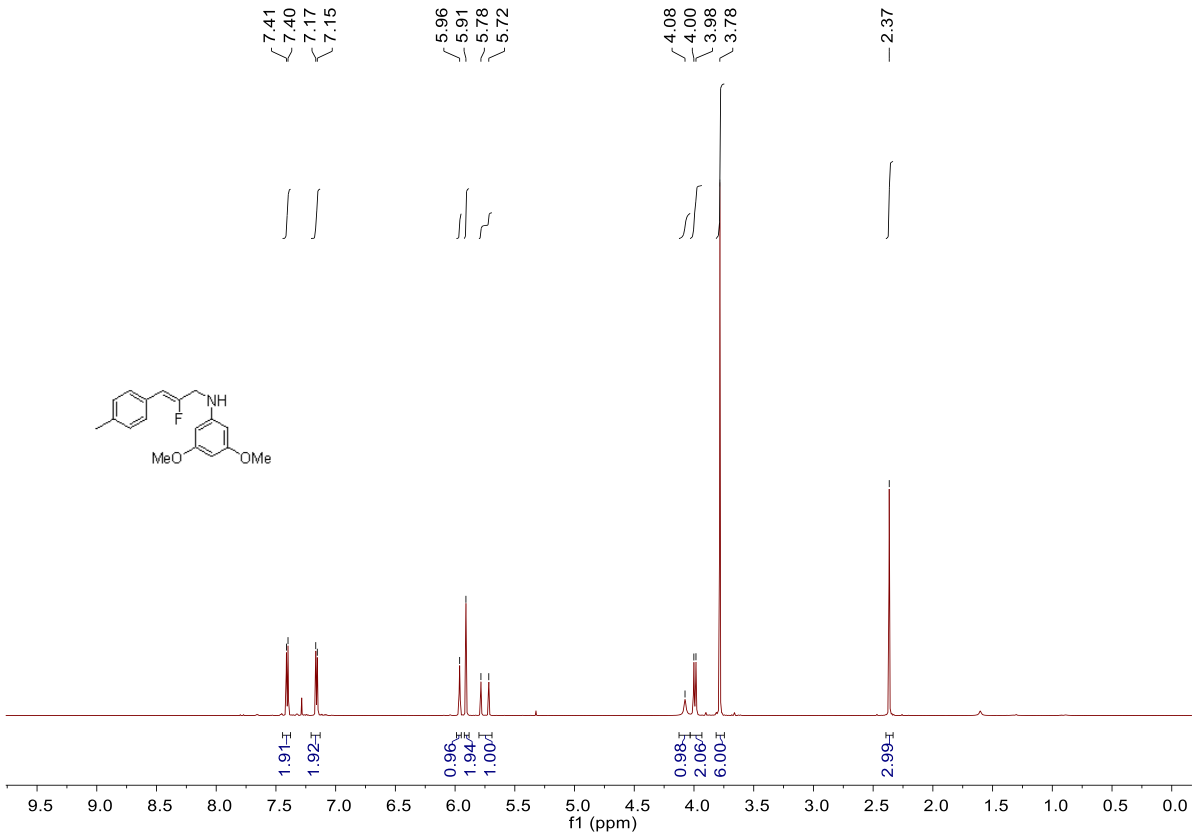

^{19}F NMR (**c25**)

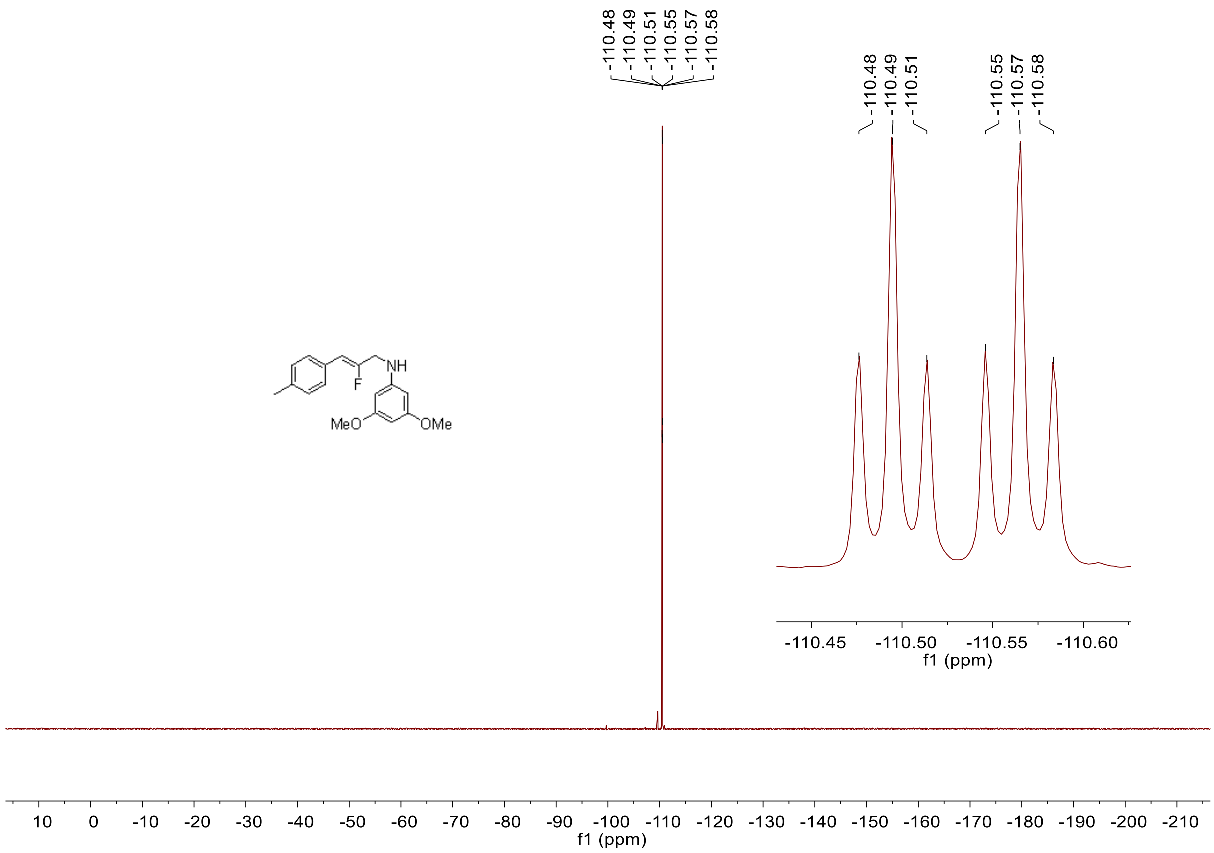

^{13}C NMR (**c25**)

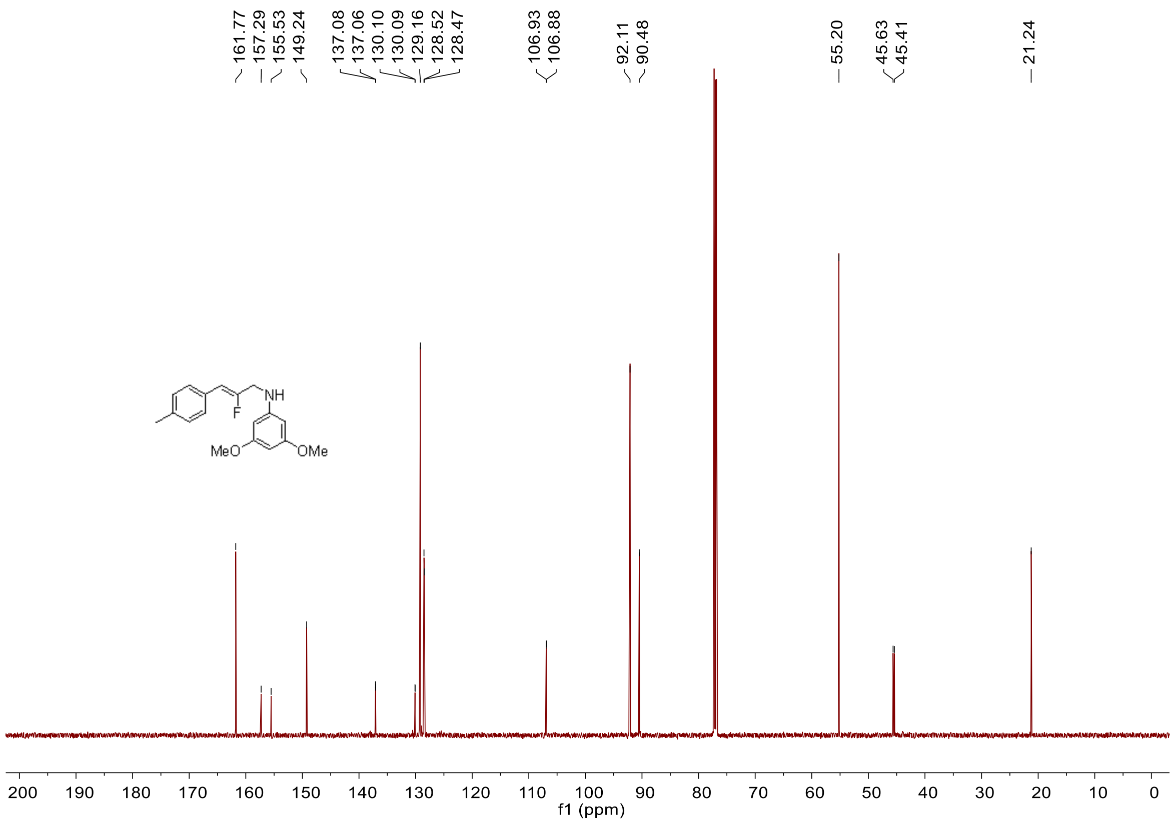

^{1}H NMR (**c26**)

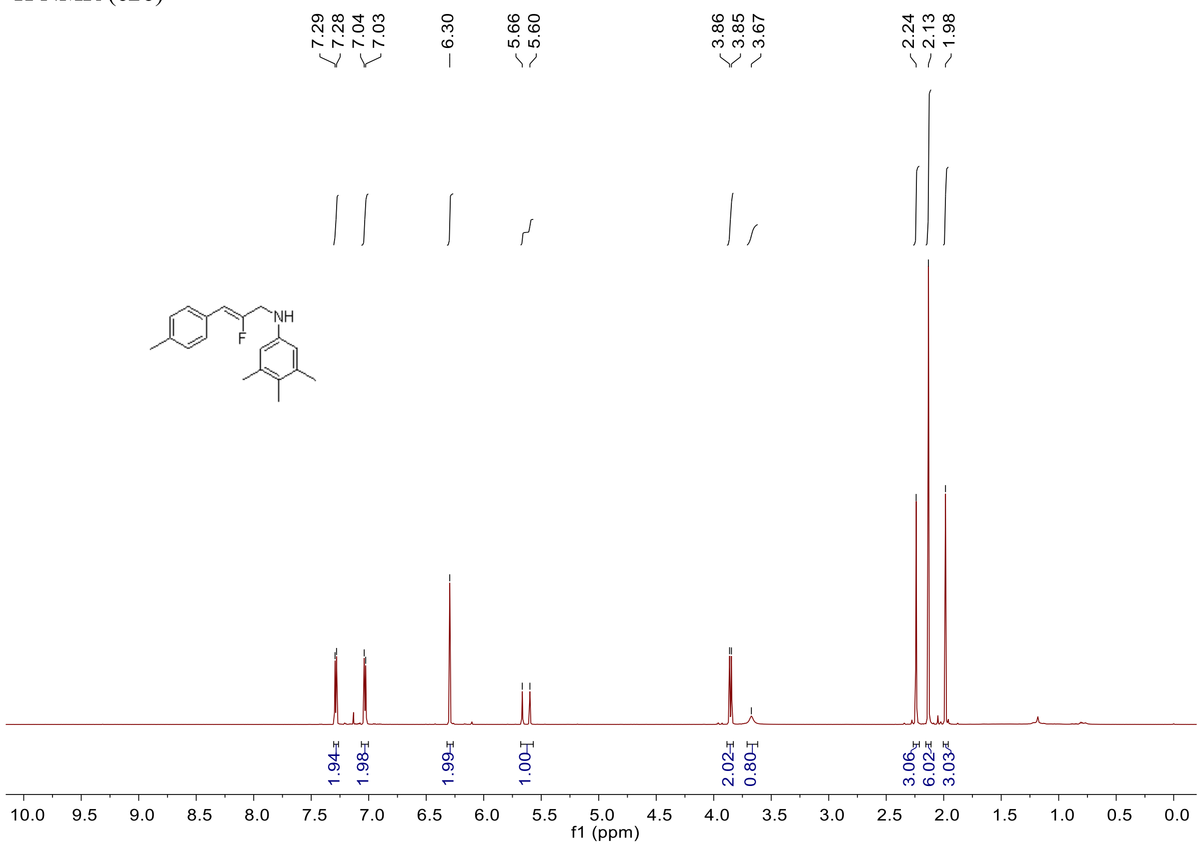

^{19}F NMR (**c26**)

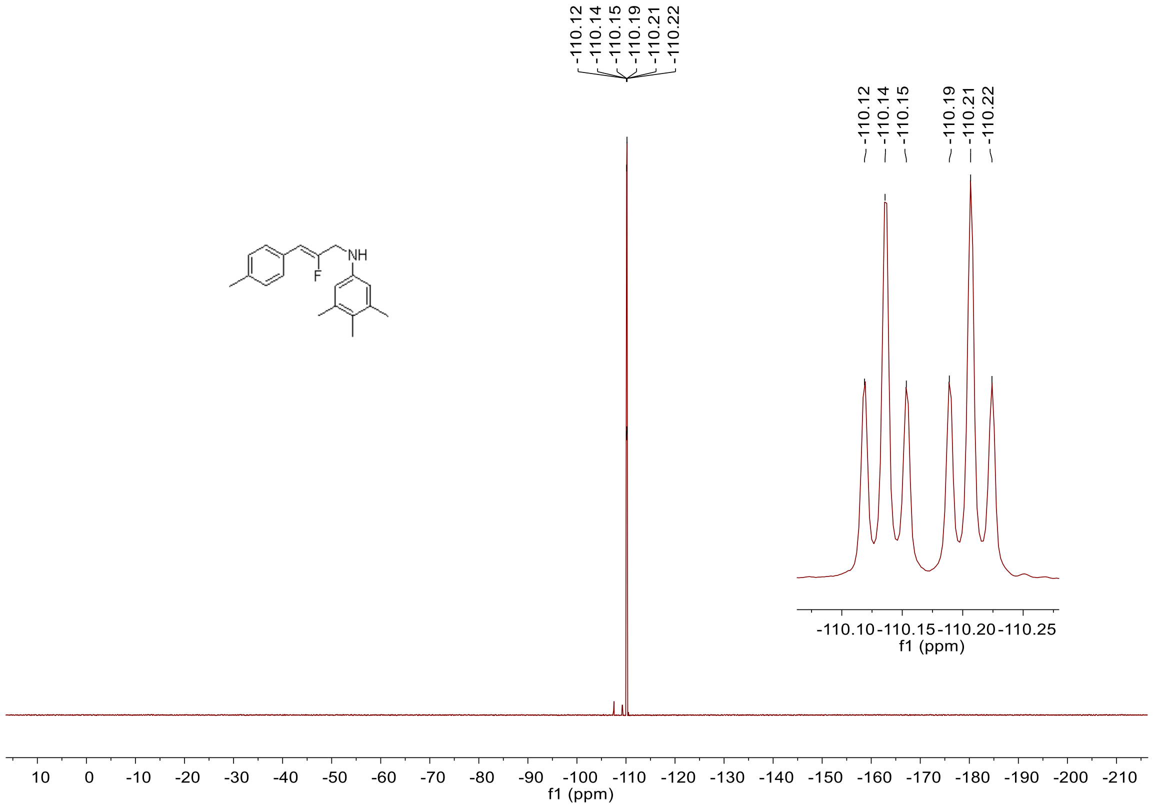

^{13}C NMR (**c26**)

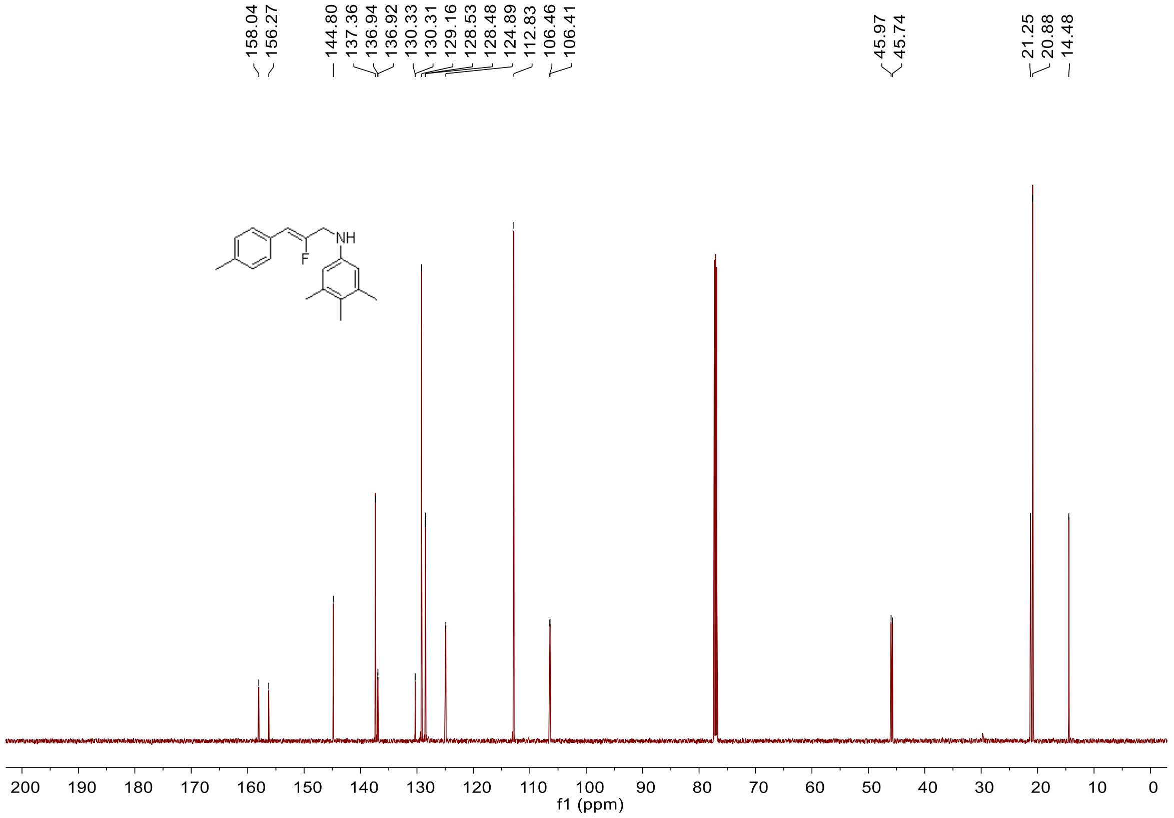

^{1}H NMR (**c27**)

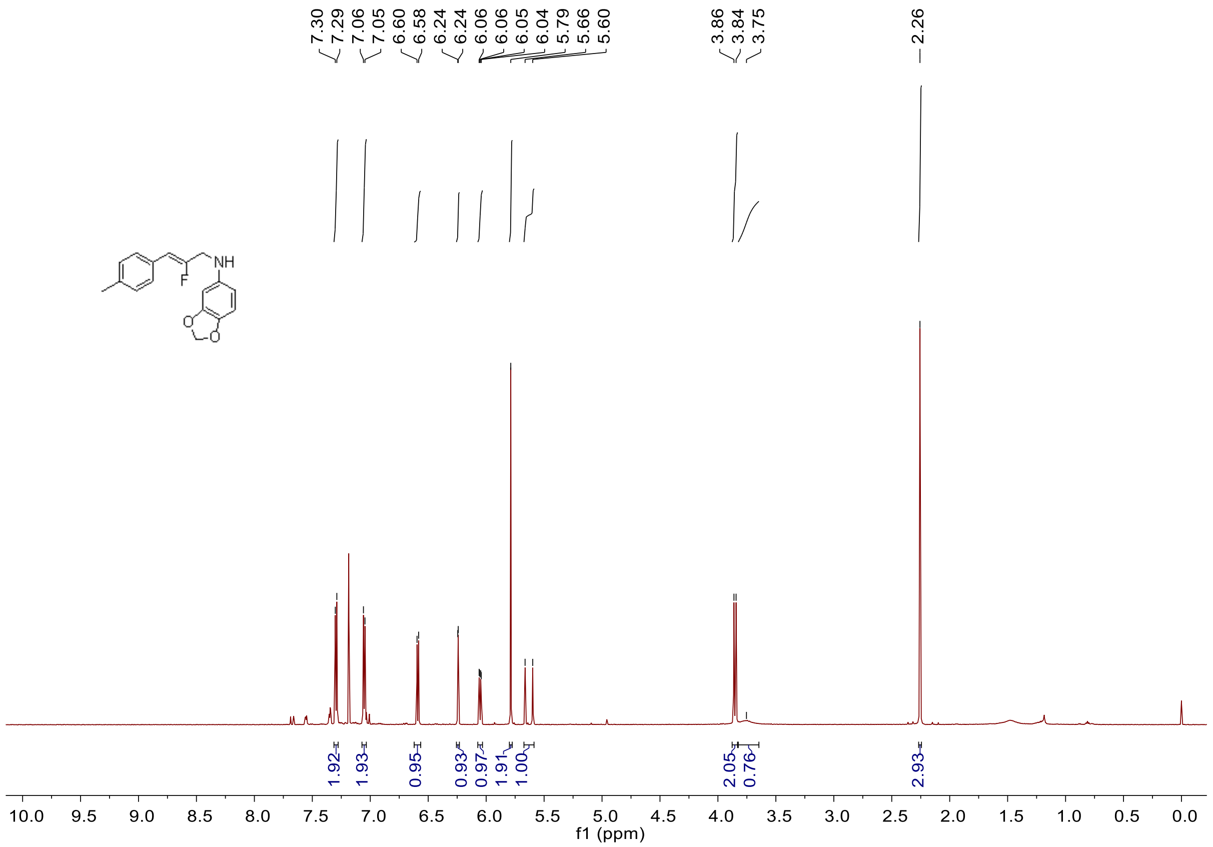

^{19}F NMR (**c27**)

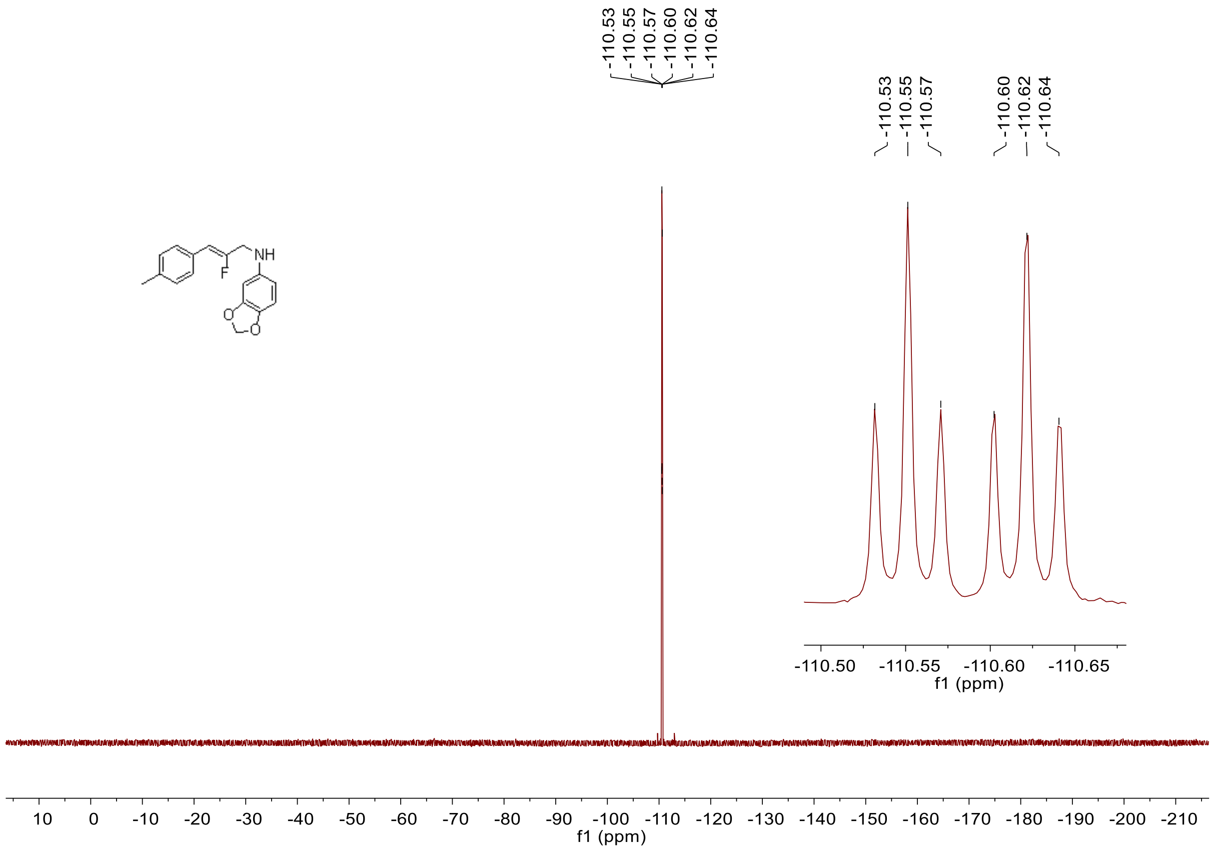

^{13}C NMR (**c27**)

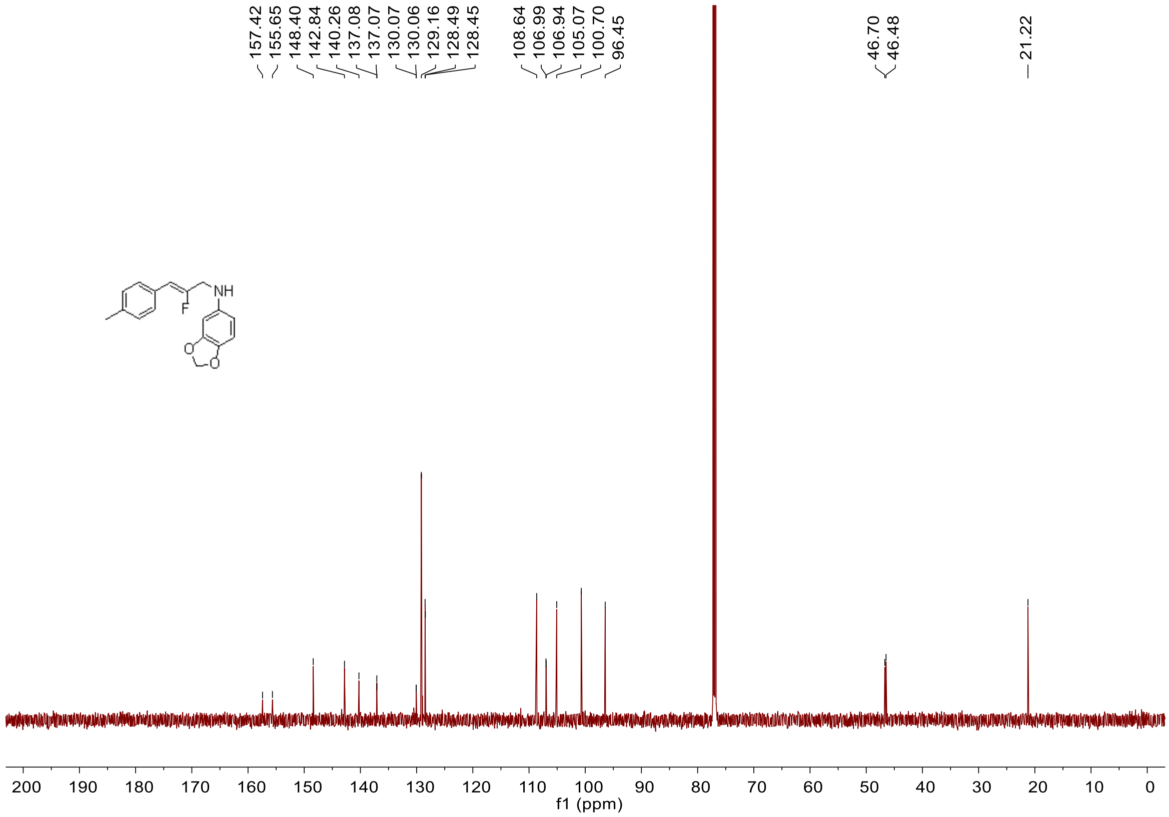

^{1}H NMR (**c28**)

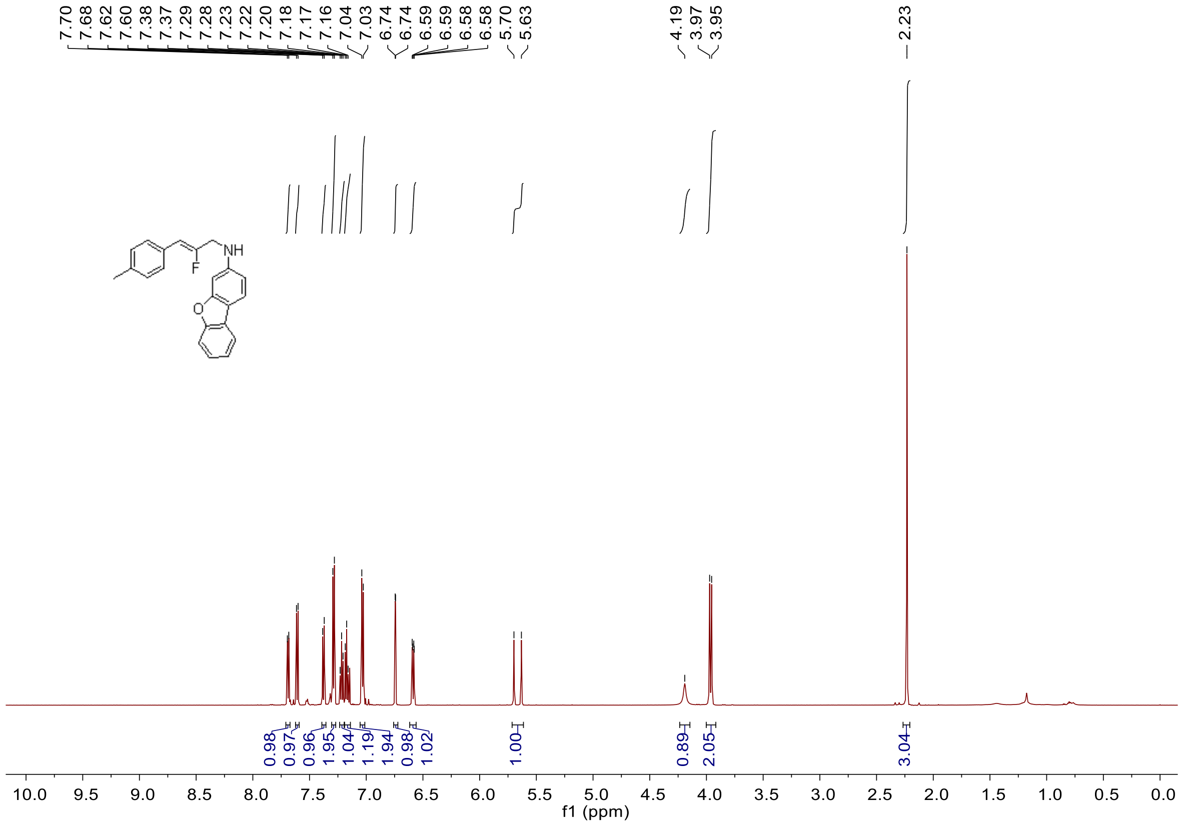

^{19}F NMR (**c28**)

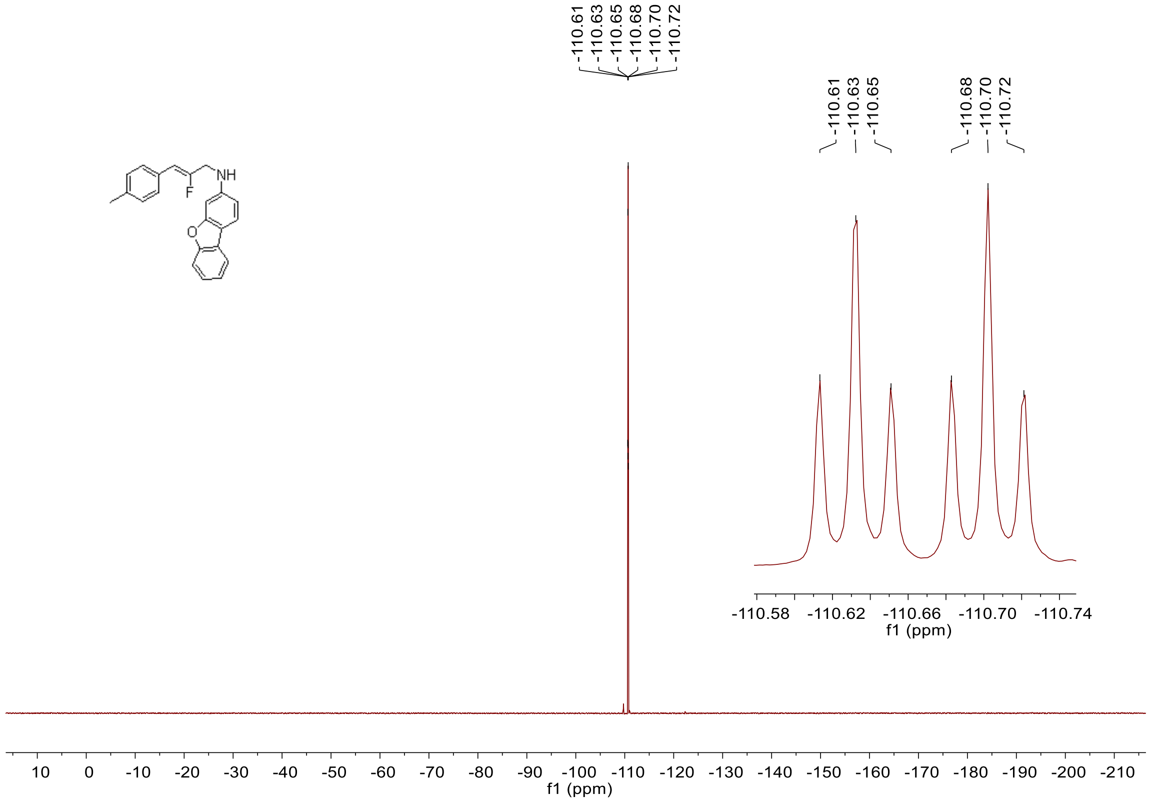

^{13}C NMR (**c28**)

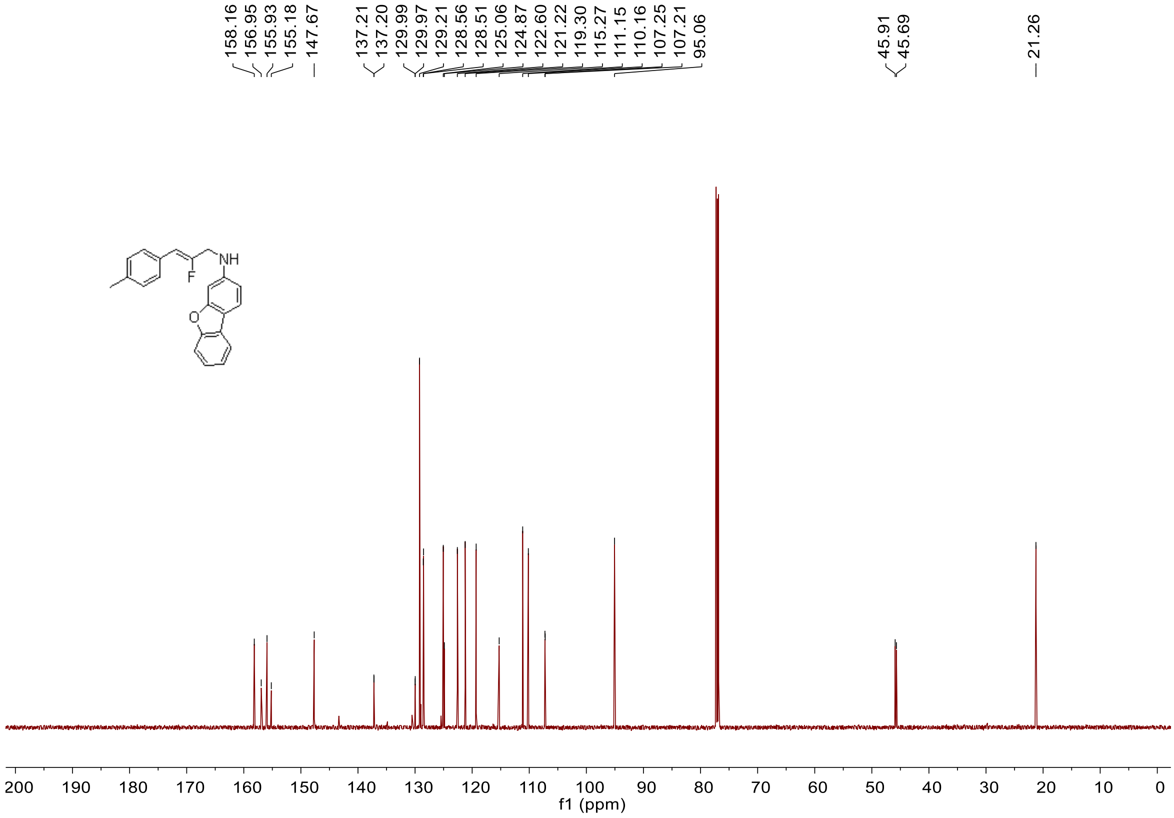

^{1}H NMR (**c29**)

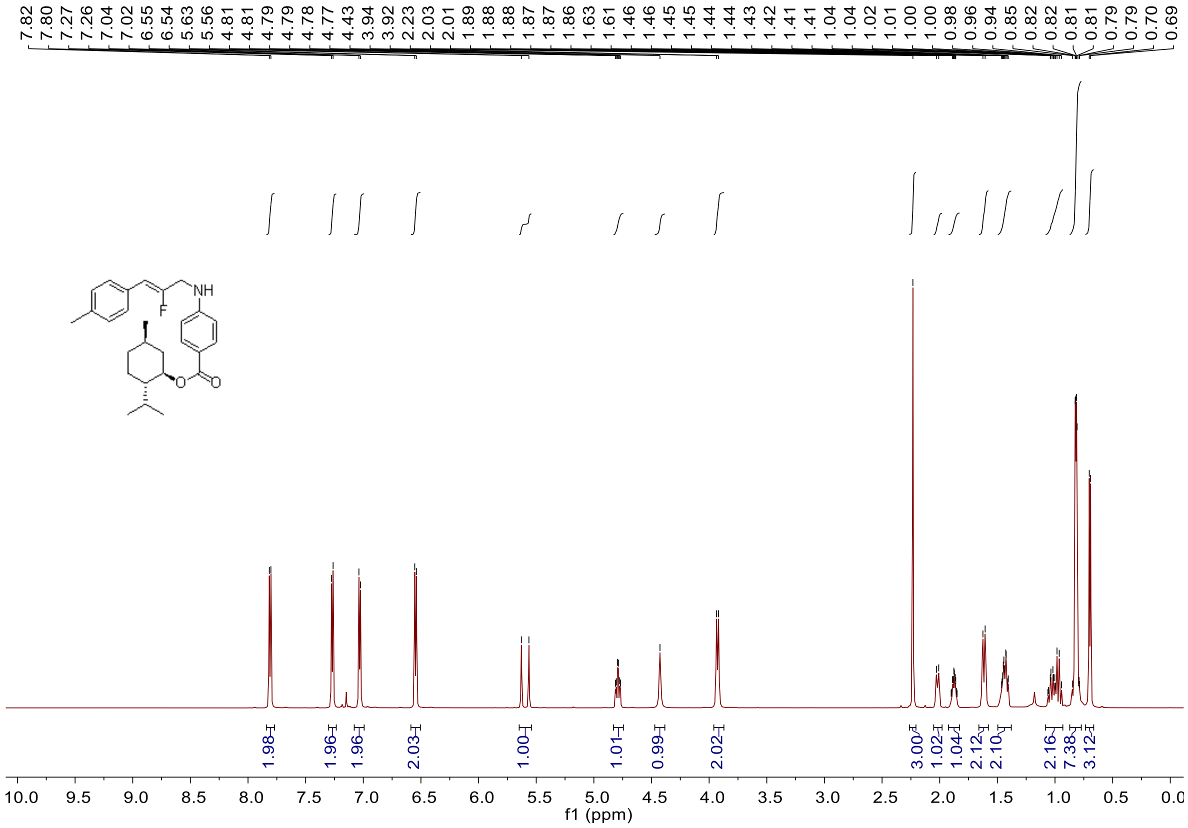

^{19}F NMR (**c29**)

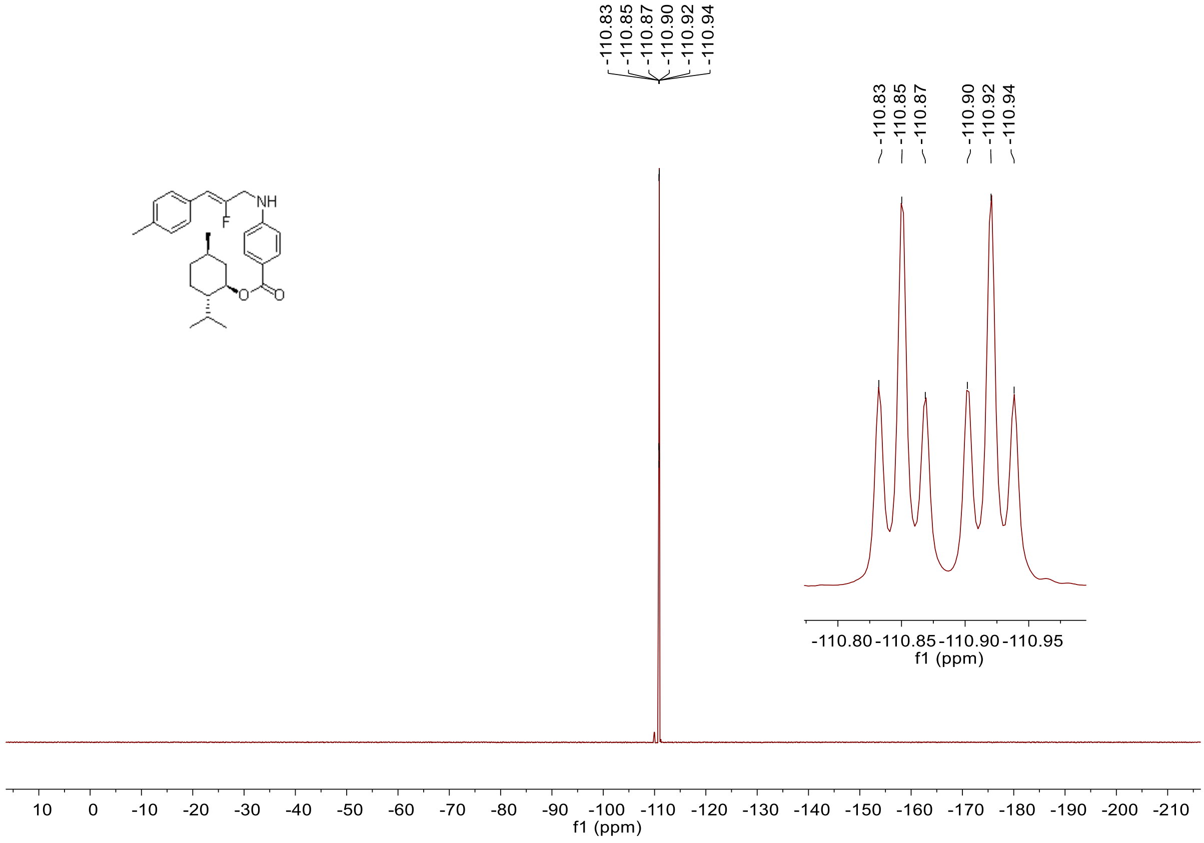

^{13}C NMR (**c29**)

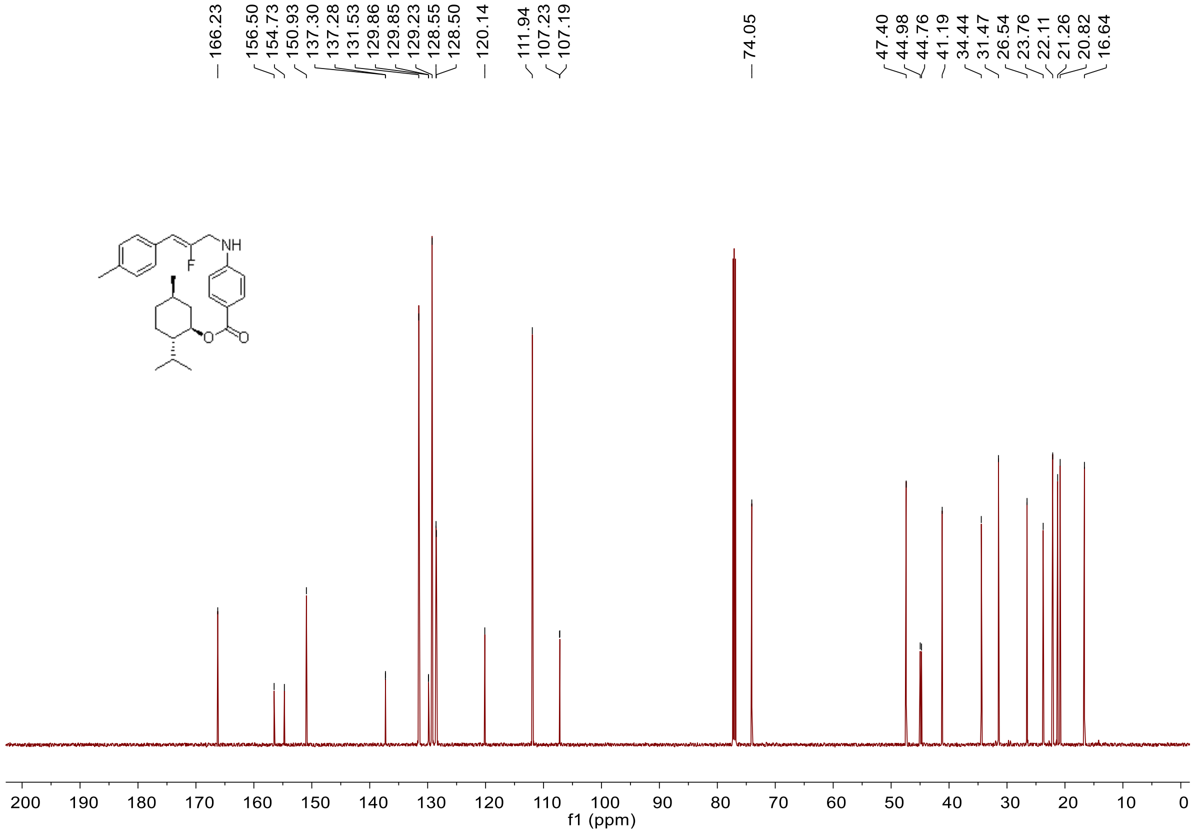

^{1}H NMR (**c30**)

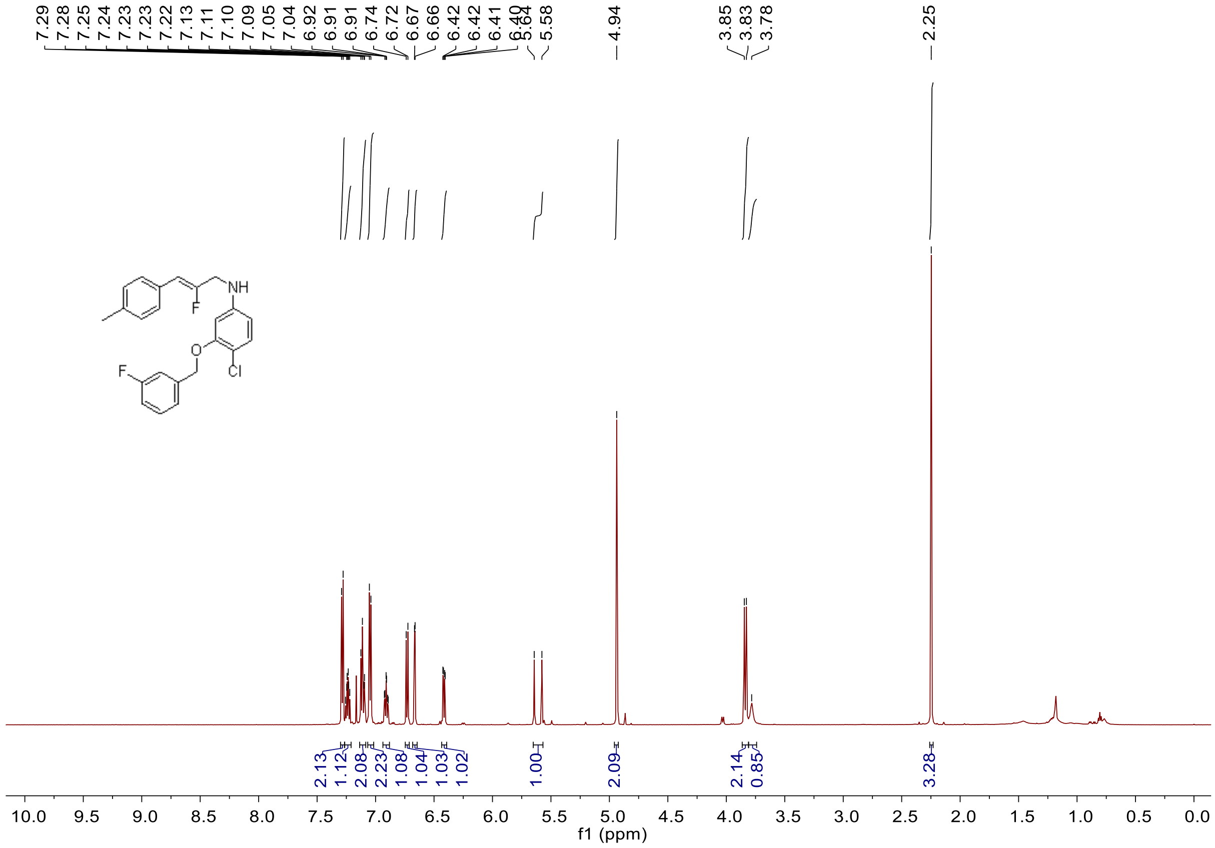

^{19}F NMR (**c30**)

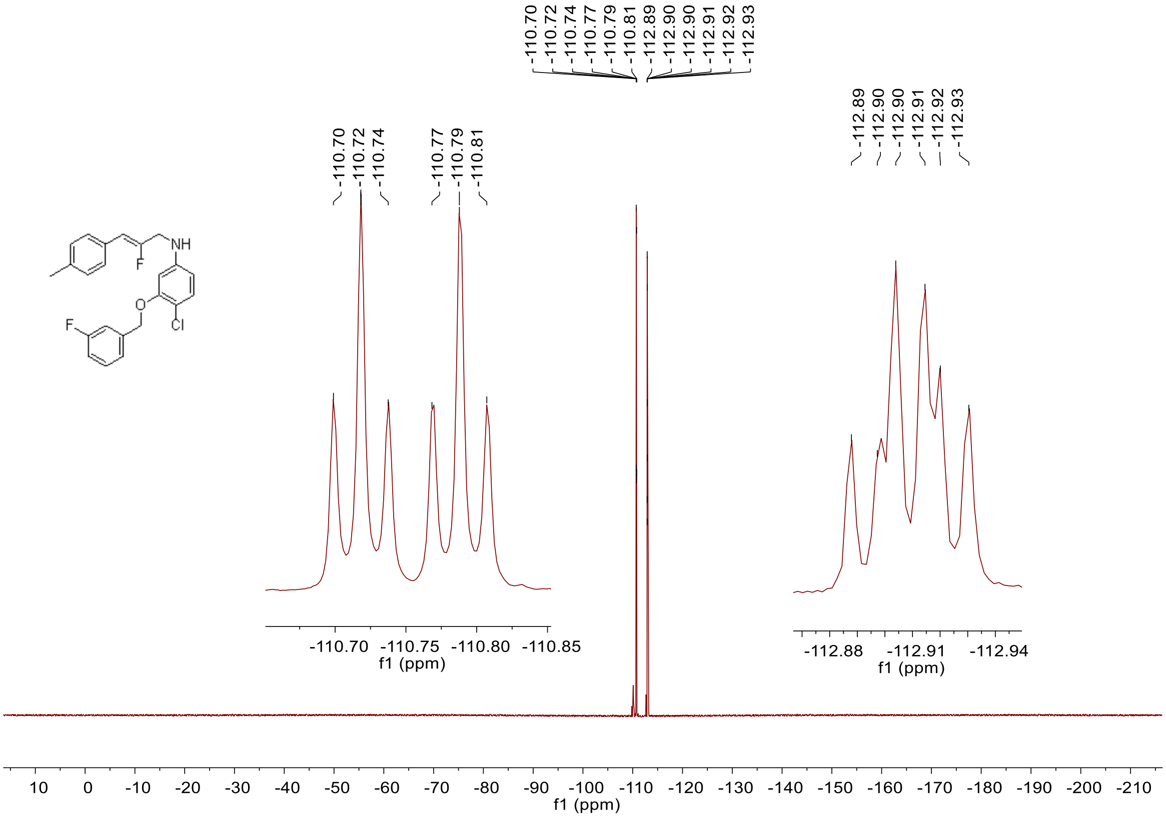

^{13}C NMR (**c30**)

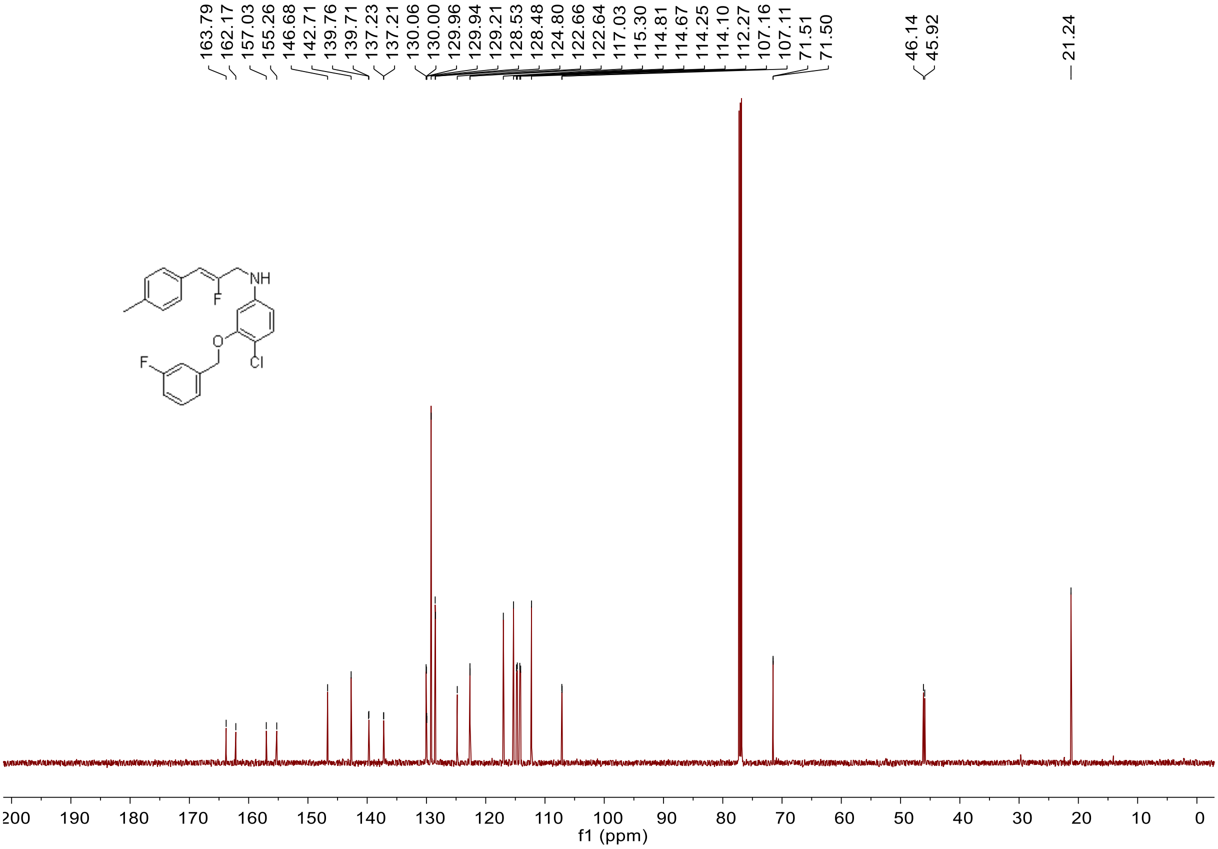

^{1}H NMR (**c31**)

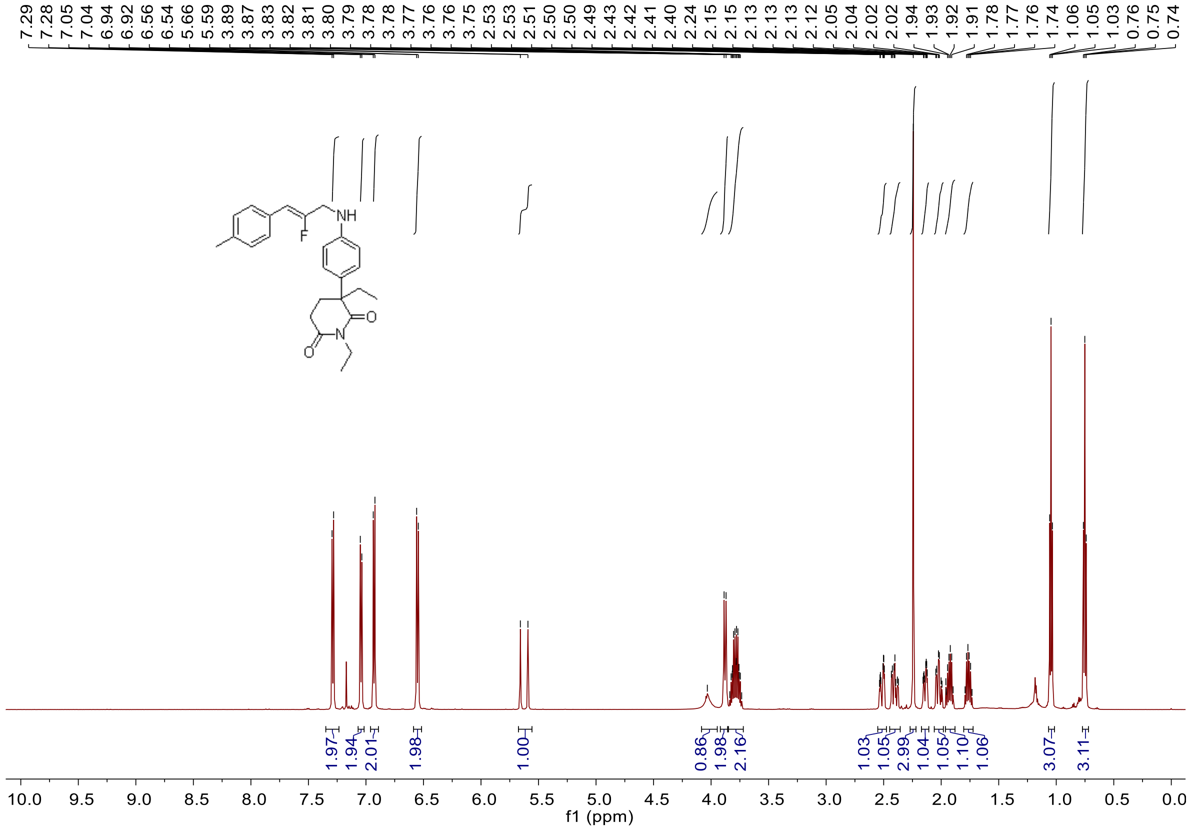

^{19}F NMR (**c31**)

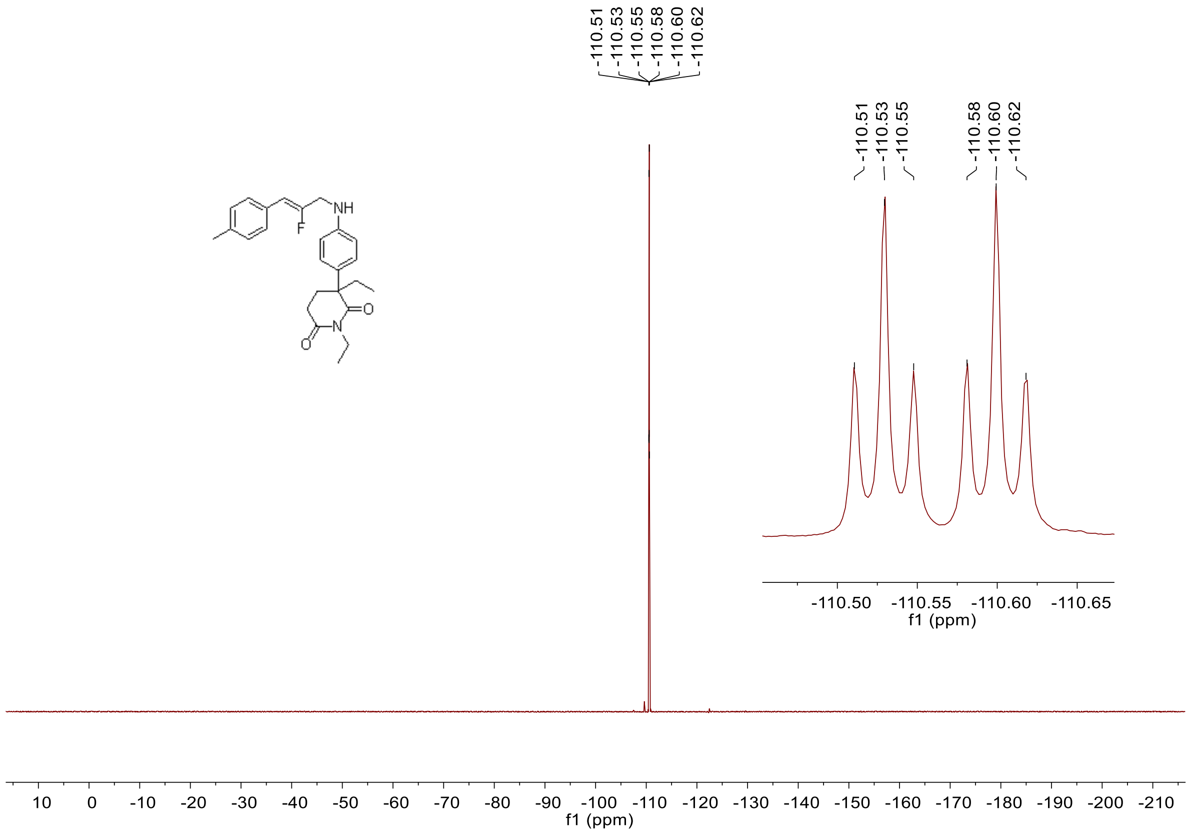

^{13}C NMR (**c31**)

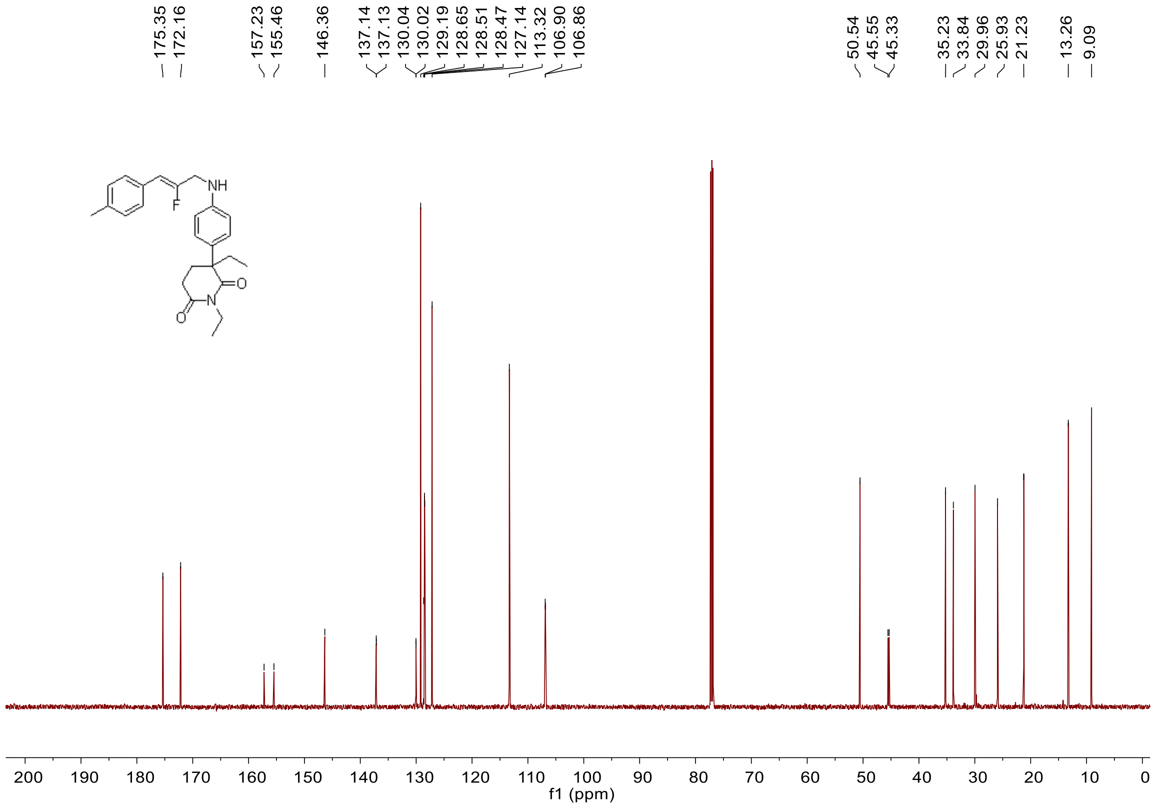

^{1}H NMR (**c32**)

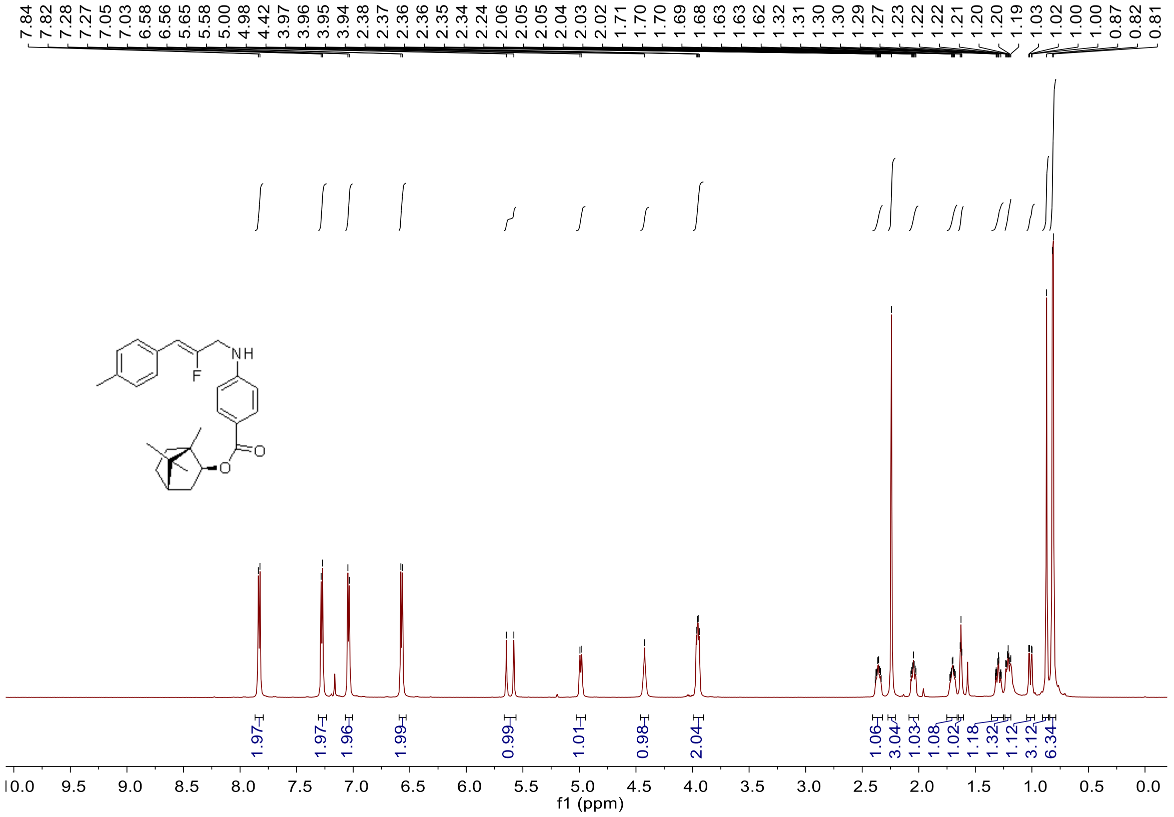

^{19}F NMR (**c32**)

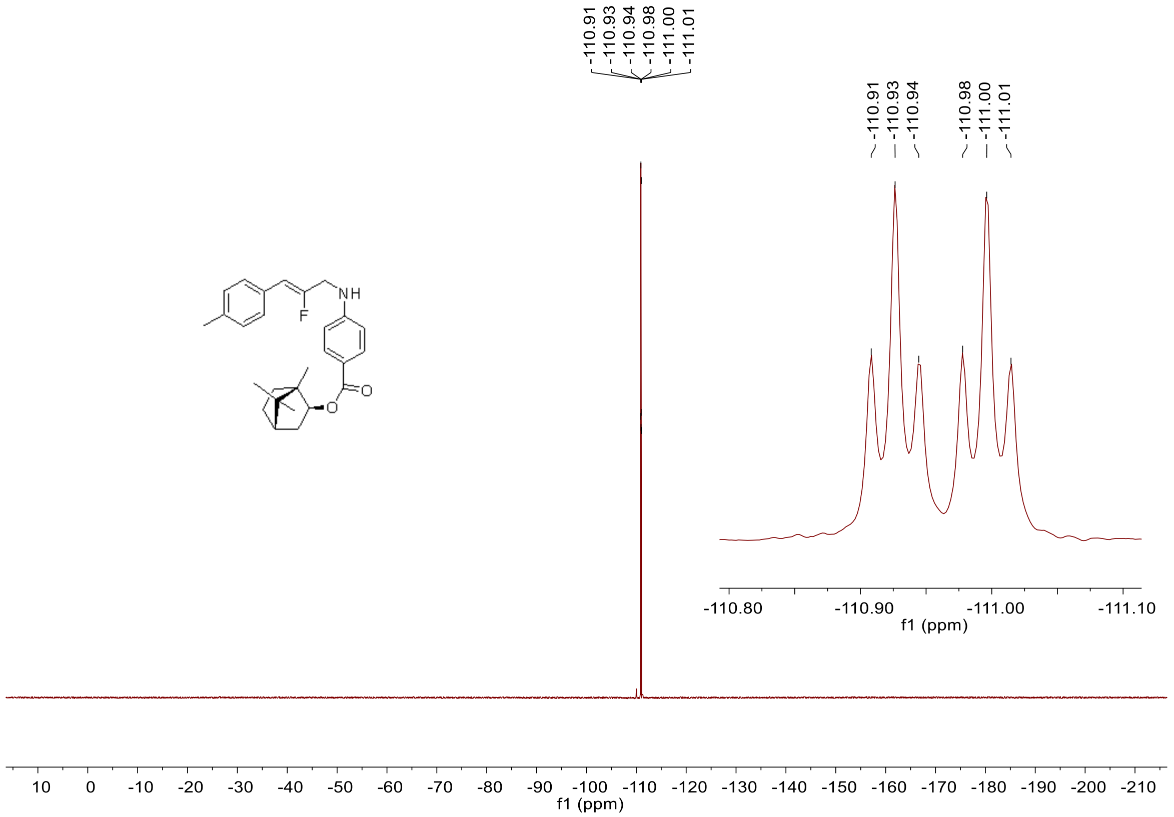

^{13}C NMR (**c32**)

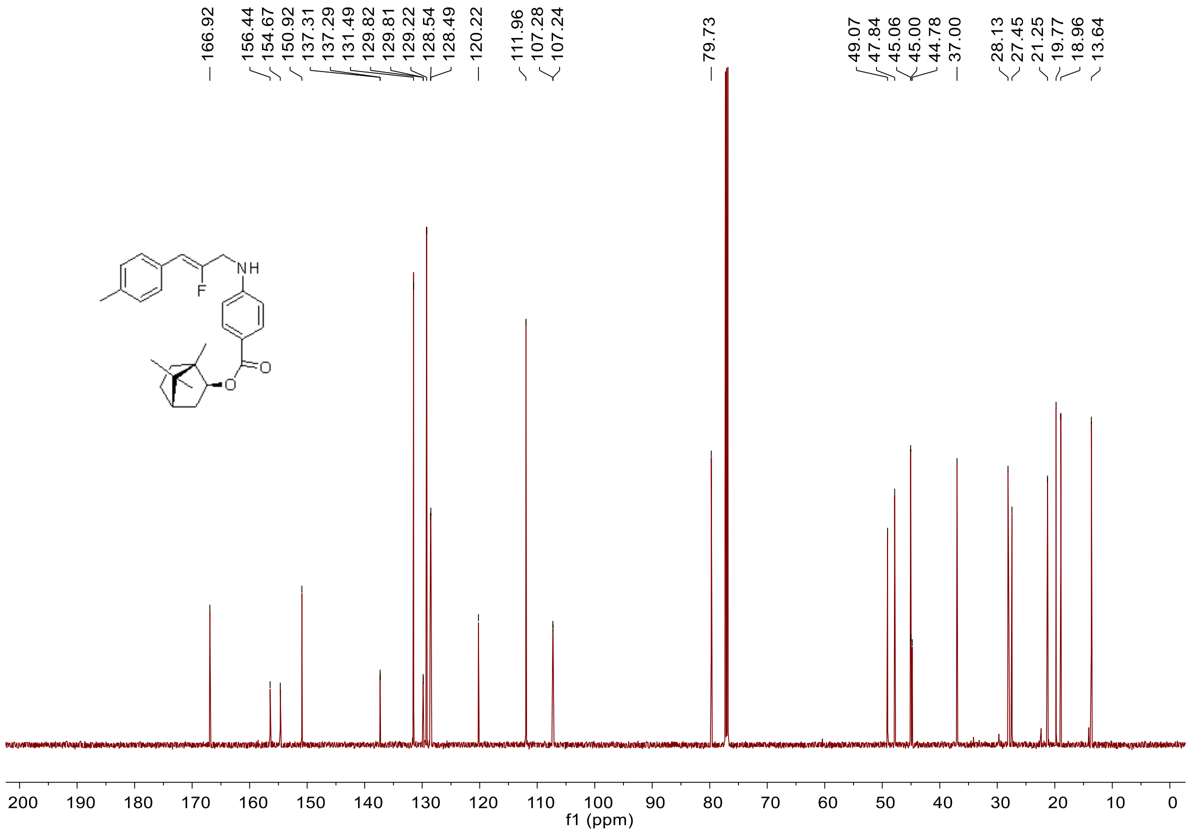

^{1}H NMR (**c33**)

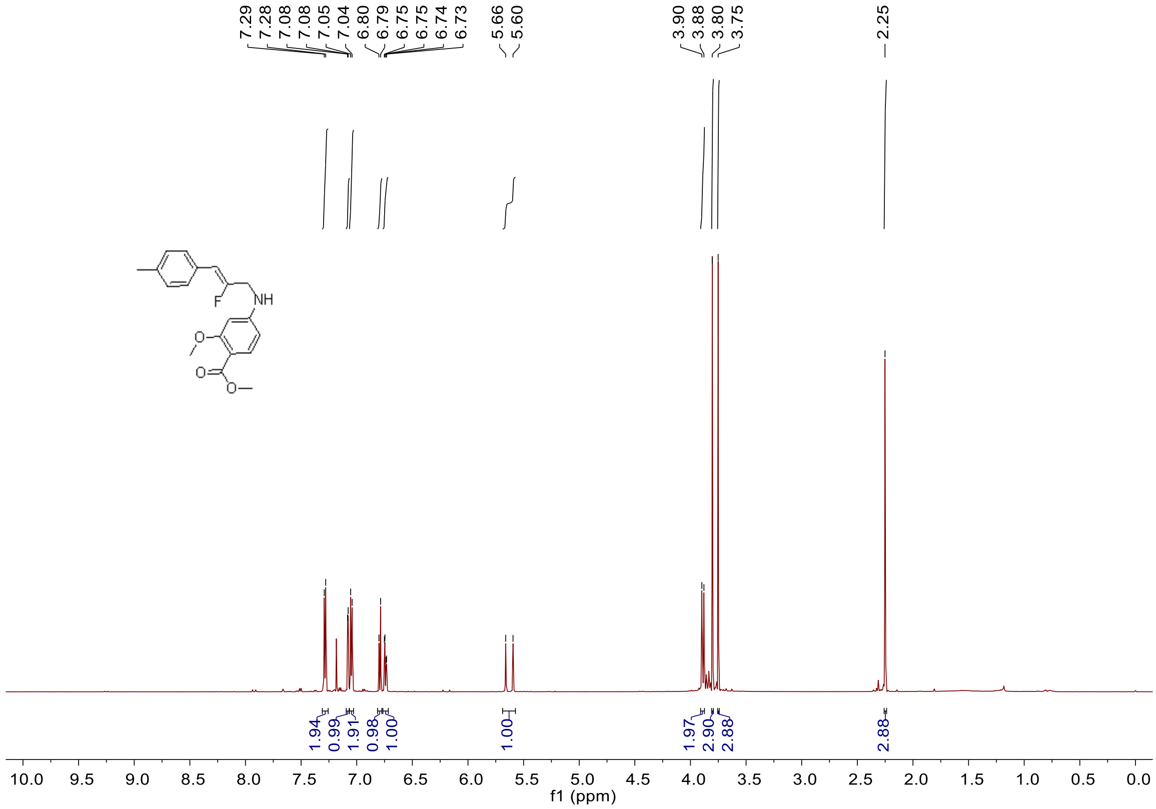

^{19}F NMR (**c33**)

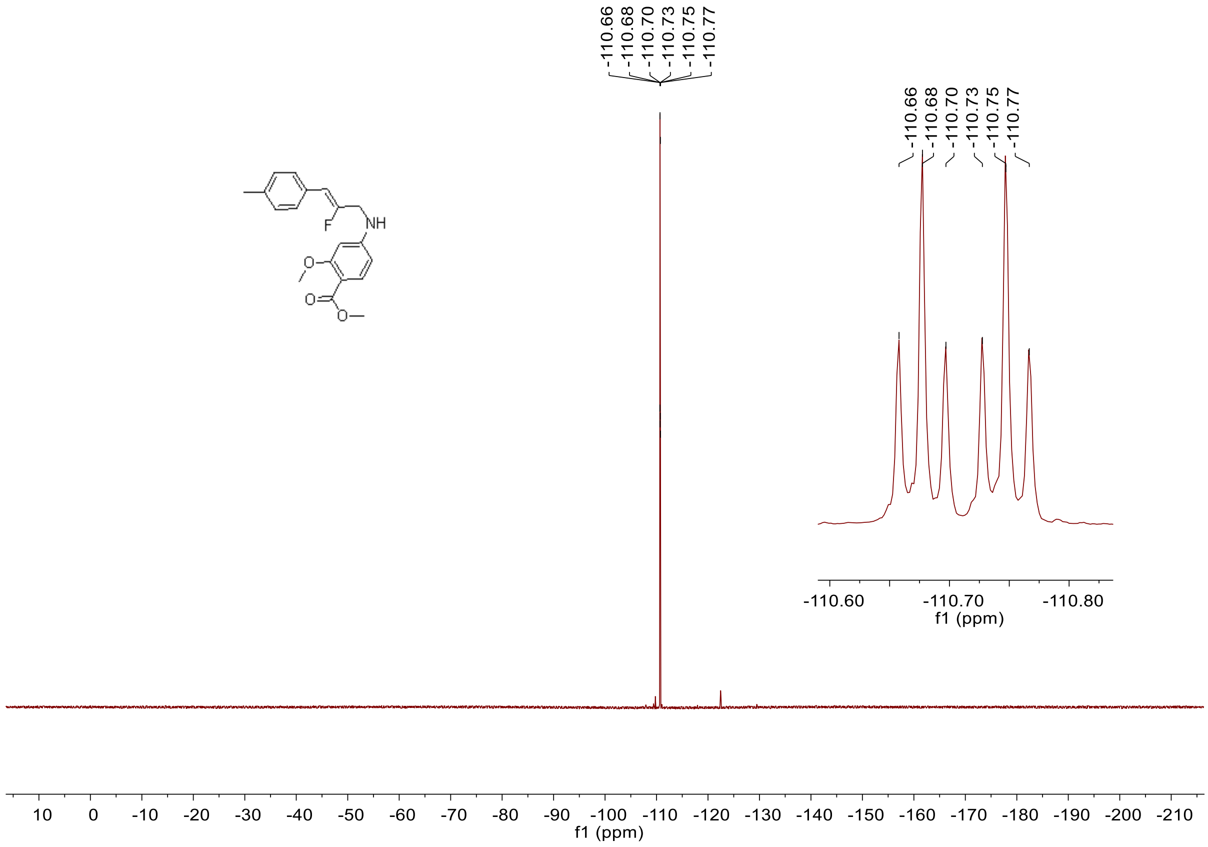

^{13}C NMR (**c33**)

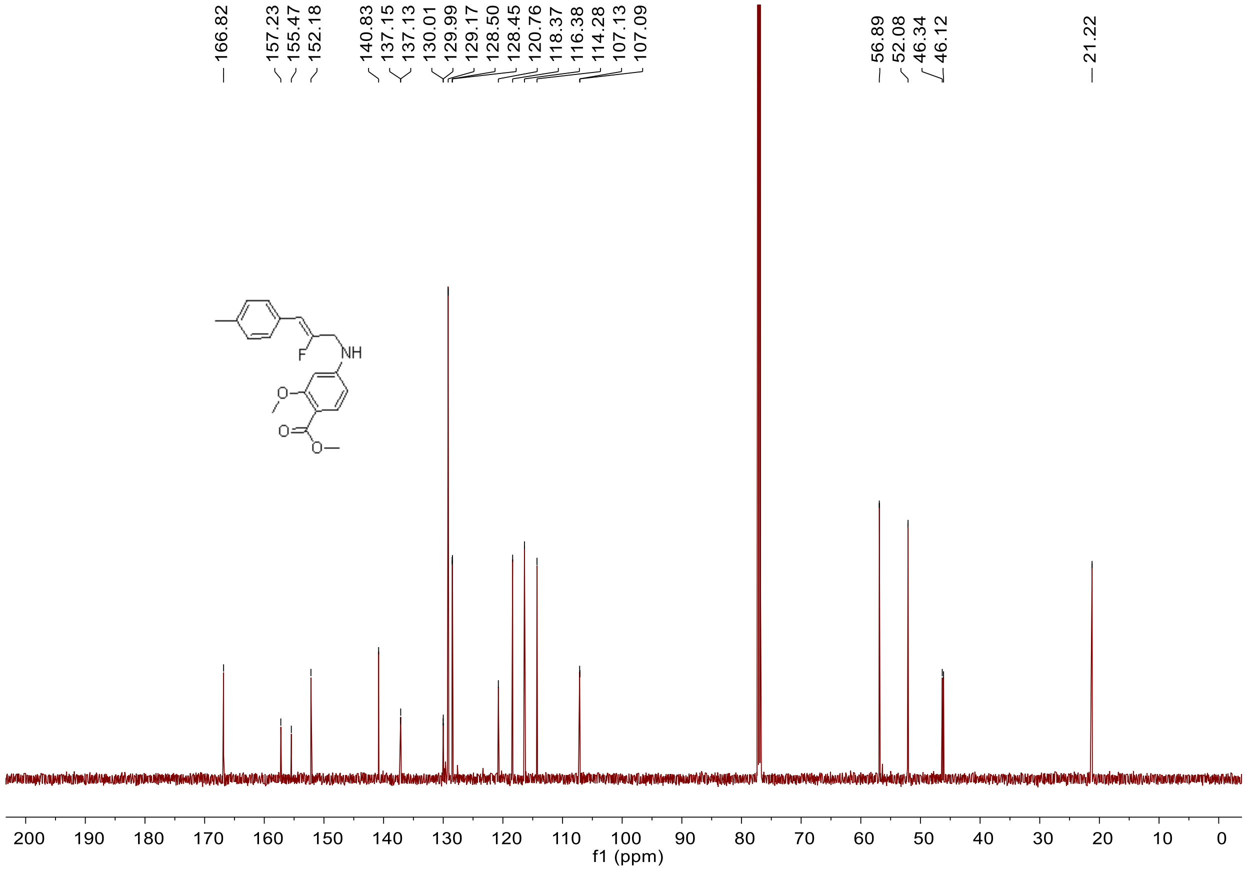

166.82
157.23
155.47
152.18
140.83
137.15
137.13
130.01
129.99
129.17
128.50
128.45
120.76
118.37
116.38
114.28
107.13
107.09
56.89
52.08
46.34
46.12
21.22
F
NH
O
O
O
200 190 180 170 160 150 140 130 120 110 100 90 80 70 60 50 40 30 20 10 0
f1 (ppm)

^{1}H NMR (**d1**)

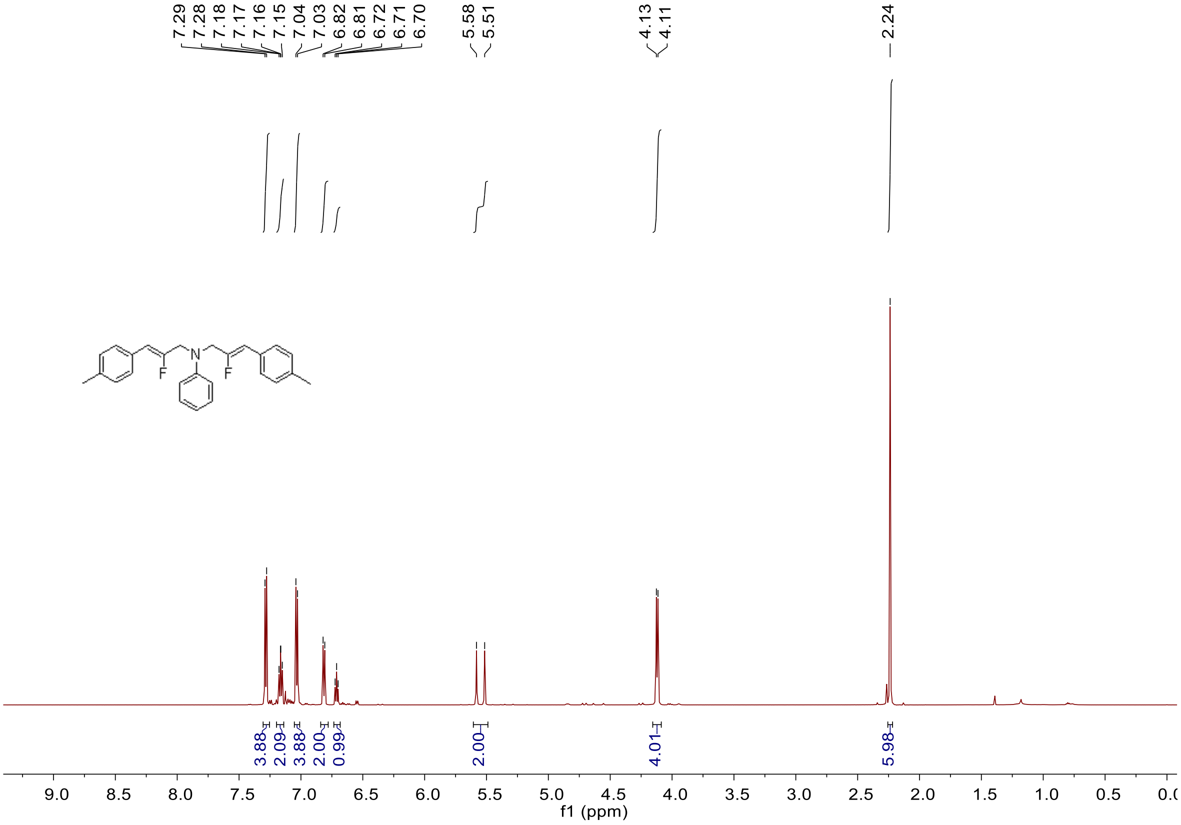

^{19}F NMR (**d1**)

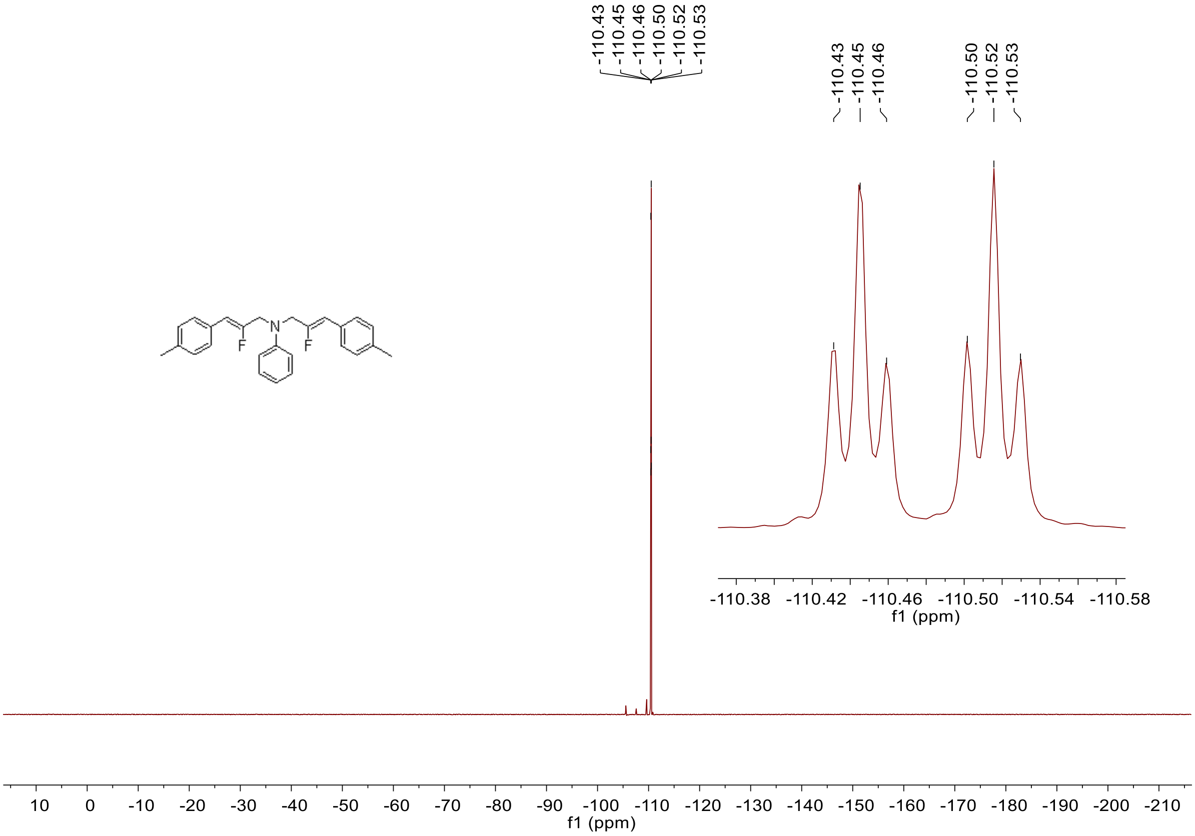

^{13}C NMR (**d1**)

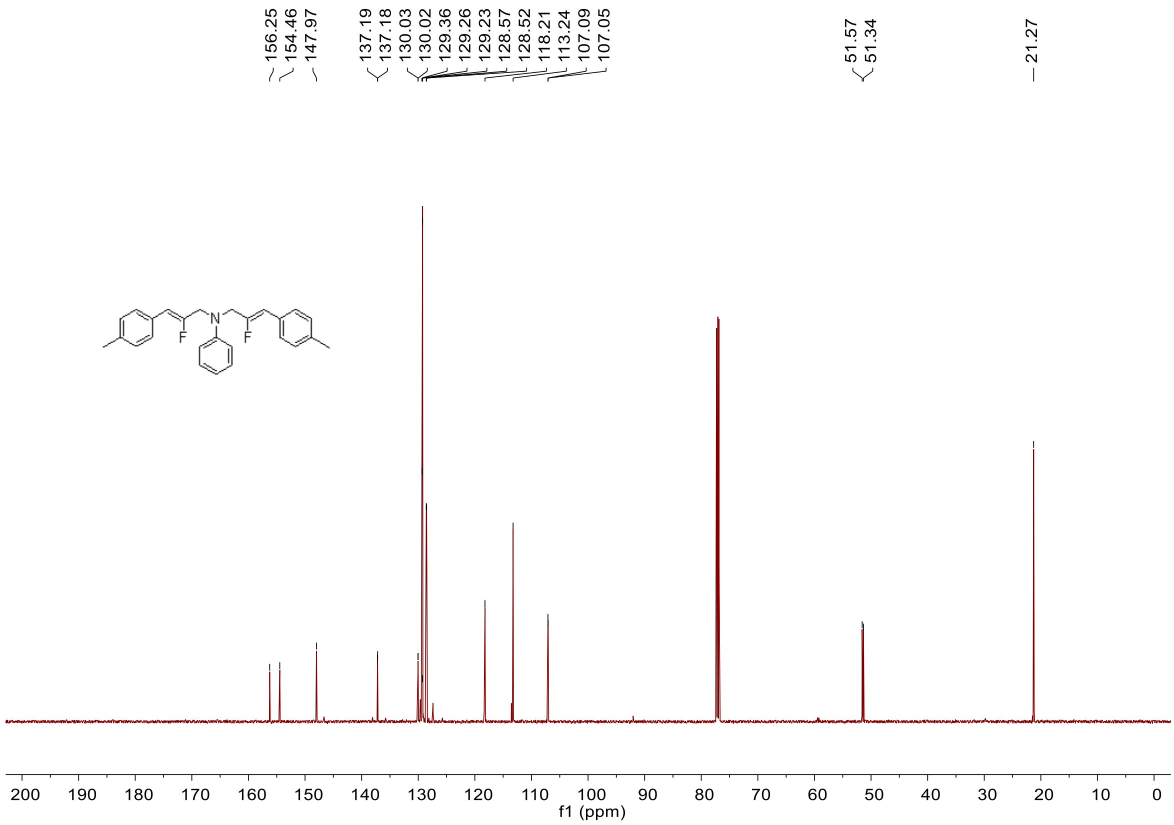

^{1}H NMR (**d2**)

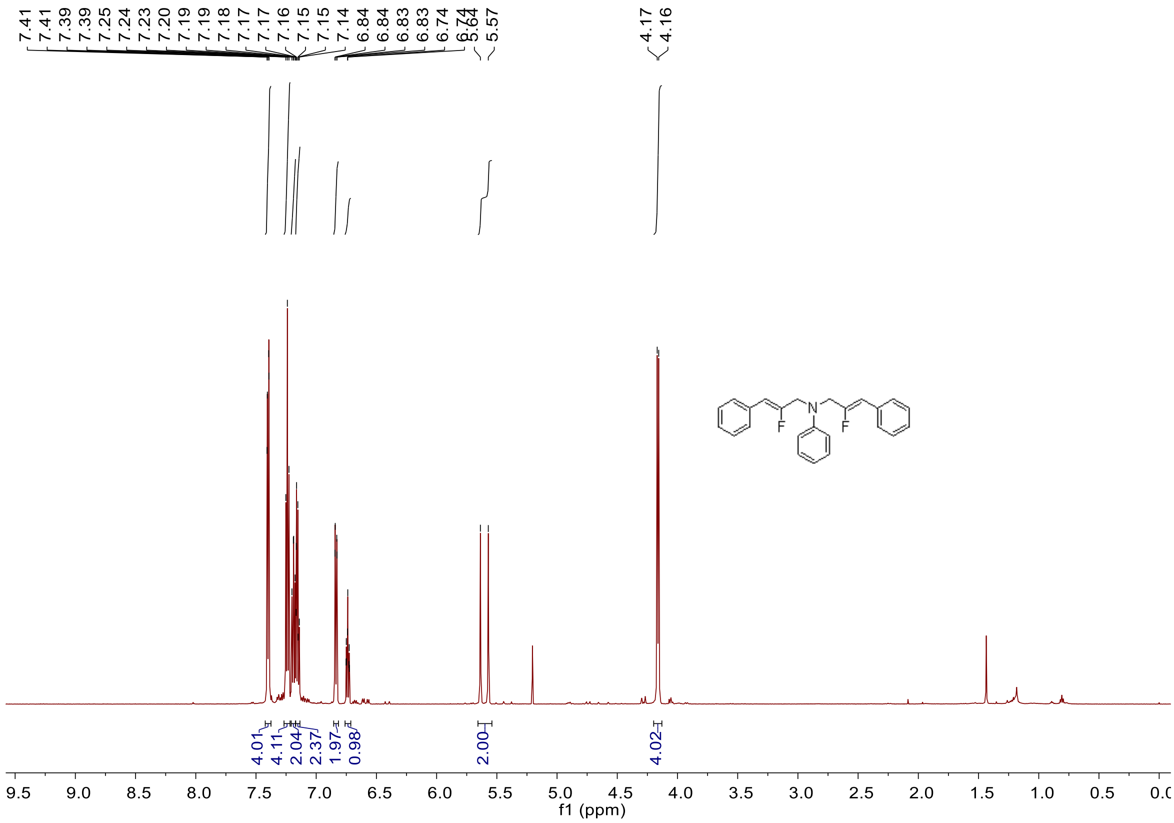

^{19}F NMR (**d2**)

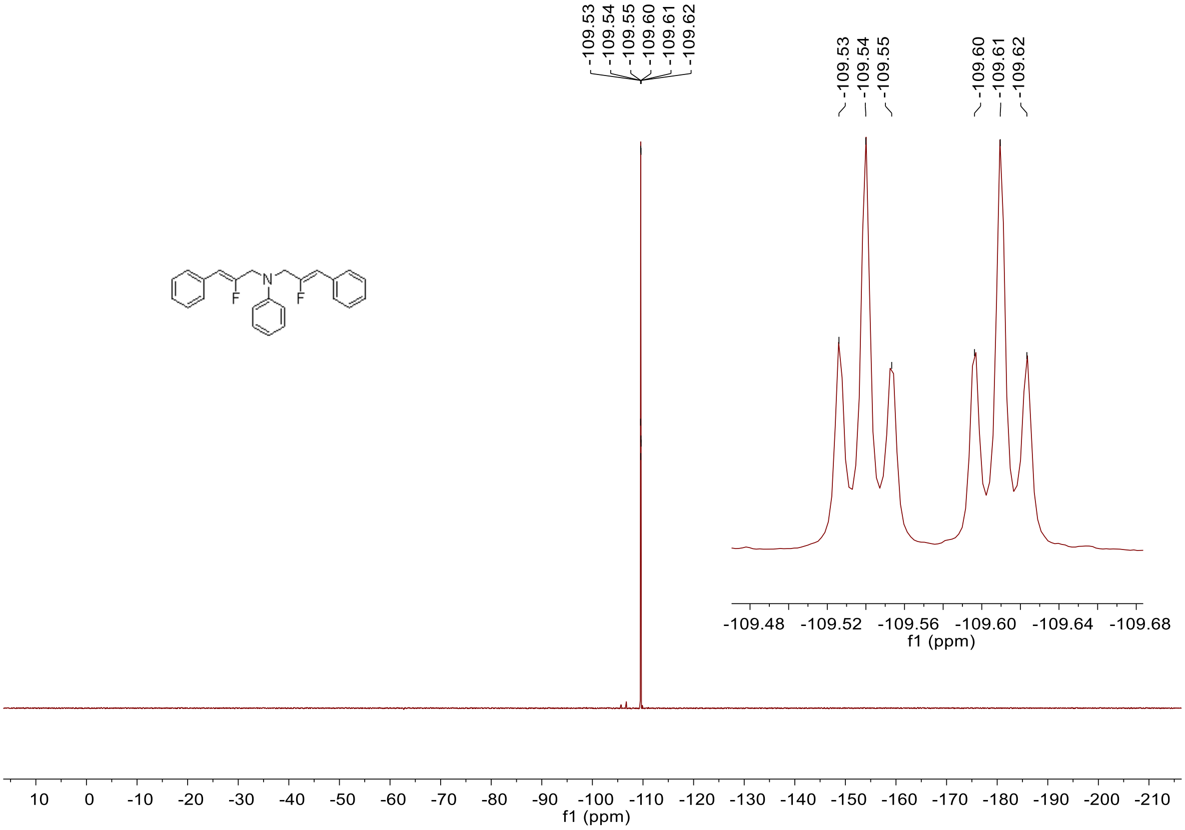

^{13}C NMR (**d2**)

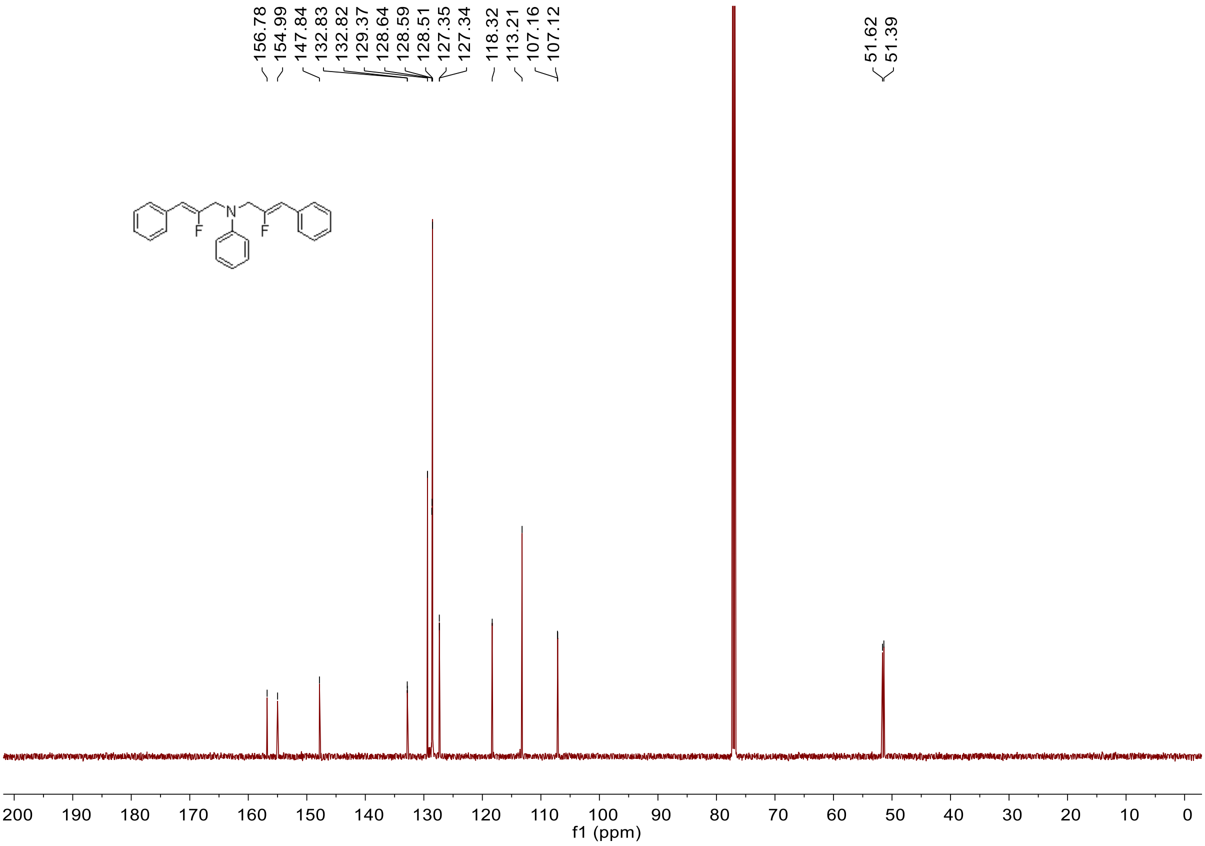

^{1}H NMR (**d3**)

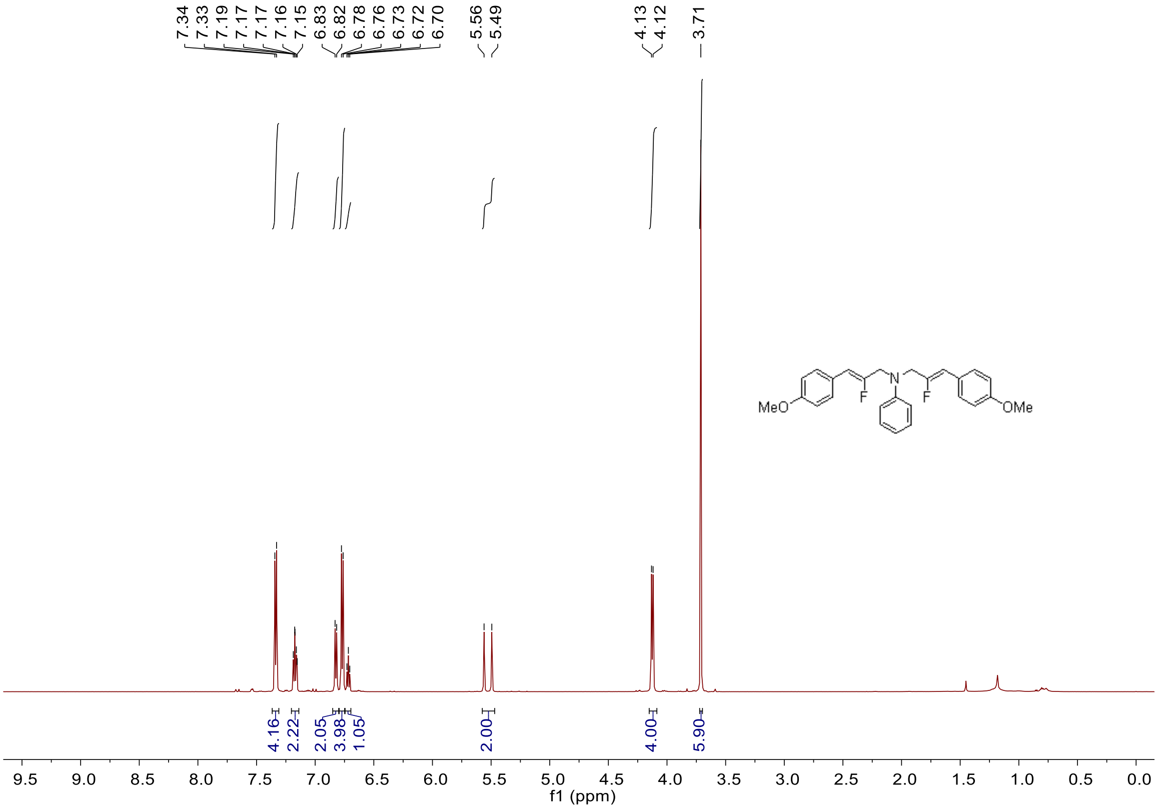

^{19}F NMR (**d3**)

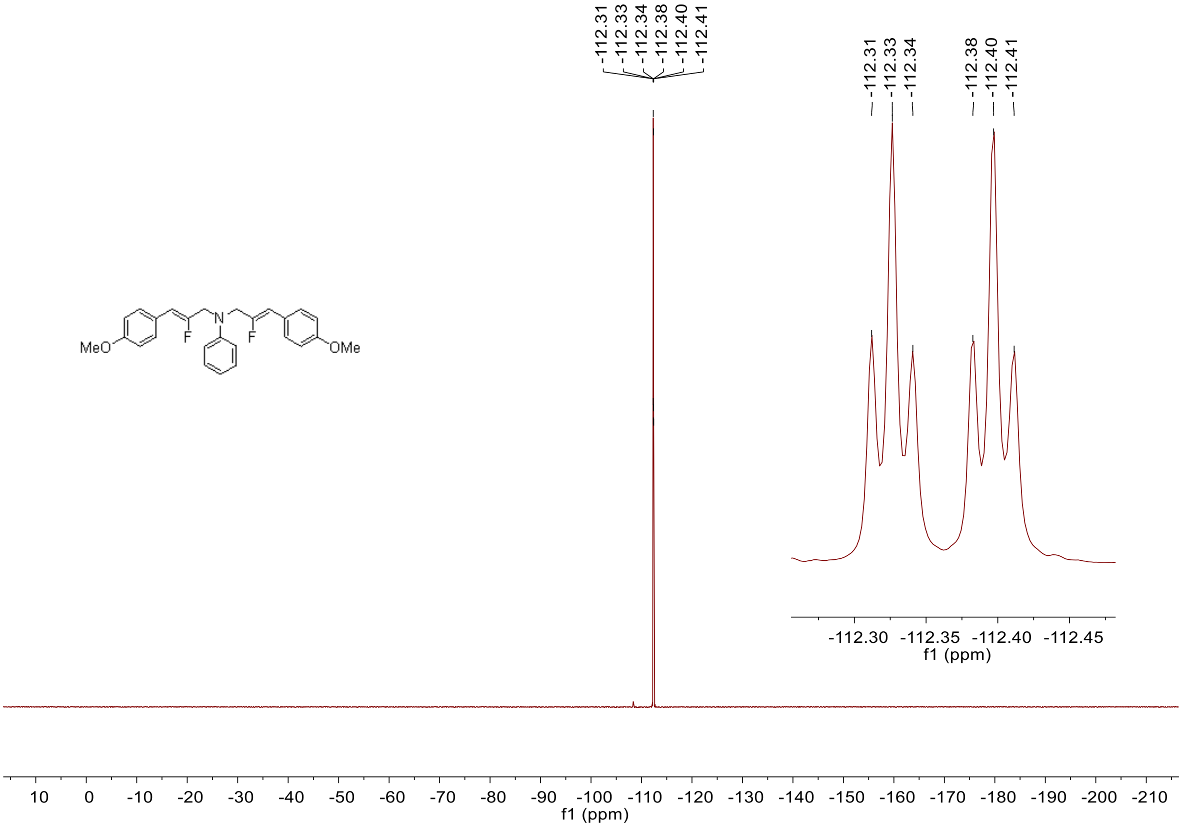

^{13}C NMR (**d3**)

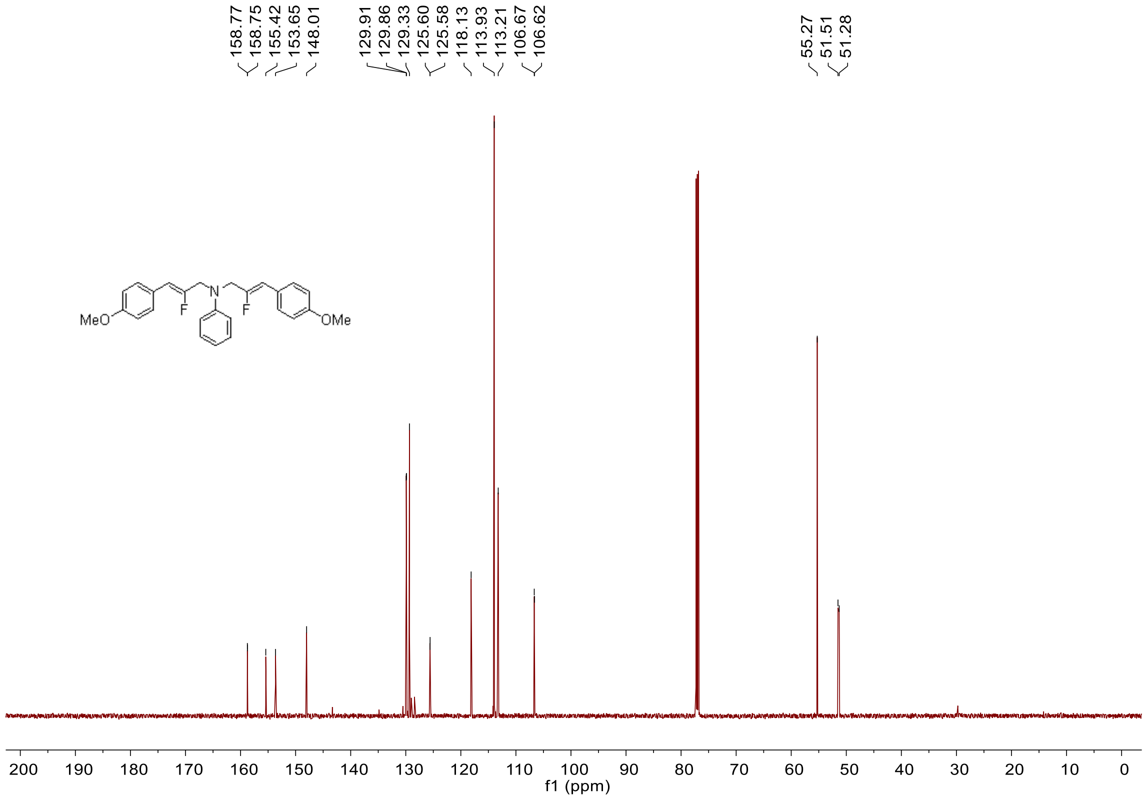

^{1}H NMR (**d4**)

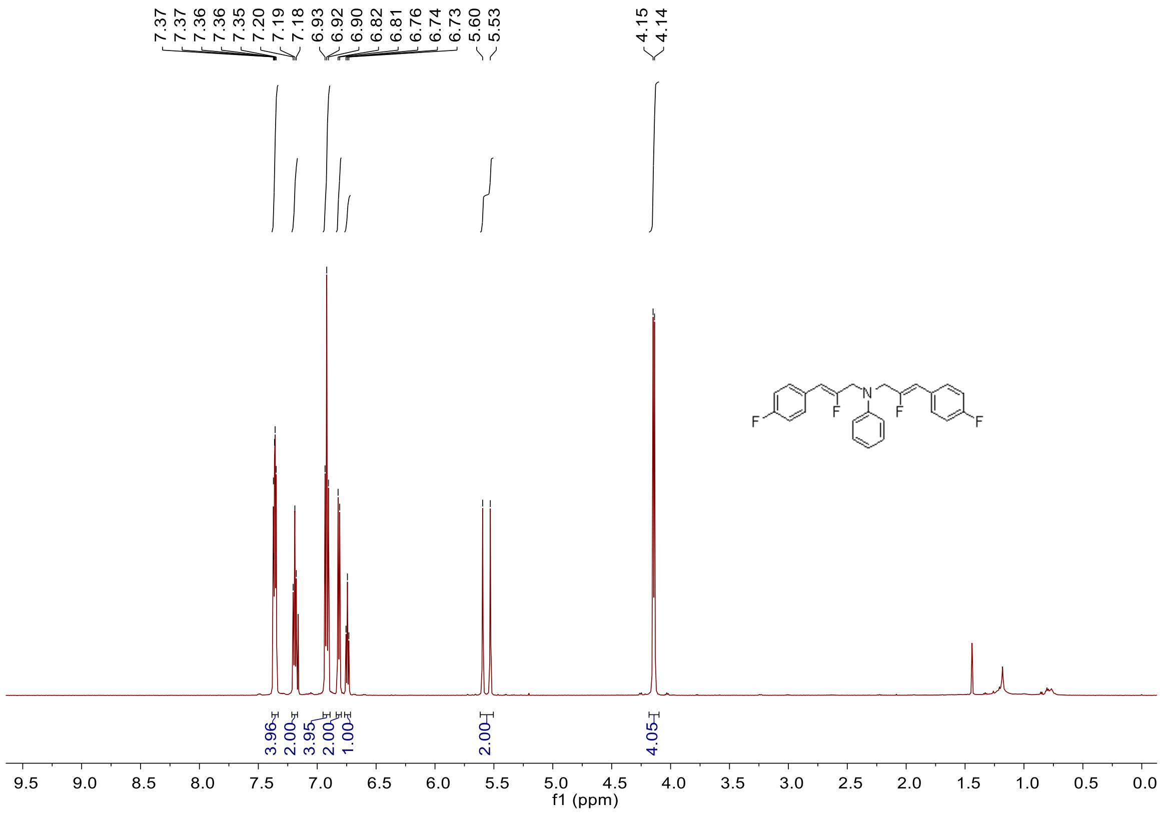

^{19}F NMR (**d4**)

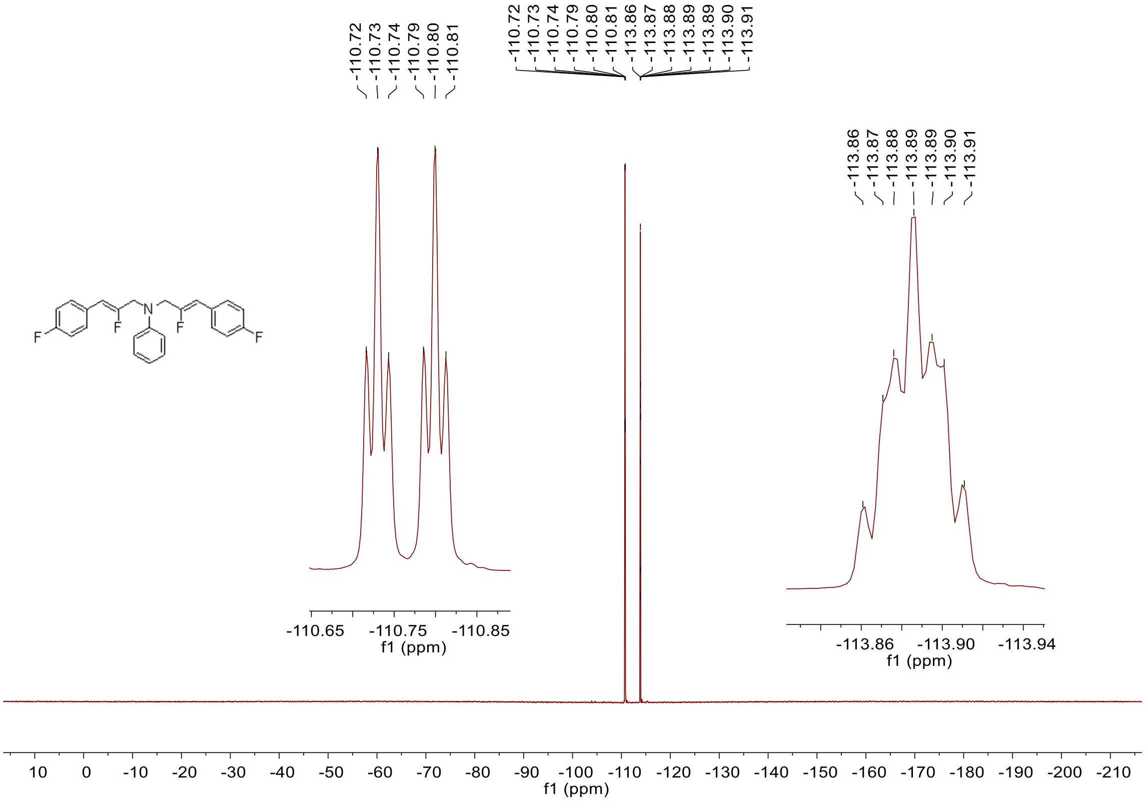

^{13}C NMR (**d4**)

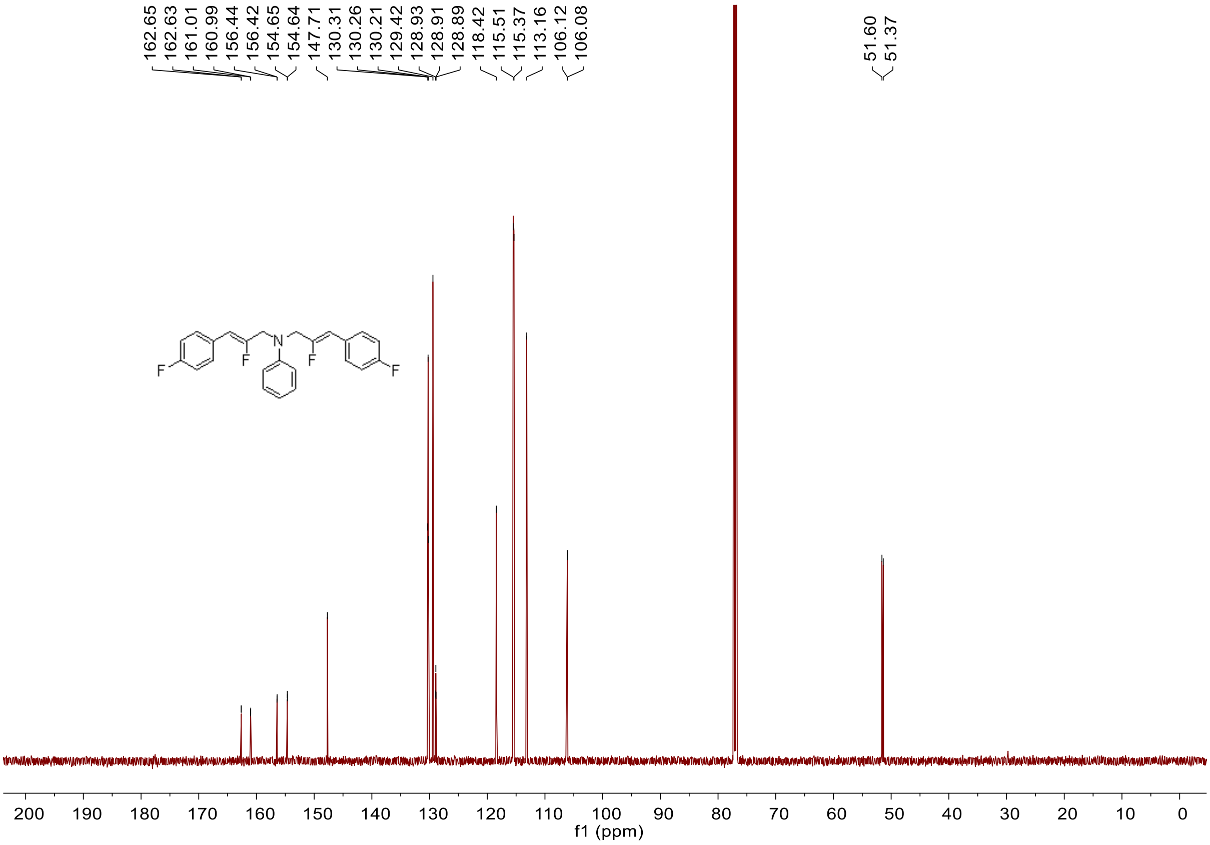

^{1}H NMR (**d5**)

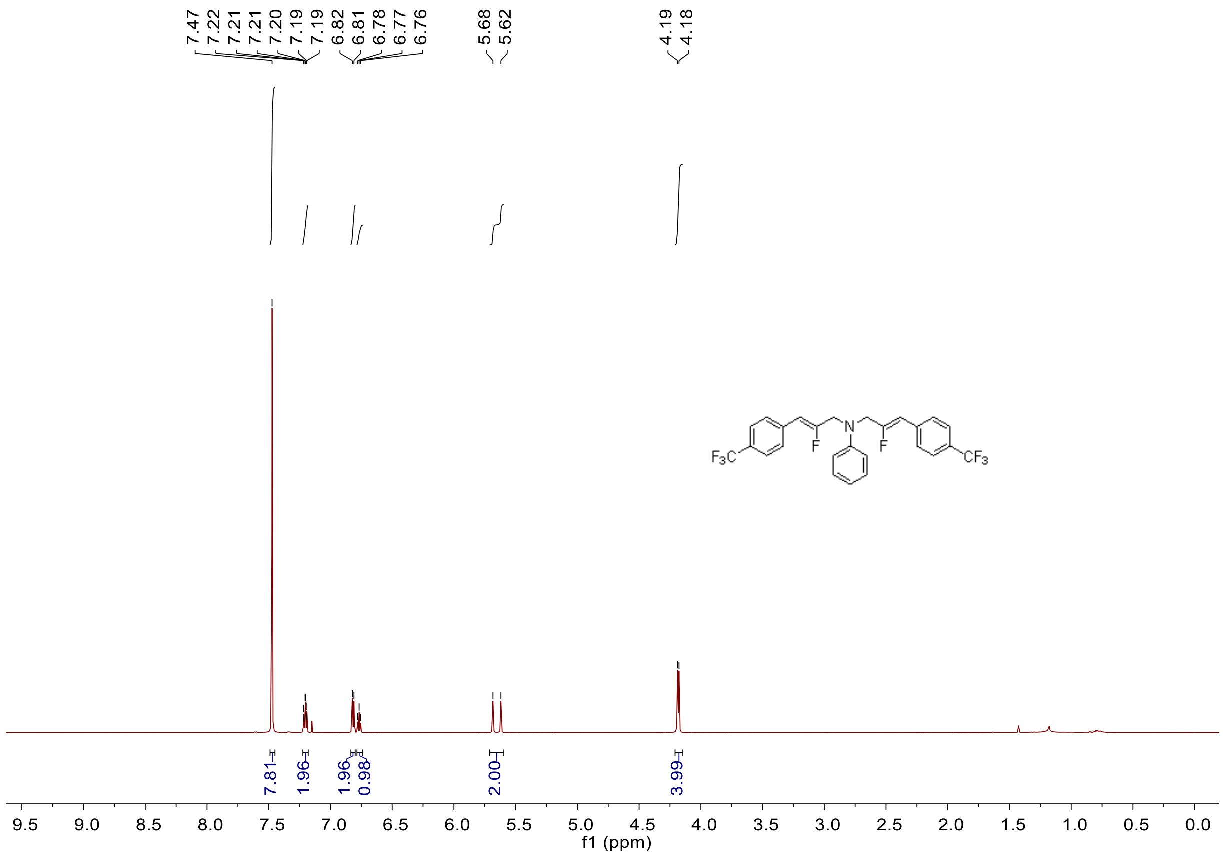

^{19}F NMR (**d5**)

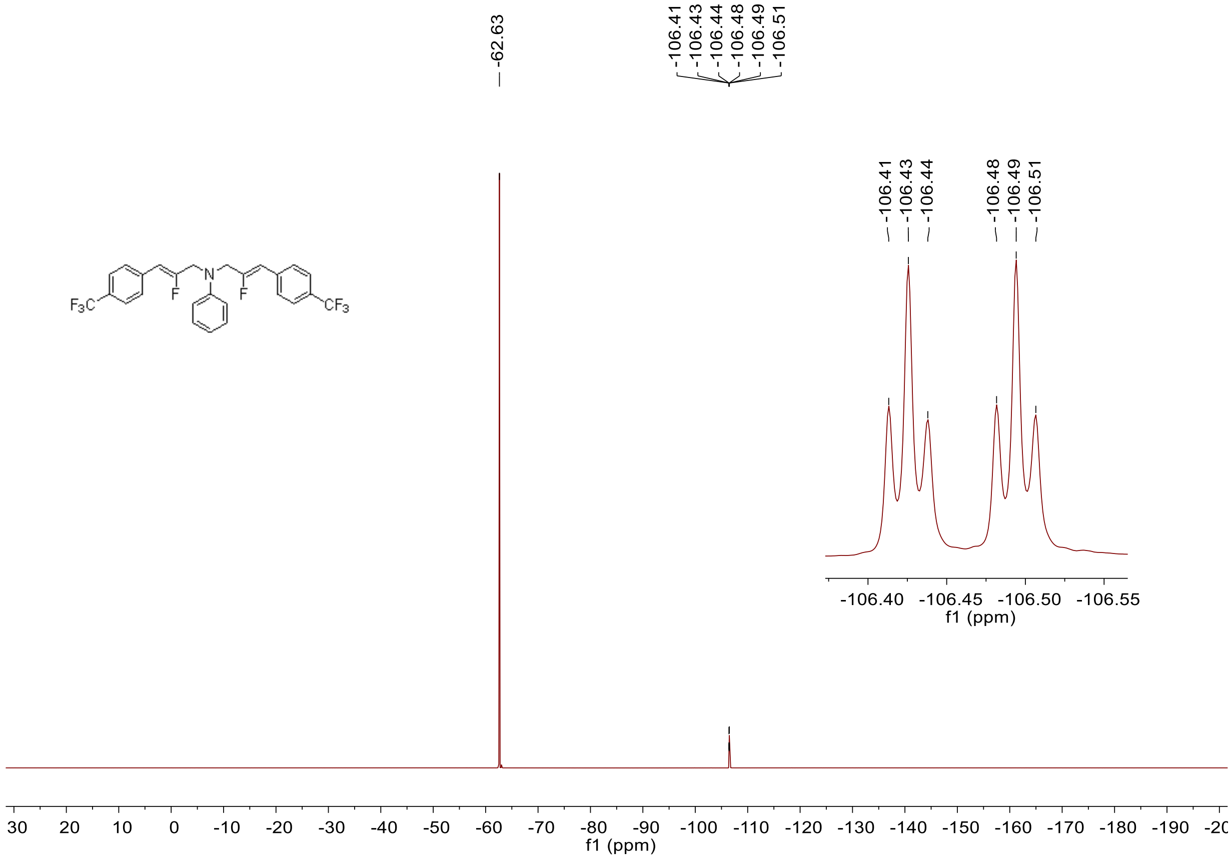

^{13}C NMR (**d5**)

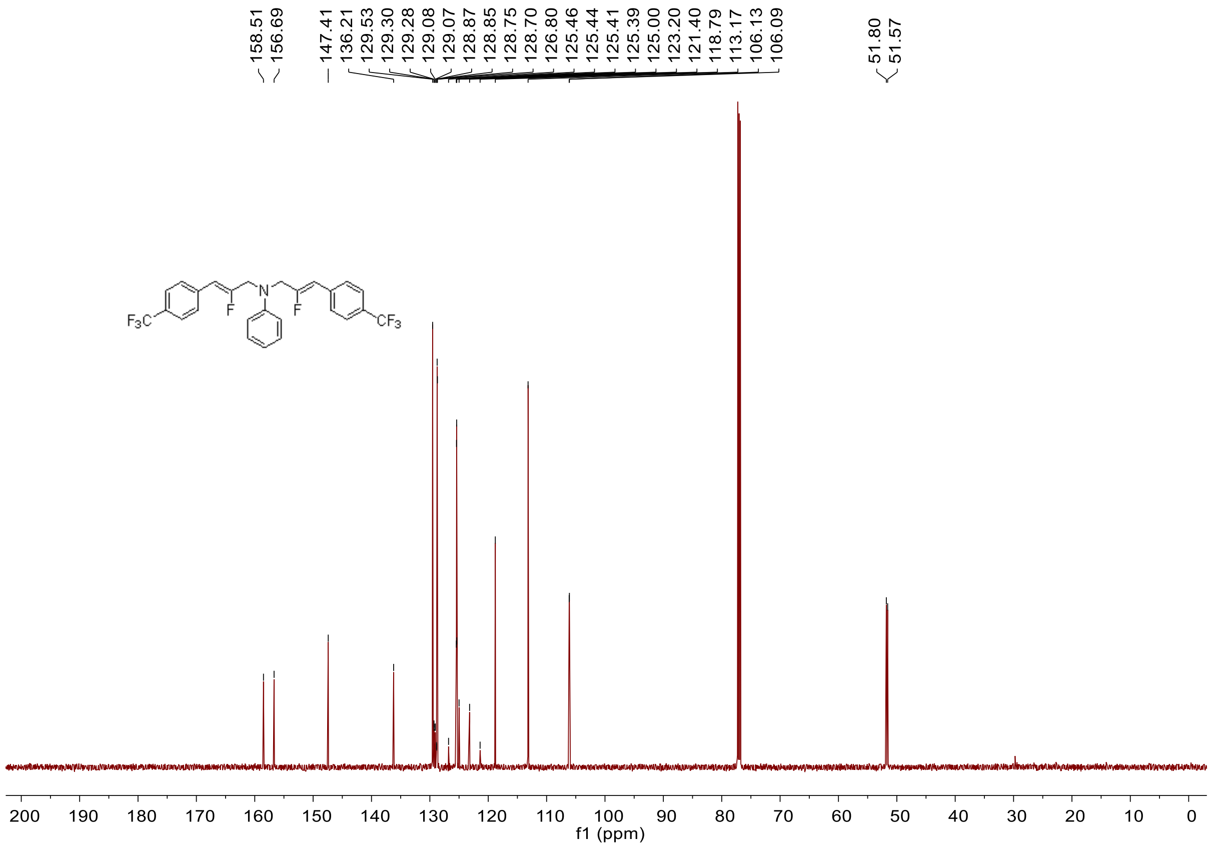

^{1}H NMR (**d6**)

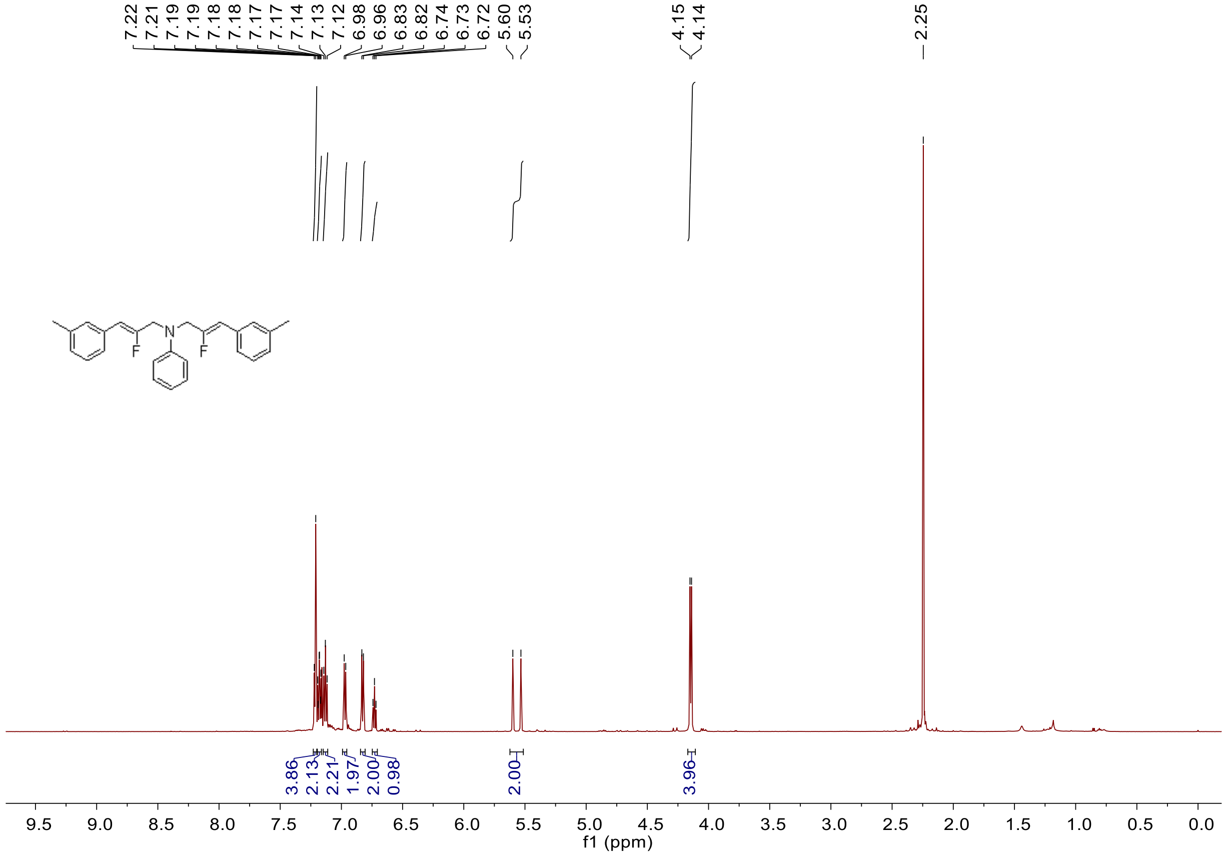

^{19}F NMR (**d6**)

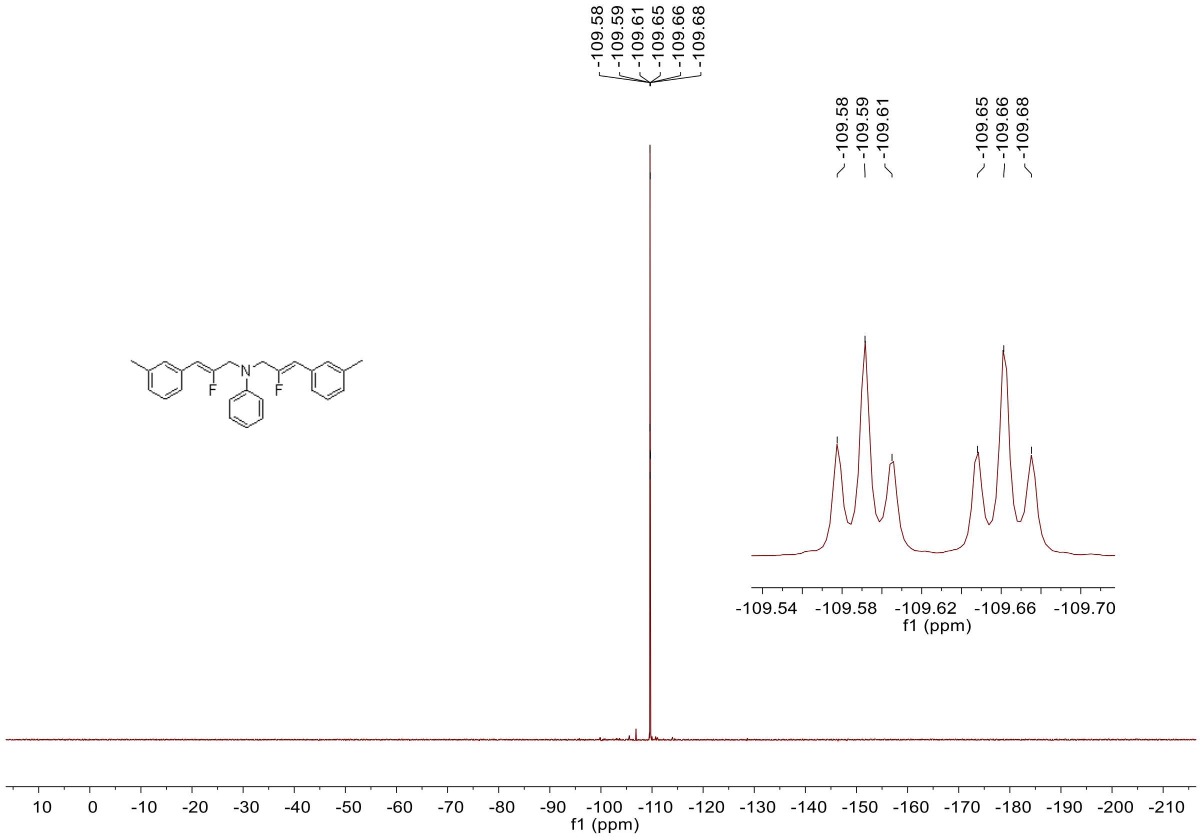

^{13}C NMR (**d6**)

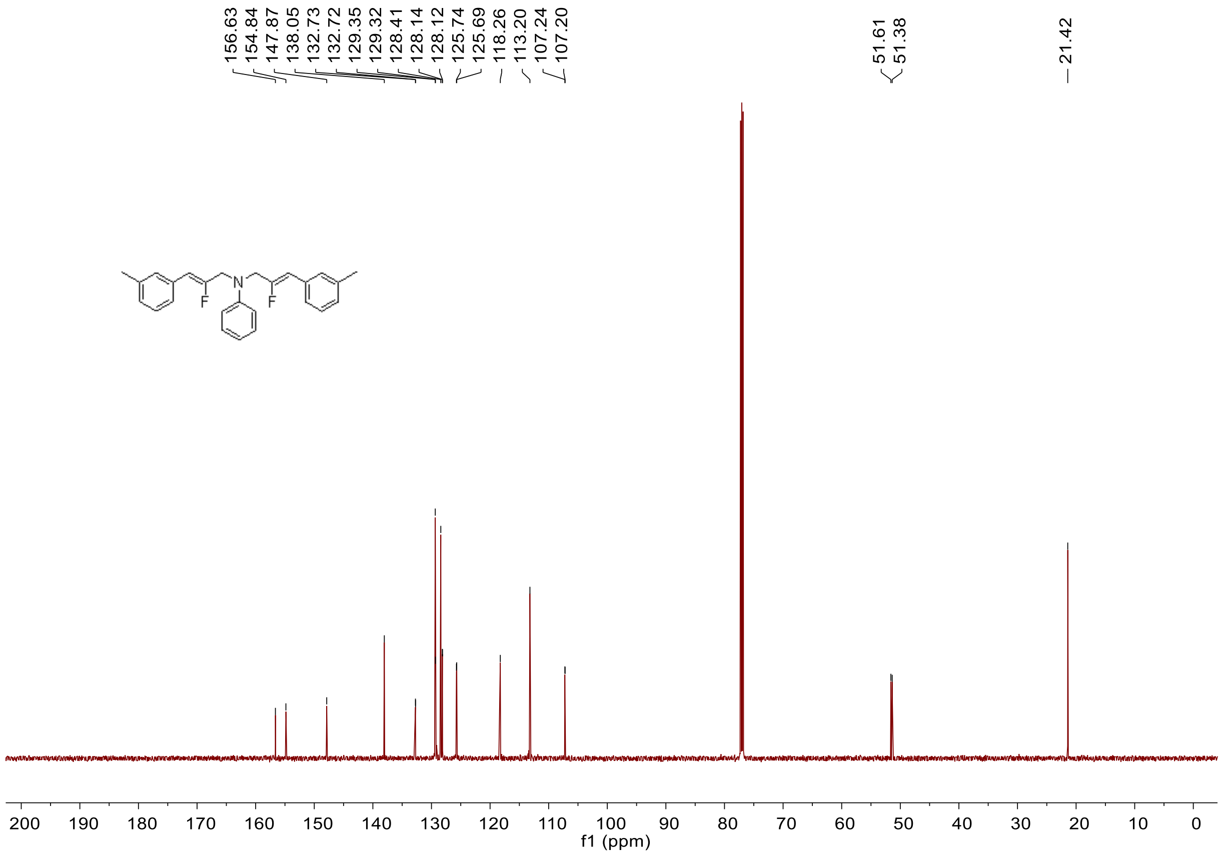

^{1}H NMR (**d7**)

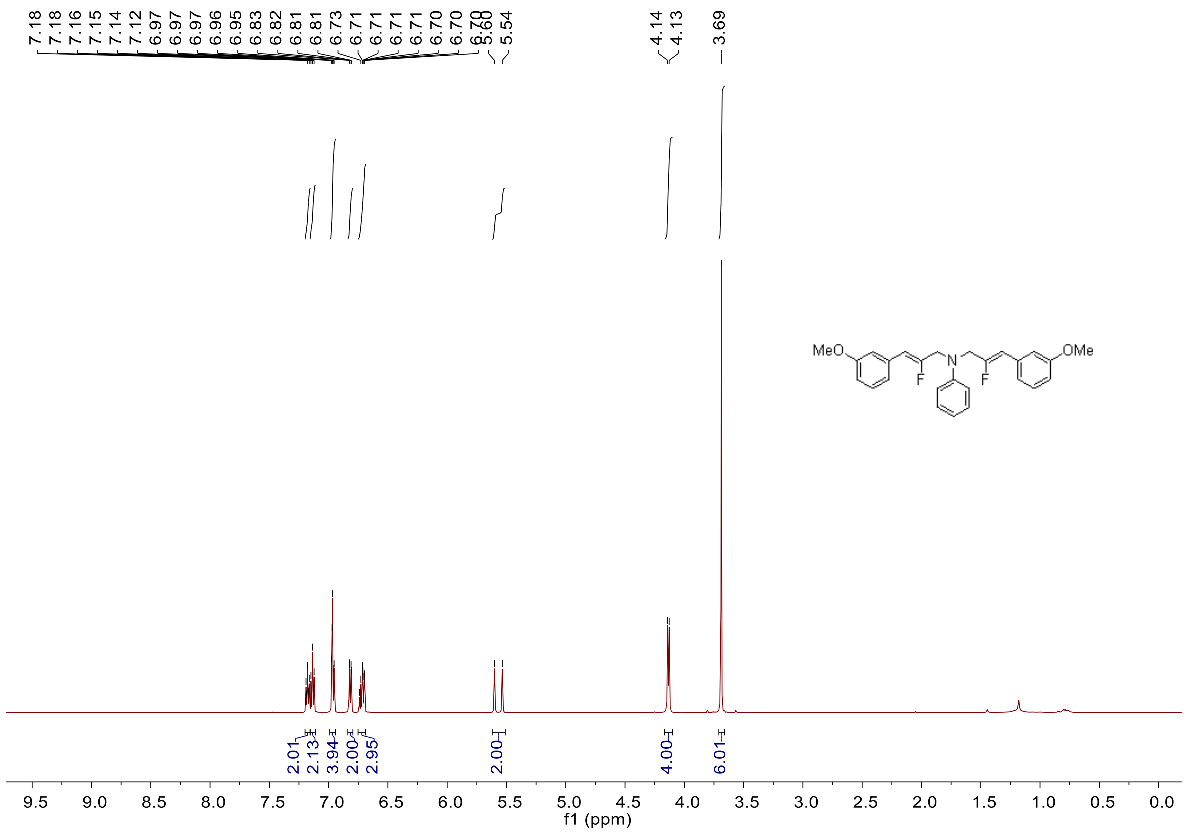

^{19}F NMR (**d7**)

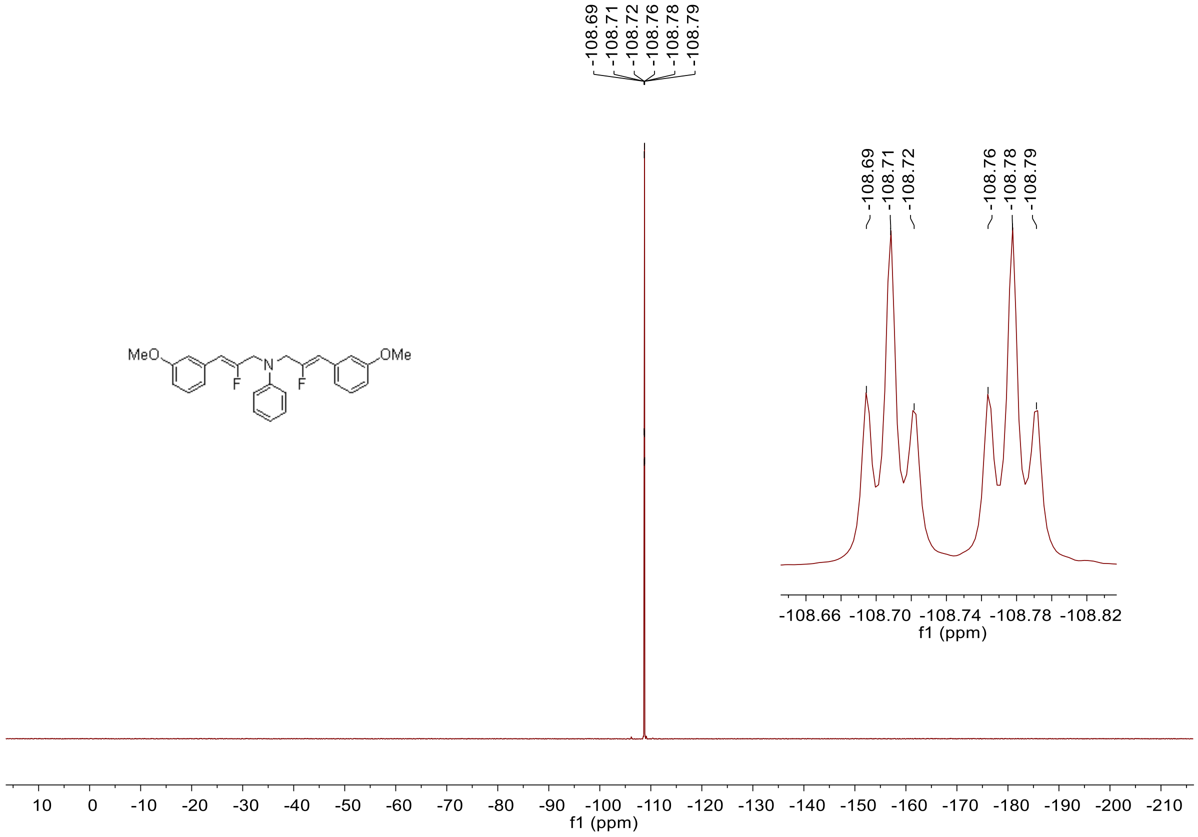

^{13}C NMR (**d7**)

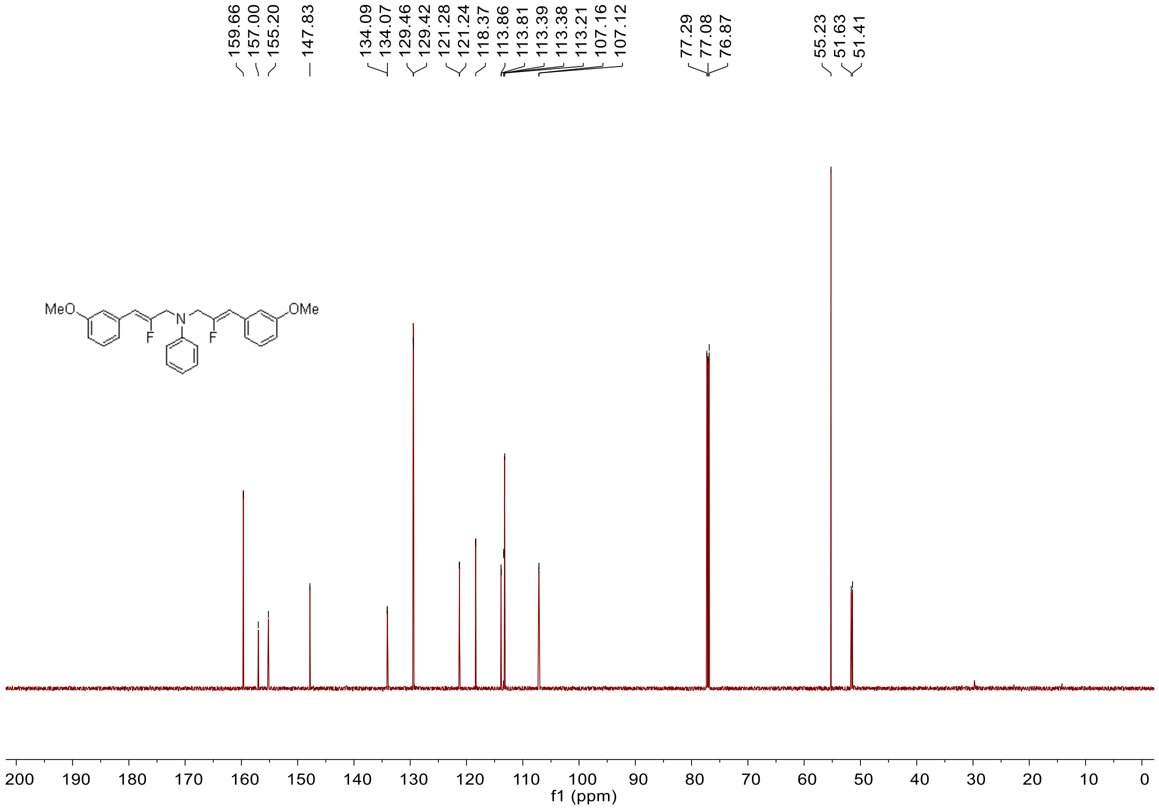

^{1}H NMR (**d8**)

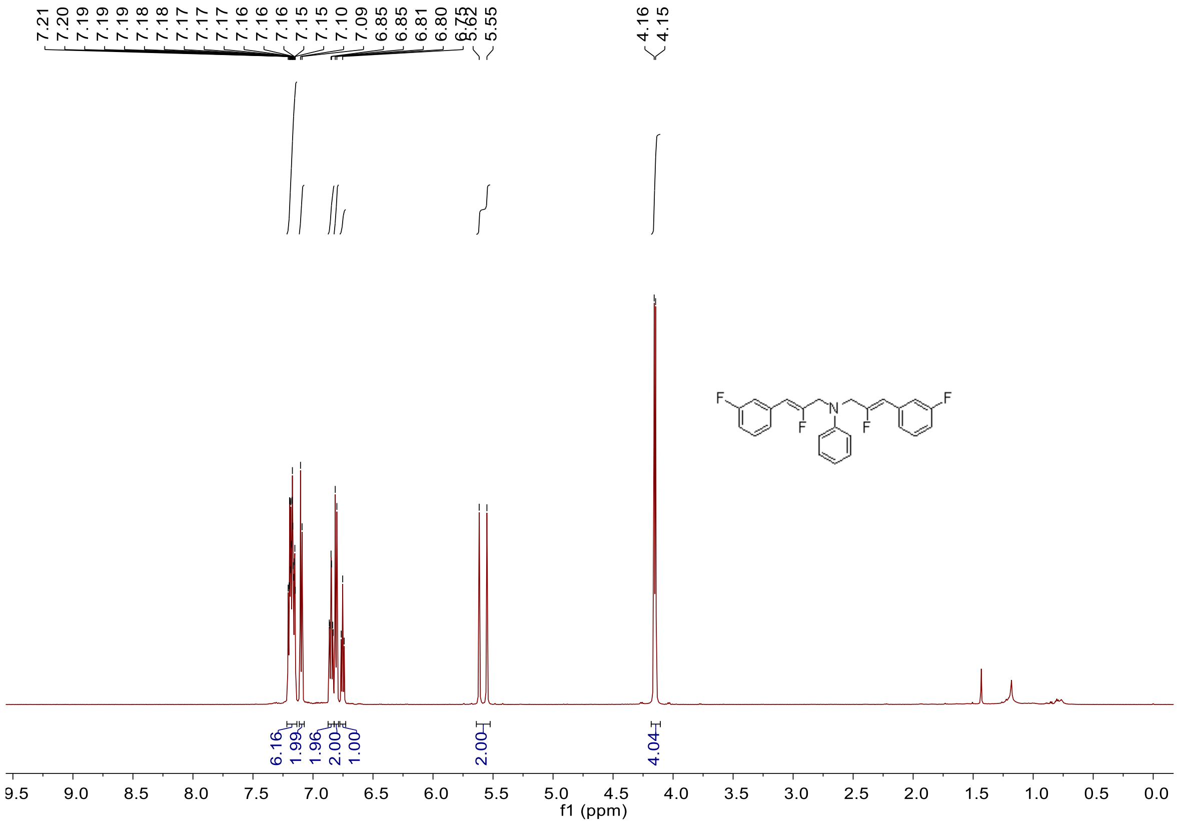

^{19}F NMR (**d8**)

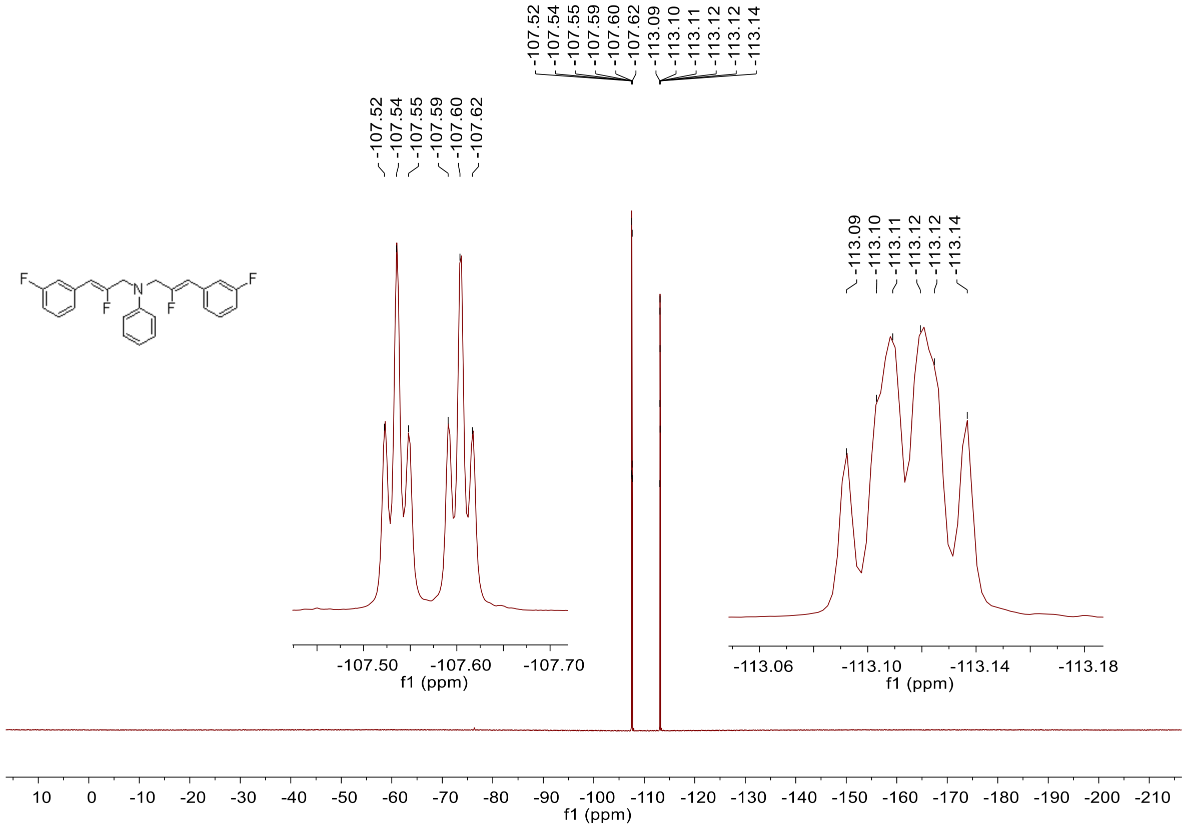

^{13}C NMR (**d8**)

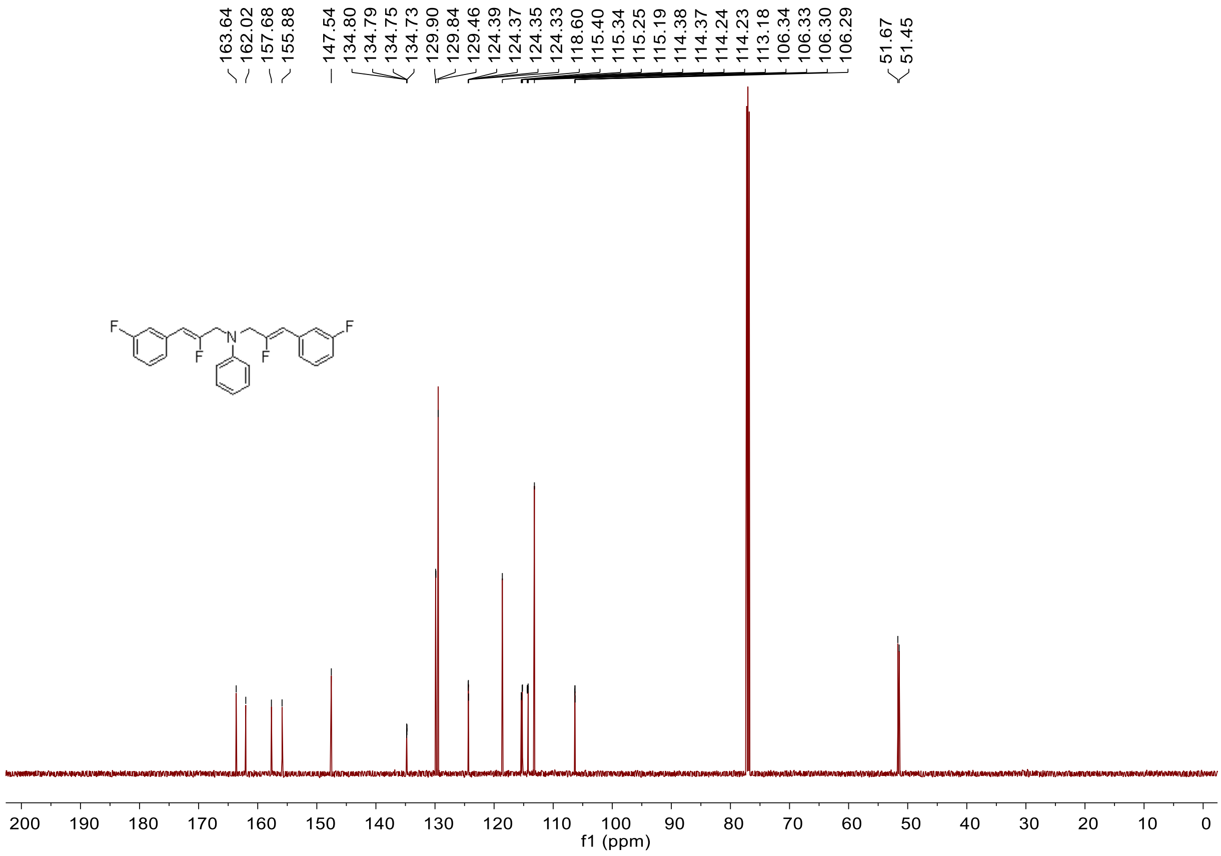

^{1}H NMR (**d9**)

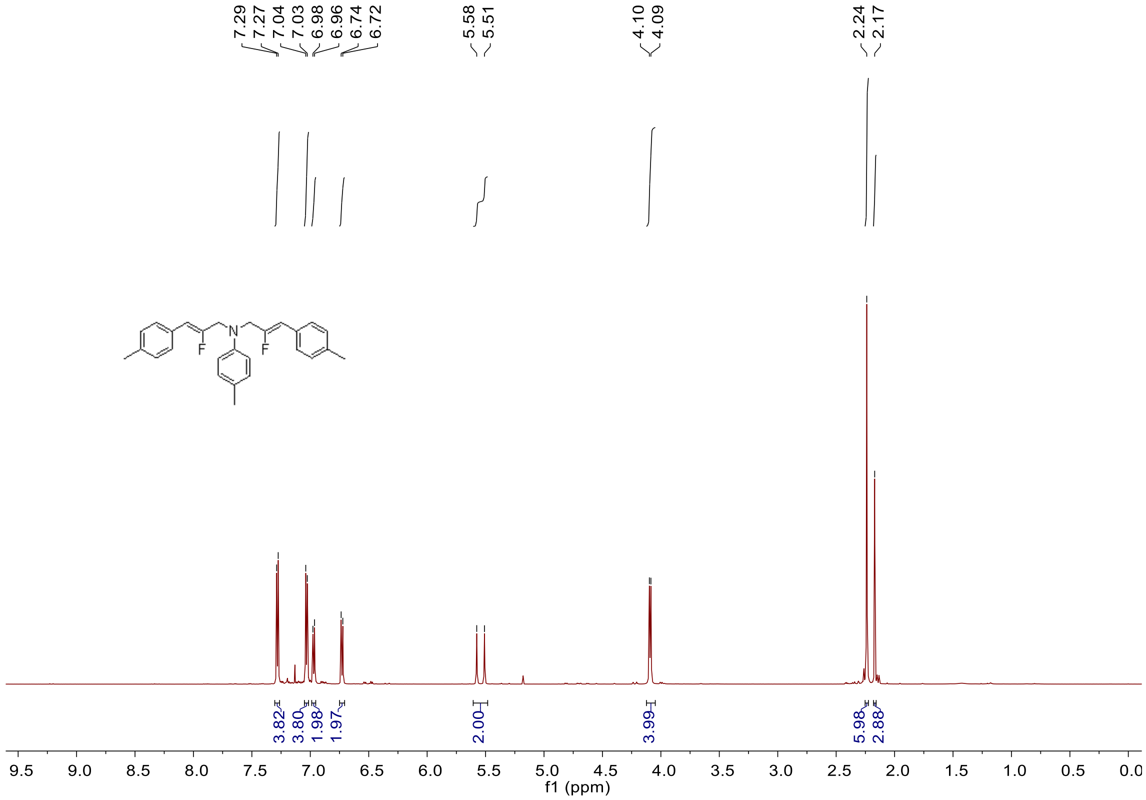

^{19}F NMR (**d9**)

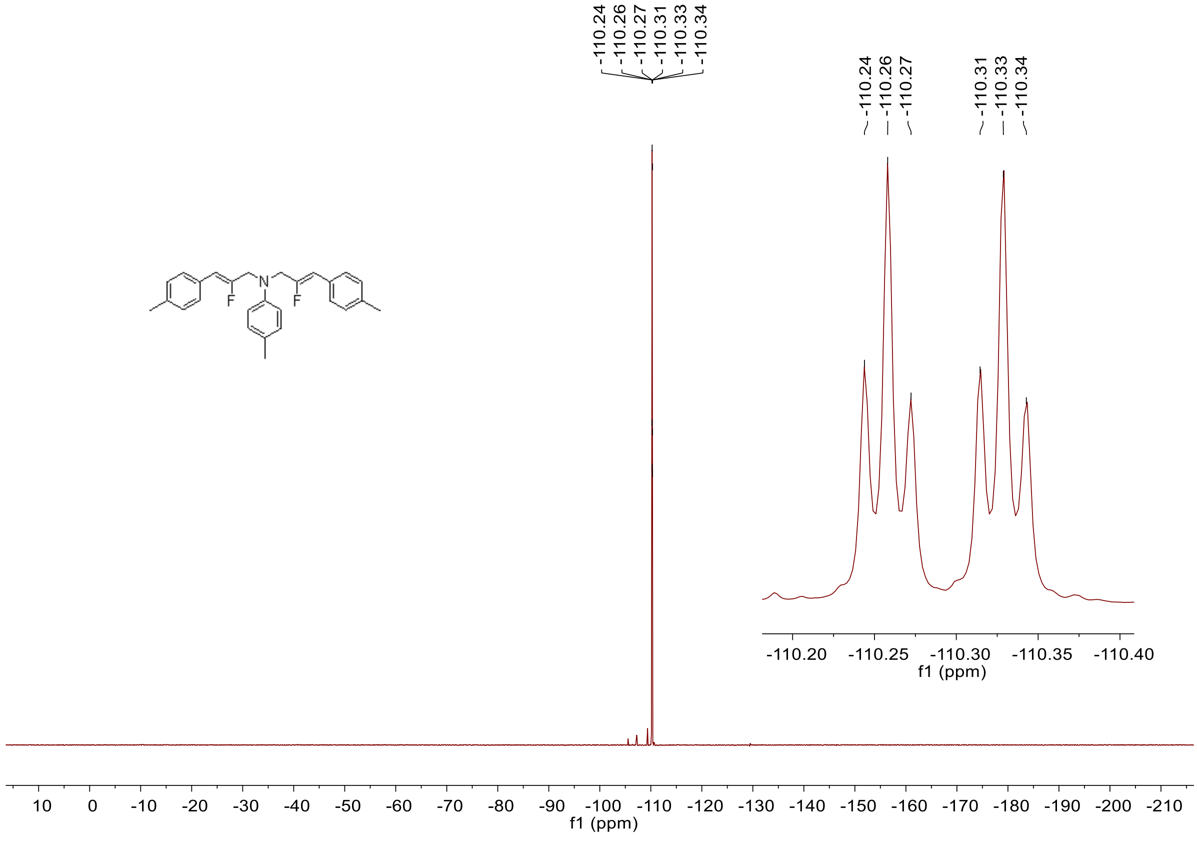

^{13}C NMR (**d9**)

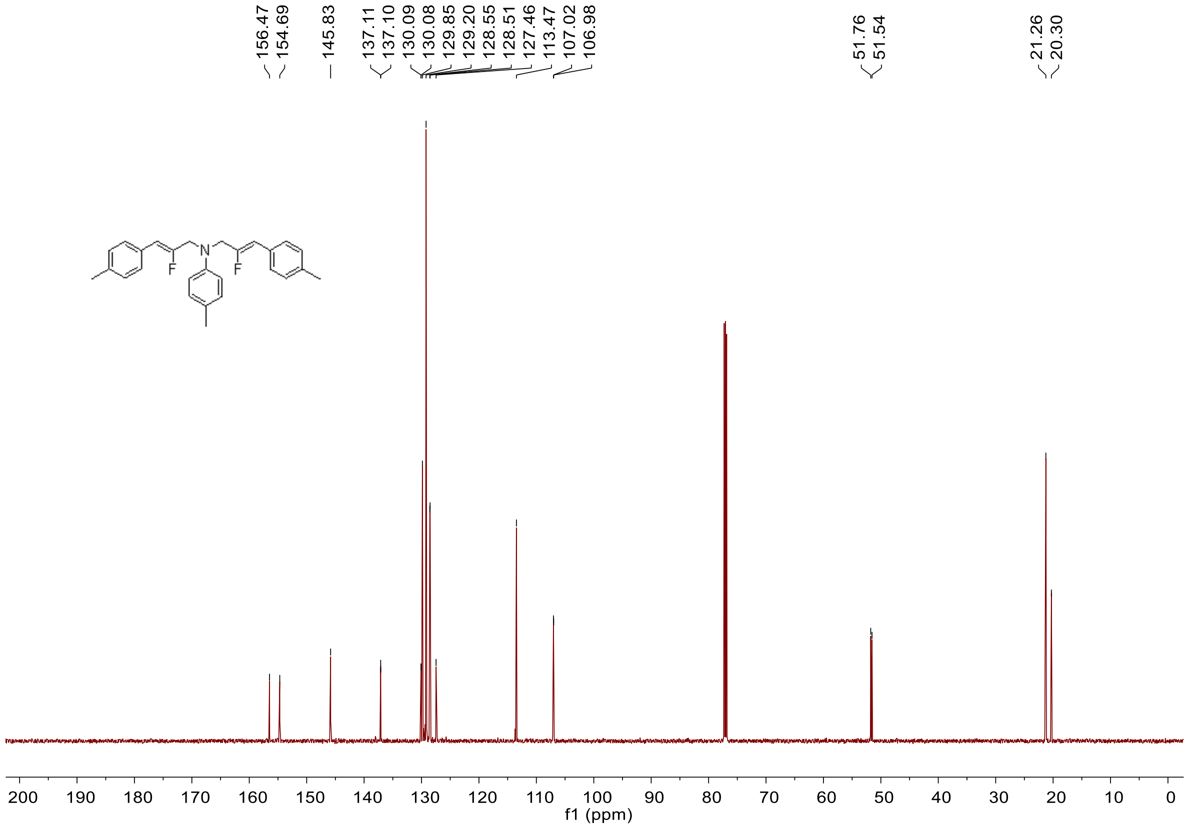

^{1}H NMR (**d10**)

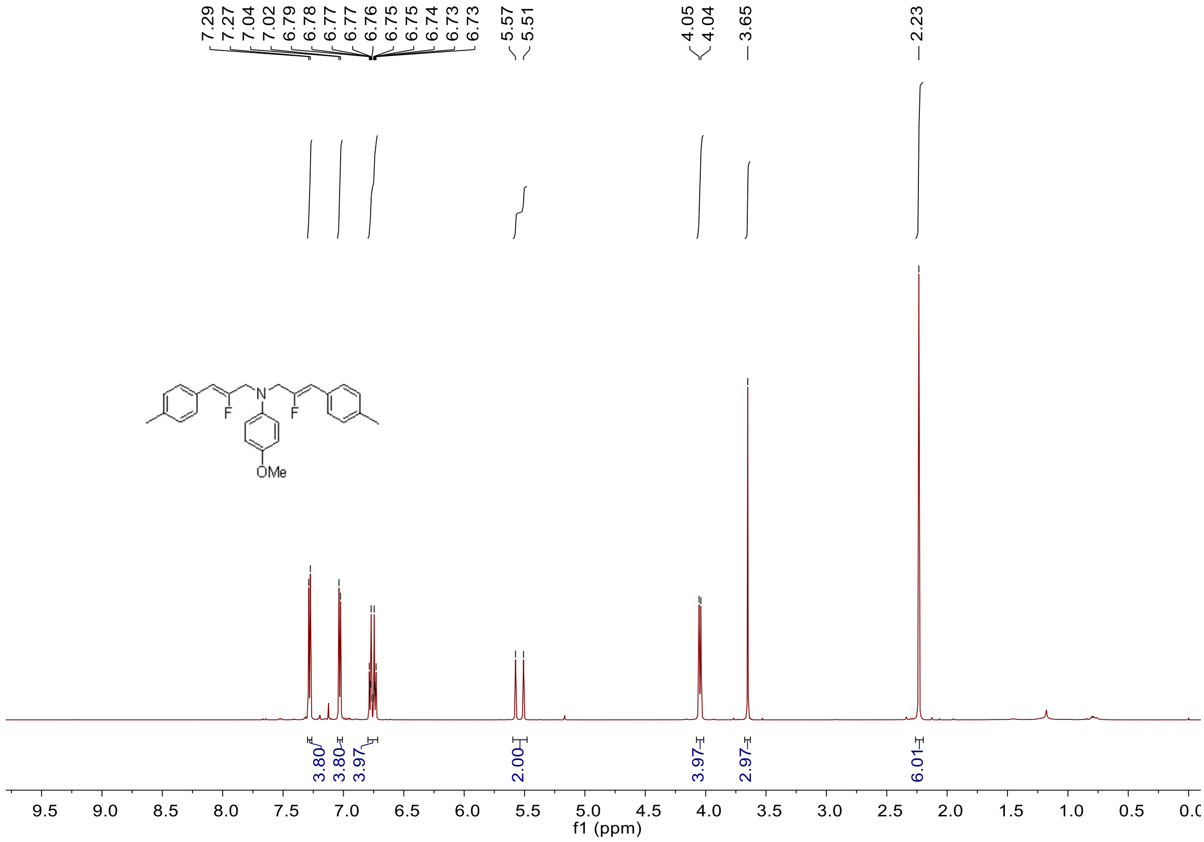

^{19}F NMR (**d10**)

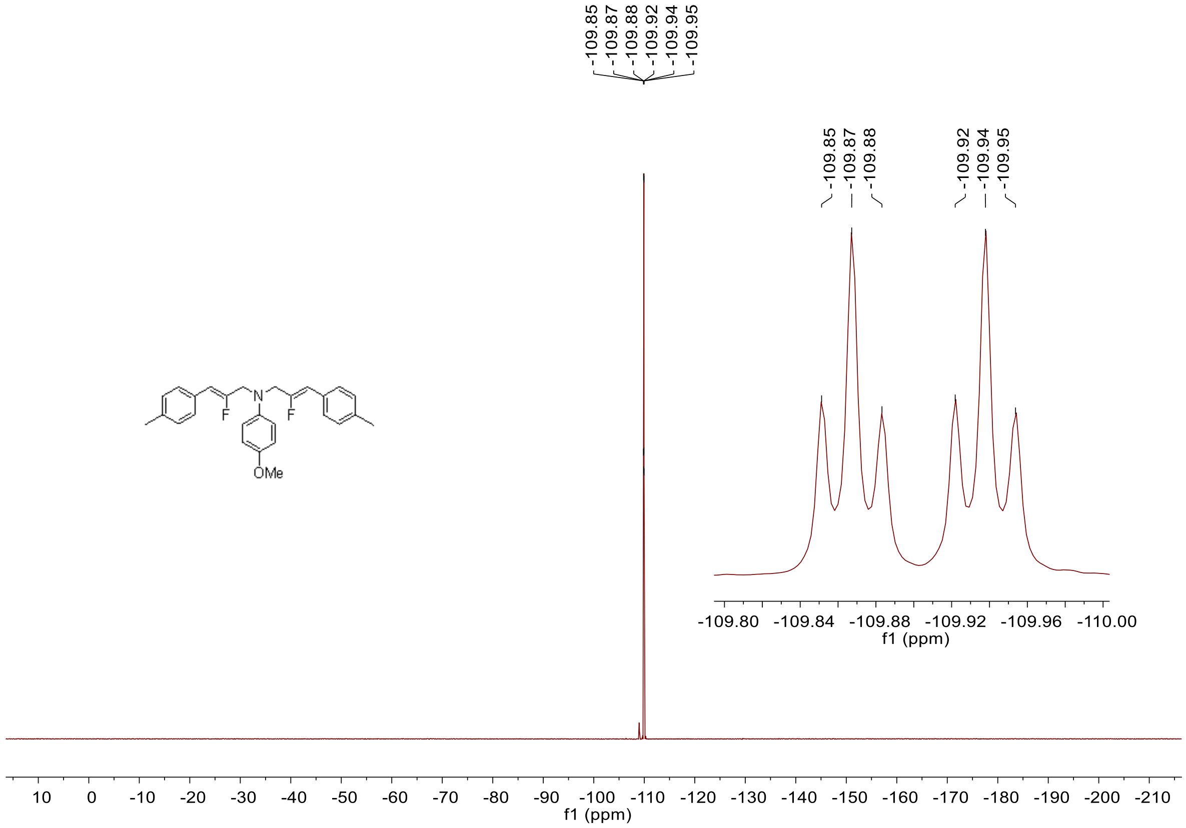

^{13}C NMR (**d10**)

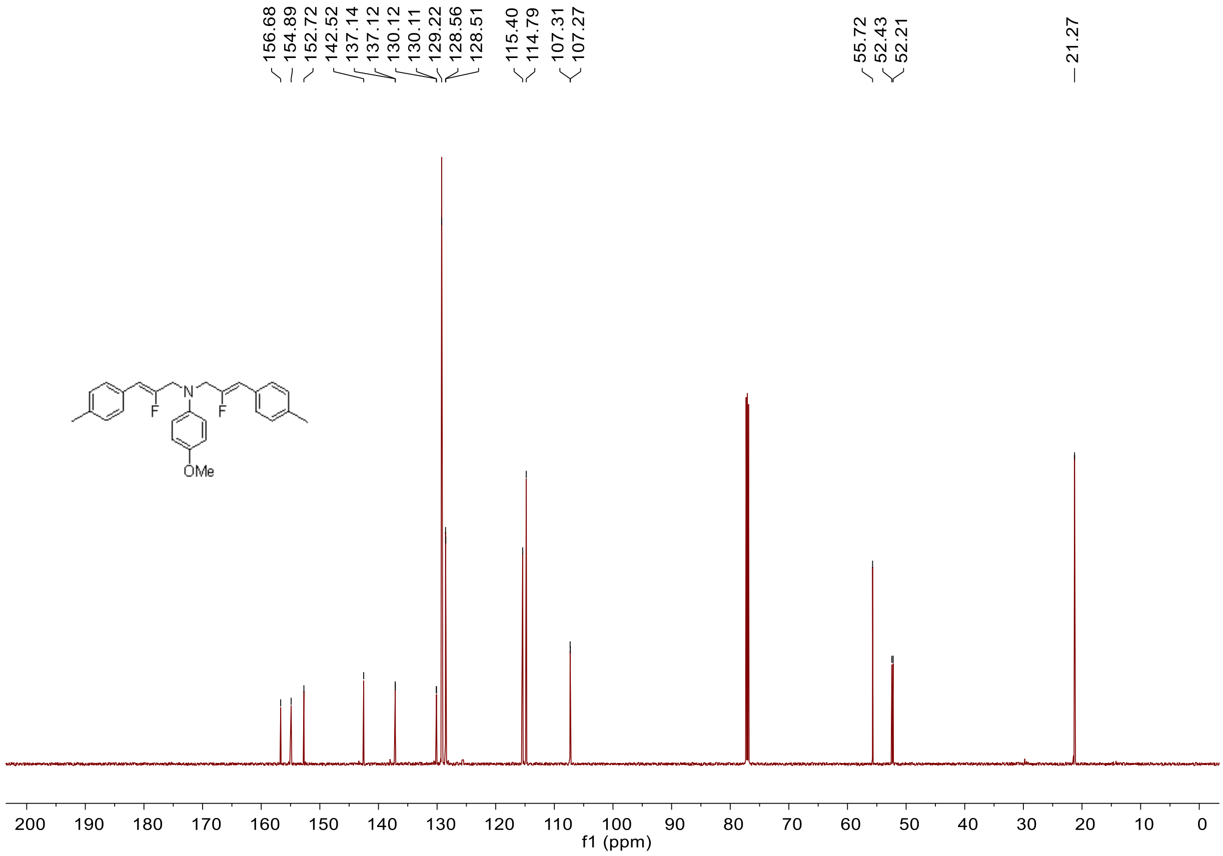

^{1}H NMR (**d11**)

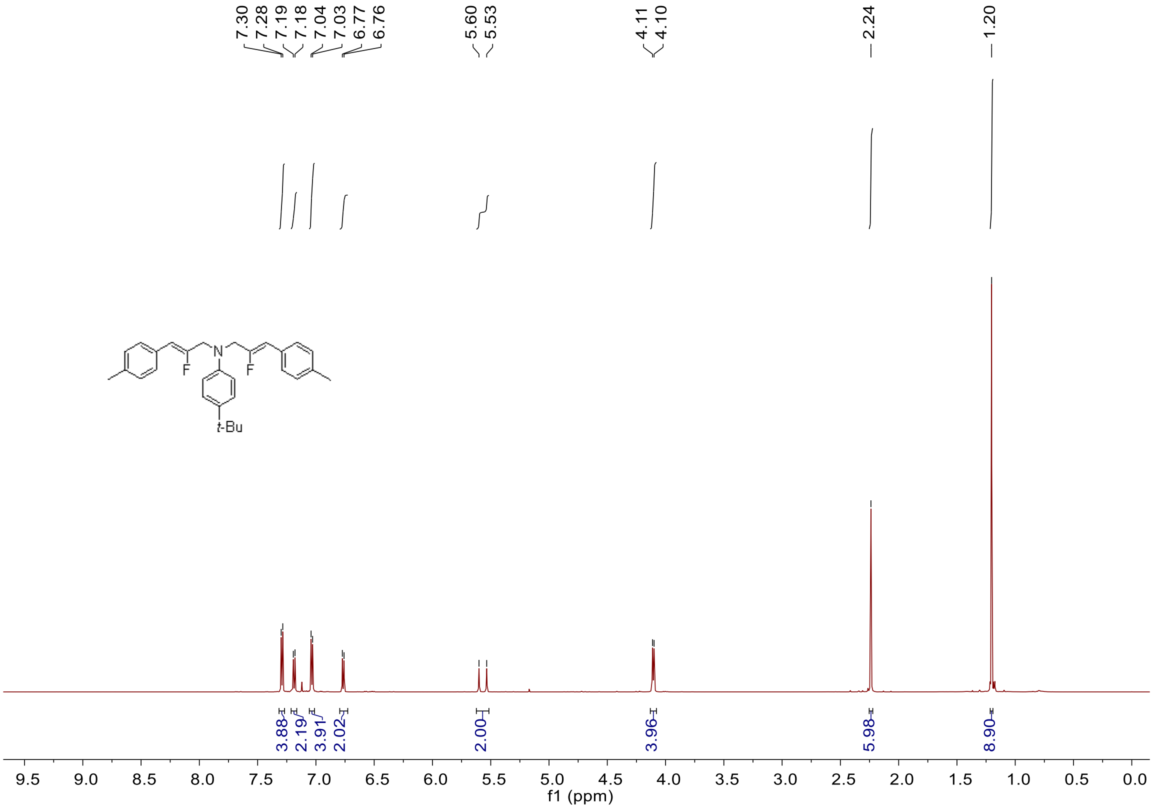

^{19}F NMR (**d11**)

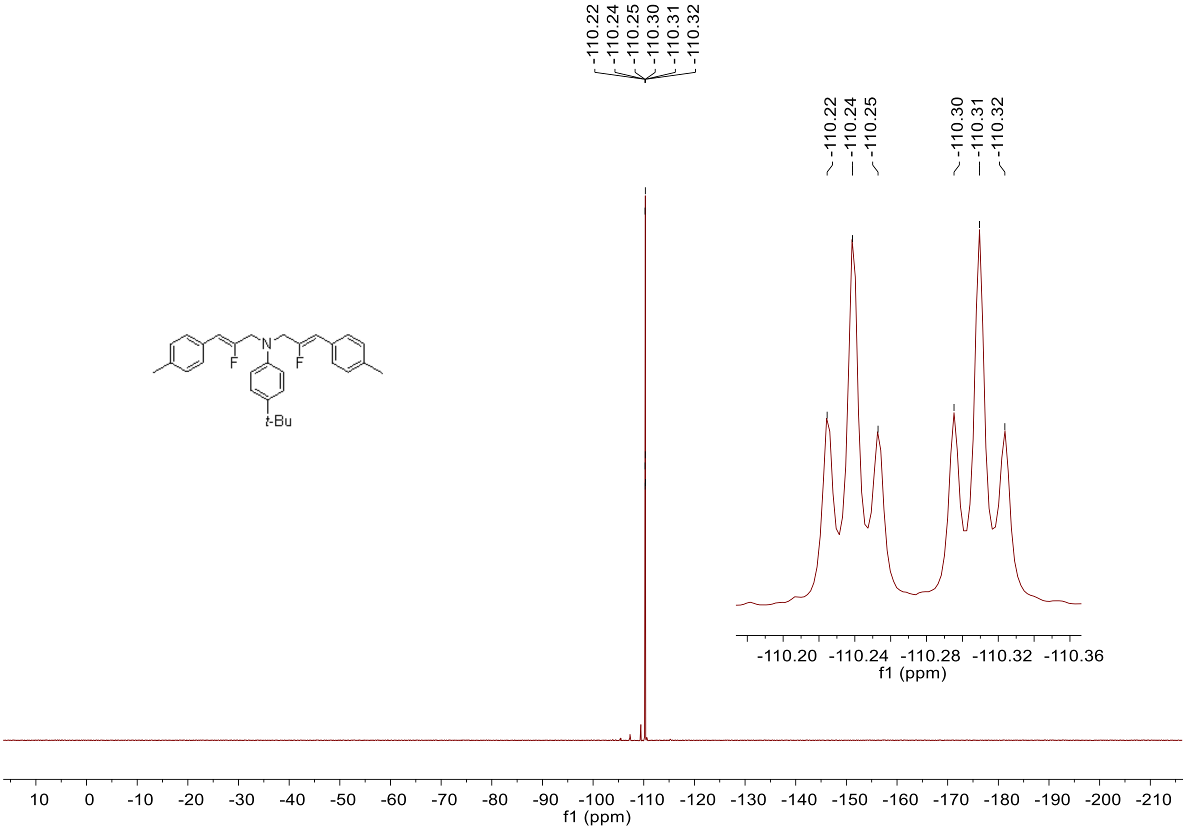

^{13}C NMR (**d11**)

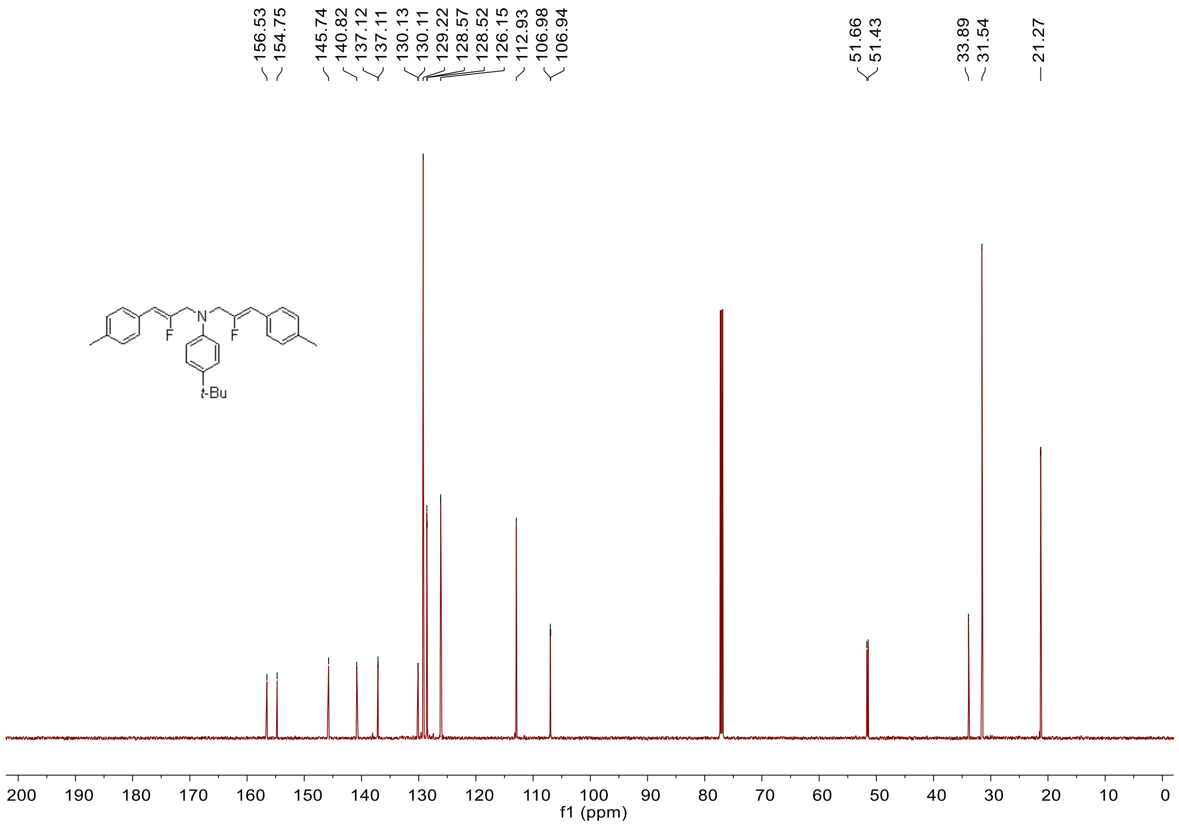

^{1}H NMR (**d12**)

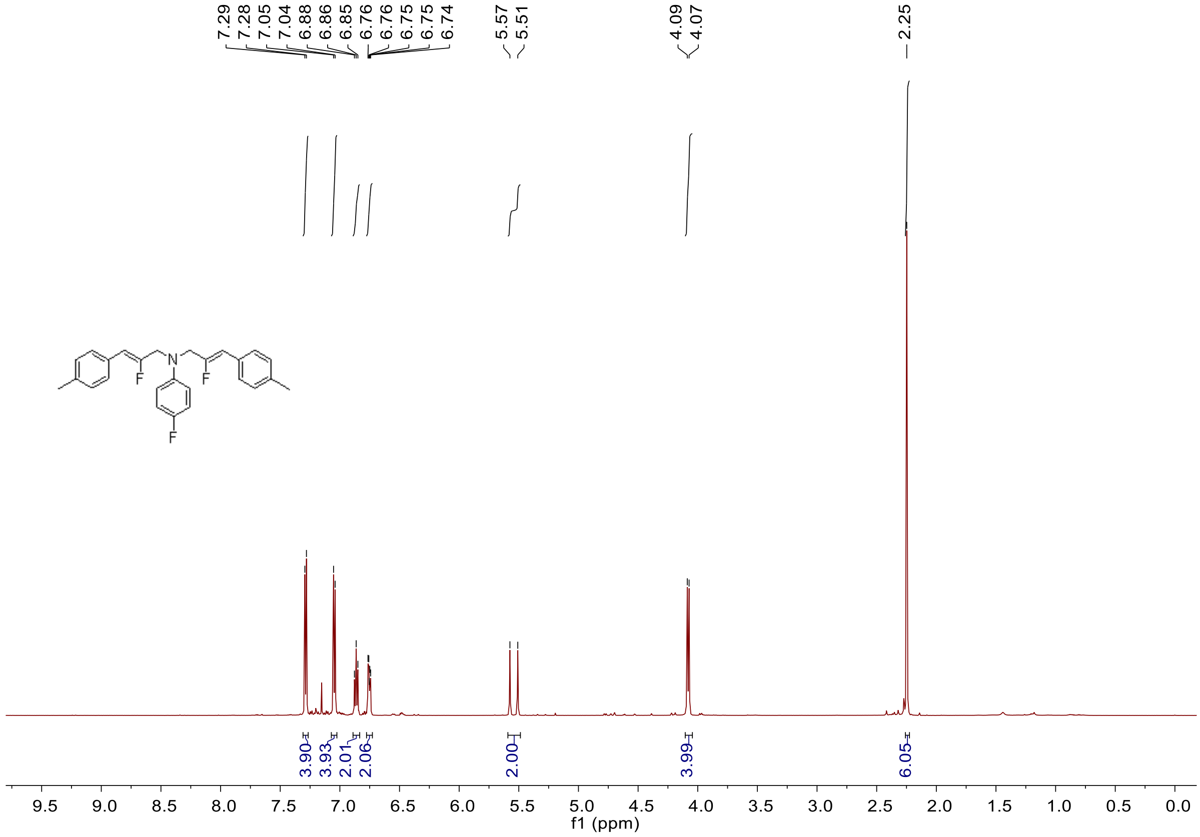

^{19}F NMR (**d12**)

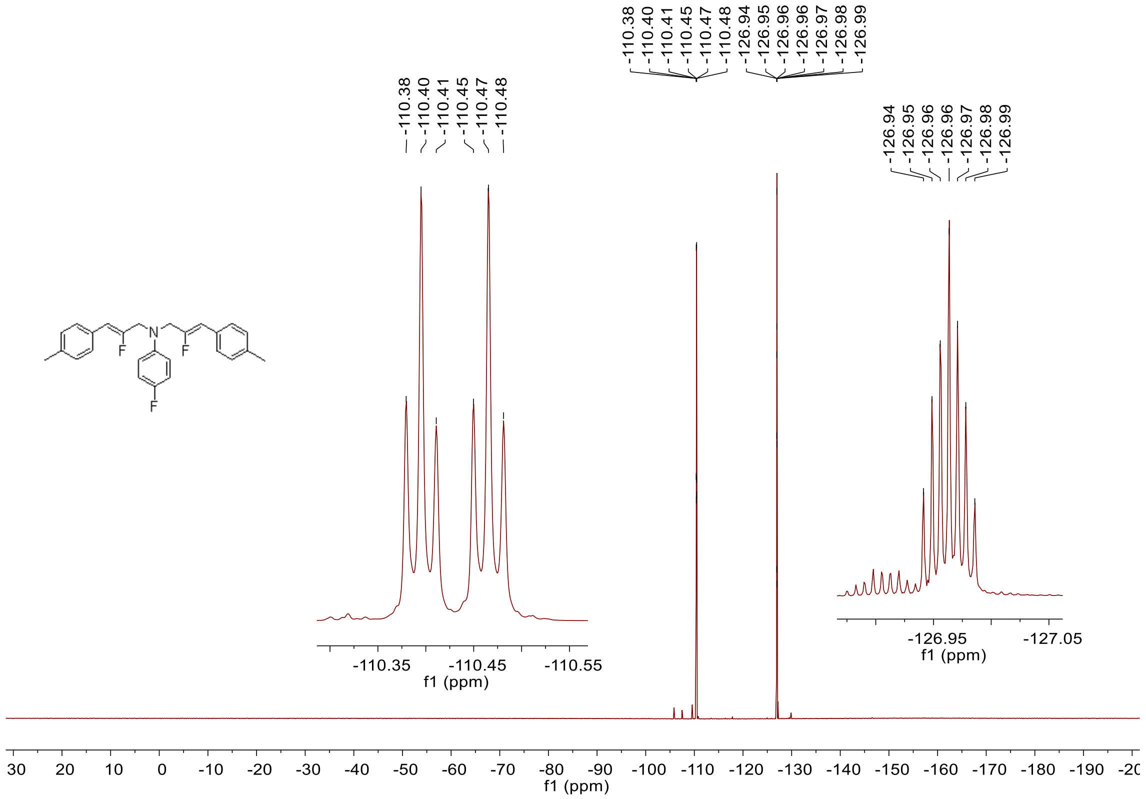

^{13}C NMR (**d12**)

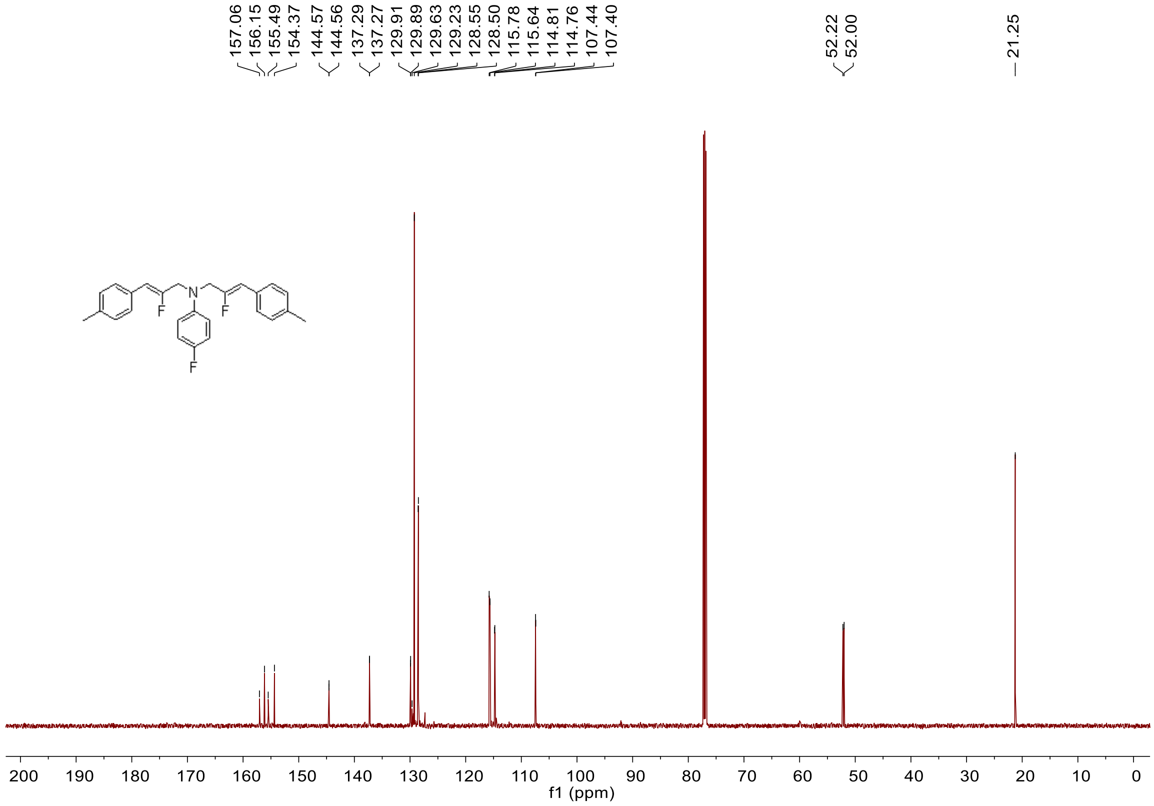

^{1}H NMR (**d13**)

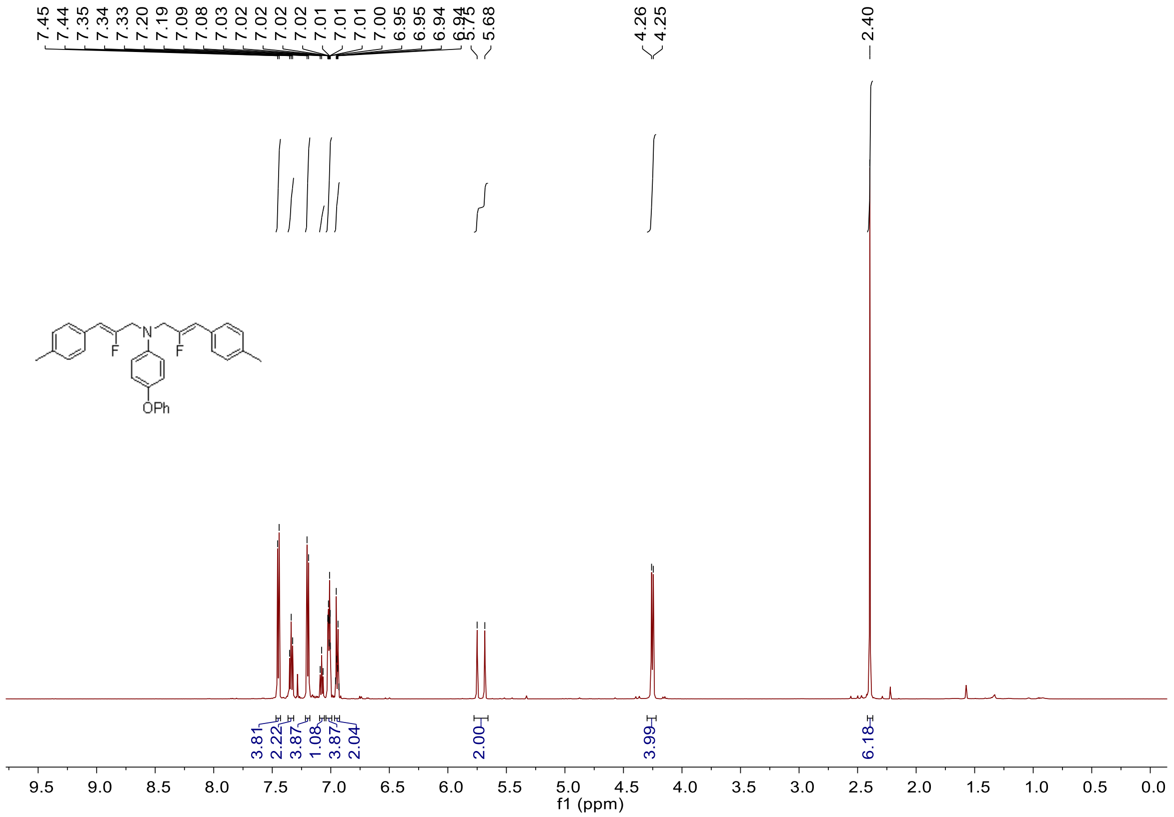

^{19}F NMR (**d13**)

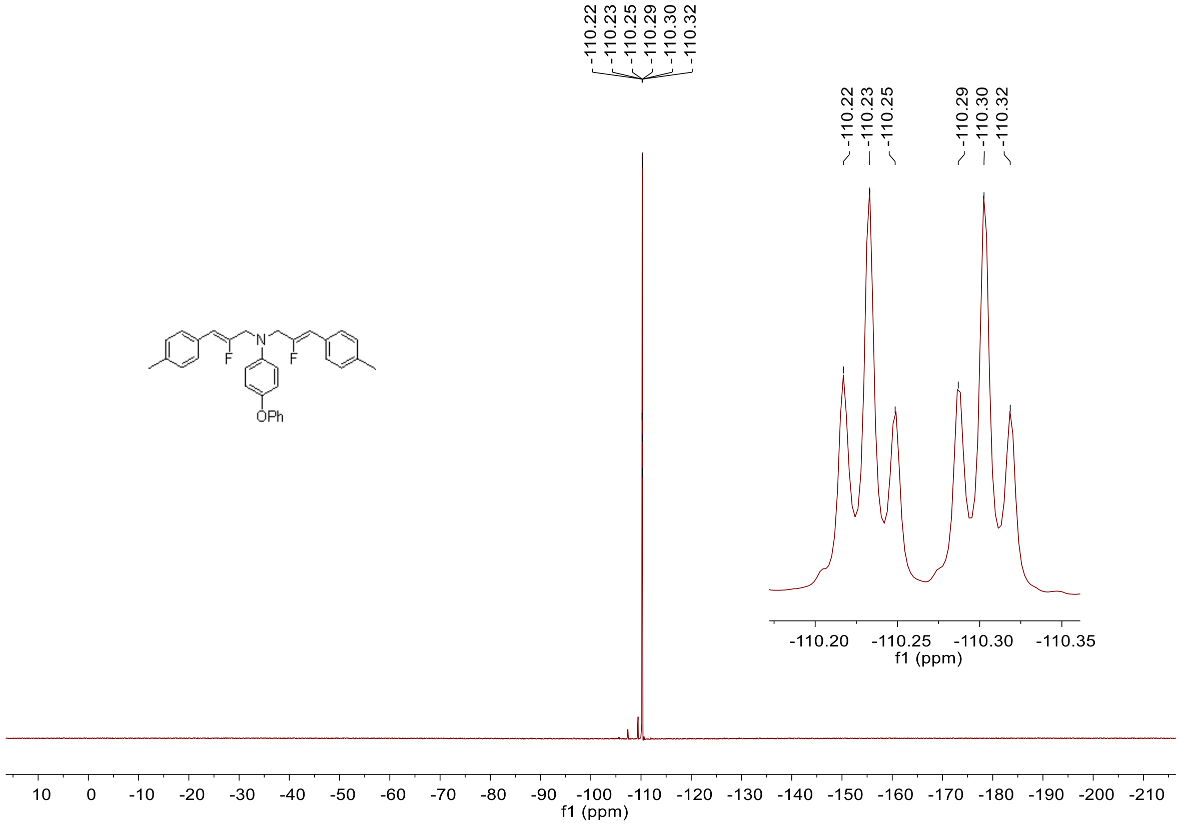

^{13}C NMR (**d13**)

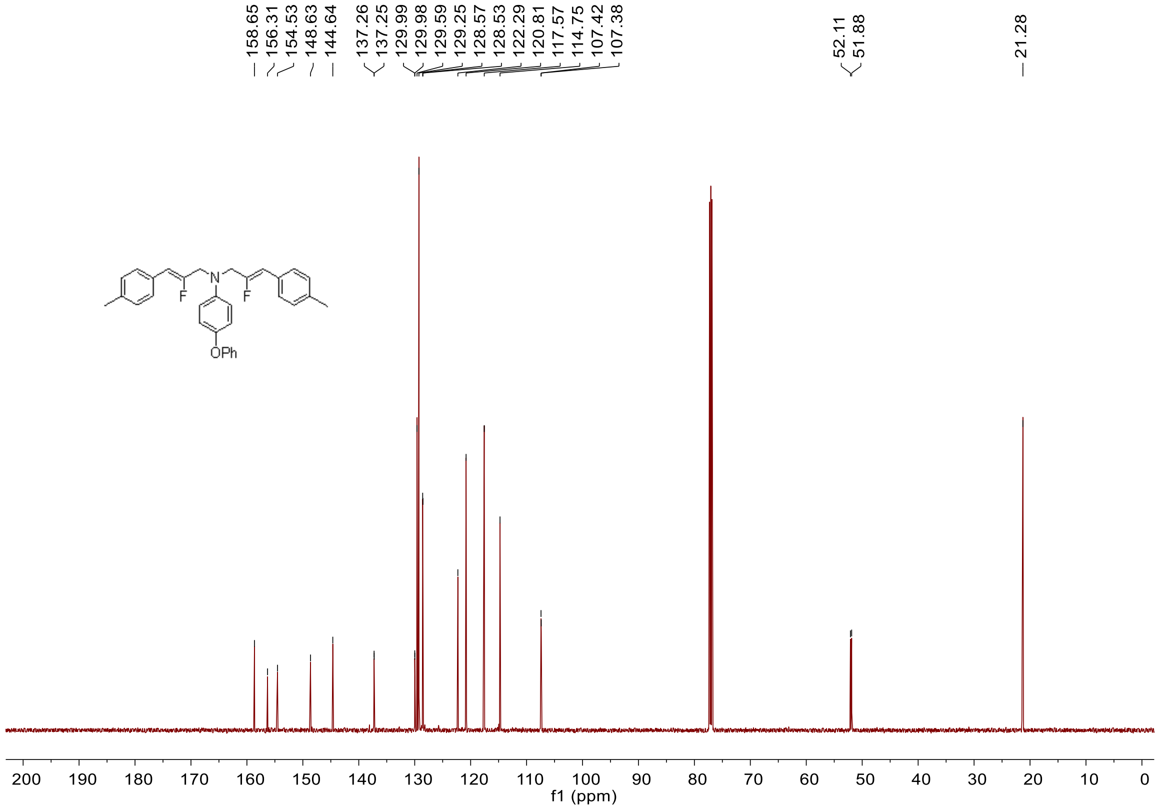

^{1}H NMR (**d14**)

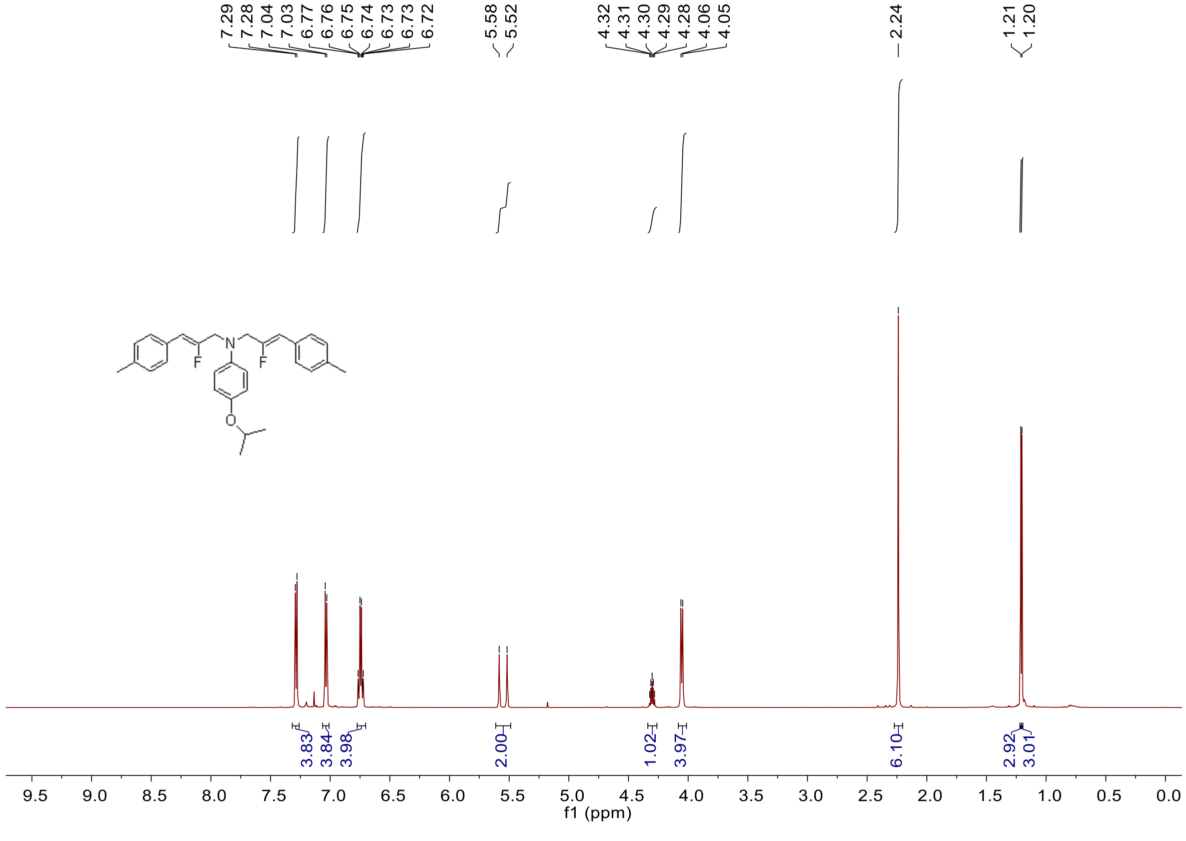

^{19}F NMR (**d14**)

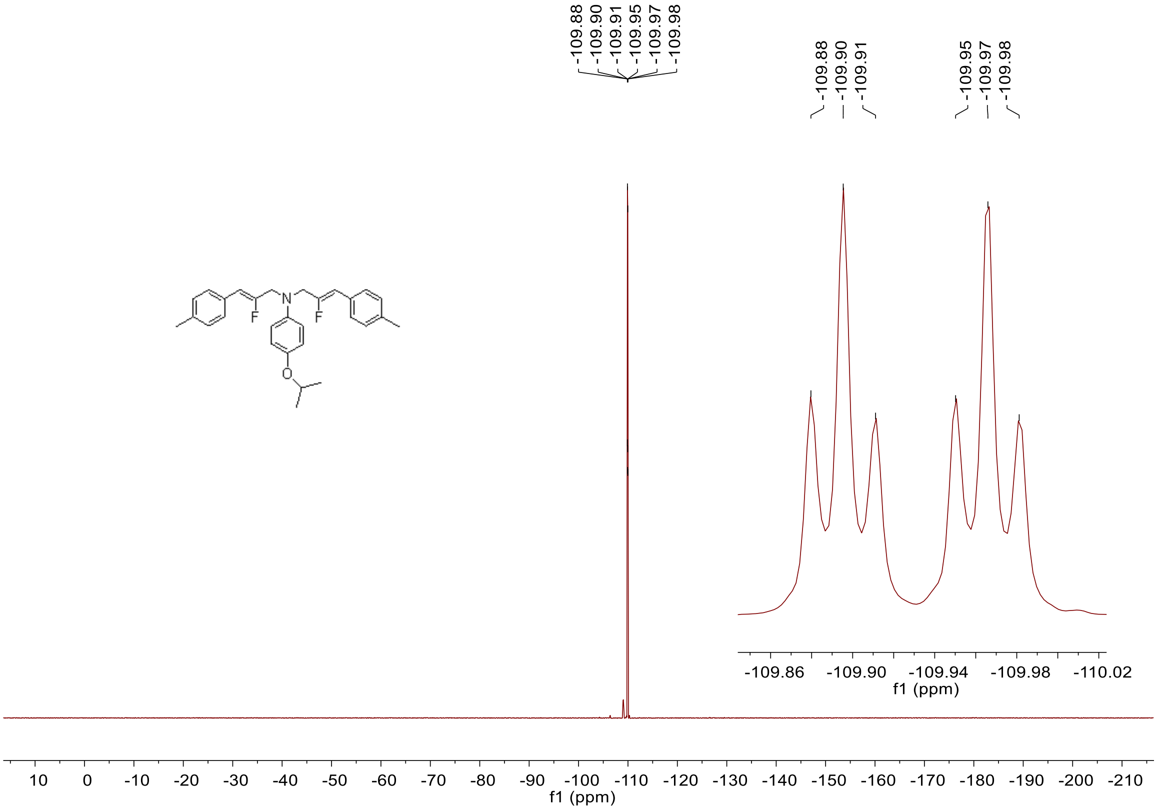

^{13}C NMR (**d14**)

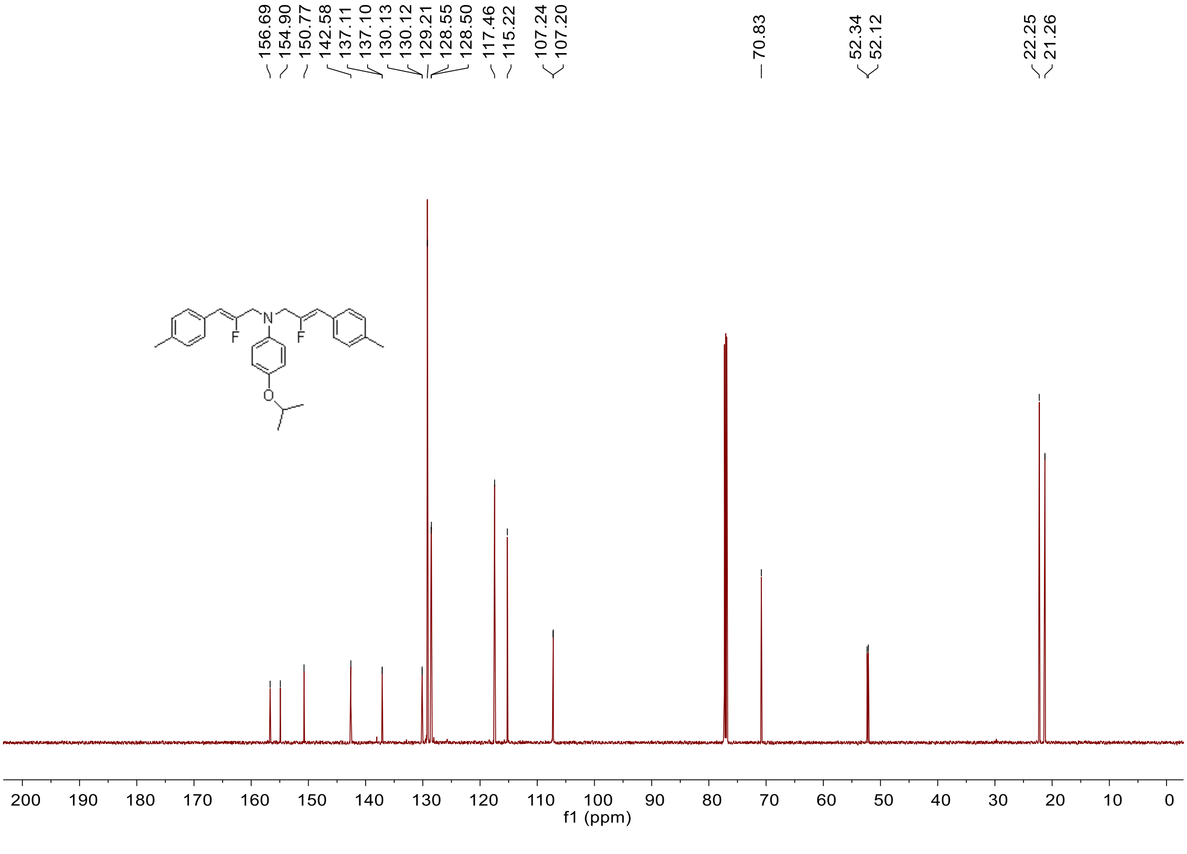

^{1}H NMR (**d15**)

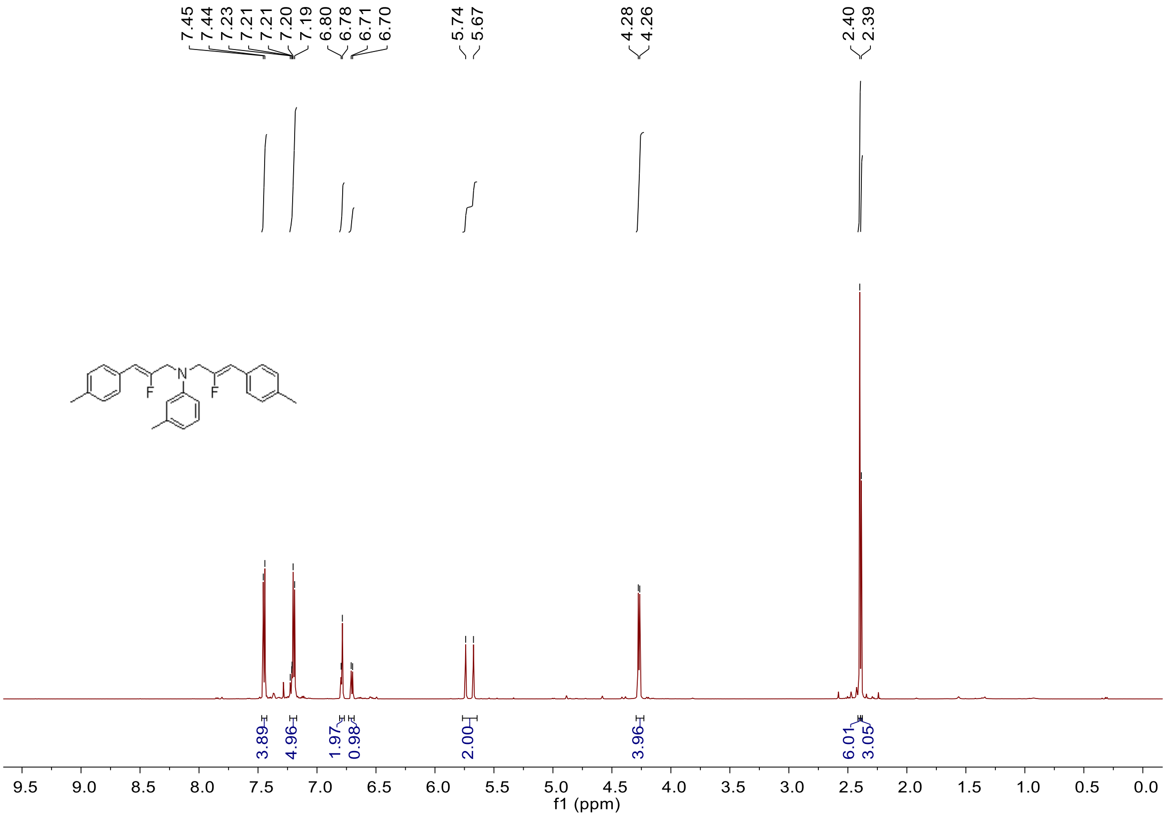

^{19}F NMR (**d15**)

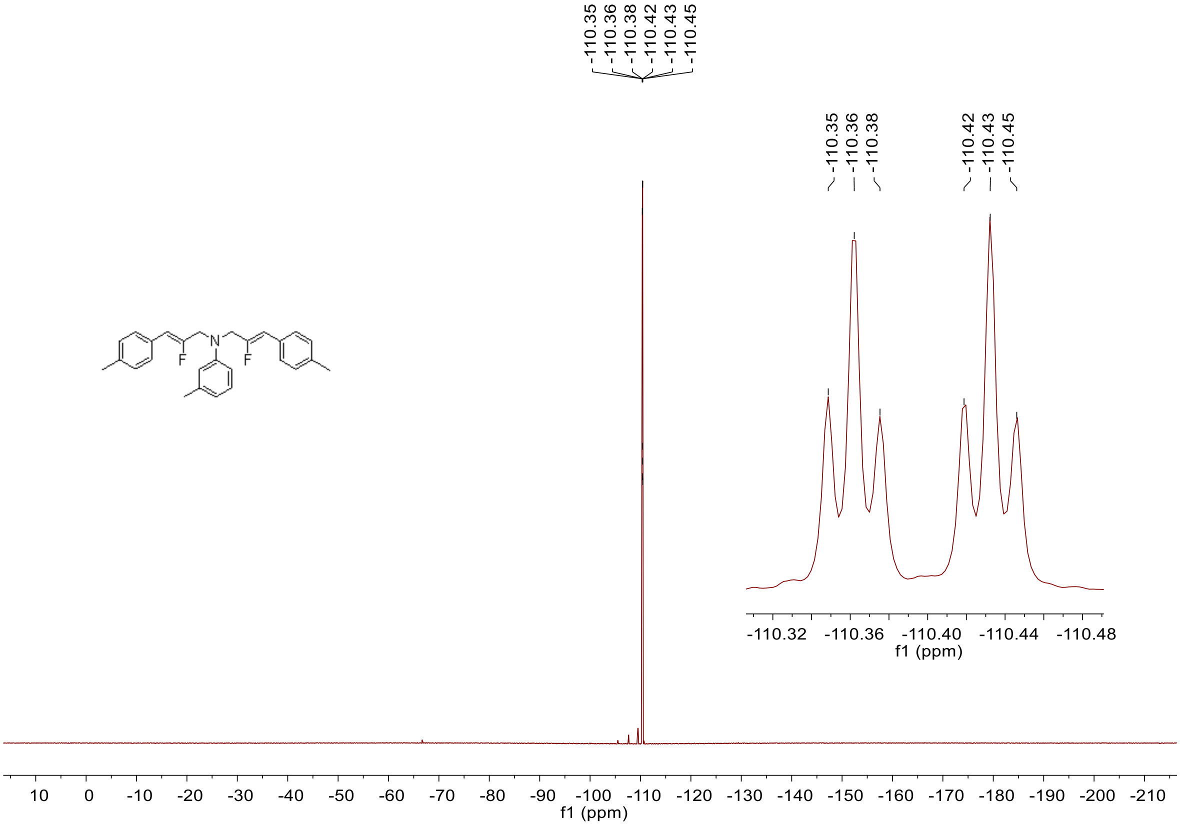

^{13}C NMR (**d15**)

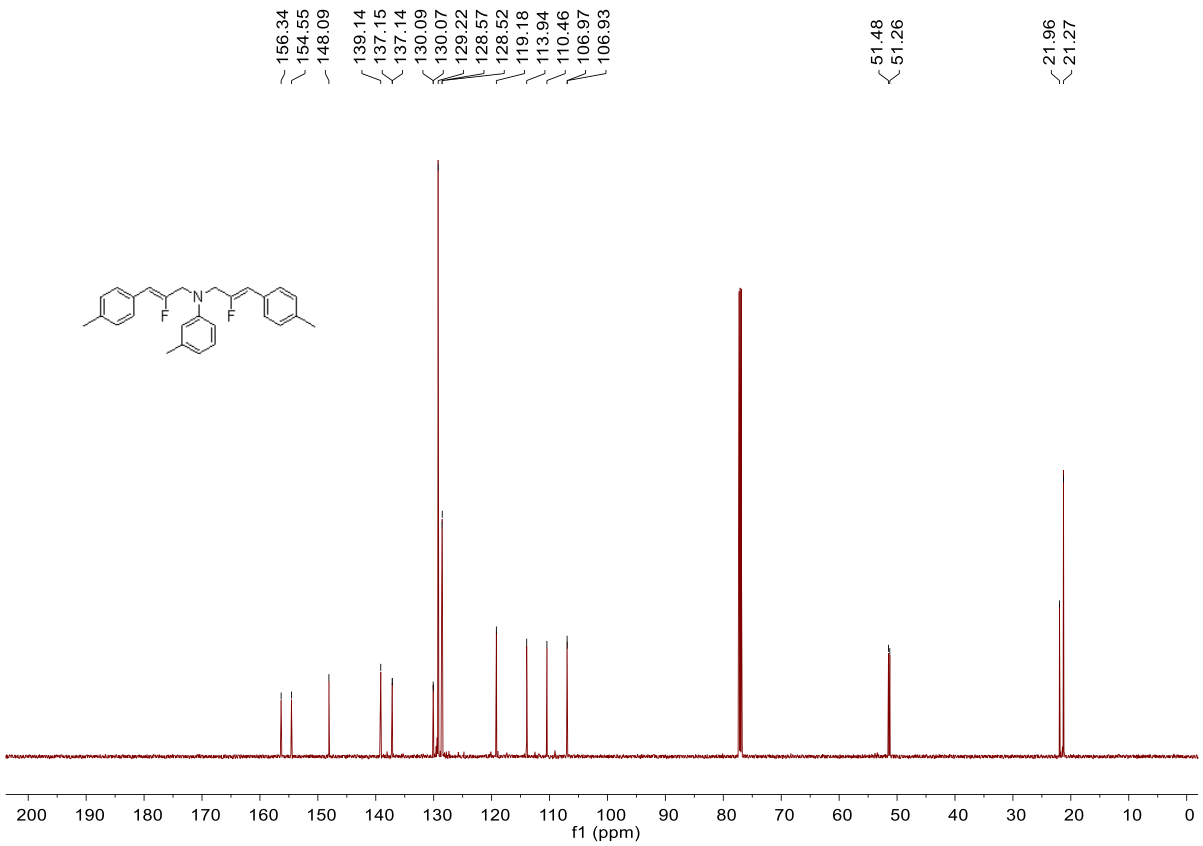

^{1}H NMR (**d16**)

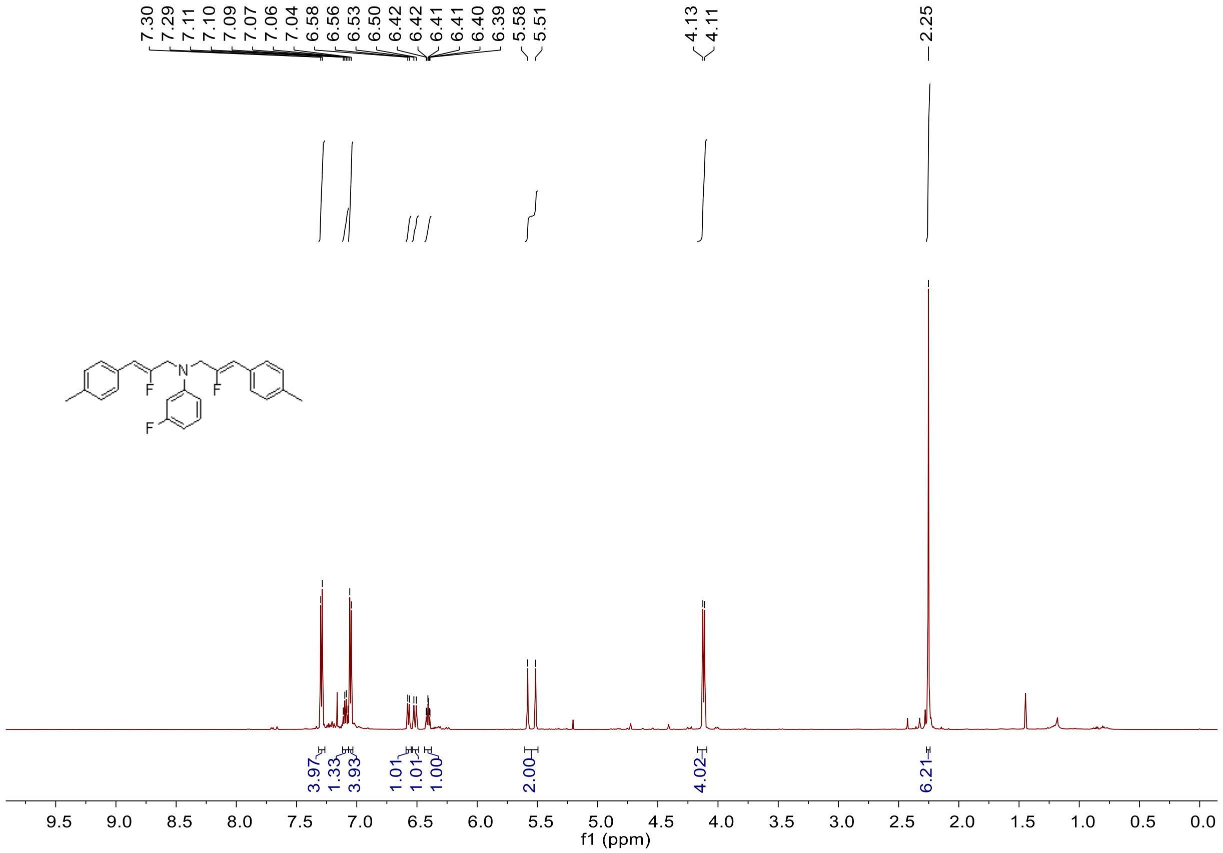

^{19}F NMR (**d16**)

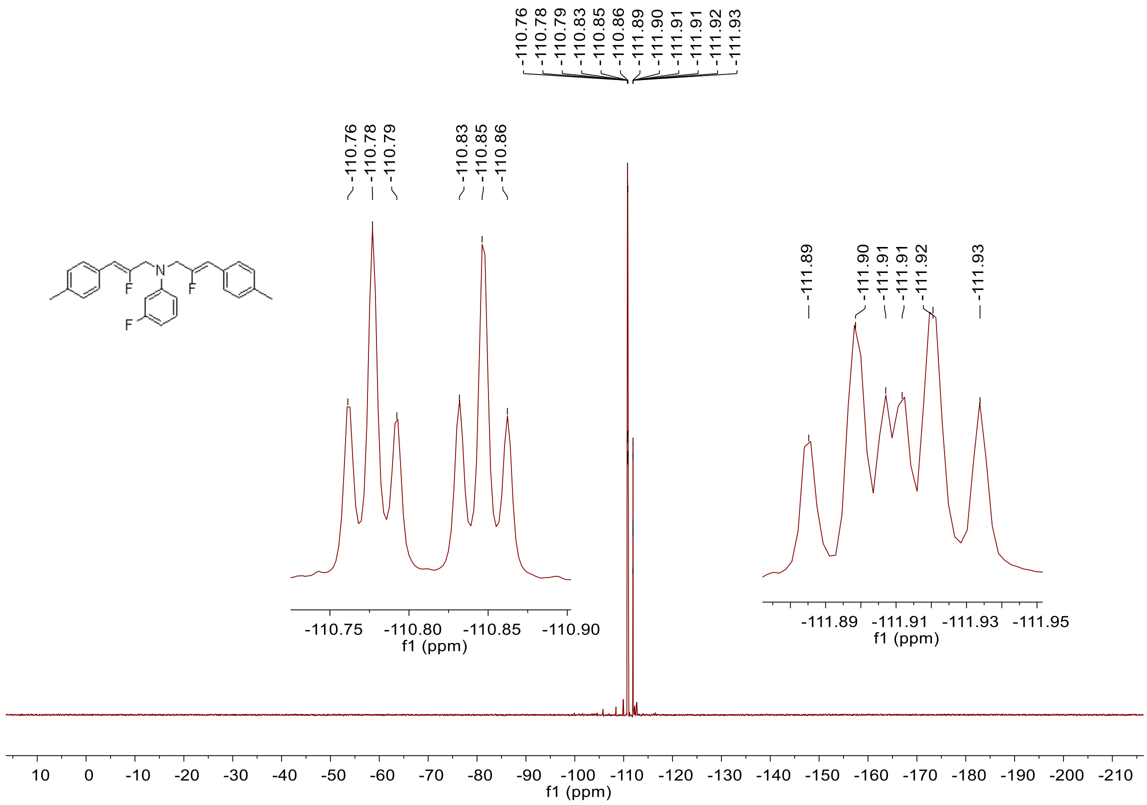

^{13}C NMR (**d16**)

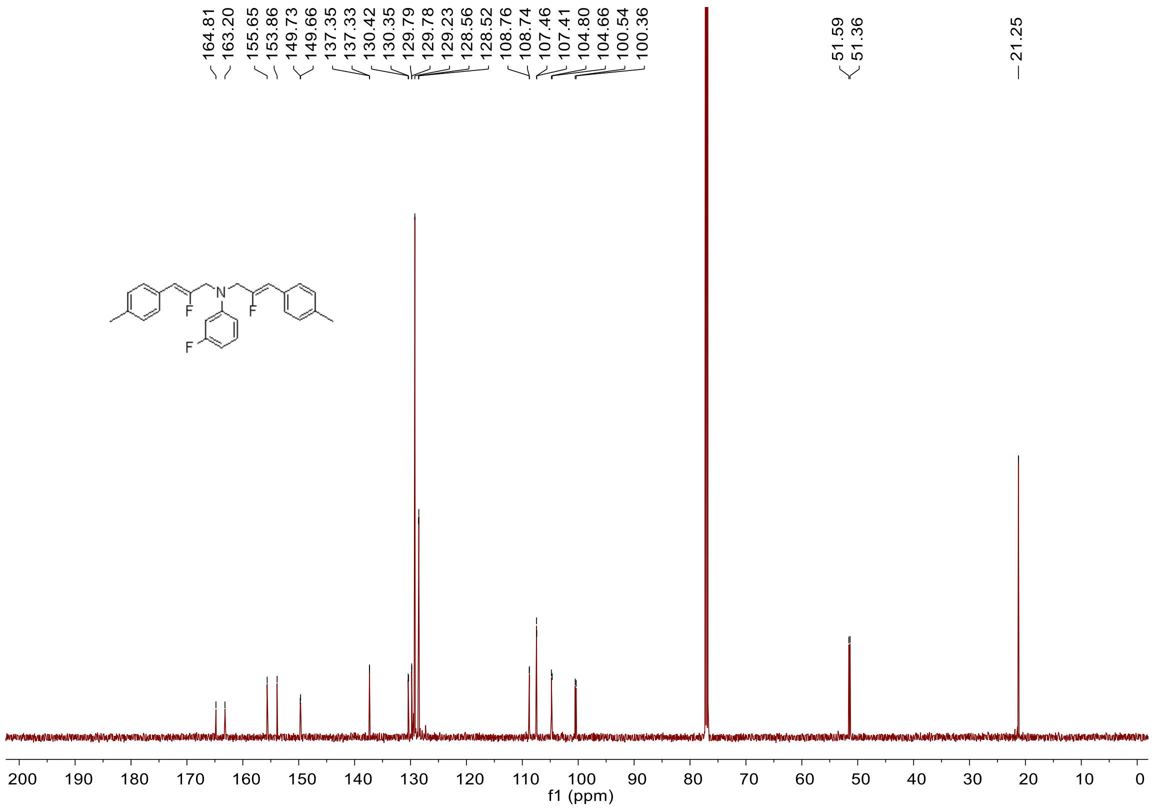

^{1}H NMR (**d17**)

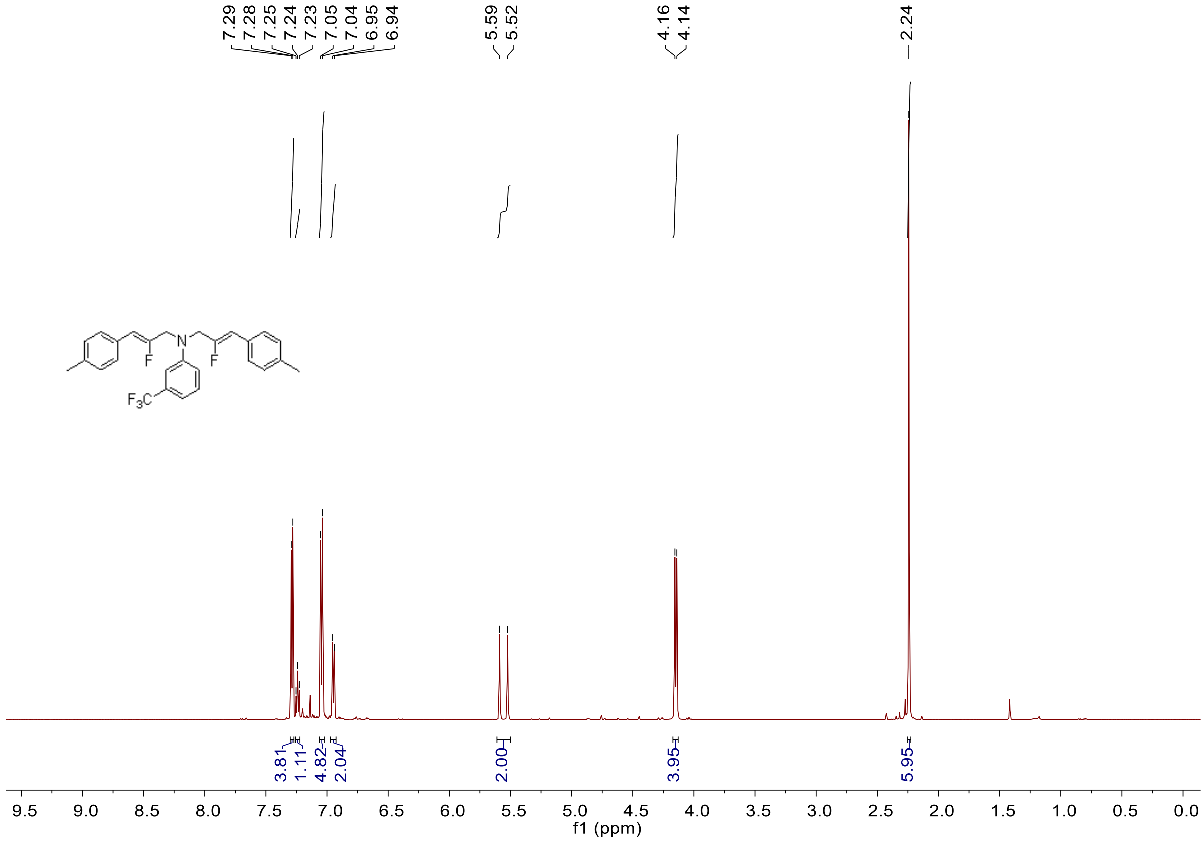

^{19}F NMR (**d17**)

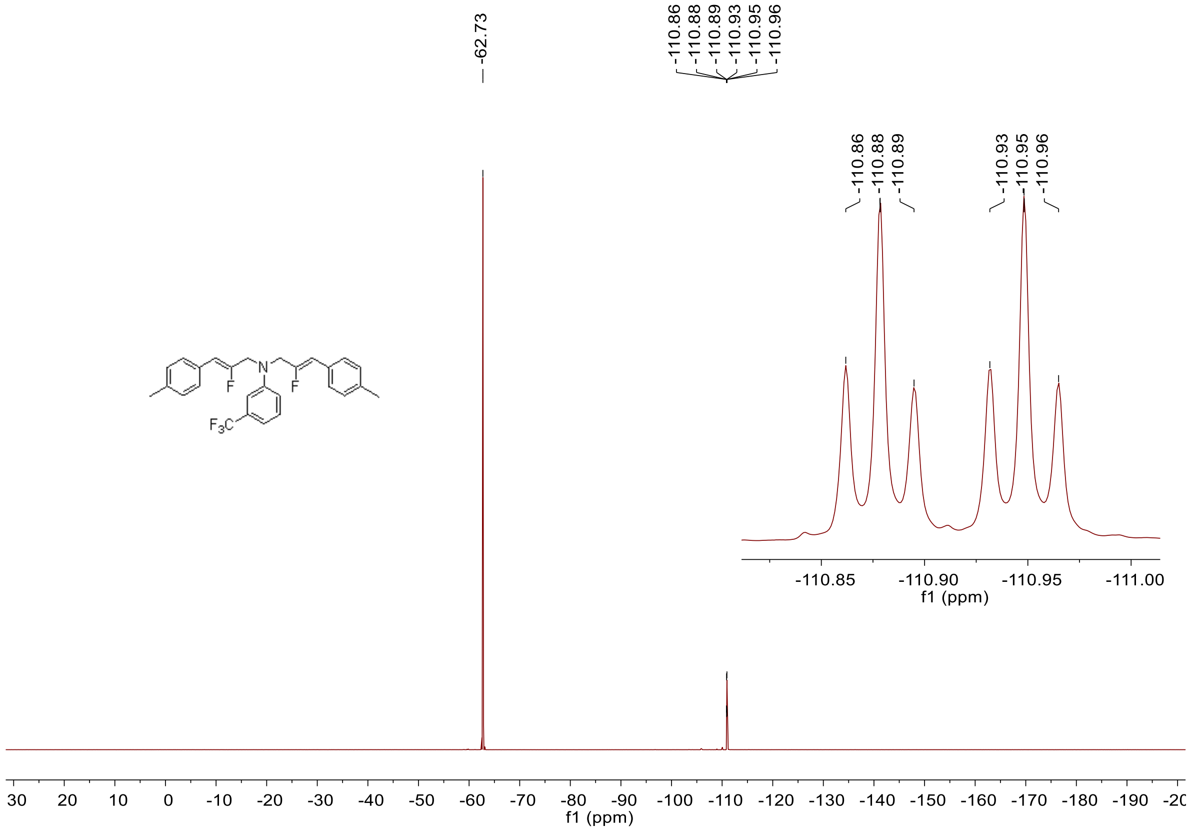

^{13}C NMR (**d17**)

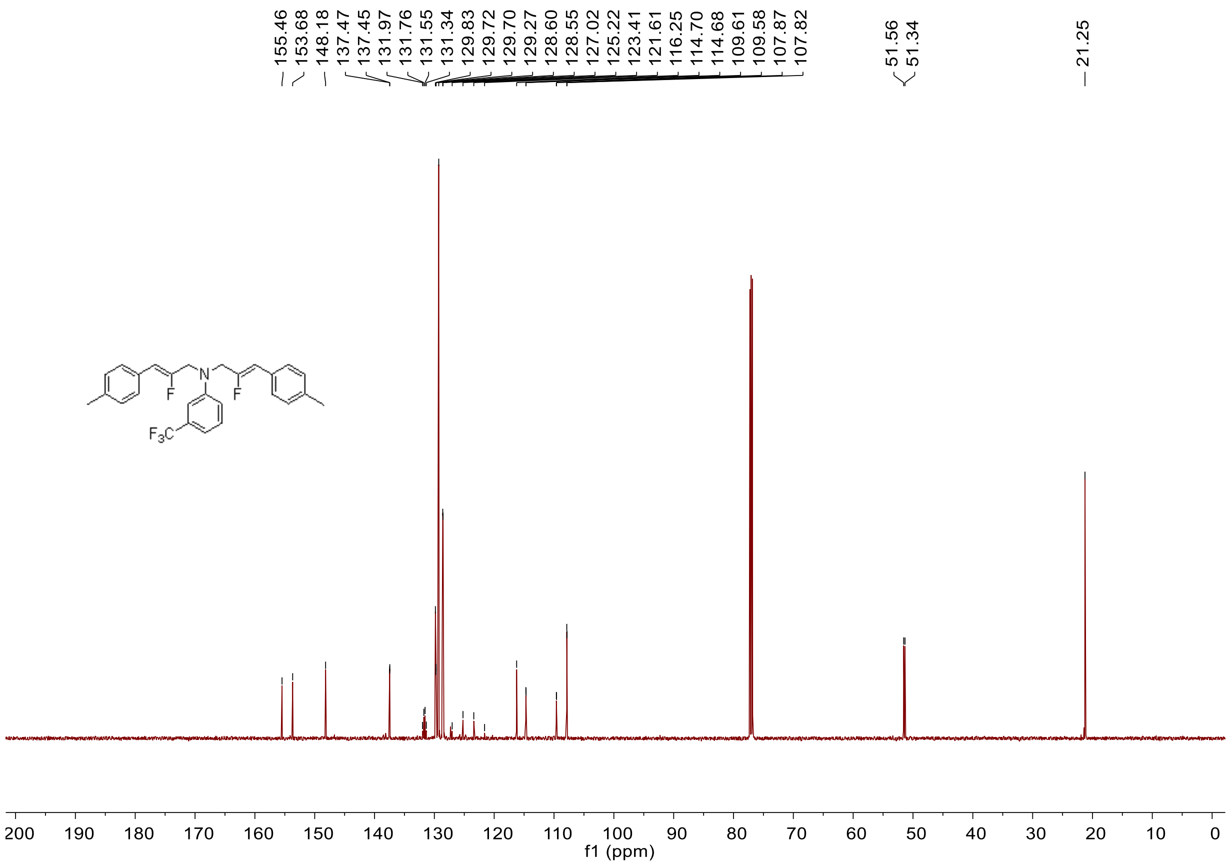

^{1}H NMR (**d18**)

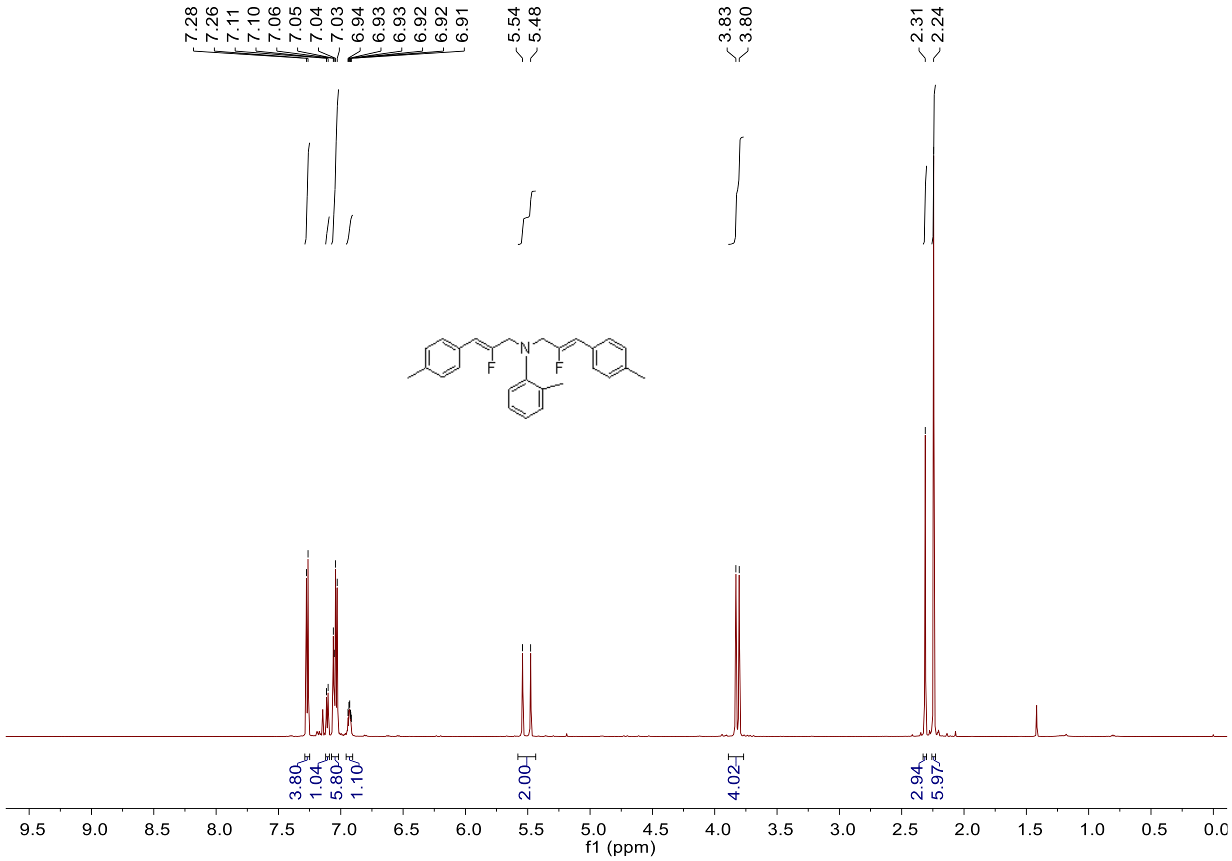

^{19}F NMR (**d18**)

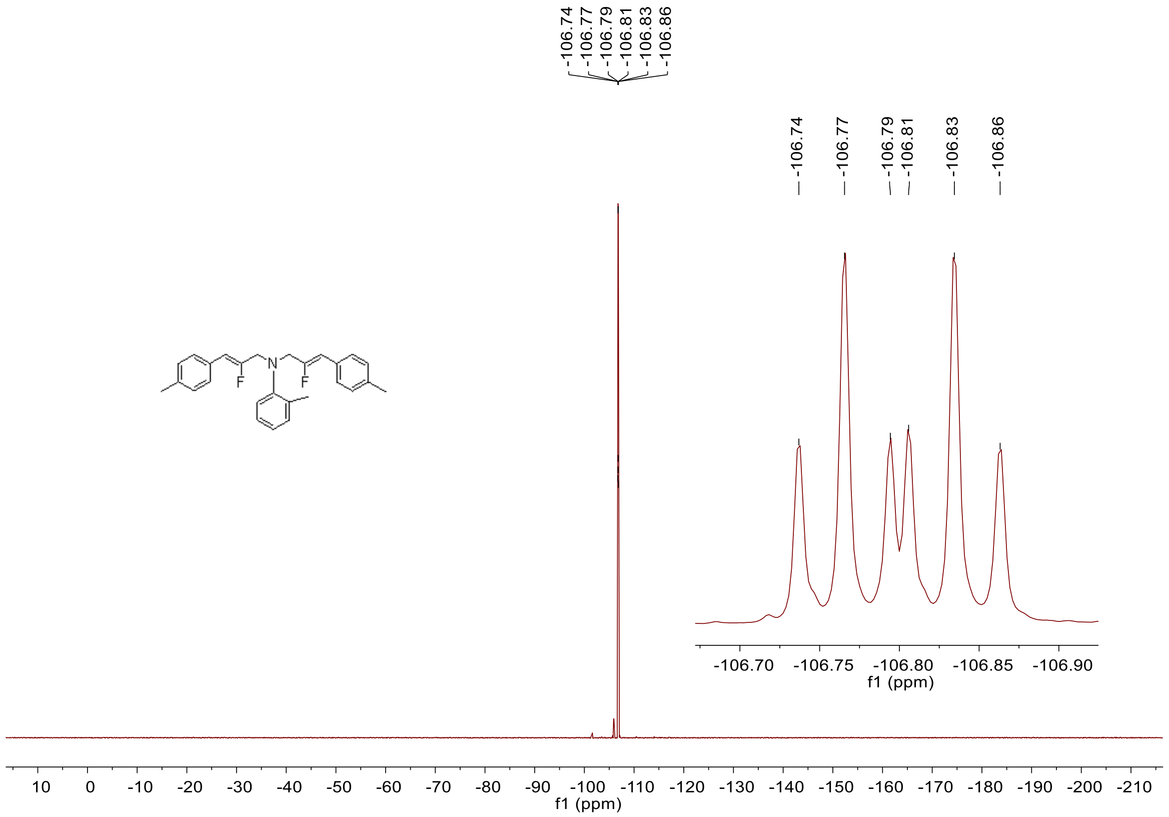

^{13}C NMR (**d18**)

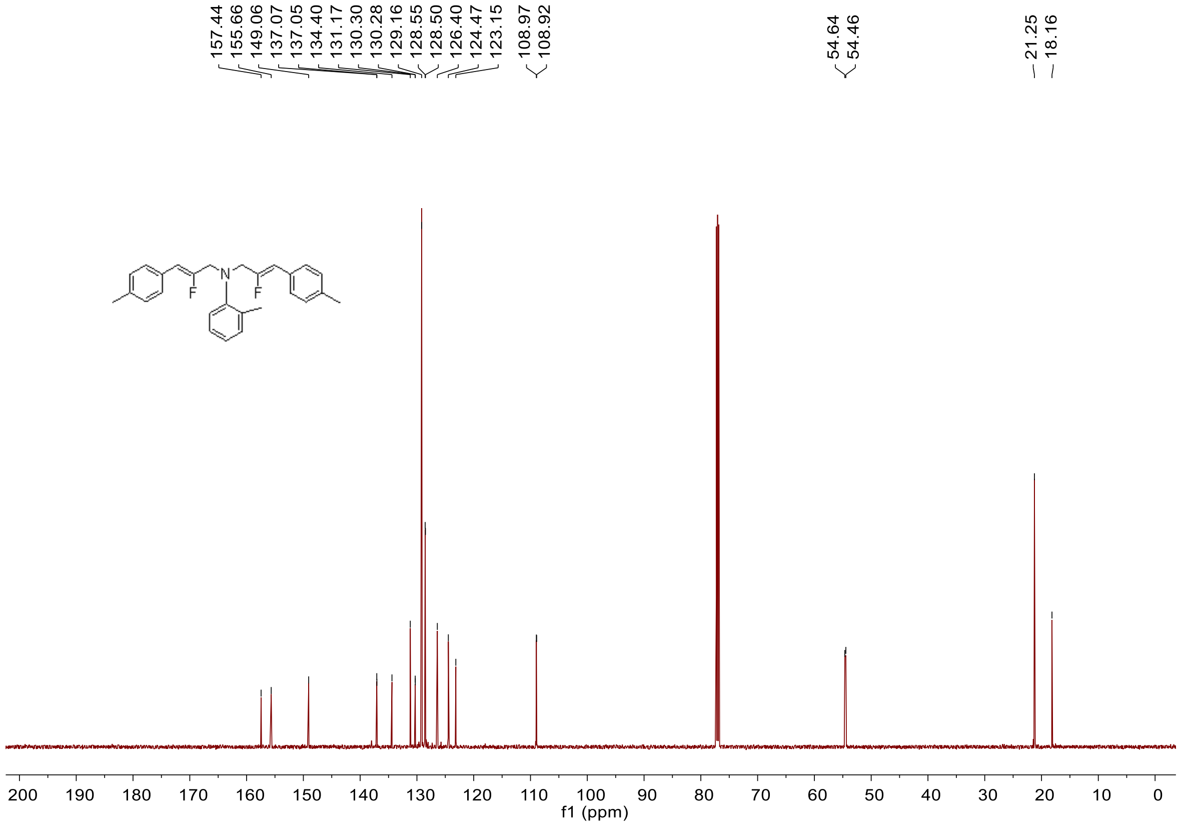

^{1}H NMR (**d19**)

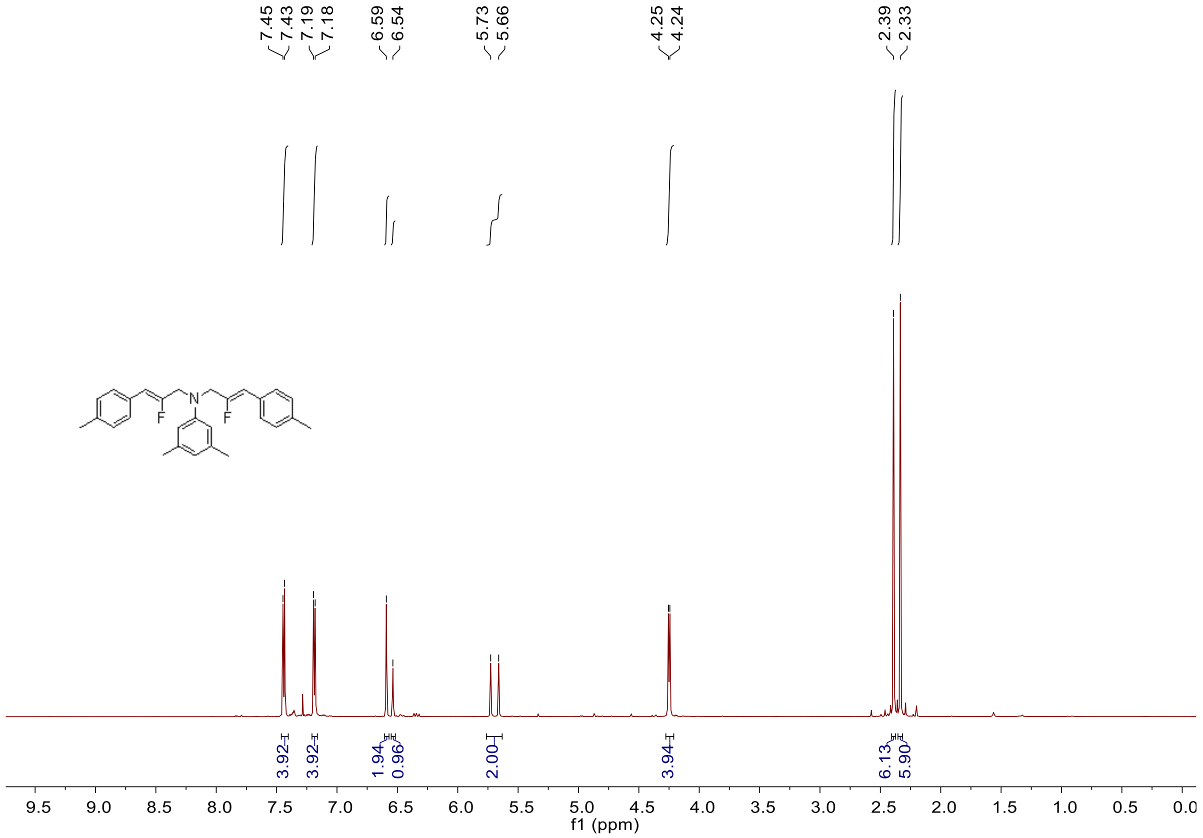

^{19}F NMR (**d19**)

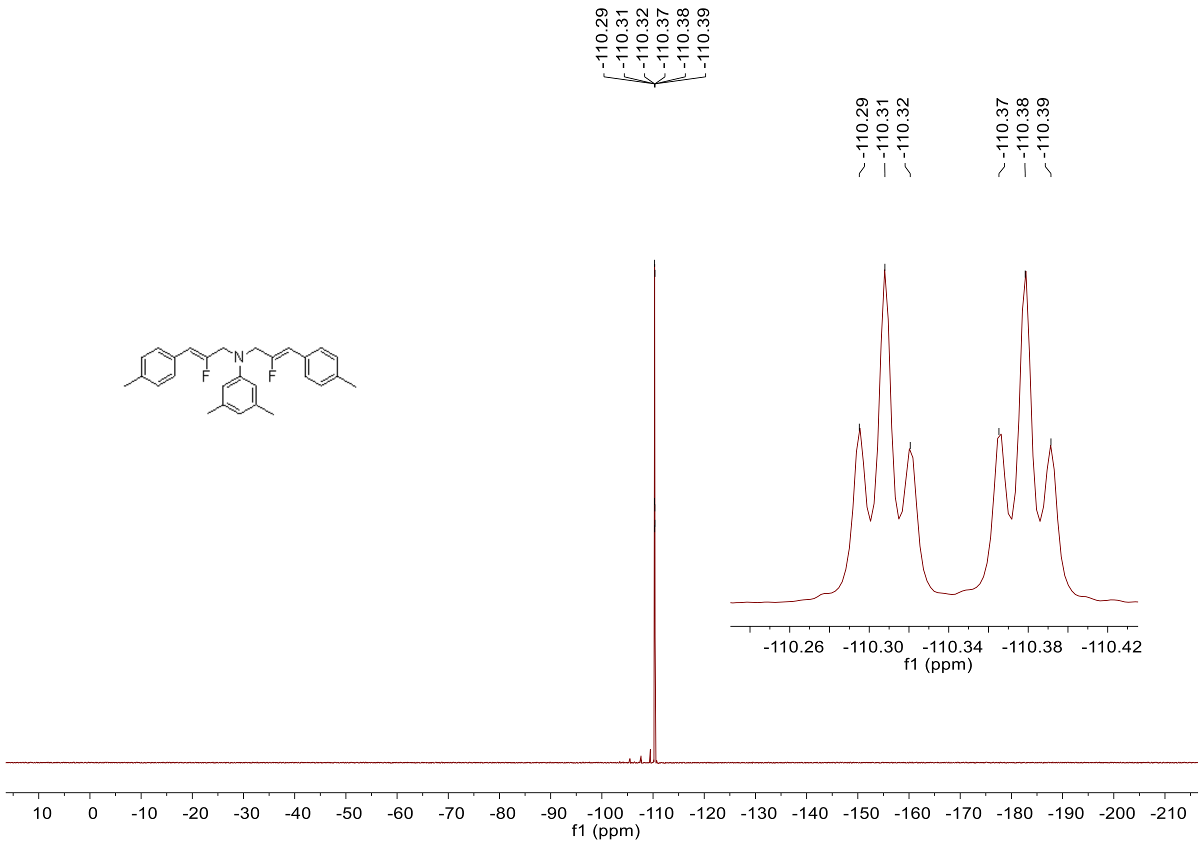

^{13}C NMR (**d19**)

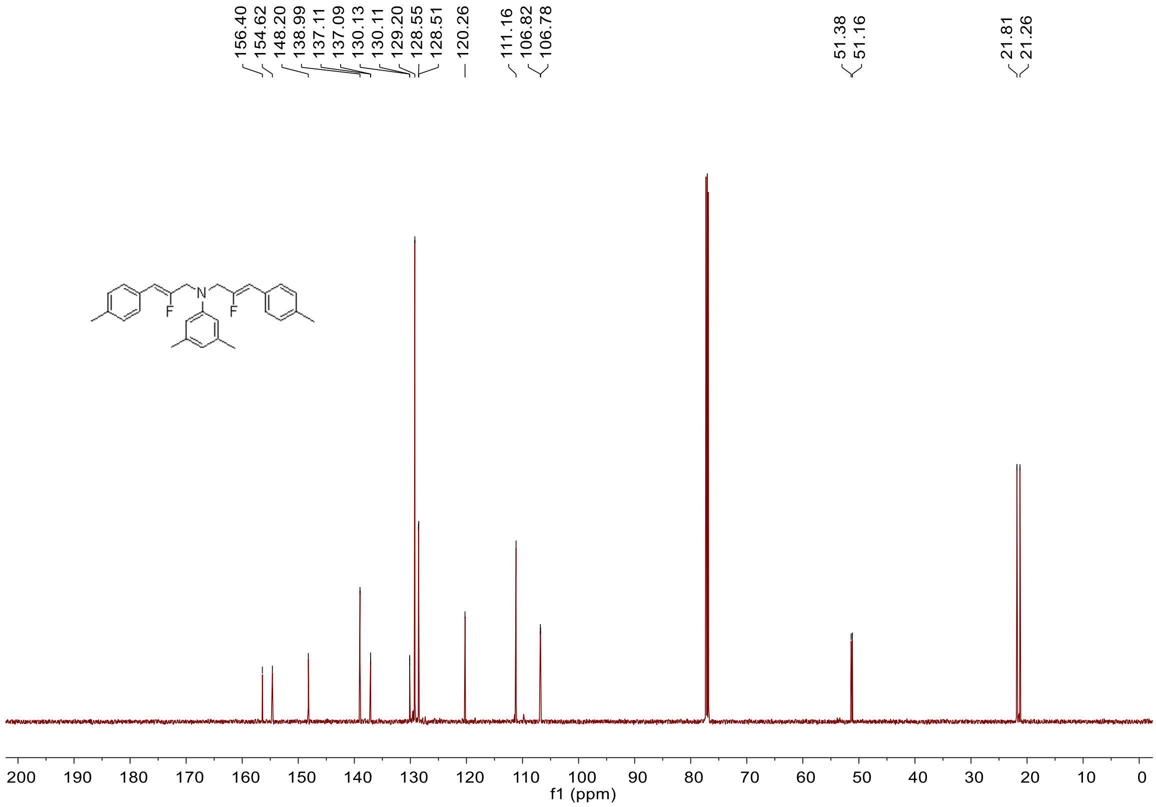

^{1}H NMR (**d20**)

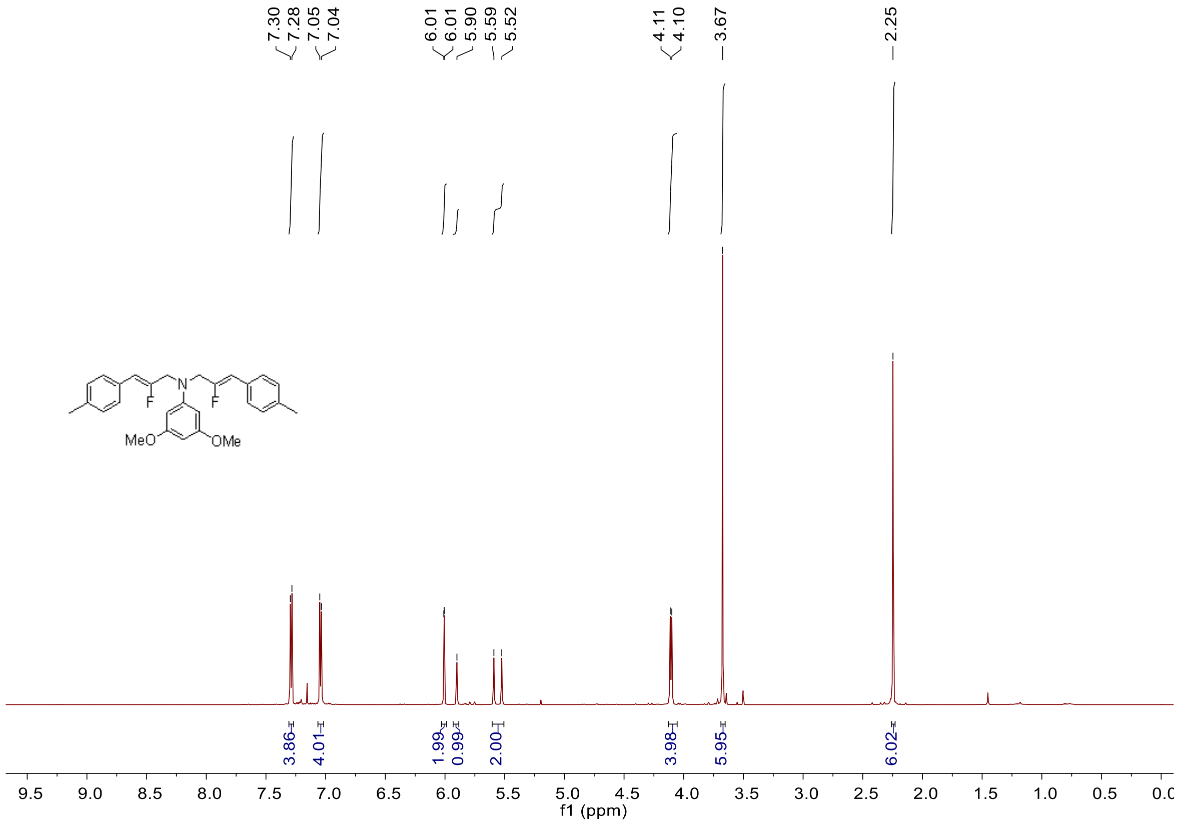

^{19}F NMR (**d20**)

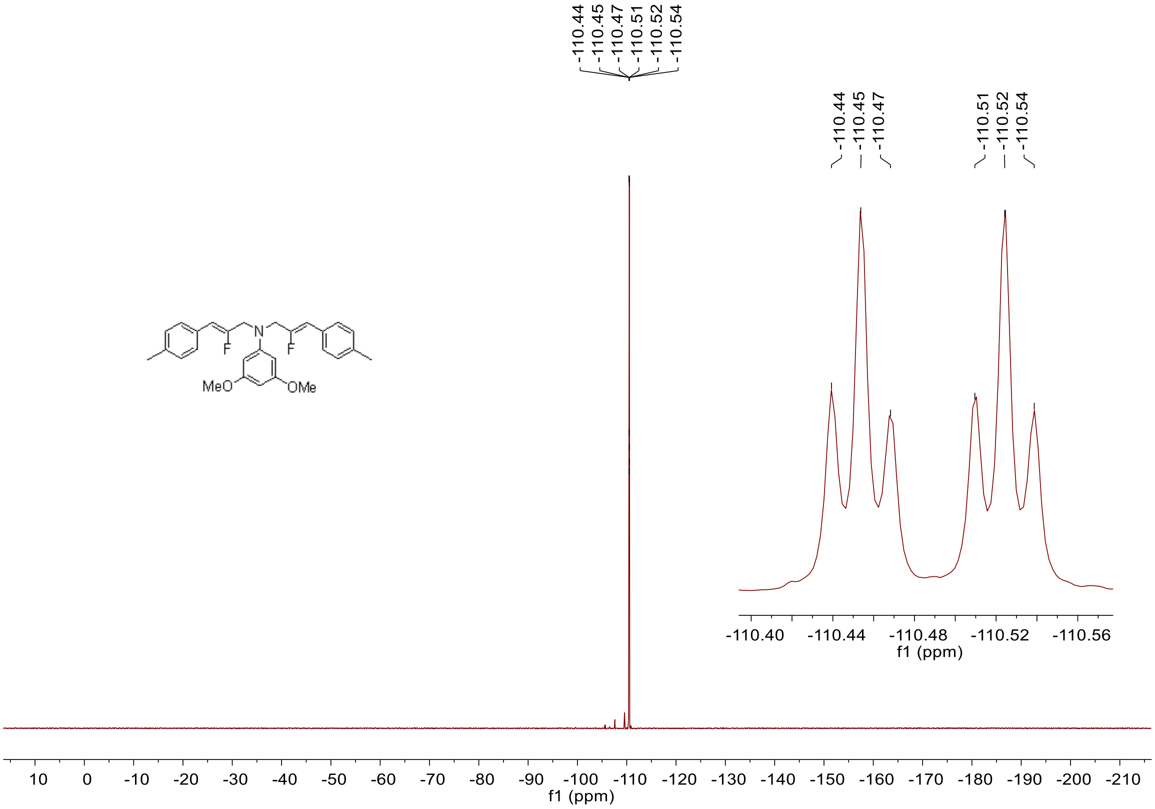

^{13}C NMR (**d20**)

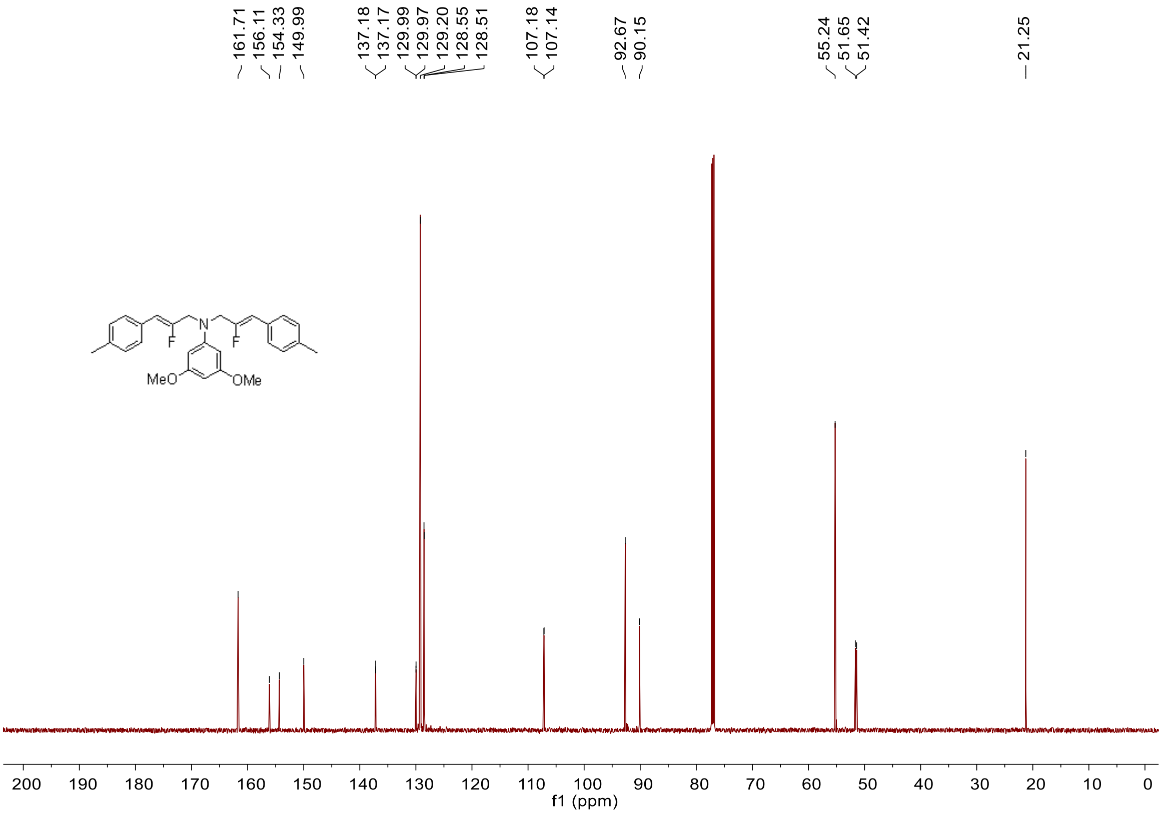

^{1}H NMR (**d21**)

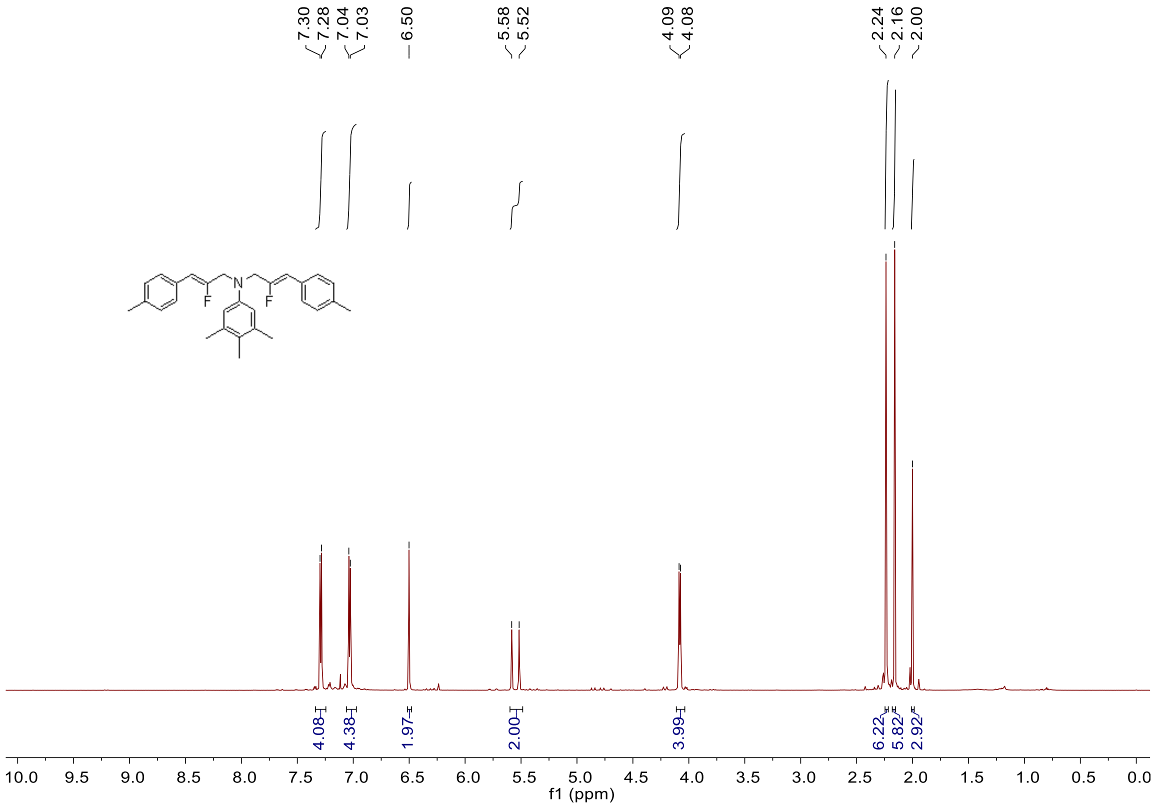

^{19}F NMR (**d21**)

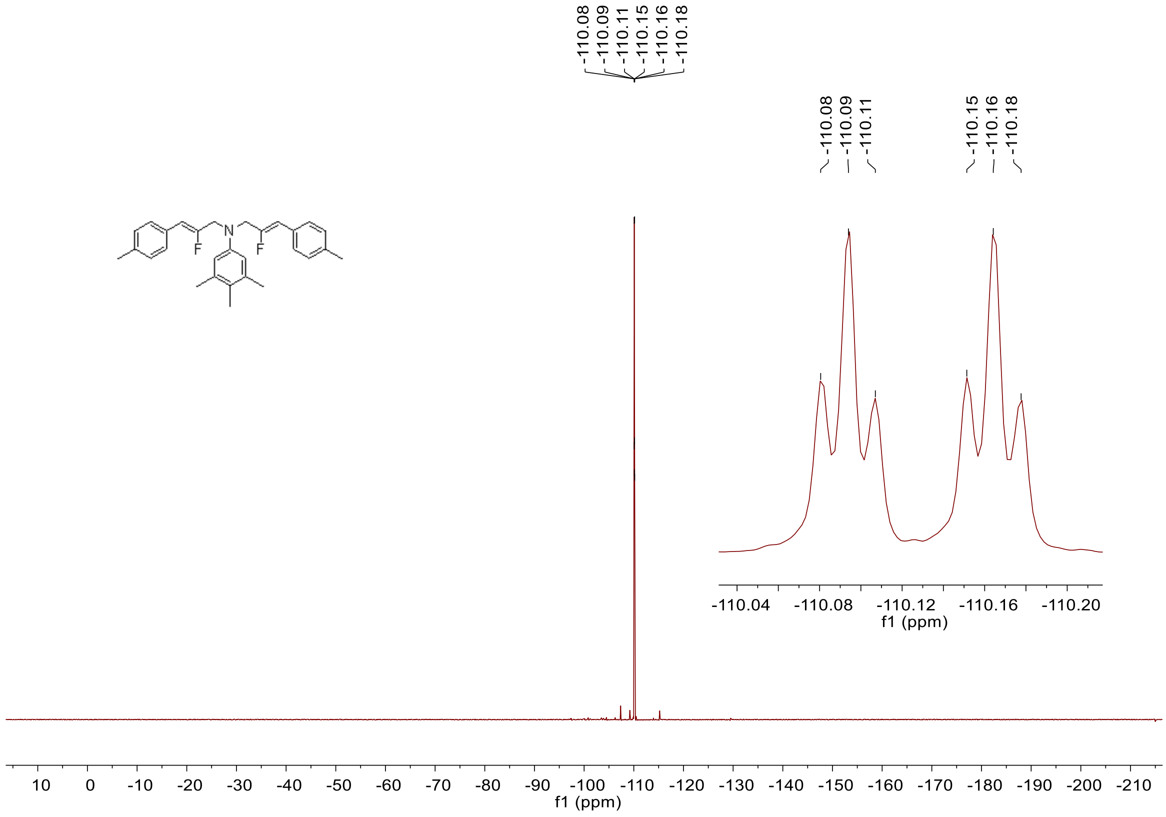

^{13}C NMR (**d21**)

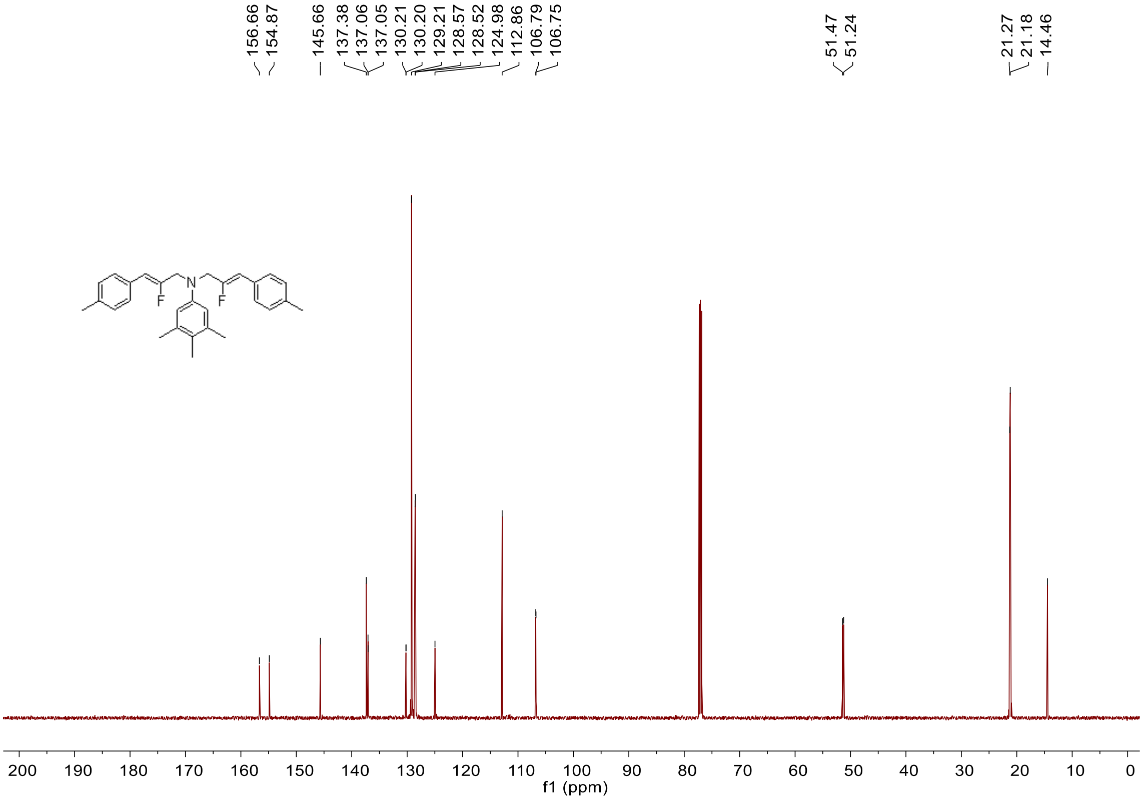

^{1}H NMR (**d22**)

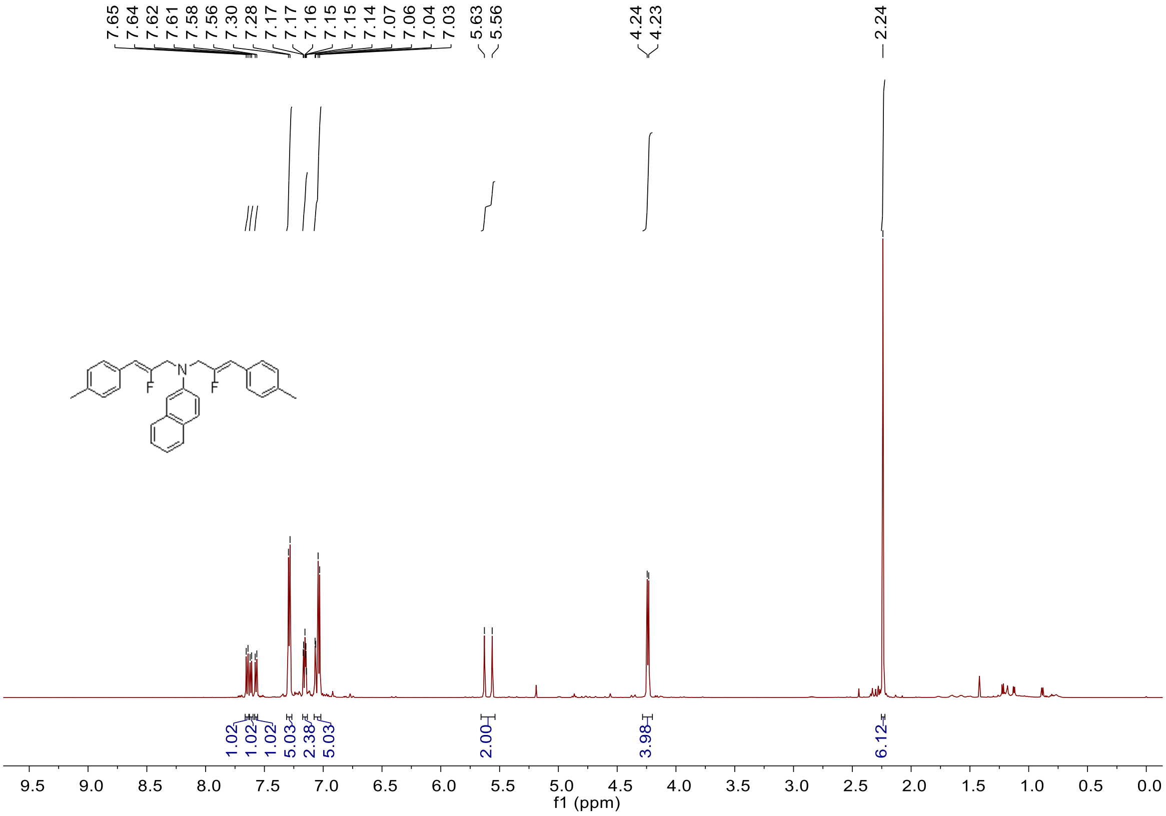

^{19}F NMR (**d22**)

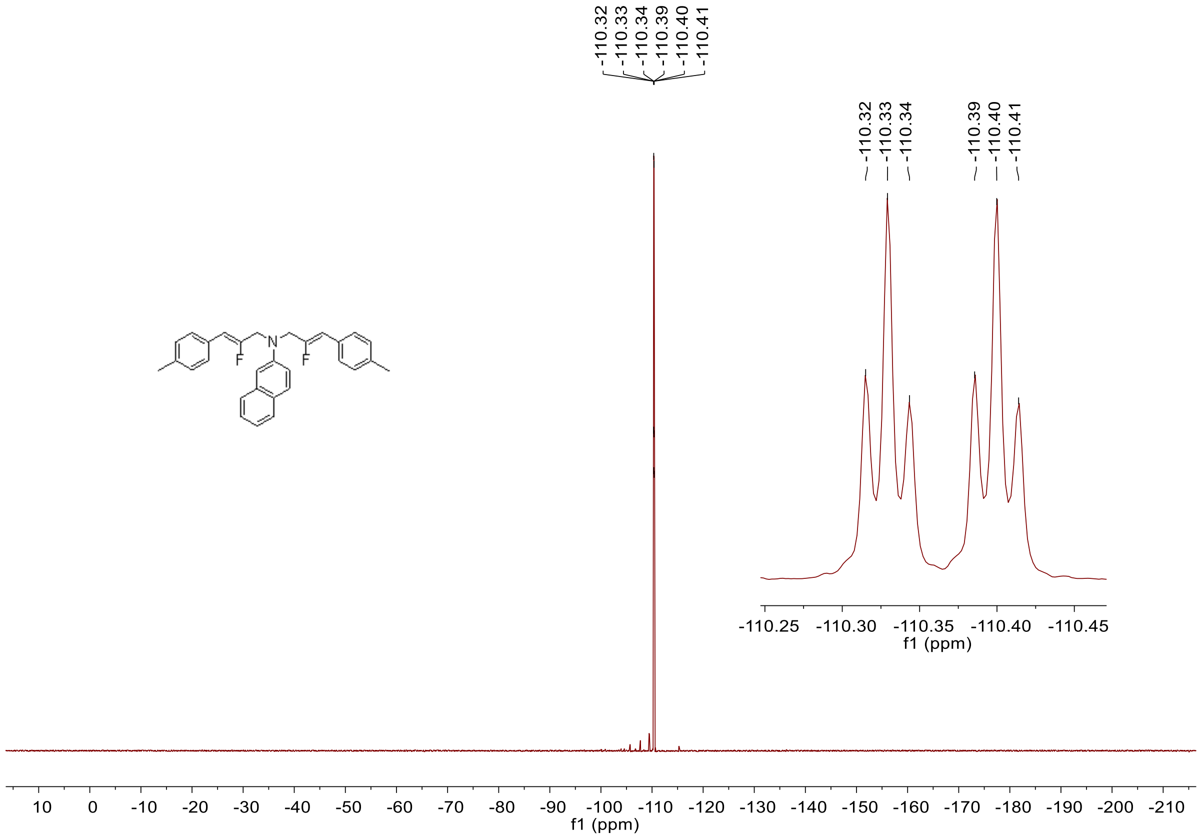

^{13}C NMR (**d22**)

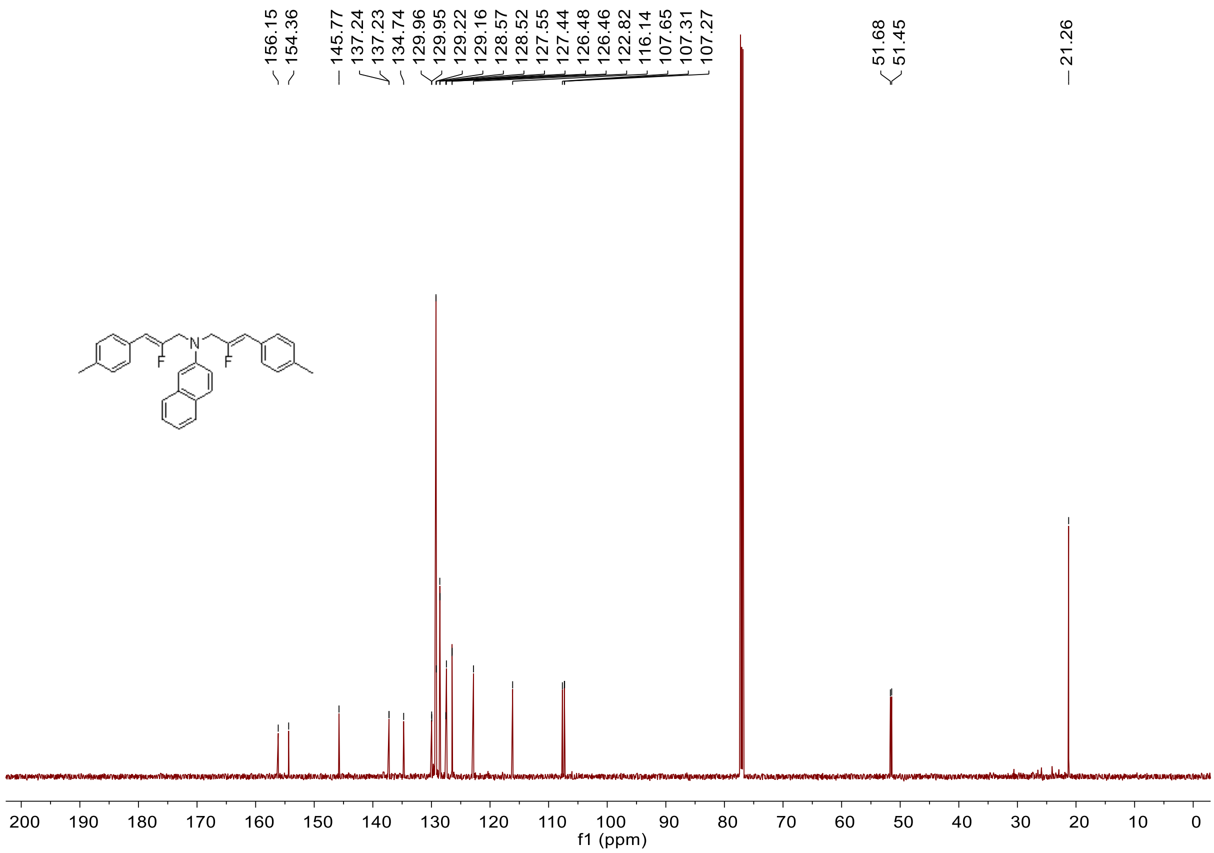

^{1}H NMR (**d23**)

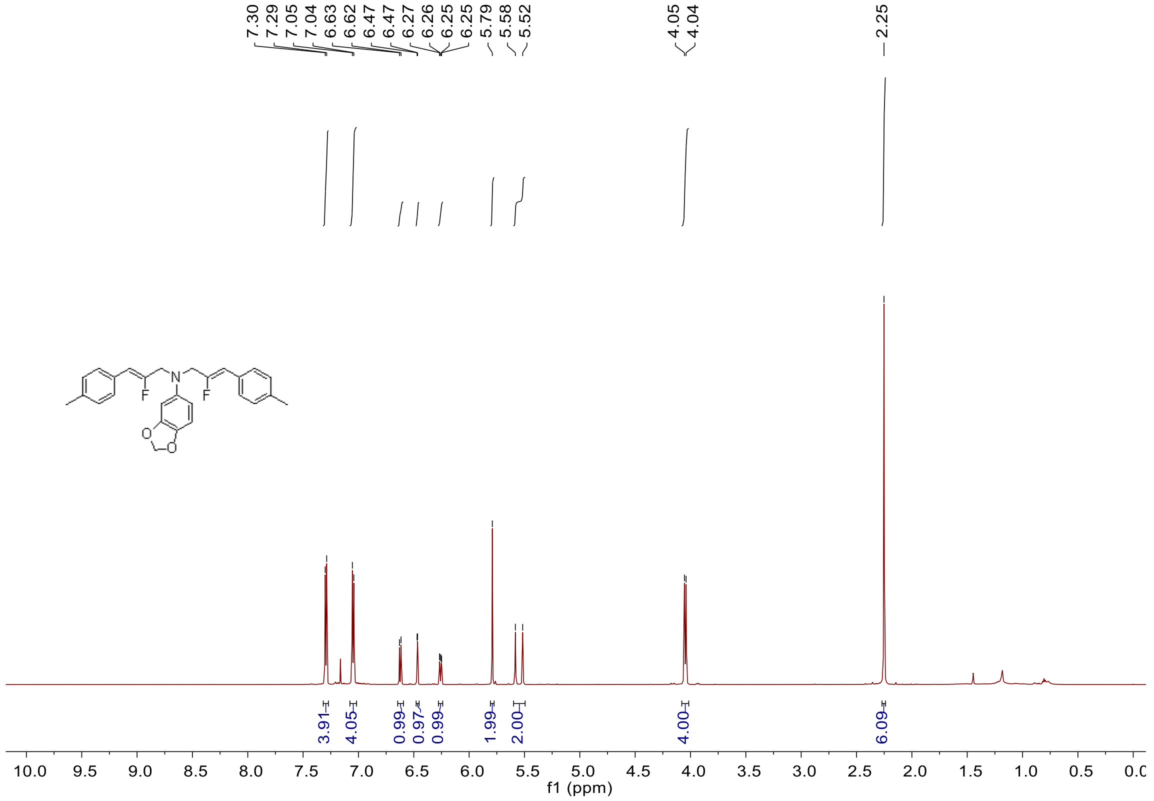

^{19}F NMR (**d23**)

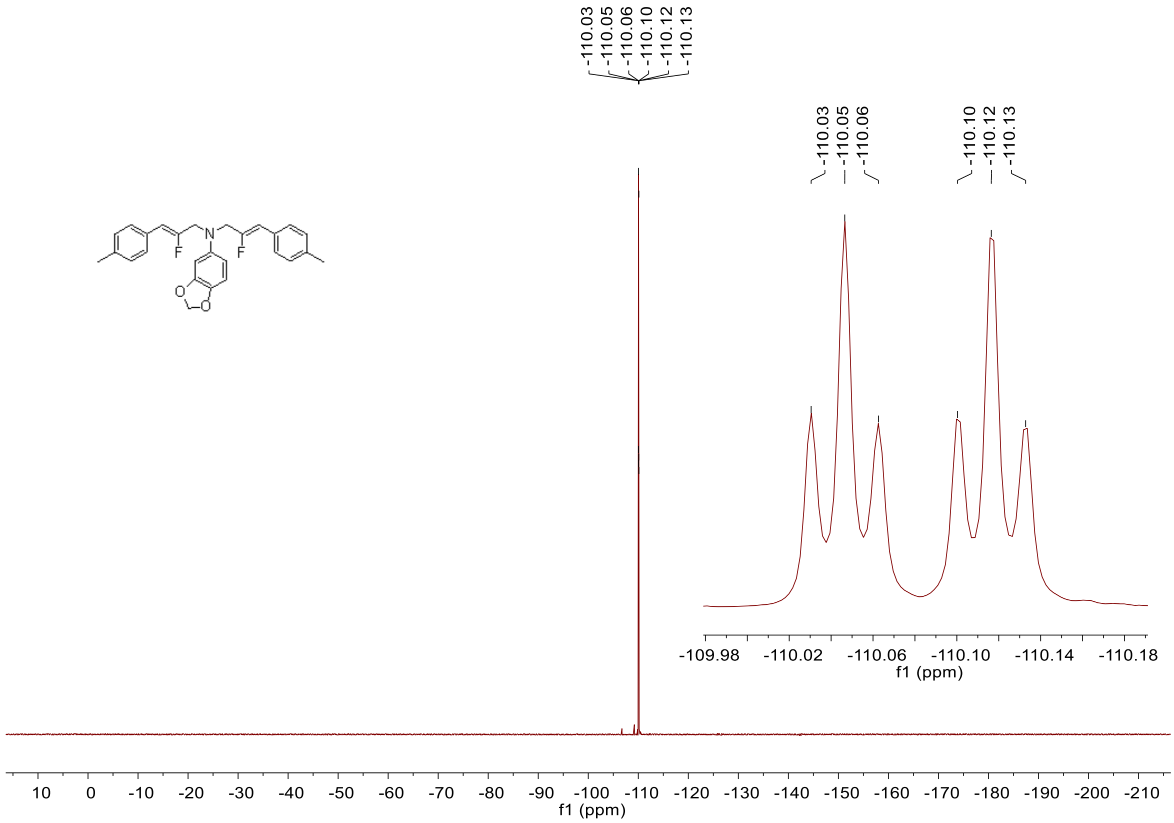

^{13}C NMR (**d23**)

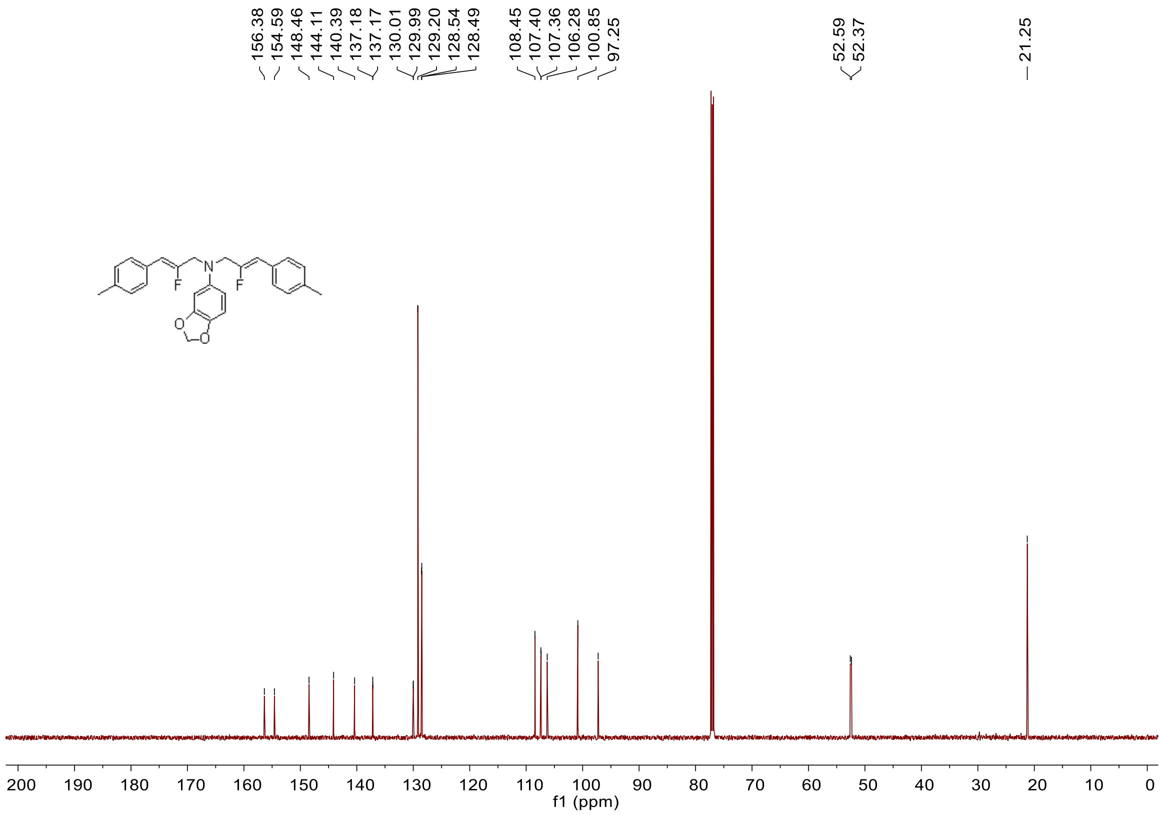

^{1}H NMR (**d24**)

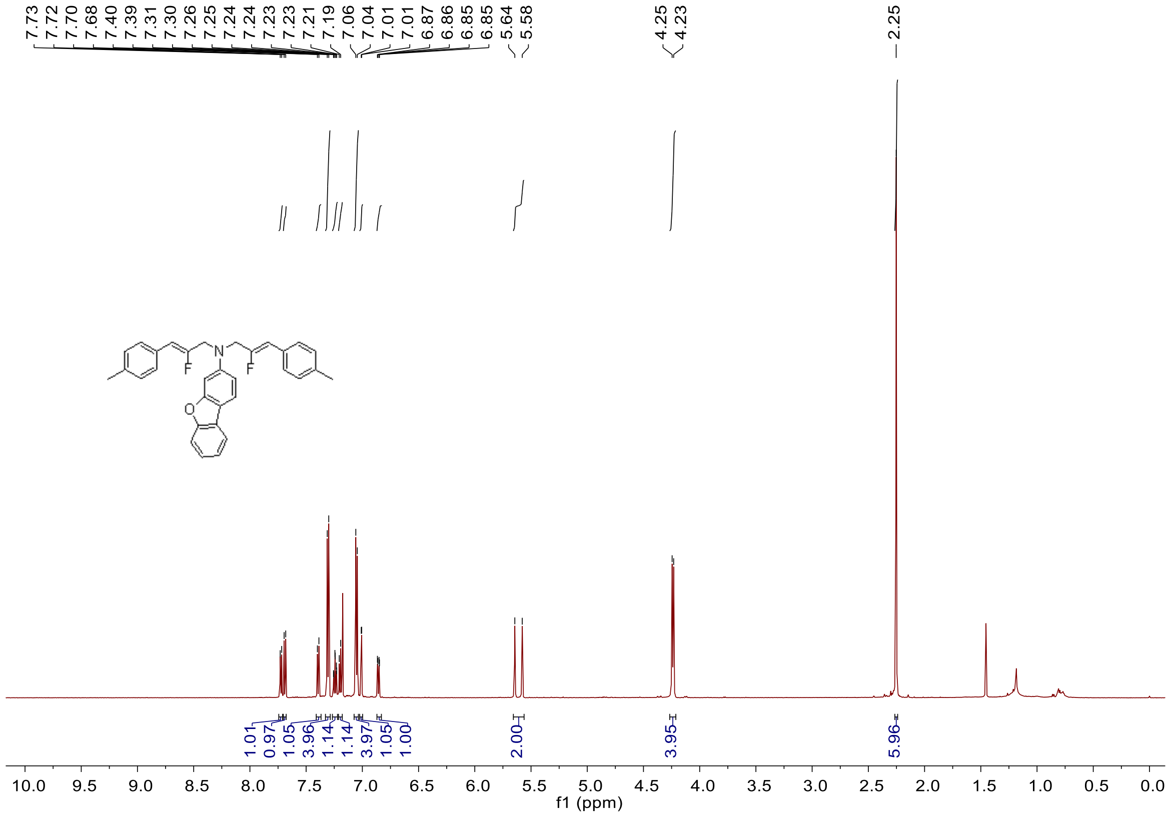

^{19}F NMR (**d24**)

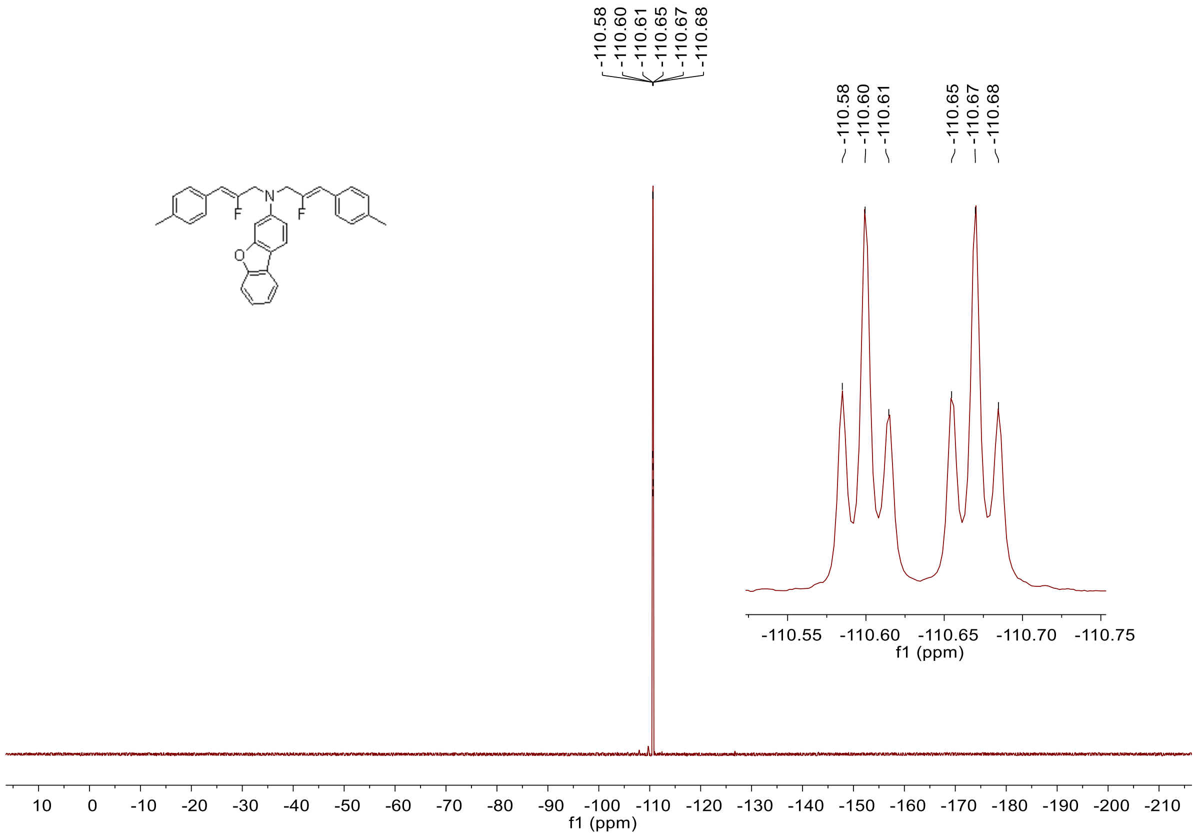

^{13}C NMR (**d24**)

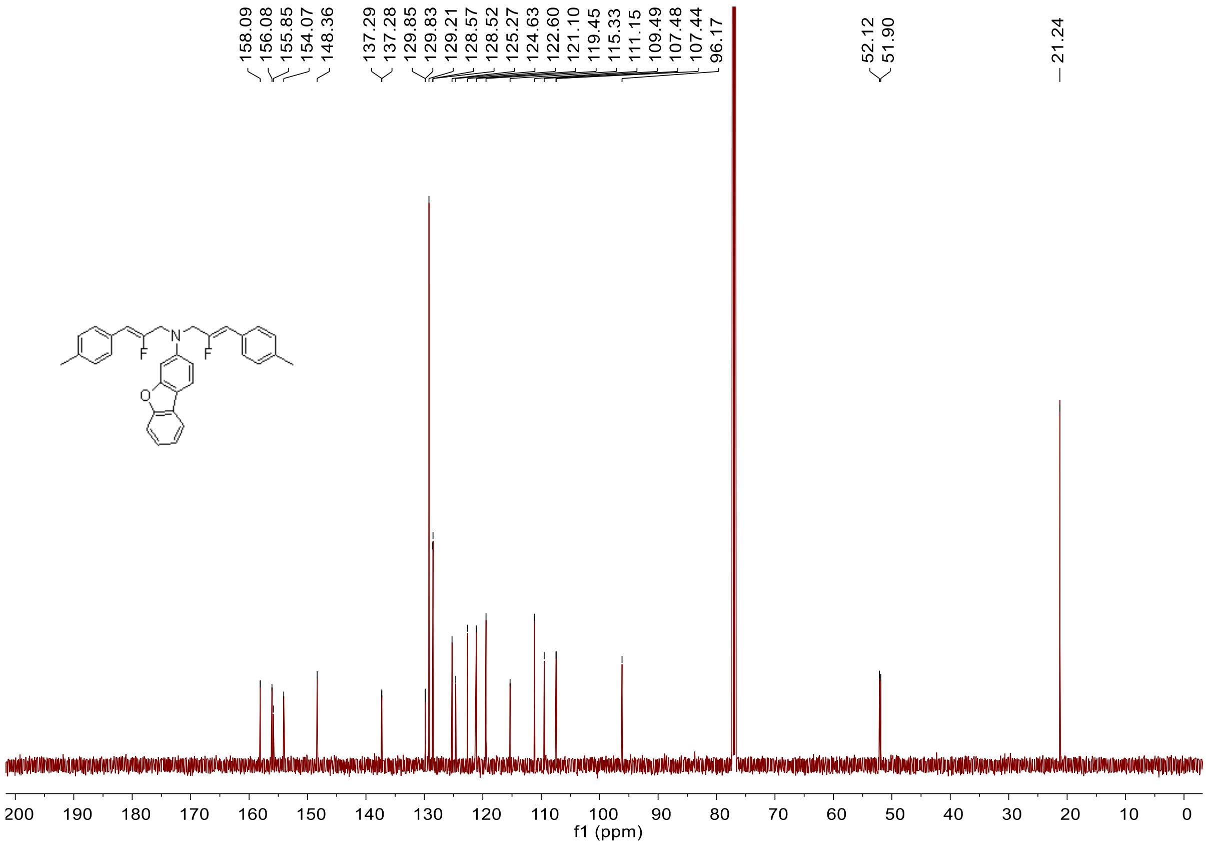

^{1}H NMR (**d25**)

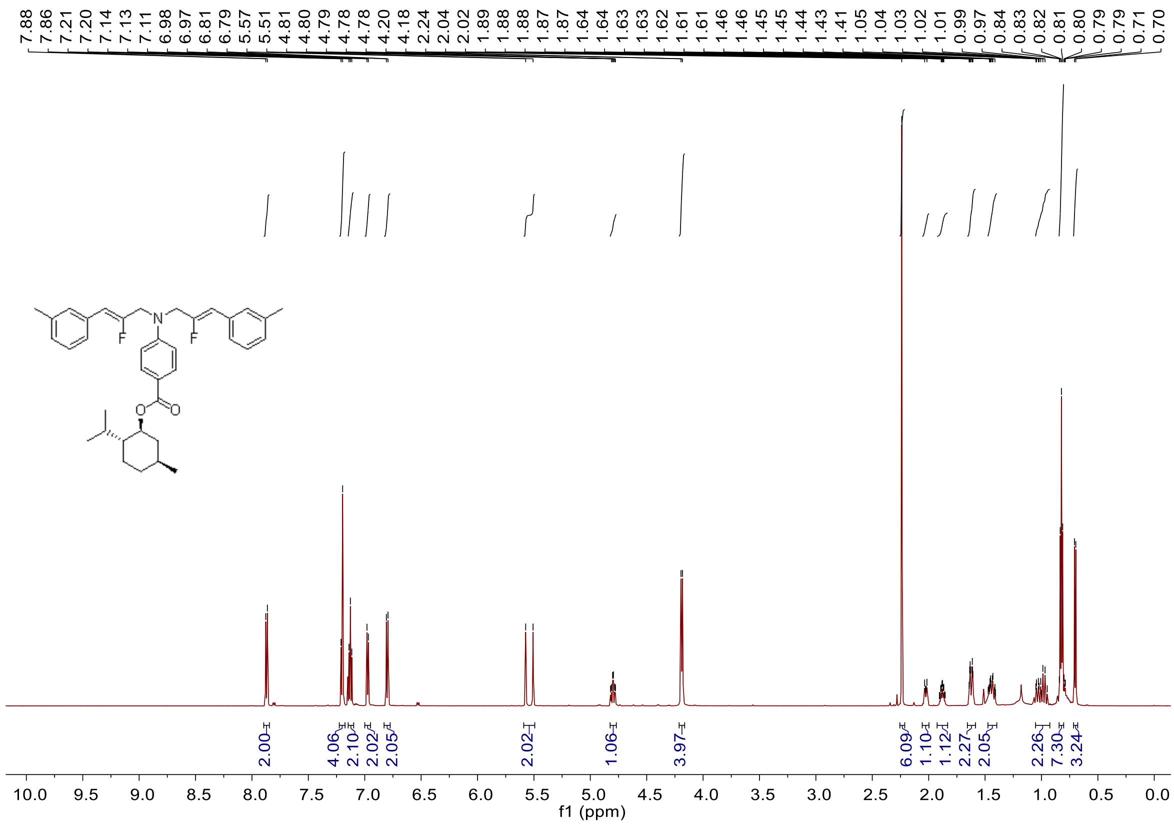

^{19}F NMR (**d25**)

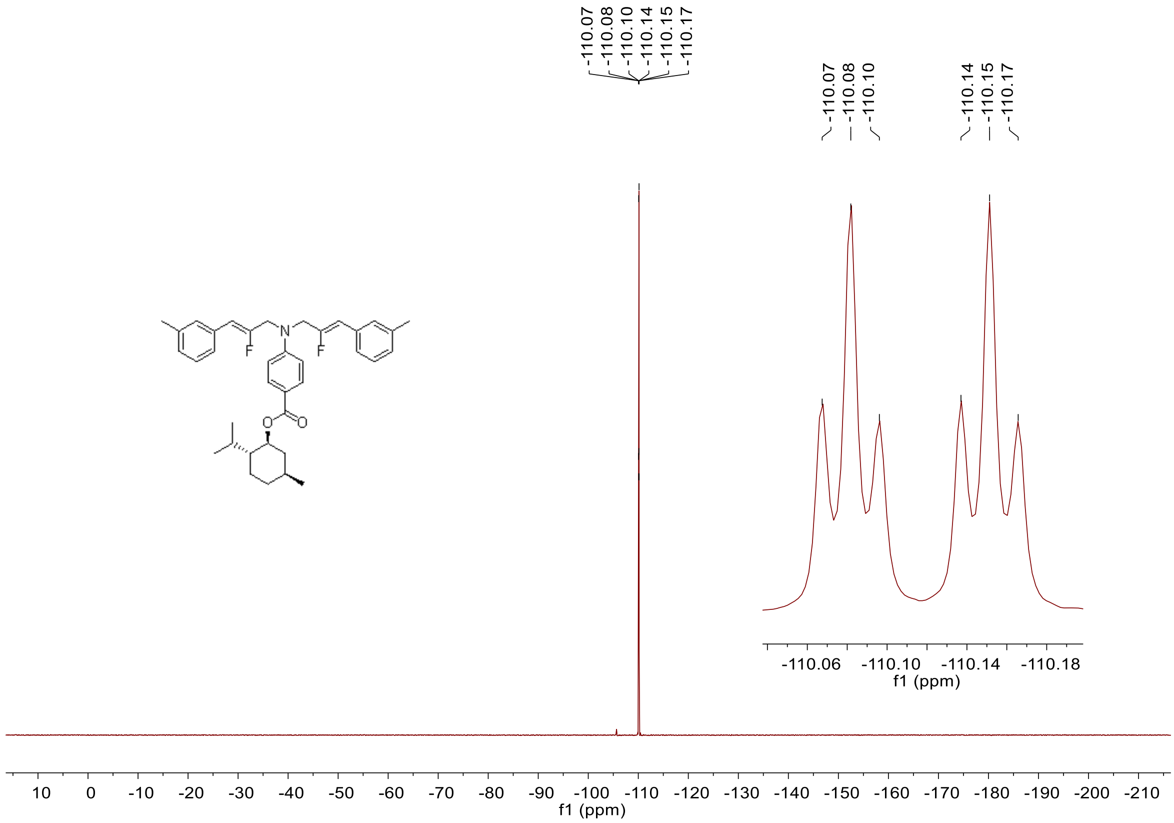

^{13}C NMR (**d25**)

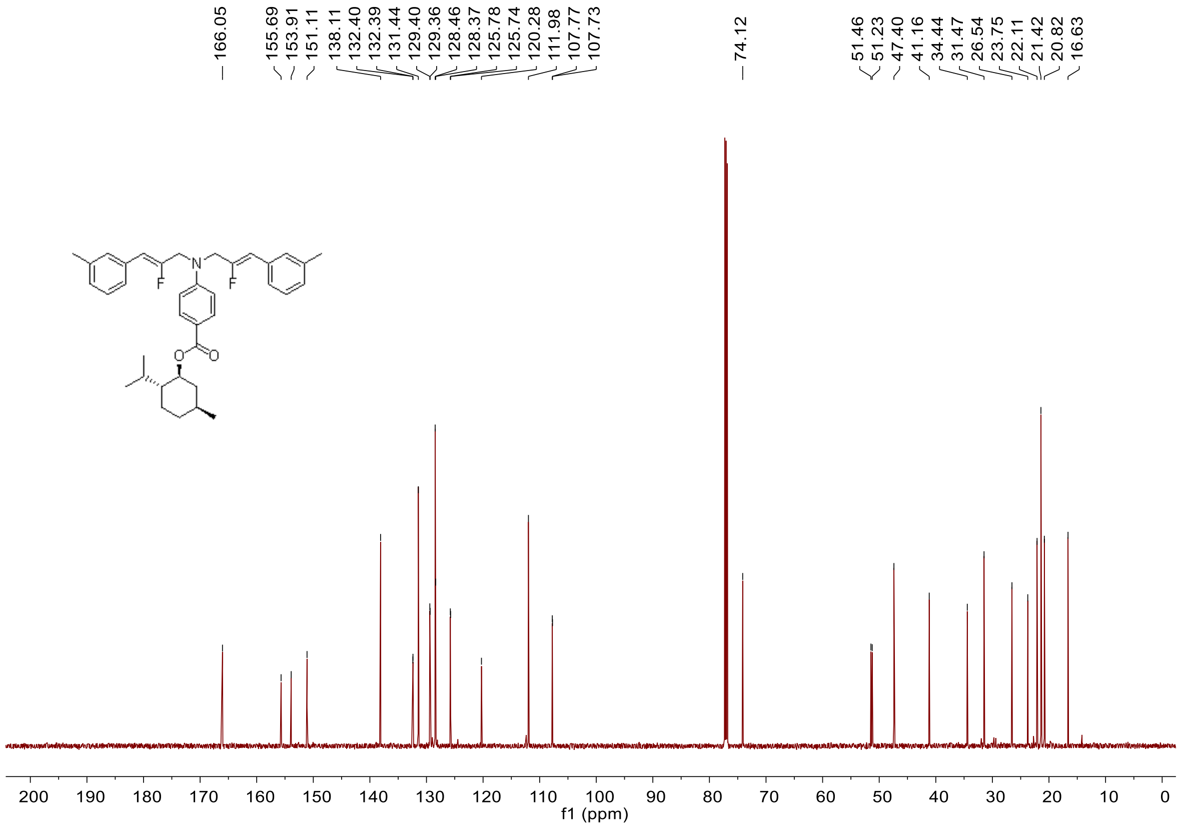

^{1}H NMR (**d26**)

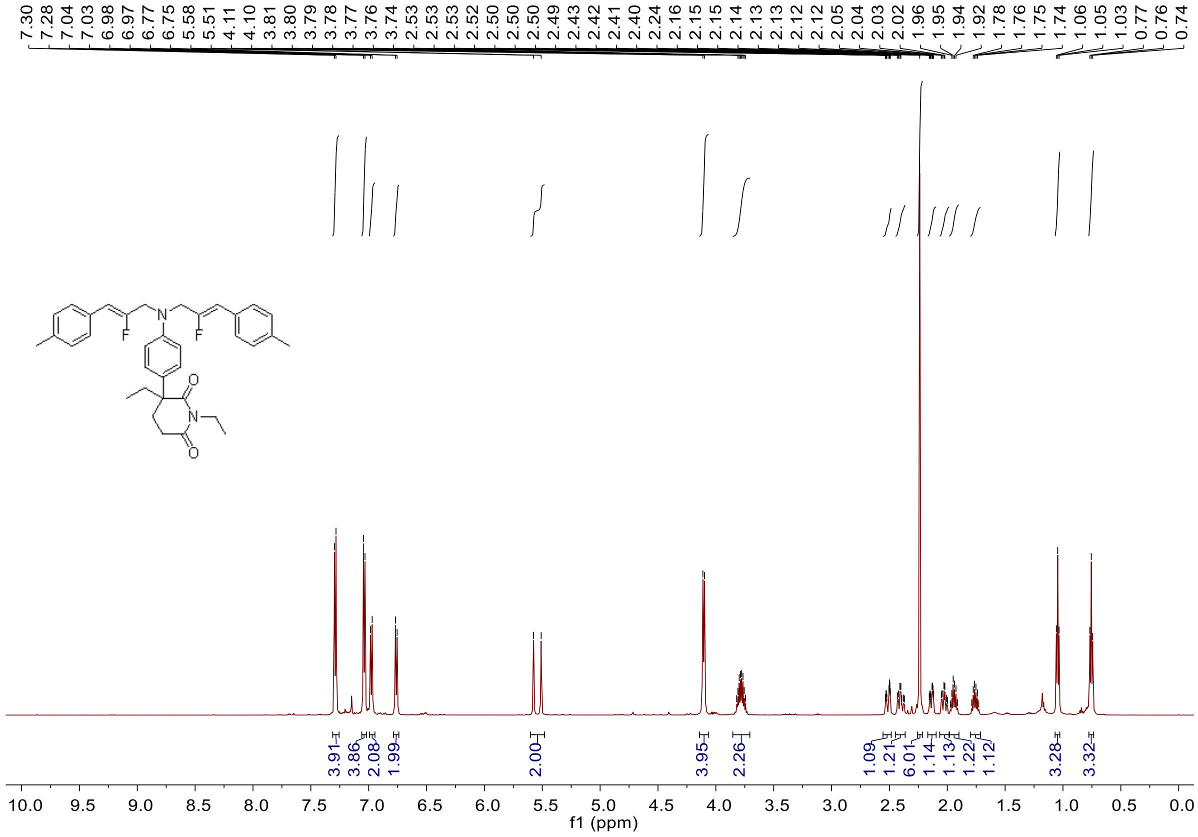

^{19}F NMR (**d26**)

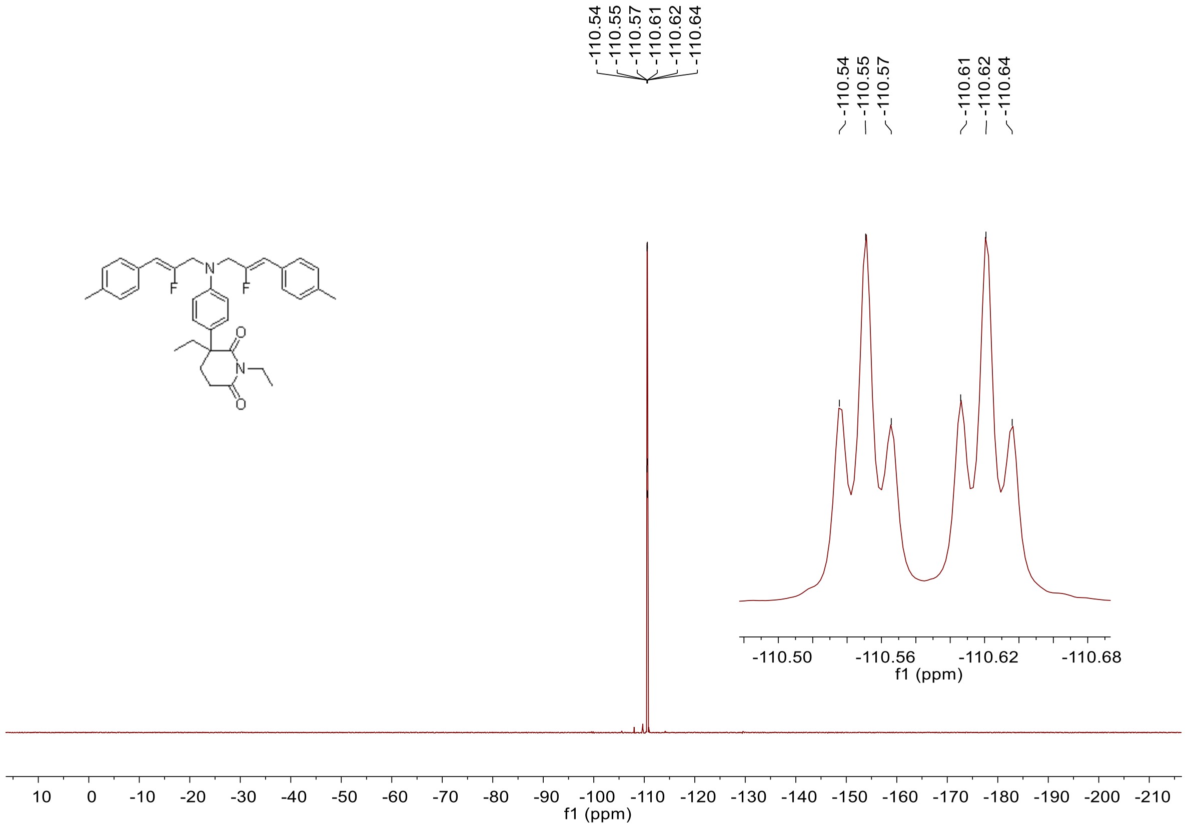

^{13}C NMR (**d26**)

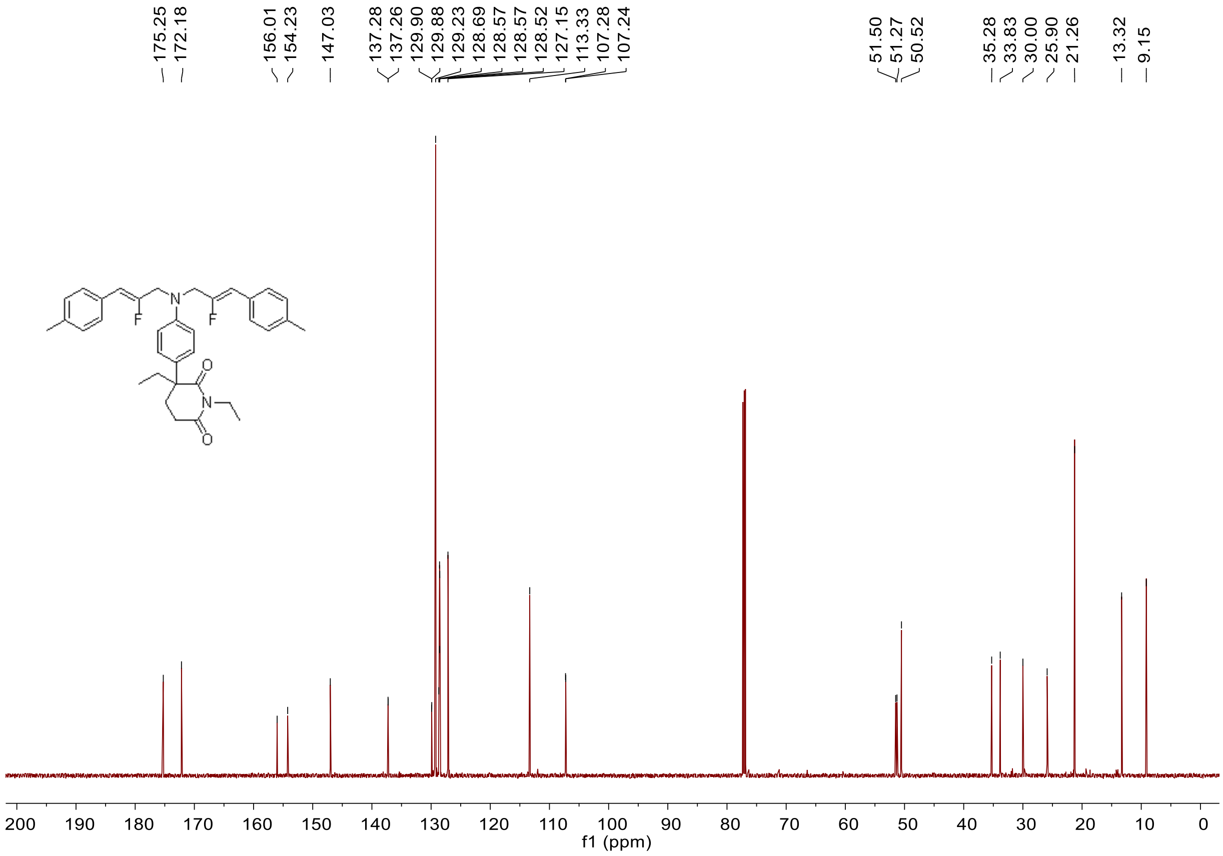

^{1}H NMR (**d27**)

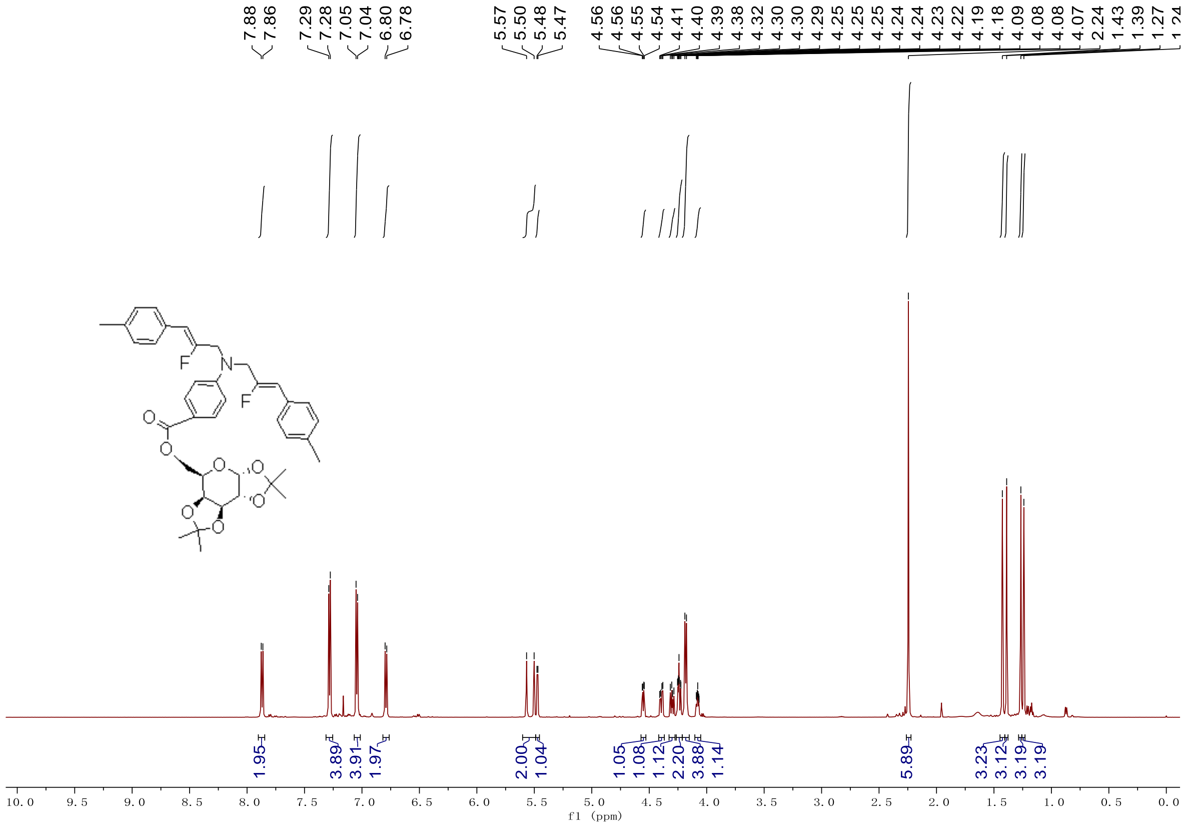

^{19}F NMR (**d27**)

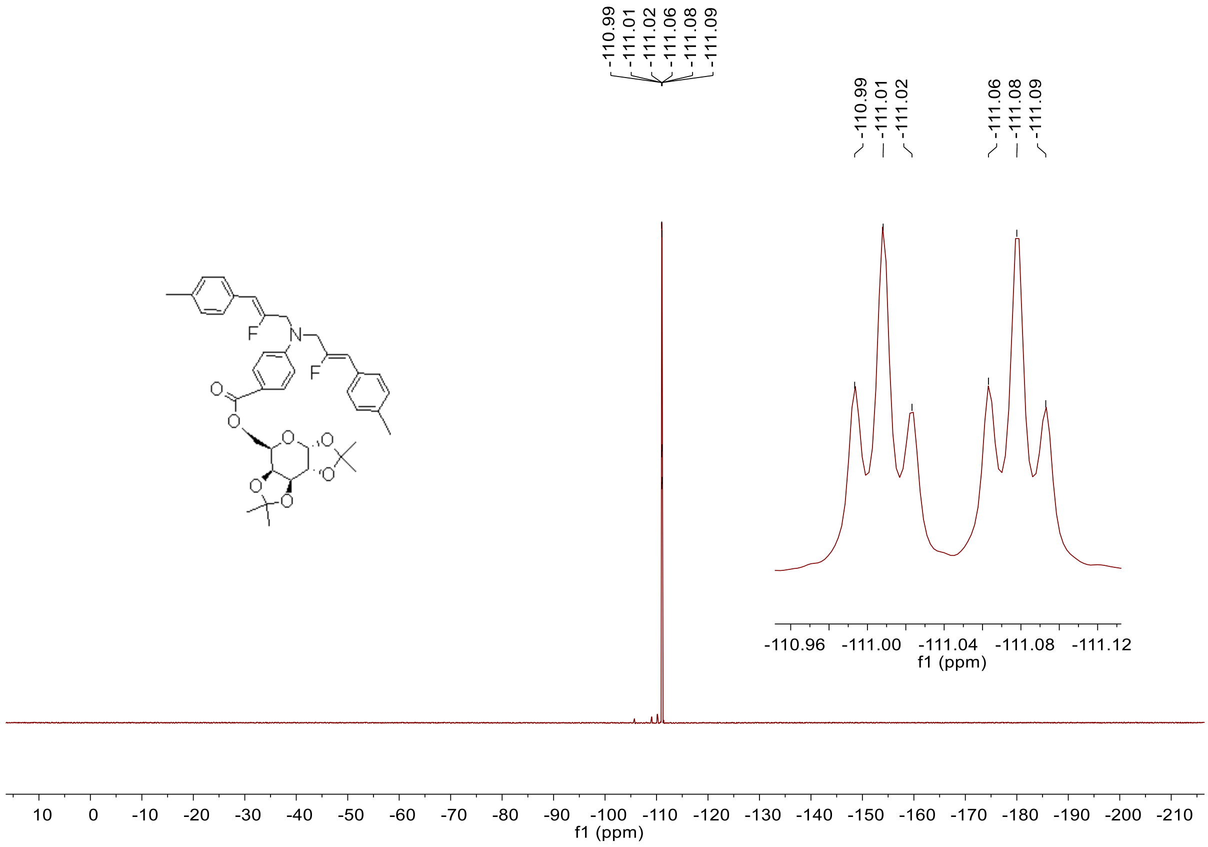

^{13}C NMR (**d27**)

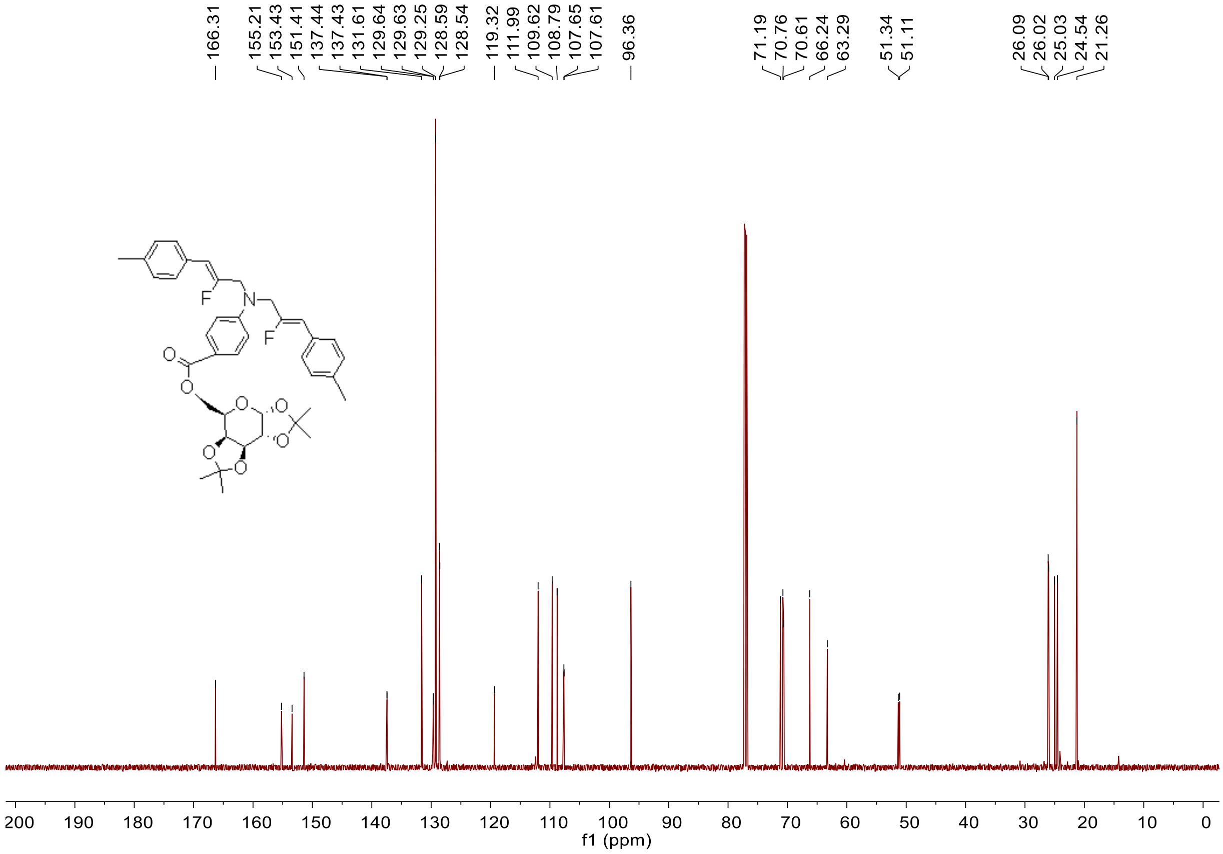

^{1}H NMR (**d28**)

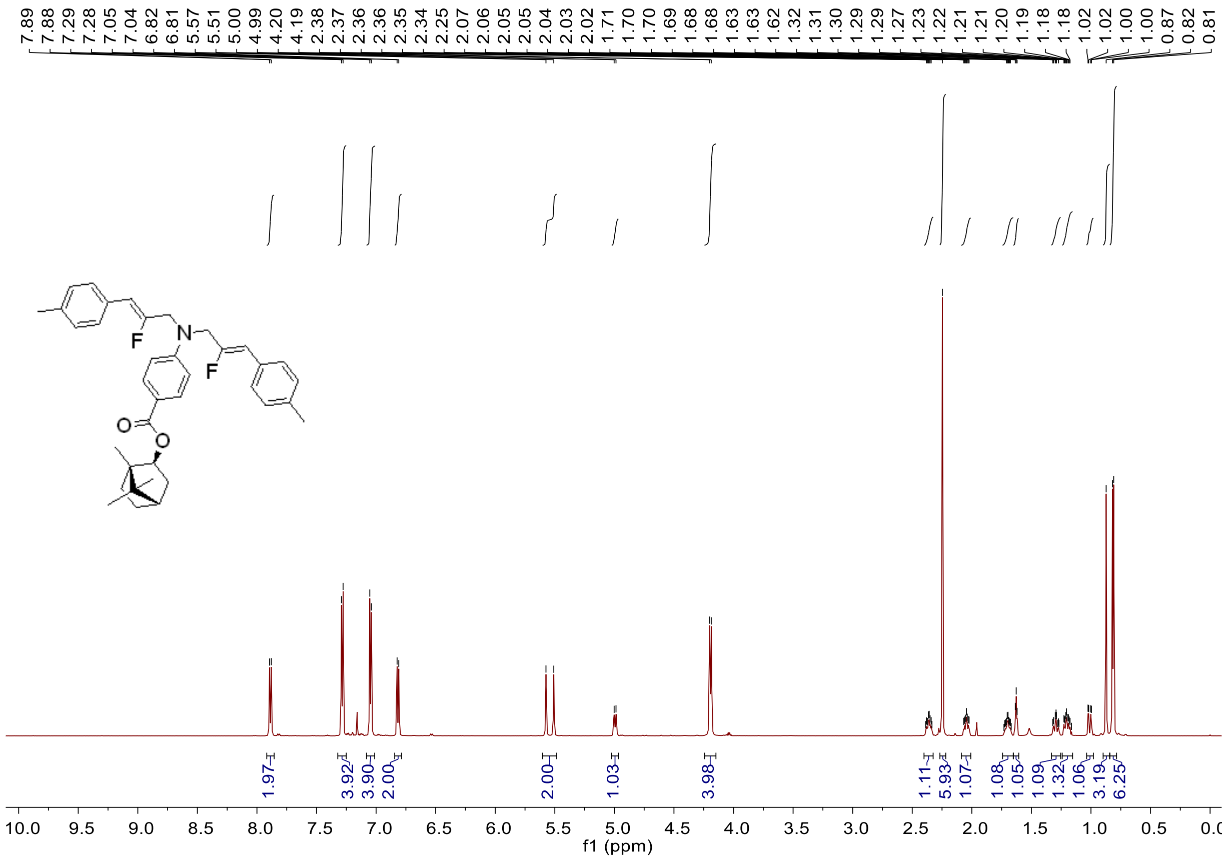

^{19}F NMR (**d28**)

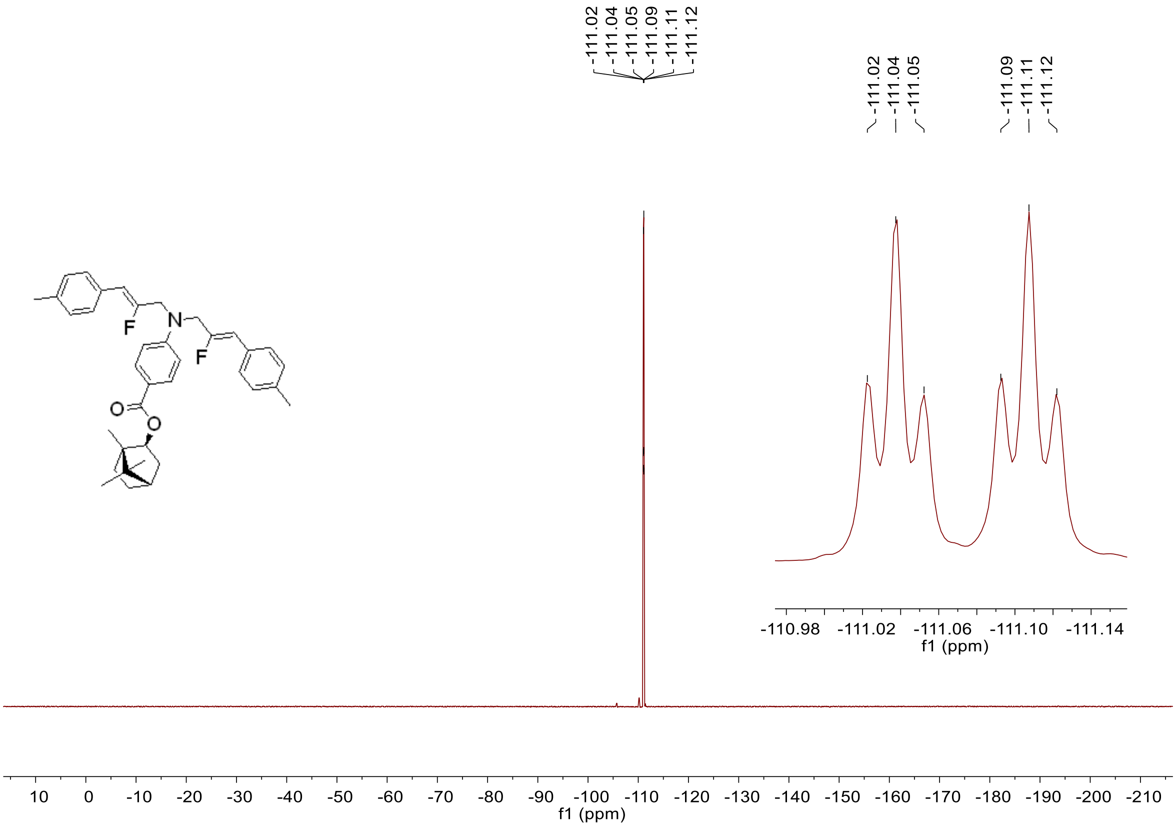

^{13}C NMR (**d28**)

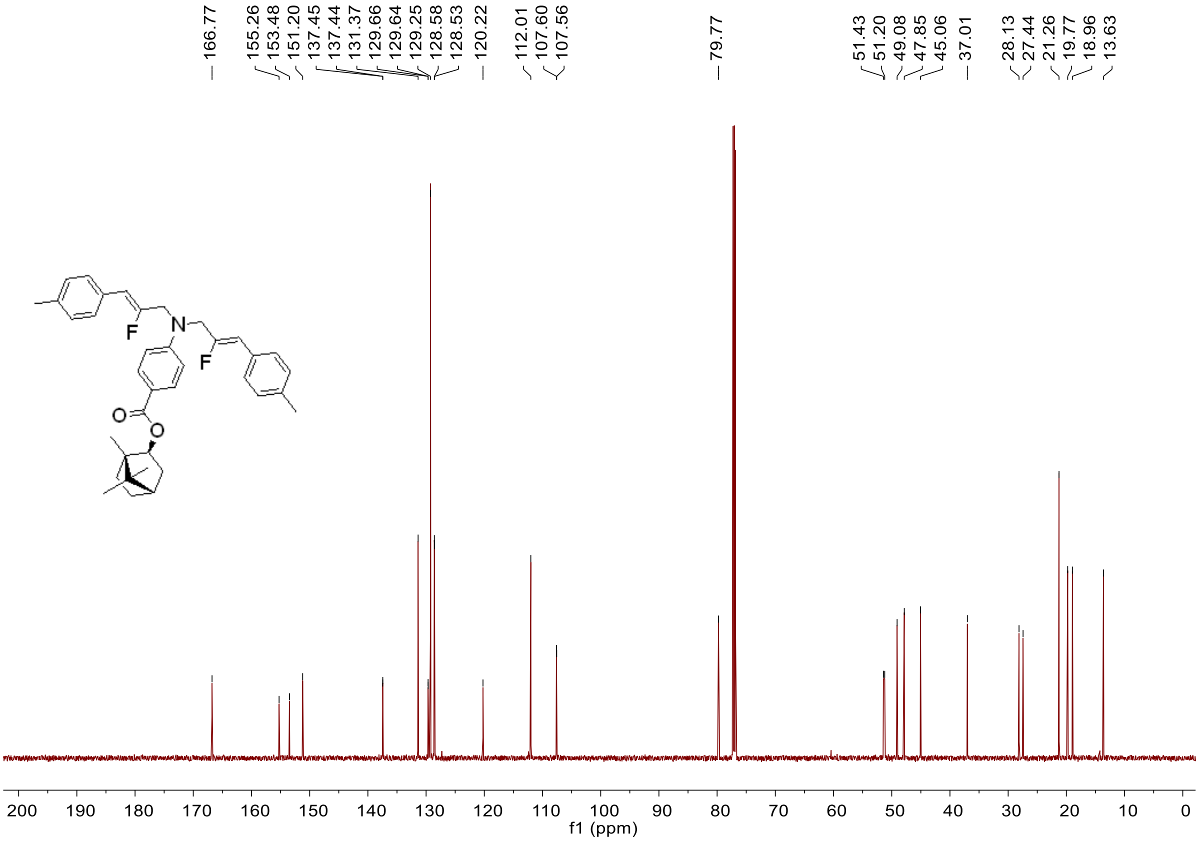

^{1}H NMR (**d29**)

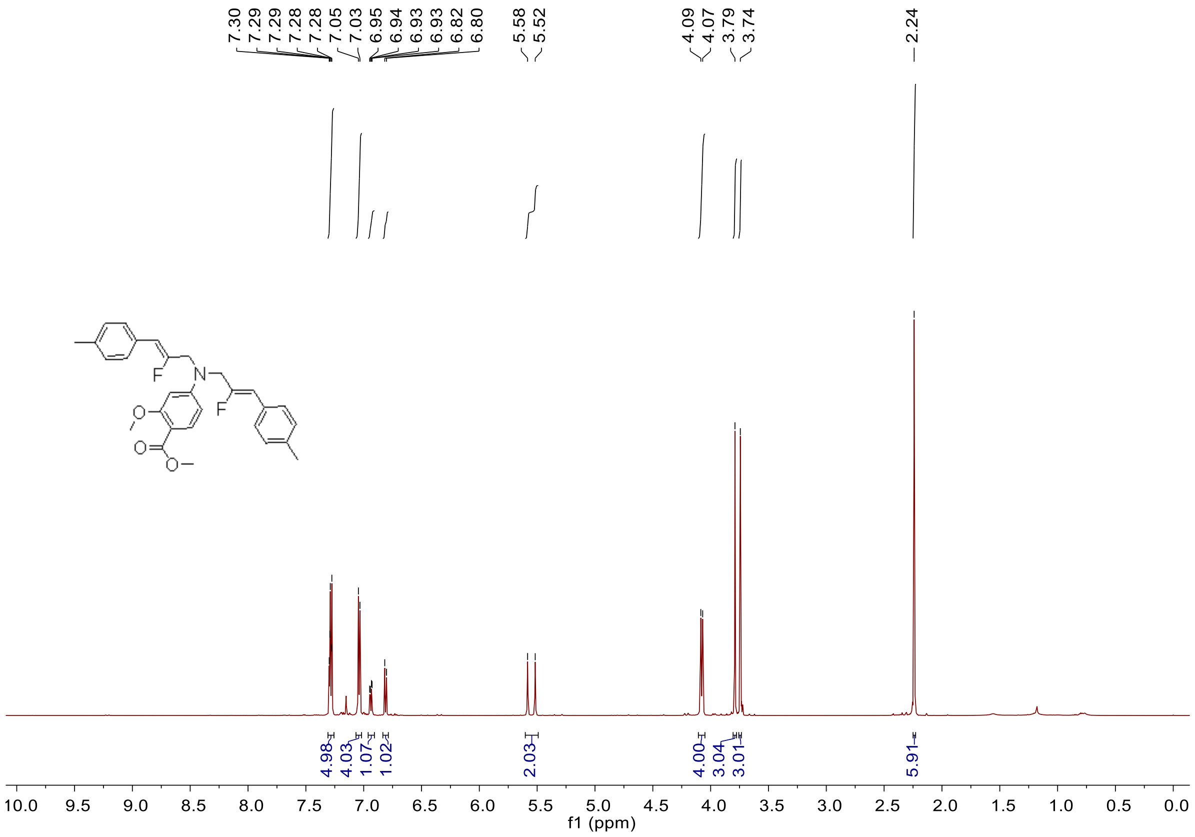

^{19}F NMR (**d29**)

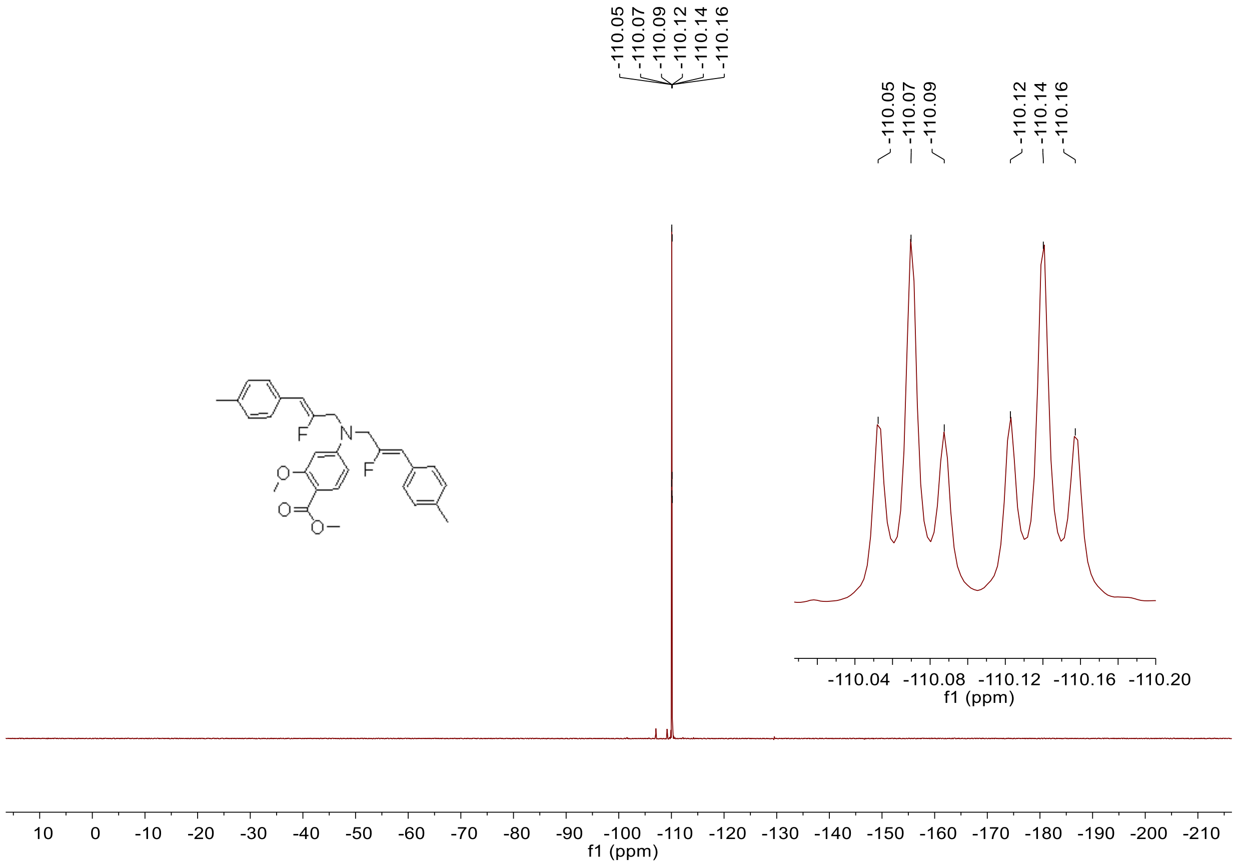

^{13}C NMR (**d29**)

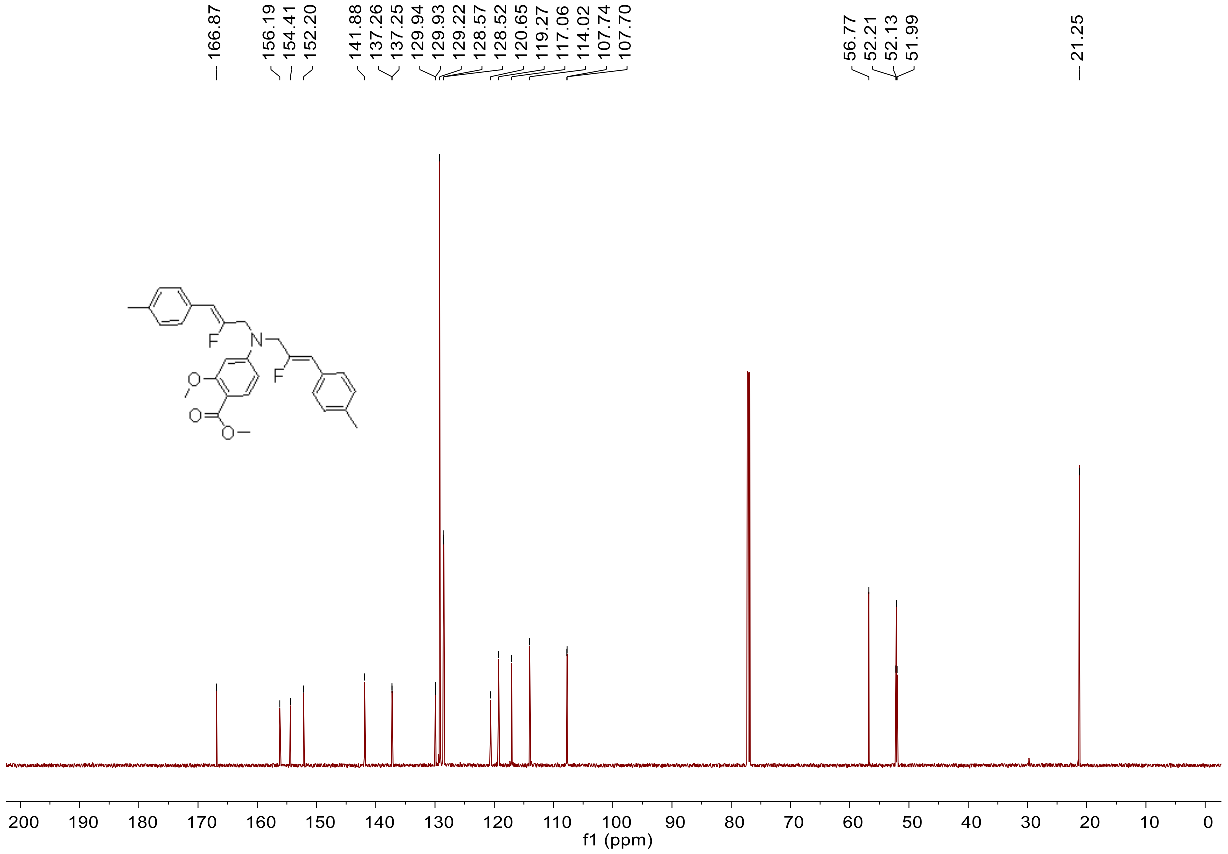

^{1}H NMR (**e1**)

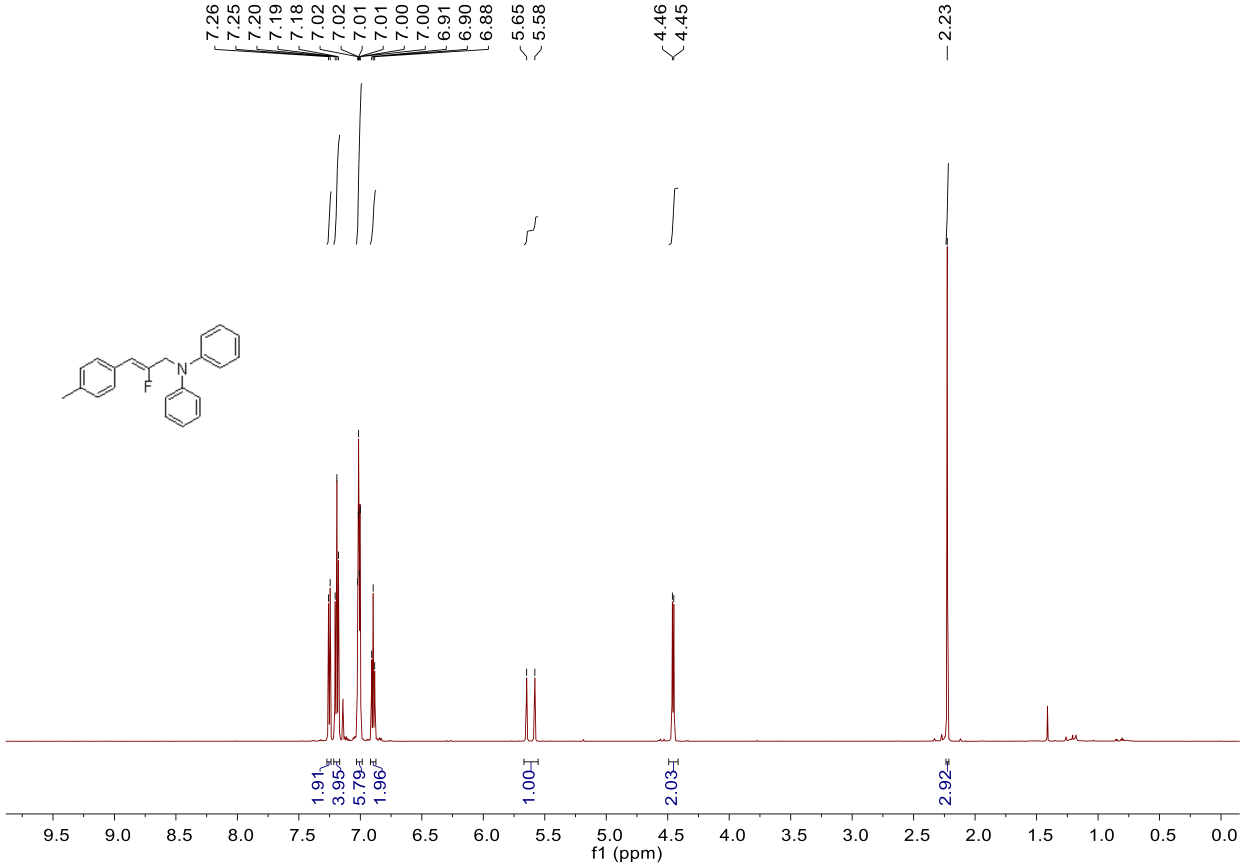

[13]F NMR (**e1**)

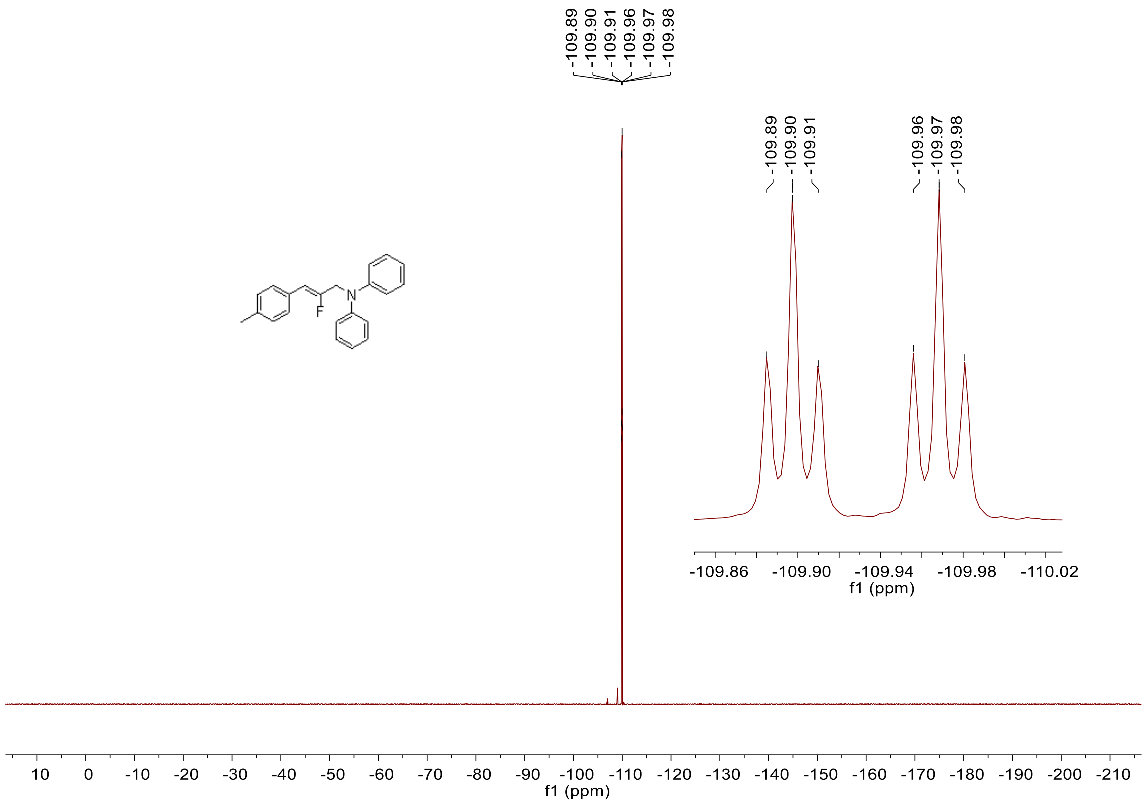

^{13}C NMR (**e1**)

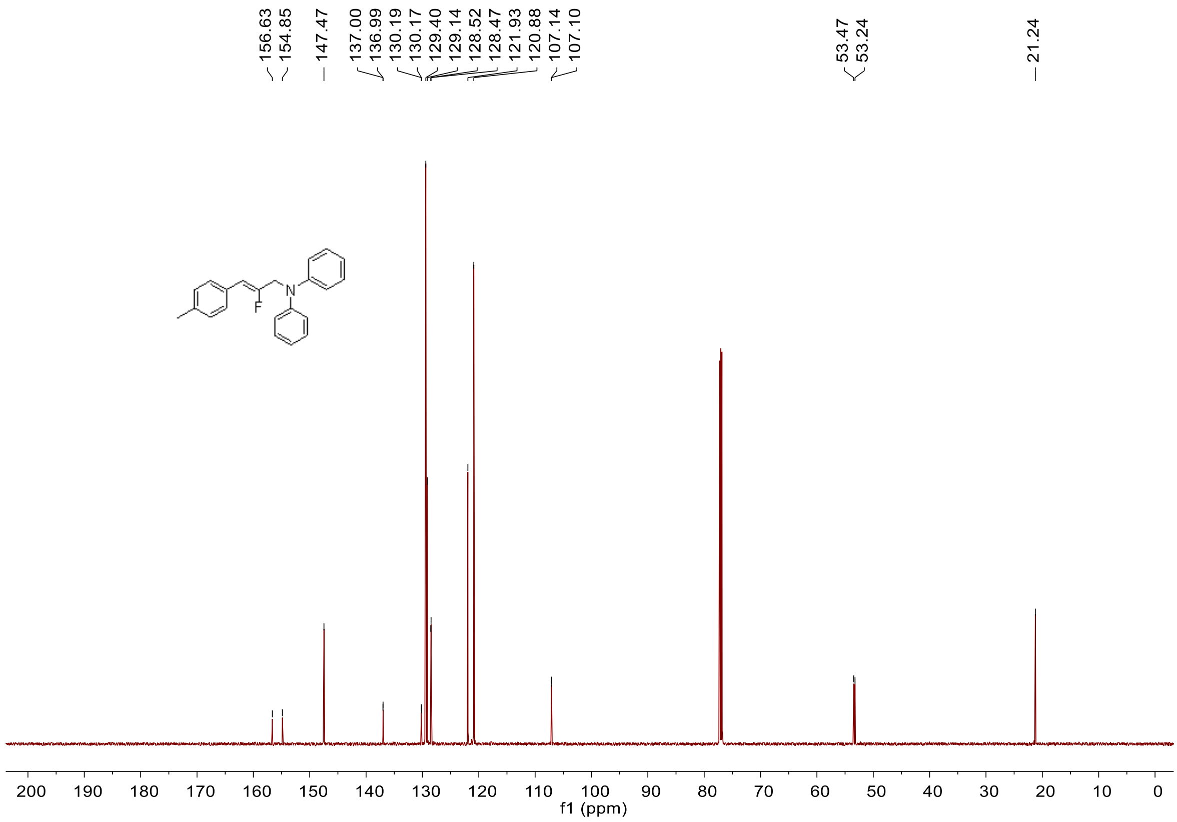

^{1}H NMR (**d30**)

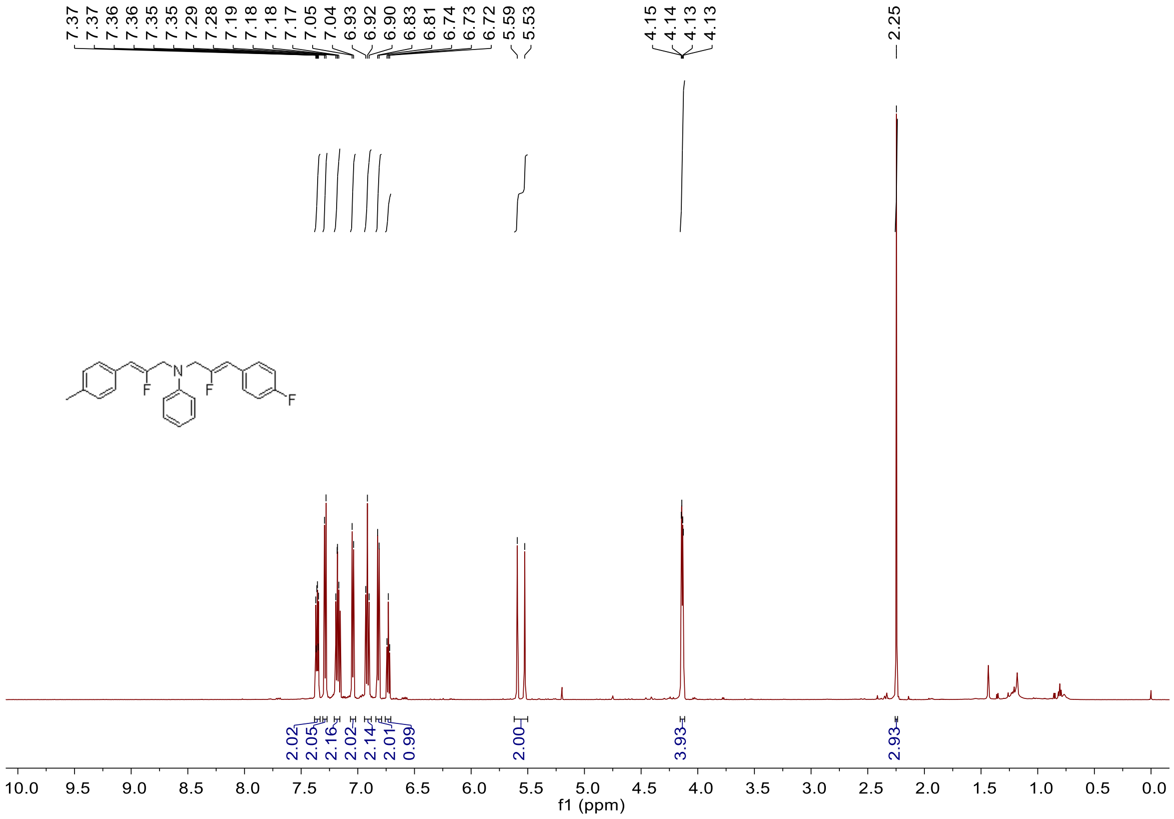

[13]F NMR (**d30**)

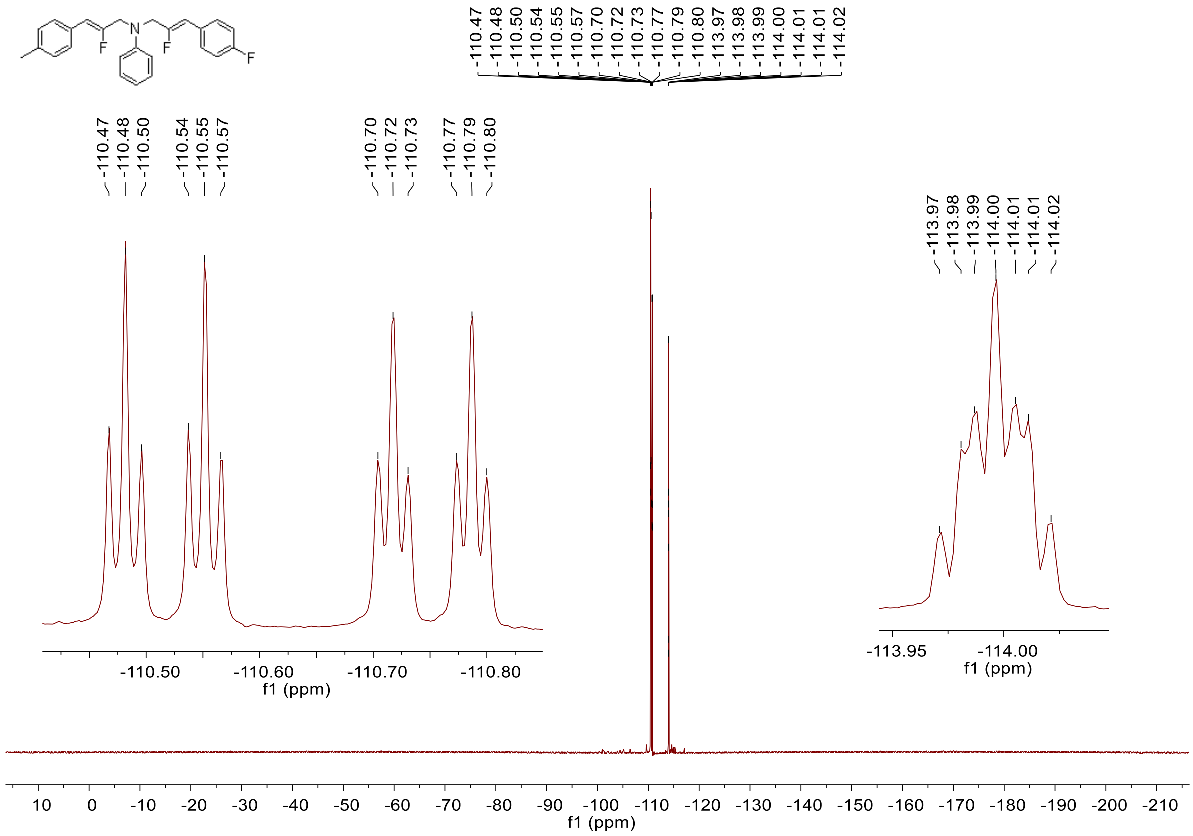

^{13}C NMR (**d30**)

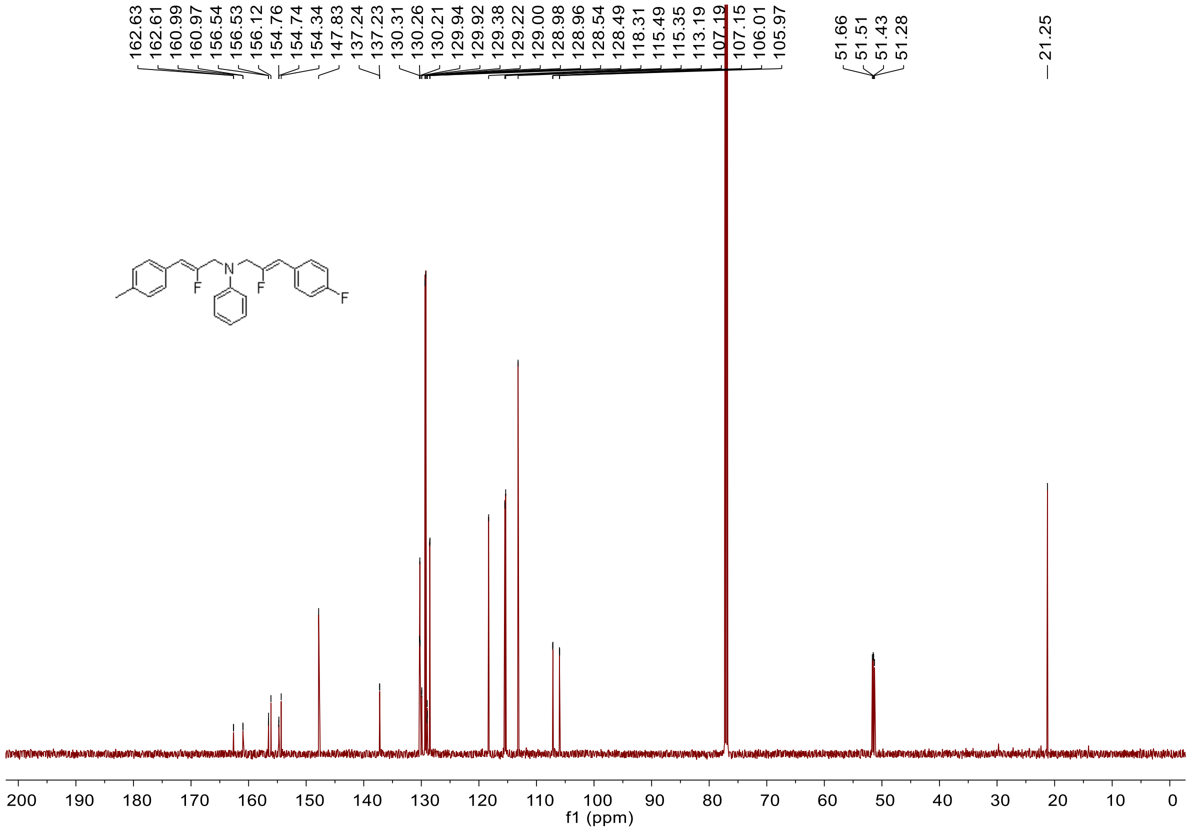
162.63
162.61
160.99
160.97
156.54
156.53
156.12
154.76
154.74
154.34
147.83
137.24
137.23
130.31
130.26
130.21
129.94
129.92
129.38
129.22
129.00
128.98
128.96
128.54
128.49
118.31
115.49
115.35
113.19
107.19
107.15
106.01
105.97
51.66
51.51
51.43
51.28
21.25
F
N
F
F
200 190 180 170 160 150 140 130 120 110 100 90 80 70 60 50 40 30 20 10 0
f1 (ppm)
